# Supplementary material for: A single-cell transcriptomic atlas of sensory-dependent gene expression in developing mouse visual cortex
Source: Development. 2025 Mar 27;152(20):dev204244. doi: 10.1242/dev.204244 (PMC12599534; doi:10.1242/dev.204244)
Supplement: Supplementary information [file develop-152-204244-s1.pdf]

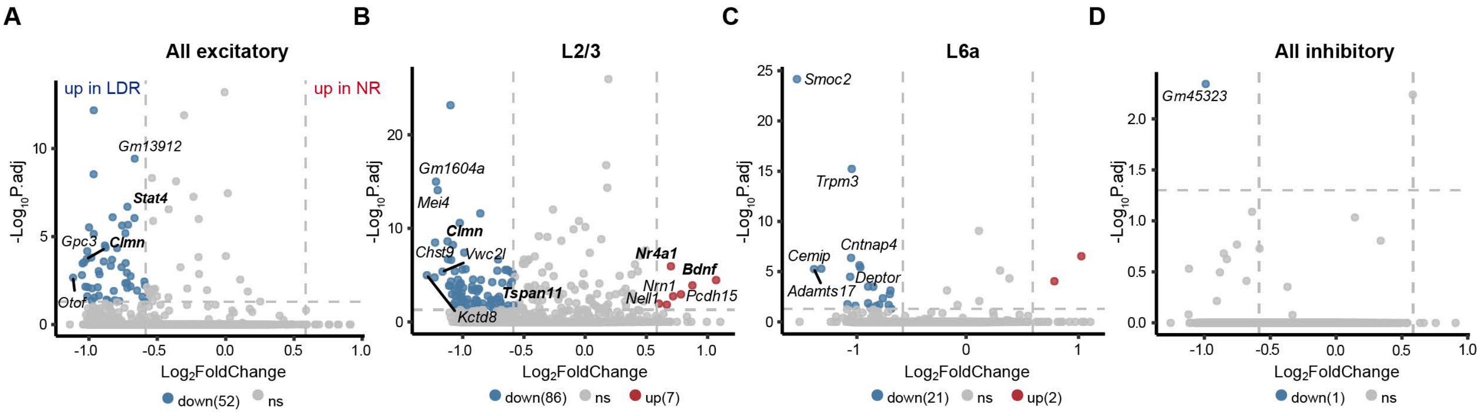

Fig. S1. Sensory deprivation upregulates a cohort of genes in excitatory neurons. (A) Volcano plot demonstrating transcripts that were significantly differentially expressed (differentially expressed genes, DEGs) in aggregated excitatory neuron clusters after LDR compared to normally reared (NR) control mice. Y-axis, negative Log(10) adjusted p value (threshold of p.adj < 0.05 indicated by dashed horizontal line). X-axis, Log(2) fold change (threshold of $log_2(1.5)$ indicated by dashed vertical lines). Red, genes that are more highly expressed in the NR condition (up in NR). Blue, genes that are more highly expressed in the LDR condition (up in LDR). (B) Volcano plot of DEGs altered by sensory deprivation in excitatory L2/3 neurons. (C) Volcano plot of DEGs altered by sensory deprivation in L6a neurons. (D) Volcano plot of DEGs altered by sensory deprivation in aggregated inhibitory clusters.

Development • Supplementary information

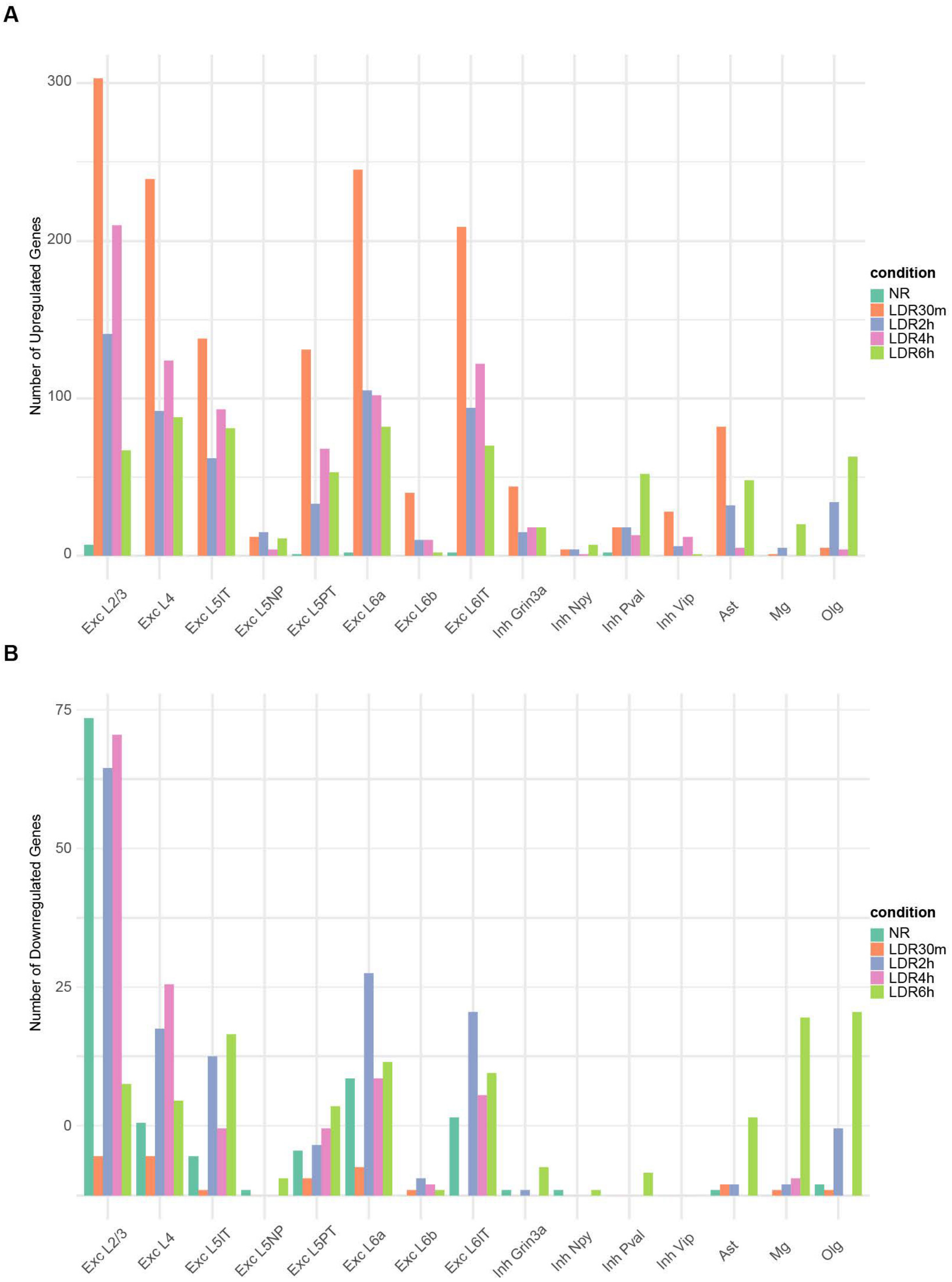

Fig. S2. Numbers of differentially expressed genes by cell type. (A) Bar graph illustrating the total number of transcripts that were upregulated following light re-exposure or in normally reared mice versus the LDR condition. Significantly upregulated transcripts met a threshold of FDR < 0.05 at a log-2 fold-change induction of at least 1.5. (B) Same as (A) but displaying genes downregulated in response to sensory experience.

Development • Supplementary information

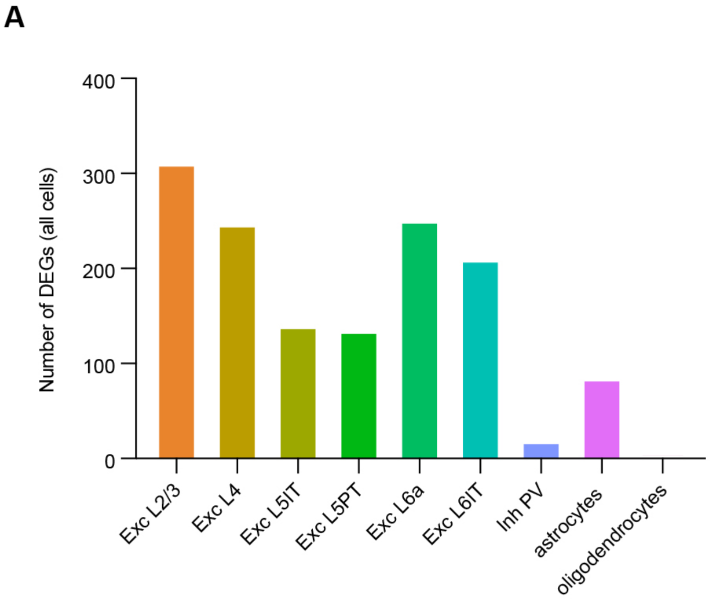

Fig. S3. Numbers of differentially expressed genes before and after downsampling. (A) Bar graph demonstrating the numbers of differentially expressed genes (DEGs) identified across the cell types for which at least 400 cells were present at each timepoint. Also see Figure S2. (B) Same as in (A) but analyzed after downsampling all cell types to 400 cells. Note the pattern of gene dysregulation is largely unaffected by downsampling.

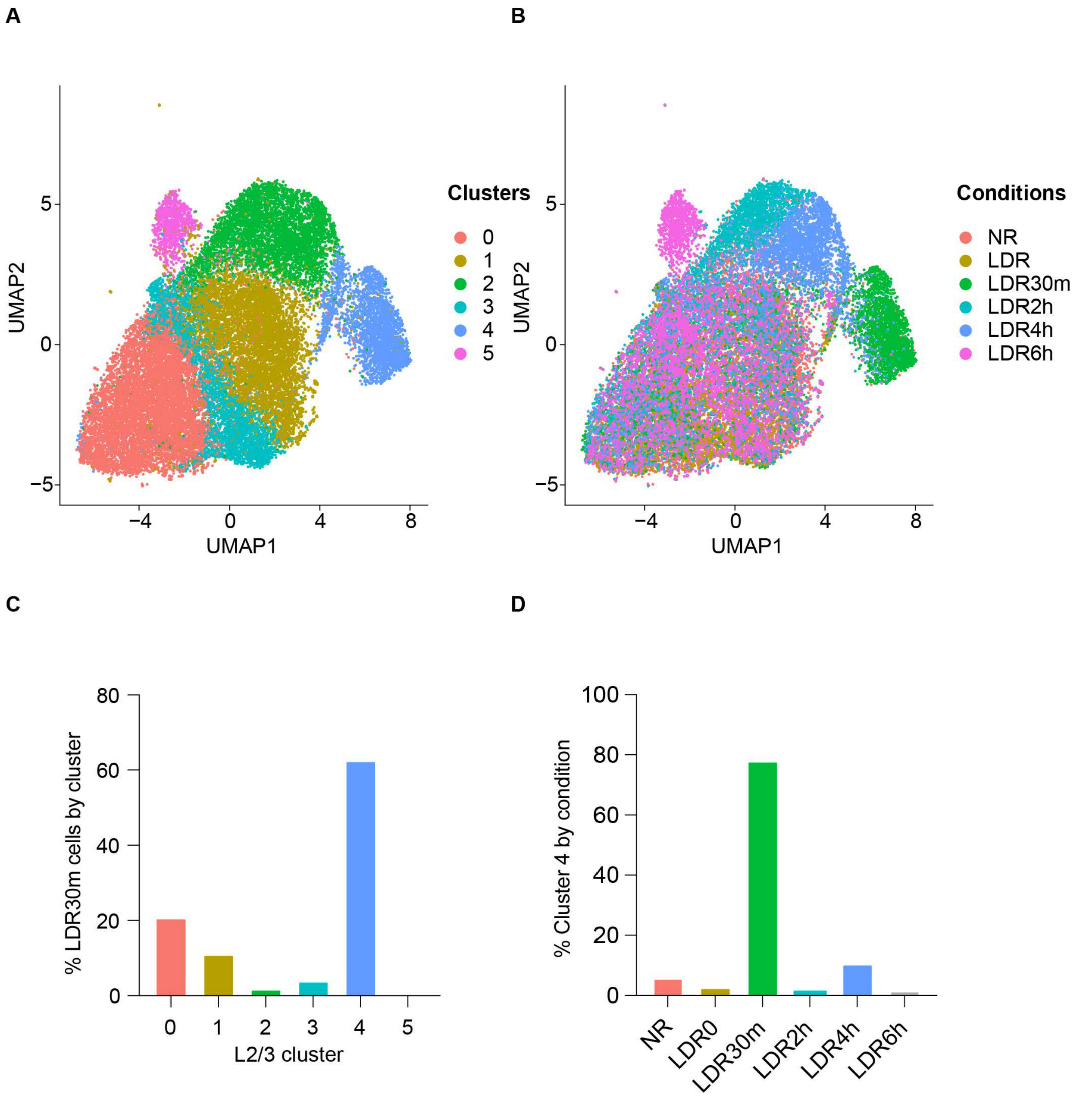

Fig. S4. Distinct transcriptomic states of L2/3 excitatory neurons following sensory stimulation. (A) UMAP plot demonstrating the transcriptomically distinct subsets of L2/3 excitatory neurons in the dataset. Colors represent distinct sub-clusters. (B) UMAP plot as in (A) but with cells colored according to timepoint. Note that cluster 4 is predominantly composed of cells from the LDR30m condition. (C) Quantification of the percentage of cells in the LDR30m condition found in each L2/3 sub-cluster. (D) The percentage of cluster 4 derived from cells of each timepoint.

Development • Supplementary information

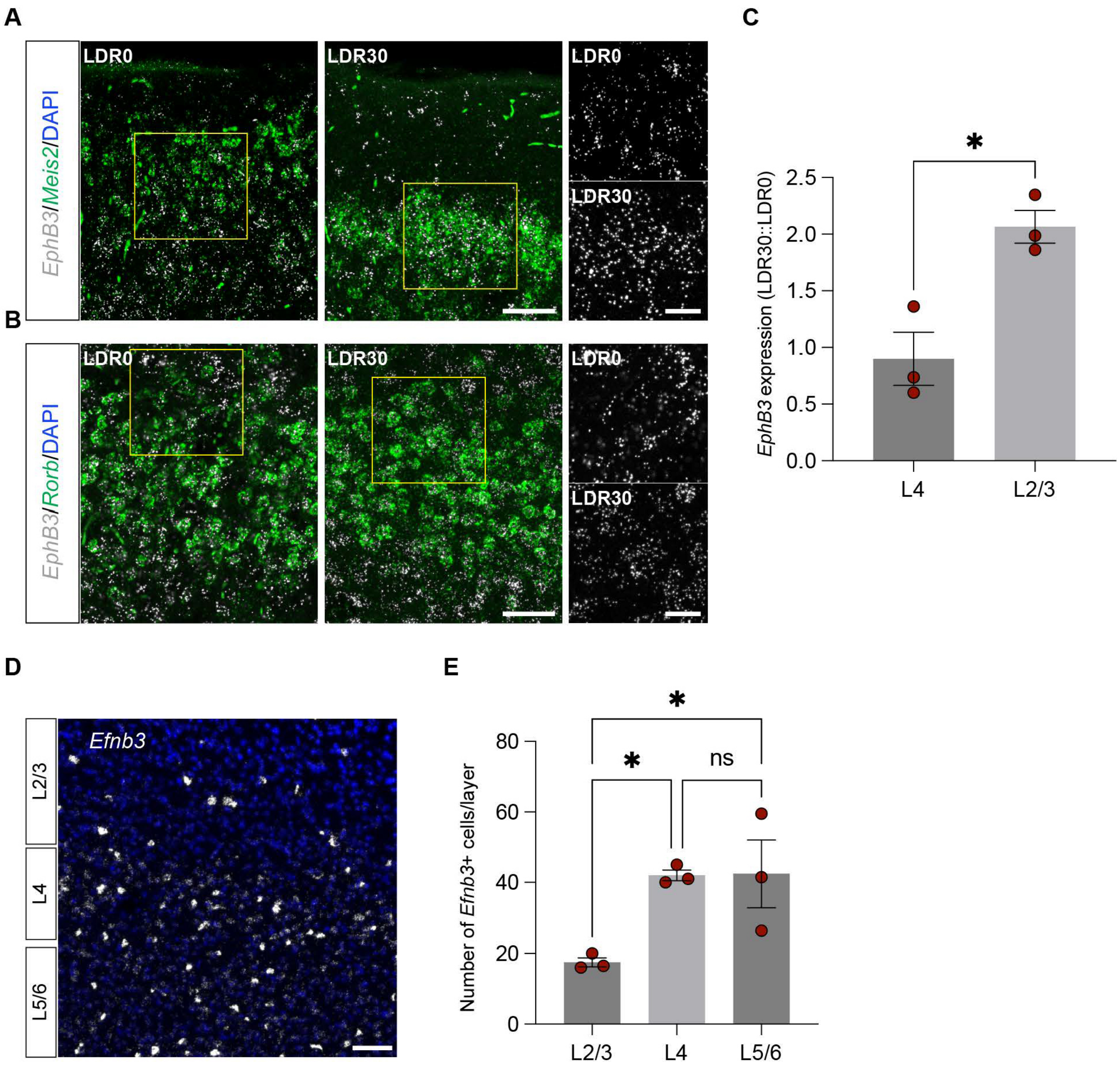

Fig. S5. Inducible *Ephb3* expression in L2/3 and its implications for synaptic signaling between upper and deeper layers of V1. (A) Example confocal images of V1 sections subjected to fluorescence *in situ* hybridization (FISH) and probed for the axon guidance receptor *Ephb3* (white) and the L2/3 neuron marker *Meis2* (green). Insets are outlined in yellow. Scale bar, 100 μm. Inset scale bar, 50 μm. (B) Same as in (A) but probed for *Ephb3* (white) alongside the L4 neuron marker *Rorb* (green). (C) Quantification of *Ephb3* expression in L4 versus L2/3 neurons based upon FISH. (D) Lower magnification (20X) confocal image of *Efnb3* (white; DAPI shown in blue), the ligand of *Ephb3*, across cortical layers. Scale bar, 100 μm. (E) Quantification of the number of cells expressing *Efnb3* by cortical layer. Statistics: For both (C) and (E), n = 3 where each replicate is one mouse. For (C), unpaired student's *t* test, *p<0.05. For (E), One-way ANOVA followed by Tukey's post tests, *p<0.05. For (C) and (E), error bars indicate mean +/- s.e.m.

Development • Supplementary information

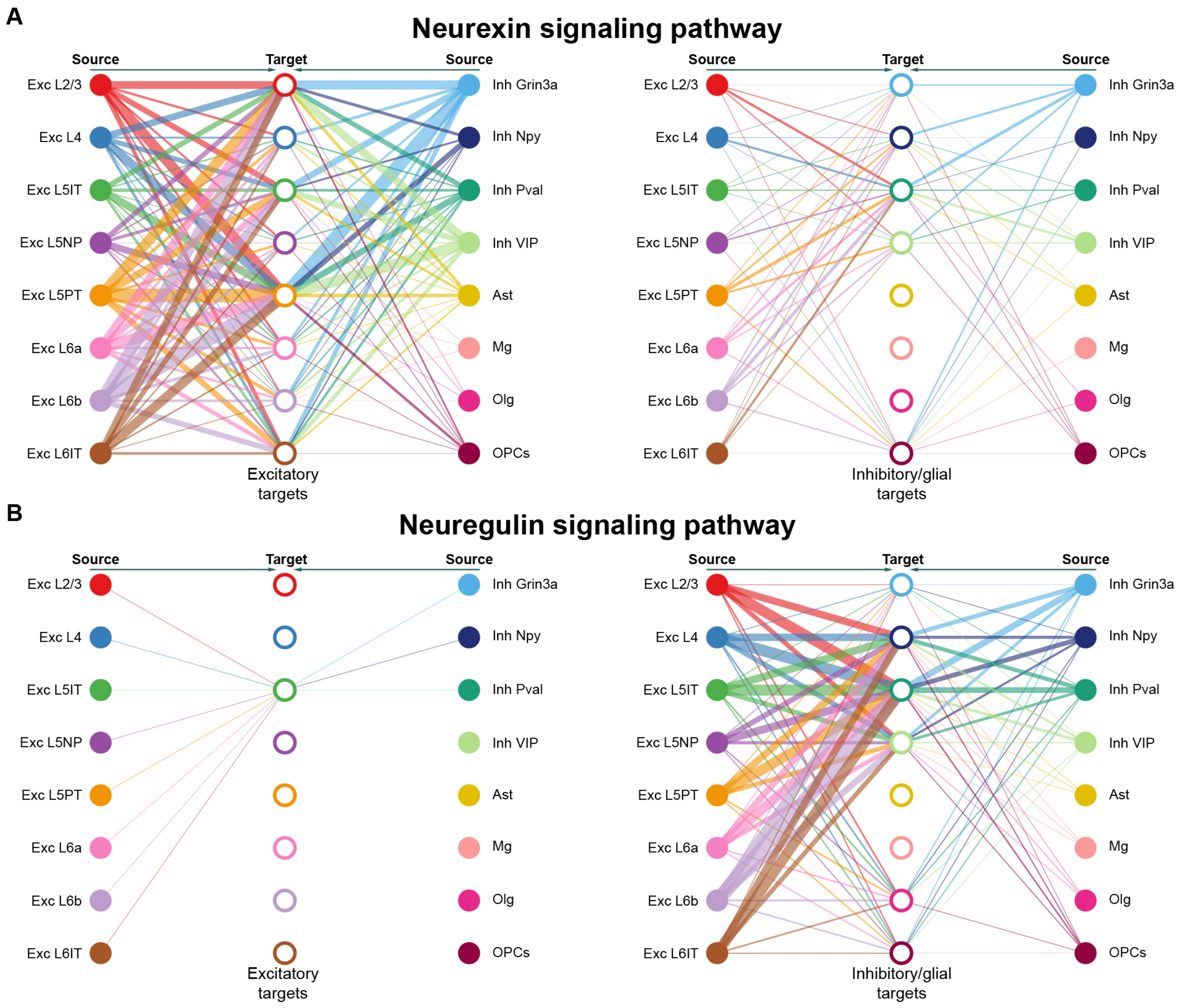

Fig. S6. Excitatory-excitatory signaling and excitatory-inhibitory signaling mediated by neurexin and neuregulin pathways, respectively. (A) Hierarchical plot showing Nrxn-mediated interactions from excitatory to excitatory neurons (left) and from excitatory to inhibitory and glial cells (right). (B) Hierarchical plot showing Nrg-mediated interactions from excitatory to excitatory neurons (left) and from excitatory to inhibitory and glial cells (right).

Development • Supplementary information

Table S1. Numbers of each cell type included in the final dataset by condition.

	NR	LDR	LDR30m	LDR2h	LDR4h	LDR6h
Astrocytes	2232	812	851	1882	842	1705
Exc L2/3	6866	4284	3555	4196	4332	4249
Exc L4	6931	3723	2679	4648	3642	4412
Exc L5IT	1460	782	828	871	866	745
Exc L5NP	666	331	298	592	358	407
Exc L5PT	1046	554	577	643	727	526
Exc L6a	3522	2031	1701	2648	2131	2227
Exc L6b	220	143	92	130	182	97
Exc L6IT	2052	1140	950	1482	1320	1166
Inh Grin3a	584	325	259	416	276	358
Inh Npy	277	199	152	258	177	209
Inh Pval	1110	529	442	778	581	678
Inh Vip	378	258	168	250	146	252
Microglia	867	577	289	681	303	617
Oligodendrocytes	1752	939	633	1407	745	1288
OPCs	586	288	30	549	136	430

Table S2. Differentially expressed genes upregulated by experience in each cell type compared to LDR.

	avg.Log2FC	p_val_adj	Cell type	Timepoint
**Dclk1**	1.005104927	2.59286240502632e-11	Ast	LDR2h
**Fth1**	1.478072999	1.71144379718721e-07	Ast	LDR2h
**mt-Co1**	1.046251365	1.128468082418e-06	Ast	LDR2h
**Sema3c**	2.074710768	1.66746164476577e-06	Ast	LDR2h
**Hes1**	1.100947362	3.69781162138515e-06	Ast	LDR2h
**Calm1**	1.007660254	1.21650964876472e-05	Ast	LDR2h
**1700054A03Rik**	1.297057354	1.34195101583624e-05	Ast	LDR2h
**Nrgn**	1.06217755	6.93868895233717e-05	Ast	LDR2h
**Cox7c**	1.005182526	0.000509913	Ast	LDR2h
**AC149090.1**	0.960703527	0.000804381	Ast	LDR2h
**Egr1**	0.972260851	0.001229564	Ast	LDR2h
**Hspa8**	0.916701022	0.001304979	Ast	LDR2h
**Rps29**	1.104721803	0.002580595	Ast	LDR2h
**Gm11867**	0.933597766	0.009749737	Ast	LDR2h
**Camk2n1**	0.770596928	0.012187811	Ast	LDR2h
**Cox8a**	0.933959631	0.013261393	Ast	LDR2h
**Ubb**	1.162325595	0.013603178	Ast	LDR2h
**BC018473**	0.818698016	0.013957106	Ast	LDR2h
**mt-Co2**	0.952486176	0.014532891	Ast	LDR2h
**Sat1**	0.839649829	0.016548614	Ast	LDR2h
**Rps8**	0.88088674	0.016851603	Ast	LDR2h
**mt-Co3**	0.938340407	0.020127042	Ast	LDR2h
**Xlr5a**	0.941789954	0.021822237	Ast	LDR2h
**Ppp1r3c**	0.861081308	0.026934436	Ast	LDR2h
**Rpl13**	0.866461443	0.033042004	Ast	LDR2h
**Rps21**	1.011452974	0.033275578	Ast	LDR2h
**Rplp1**	0.960402492	0.03391158	Ast	LDR2h
**Atp6v0b**	0.815774718	0.034044607	Ast	LDR2h
**Hsp90ab1**	0.794466287	0.036192768	Ast	LDR2h
**Egr3**	0.7647604	0.038529046	Ast	LDR2h
**Tpt1**	0.989604713	0.039136387	Ast	LDR2h
**Cox4i1**	1.043783218	0.049542499	Ast	LDR2h
**Gm47423**	2.199056484	5.72558617122393e-38	Ast	LDR30m

Development • Supplementary information

**Zswim6**	1.970590692	2.89037921886044e-36	Ast	LDR30m
**Rfx4**	1.802067669	7.93084029745543e-36	Ast	LDR30m
**Eprs**	2.332933508	1.81839881781633e-35	Ast	LDR30m
**Usp2**	2.242547131	6.47466045638821e-35	Ast	LDR30m
**Epas1**	1.861219402	7.52021381725598e-27	Ast	LDR30m
**Coq10b**	1.949785372	8.63686783206695e-24	Ast	LDR30m
**Fgfr2**	1.271896437	6.53415452163816e-23	Ast	LDR30m
**Arhgap31**	1.727804526	4.78822180869424e-22	Ast	LDR30m
**Ntrk2**	1.227549345	4.18686199422843e-20	Ast	LDR30m
**Map3k19**	1.949871652	5.15539466437653e-20	Ast	LDR30m
**Usp53**	1.43155496	1.4548883514828e-18	Ast	LDR30m
**Irs2**	1.917361197	5.38481675718863e-18	Ast	LDR30m
**Nr4a1**	1.948688532	7.79987076496933e-16	Ast	LDR30m
**Nr4a3**	2.78679556	2.89681012097004e-15	Ast	LDR30m
**Per1**	1.602487689	3.85684886630125e-15	Ast	LDR30m
**1700016P03Rik**	1.932044714	7.64703825124433e-15	Ast	LDR30m
**Cerk**	1.610591468	1.2113745318294e-14	Ast	LDR30m
**Ifrd1**	1.422959391	9.62700831787658e-14	Ast	LDR30m
**Gem**	1.858600383	1.57917819383849e-13	Ast	LDR30m
**Htra1**	1.152077987	9.57950438796773e-13	Ast	LDR30m
**Slc3a2**	1.445243593	4.81611905136386e-10	Ast	LDR30m
**Slc25a3**	1.341840502	5.02186916557438e-10	Ast	LDR30m
**Frmd6**	1.359634809	2.09942654162761e-09	Ast	LDR30m
**Sik1**	1.885923292	2.35572350004401e-09	Ast	LDR30m
**Cables1**	1.330082697	2.39976838528767e-09	Ast	LDR30m
**Emd**	1.714704301	3.92555859146371e-09	Ast	LDR30m
**Dio2**	1.140336834	4.14735396417755e-09	Ast	LDR30m
**Bpnt1**	1.469484396	4.71936219986838e-09	Ast	LDR30m
**Dhrs3**	1.386218229	1.5506732689026e-08	Ast	LDR30m
**Nr4a2**	2.503453106	1.84691362618388e-08	Ast	LDR30m
**Elovl5**	1.210187261	4.79817934278933e-08	Ast	LDR30m
**Rheb**	1.217122648	7.37549775863607e-08	Ast	LDR30m
**Gm49380**	1.339291277	2.6296956237647e-07	Ast	LDR30m
**Zhx2**	0.977659072	3.50861683890246e-07	Ast	LDR30m
**Arid5a**	1.267668326	3.64601705377581e-07	Ast	LDR30m
**Foxo1**	1.34403452	4.4687266958563e-07	Ast	LDR30m
**Hspa4**	1.194349103	5.99284037988159e-07	Ast	LDR30m
**Slco1c1**	1.13066063	6.59840263044479e-07	Ast	LDR30m
**Ago2**	1.099874863	7.47635859632828e-07	Ast	LDR30m

Development • Supplementary information

**Fosl2**	1.635751327	2.73494365209197e-06	Ast	LDR30m
**Pde7b**	0.854018321	4.59441540931001e-06	Ast	LDR30m
**Slc7a5**	1.214465599	5.28107822751063e-06	Ast	LDR30m
**Zfp516**	1.07857397	8.53328124653048e-06	Ast	LDR30m
**Kcnn2**	0.825485438	9.1303891852081e-06	Ast	LDR30m
**Ezr**	1.053571157	1.30784110825423e-05	Ast	LDR30m
**Ddah1**	0.858810476	1.8849814472654e-05	Ast	LDR30m
**Hdac4**	1.022949835	2.17066251433451e-05	Ast	LDR30m
**Dapk2**	1.112996485	2.78856966286269e-05	Ast	LDR30m
**Fam20a**	0.809194634	5.15659217511223e-05	Ast	LDR30m
**Ccnl1**	1.055454299	5.51646776572887e-05	Ast	LDR30m
**9330159F19Rik**	0.864667437	6.05442478301954e-05	Ast	LDR30m
**Homer1**	1.922302917	6.3461421683008e-05	Ast	LDR30m
**Fos**	1.3898668	7.23825815127036e-05	Ast	LDR30m
**Txndc11**	0.955011137	0.000109354	Ast	LDR30m
**Chp1**	0.96756988	0.000247483	Ast	LDR30m
**Sik2**	0.995607148	0.000266507	Ast	LDR30m
**Tle1**	0.862586517	0.000361067	Ast	LDR30m
**Egr3**	1.120411806	0.000361882	Ast	LDR30m
**Fam13c**	0.72949023	0.000408154	Ast	LDR30m
**Pcsk1**	1.091088573	0.000418282	Ast	LDR30m
**Mxi1**	0.961943987	0.000528417	Ast	LDR30m
**Gm6225**	0.987755265	0.000582858	Ast	LDR30m
**Aff1**	0.878406948	0.000617459	Ast	LDR30m
**Junb**	1.047374822	0.000754816	Ast	LDR30m
**Cdh20**	0.778589088	0.000951182	Ast	LDR30m
**Gm47283**	0.987333154	0.001238413	Ast	LDR30m
**Ptbp1**	0.960908554	0.001461985	Ast	LDR30m
**Vps37b**	1.045407254	0.001489715	Ast	LDR30m
**Lhfp**	0.828976838	0.003900518	Ast	LDR30m
**Ndfip1**	0.806030827	0.005921943	Ast	LDR30m
**Irak2**	0.859454709	0.007227903	Ast	LDR30m
**Pmepa1**	1.037583942	0.007751687	Ast	LDR30m
**Paqr8**	0.621729691	0.009743084	Ast	LDR30m
**Trmt61b**	0.91176893	0.010853542	Ast	LDR30m
**Ptma**	1.046452617	0.011944806	Ast	LDR30m
**Csdc2**	1.02258048	0.013954855	Ast	LDR30m
**Crem**	0.928049371	0.014984143	Ast	LDR30m
**Dleu2**	0.763550613	0.019398426	Ast	LDR30m

**Jdp2**	0.954936681	0.020521787	Ast	LDR30m
**Unc45a**	0.929767634	0.025138584	Ast	LDR30m
**Cpe**	0.597820002	0.033518517	Ast	LDR30m
**1700016P03Rik**	1.307326228	7.9721734538881e-08	Ast	LDR4h
**Gm15398**	1.371636178	4.04142095589408e-07	Ast	LDR4h
**Mpped2**	0.769545606	0.000780119	Ast	LDR4h
**Homer1**	1.564667398	0.004336752	Ast	LDR4h
**Sgsm1**	0.940593739	0.033106286	Ast	LDR4h
**Fth1**	1.523941708	3.96550338538503e-18	Ast	LDR6h
**mt-Co1**	1.219813834	1.68092302150819e-15	Ast	LDR6h
**Apoe**	1.208097713	3.6245468264062e-14	Ast	LDR6h
**Cst3**	0.979672482	1.82975828013045e-09	Ast	LDR6h
**Srsf7**	1.073441239	6.55020092774802e-09	Ast	LDR6h
**Ubb**	1.248140669	9.52673226607478e-09	Ast	LDR6h
**Gm35188**	0.946964713	5.63804633771799e-08	Ast	LDR6h
**Sema3c**	1.227268854	6.3266538763737e-08	Ast	LDR6h
**mt-Co3**	1.041217442	2.80940865942194e-07	Ast	LDR6h
**Ckb**	0.93171411	1.59790675595579e-06	Ast	LDR6h
**Gm48747**	1.018948357	2.26619360251575e-06	Ast	LDR6h
**Ptgds**	1.018241777	8.73353409033946e-06	Ast	LDR6h
**Calm1**	0.926521816	1.01872574483944e-05	Ast	LDR6h
**Cox8a**	0.991343672	1.15519815597317e-05	Ast	LDR6h
**Atp6v0c**	0.988113324	1.3473477426756e-05	Ast	LDR6h
**Tmsb4x**	1.196358017	1.40436673276801e-05	Ast	LDR6h
**Nrgn**	0.978680854	2.29464015189945e-05	Ast	LDR6h
**Rps8**	0.992447547	3.65301972316312e-05	Ast	LDR6h
**Rplp1**	0.985564298	0.00013147	Ast	LDR6h
**Cox4i1**	1.028851742	0.000134568	Ast	LDR6h
**Rpl13**	0.997209523	0.000177124	Ast	LDR6h
**Camk2n1**	0.844783424	0.00018069	Ast	LDR6h
**Hes5**	0.996100304	0.000287989	Ast	LDR6h
**Cox7c**	1.109716188	0.000299894	Ast	LDR6h
**Prpf4b**	0.81918171	0.000410196	Ast	LDR6h
**Arhgef19**	0.933593154	0.000416668	Ast	LDR6h
**Pantr2**	0.871385394	0.000681186	Ast	LDR6h
**Tpt1**	0.932268354	0.000737105	Ast	LDR6h
**Shisa4**	1.066372889	0.000769879	Ast	LDR6h
**Rpl6**	0.941735019	0.000789866	Ast	LDR6h
**Slc25a4**	0.879458226	0.000978117	Ast	LDR6h

Development • Supplementary information

**mt-Co2**	0.843440817	0.00221202	Ast	LDR6h
**Oaz1**	0.886177216	0.002963724	Ast	LDR6h
**Sycp2**	0.813142873	0.003411581	Ast	LDR6h
**Etv4**	0.869742291	0.004590306	Ast	LDR6h
**5330438D12Rik**	0.787433716	0.005290371	Ast	LDR6h
**Nnat**	1.139134621	0.005991447	Ast	LDR6h
**Ndufb9**	0.84345174	0.008261352	Ast	LDR6h
**6430590A07Rik**	0.798066475	0.010331484	Ast	LDR6h
**Fjx1**	0.827061448	0.011332737	Ast	LDR6h
**Hes1**	0.93384697	0.012620919	Ast	LDR6h
**Pcsk1n**	0.804430803	0.01432684	Ast	LDR6h
**mt-Cytb**	0.9877612	0.014475847	Ast	LDR6h
**Sycp3**	0.806075416	0.018398708	Ast	LDR6h
**Rpl27a**	0.780435842	0.02771705	Ast	LDR6h
**Ppia**	0.847946633	0.032227047	Ast	LDR6h
**Tmed9**	0.796002274	0.042974899	Ast	LDR6h
**Cox7b**	0.822204176	0.043809546	Ast	LDR6h
**Sema3c**	2.692929287	3.8991961218296e-08	Mg	LDR2h
**1700054A03Rik**	2.099099994	0.00530496	Mg	LDR2h
**Cox4i1**	1.667535352	0.01618538	Mg	LDR2h
**Selenow**	1.633614594	0.03661475	Mg	LDR2h
**Fth1**	1.585910052	0.038155906	Mg	LDR2h
**1700016P03Rik**	1.748545843	0.010669894	Mg	LDR30m
**Fth1**	1.687257884	1.75491509029928e-09	Mg	LDR6h
**C1qa**	1.495849688	4.01484097538958e-07	Mg	LDR6h
**Apoe**	1.581331769	1.19918312537293e-06	Mg	LDR6h
**Nrgn**	1.432205603	5.9812184207433e-05	Mg	LDR6h
**Srsf7**	1.272421933	7.78131152439347e-05	Mg	LDR6h
**Gm35188**	0.966266507	0.00022212	Mg	LDR6h
**Gm46367**	1.106551566	0.000644887	Mg	LDR6h
**Lysmd4**	0.931032225	0.002544187	Mg	LDR6h
**Cox4i1**	1.397785586	0.004407815	Mg	LDR6h
**Malat1**	1.104410881	0.00586122	Mg	LDR6h
**mt-Co3**	0.854542489	0.005933671	Mg	LDR6h
**Ubb**	1.427556641	0.007721327	Mg	LDR6h
**Sema3c**	1.547575285	0.010416619	Mg	LDR6h
**mt-Co2**	0.993534209	0.013414447	Mg	LDR6h
**H3f3b**	1.371745693	0.019248278	Mg	LDR6h
**mt-Co1**	0.908578198	0.02379979	Mg	LDR6h

Development • Supplementary information

**Atp6v0b**	1.019106282	0.034292732	Mg	LDR6h
**Gm20275**	1.222041703	0.03616679	Mg	LDR6h
**Selenow**	1.429998572	0.038342333	Mg	LDR6h
**Rpl9**	1.413208583	0.046769881	Mg	LDR6h
**Atp1b1**	1.137716835	7.84658788133504e-09	Olg	LDR2h
**mt-Co1**	1.192142613	2.55652227147787e-08	Olg	LDR2h
**mt-Co3**	1.161651068	1.3335940183224e-06	Olg	LDR2h
**Calm1**	1.154830843	3.05461050635277e-06	Olg	LDR2h
**Egr1**	1.006986499	1.98379267173541e-05	Olg	LDR2h
**1700054A03Rik**	1.189618584	3.85714996932062e-05	Olg	LDR2h
**Hspa8**	0.953851615	9.98075201224027e-05	Olg	LDR2h
**Ubb**	1.160486316	0.000390607	Olg	LDR2h
**Ptgds**	0.978009582	0.000435351	Olg	LDR2h
**AC149090.1**	1.01860918	0.000499744	Olg	LDR2h
**Nrgn**	0.925227235	0.000977474	Olg	LDR2h
**Tmsb4x**	1.160936861	0.001056451	Olg	LDR2h
**Gm43376**	0.992875594	0.001388664	Olg	LDR2h
**mt-Co2**	1.083493555	0.001998769	Olg	LDR2h
**Cox4i1**	1.051610521	0.002079639	Olg	LDR2h
**Mt1**	0.934812192	0.002417199	Olg	LDR2h
**Sema3c**	1.716477063	0.003248199	Olg	LDR2h
**Chgb**	0.900091541	0.004055849	Olg	LDR2h
**Atp6v0b**	0.778386013	0.00445382	Olg	LDR2h
**Ckb**	0.983178612	0.005308404	Olg	LDR2h
**Rpl6**	0.962571873	0.005912372	Olg	LDR2h
**Camk2n1**	0.758022265	0.006523875	Olg	LDR2h
**Tpt1**	1.020503381	0.00822669	Olg	LDR2h
**Sparcl1**	0.882757545	0.009820767	Olg	LDR2h
**Rpl37**	0.989328868	0.012979954	Olg	LDR2h
**Rpl18a**	0.751838197	0.01539575	Olg	LDR2h
**BC018473**	0.707415265	0.015703619	Olg	LDR2h
**Rplp1**	0.862584485	0.015740678	Olg	LDR2h
**Fau**	0.811168364	0.01644437	Olg	LDR2h
**Atxn7l3b**	0.763707832	0.022993345	Olg	LDR2h
**Ptprn**	0.831366739	0.02355868	Olg	LDR2h
**Rpl32**	0.840485262	0.03036269	Olg	LDR2h
**Egr3**	0.710206973	0.034705728	Olg	LDR2h
**Gm11867**	0.694801358	0.042198854	Olg	LDR2h
**Nr4a3**	1.144509519	2.22873106172014e-05	Olg	LDR30m

Development • Supplementary information

**1700016P03Rik**	1.946222814	0.005469544	Olg	LDR30m
**Gm47423**	0.950780789	0.018014126	Olg	LDR30m
**Zdbf2**	0.857726561	0.019797205	Olg	LDR30m
**Egr3**	0.868066324	0.024106874	Olg	LDR30m
**Spred2**	0.856496151	0.015047833	Olg	LDR4h
**Sgsm1**	0.841256699	0.029777989	Olg	LDR4h
**Ntrk2**	0.915063532	0.044189832	Olg	LDR4h
**Iqgap2**	0.882543446	0.045190958	Olg	LDR4h
**Fth1**	1.783567399	1.90869802528269e-29	Olg	LDR6h
**Apoe**	1.433037679	1.24782478688194e-17	Olg	LDR6h
**Ptgds**	1.34788026	3.57635914427448e-17	Olg	LDR6h
**mt-Co1**	1.265104295	8.42750961186686e-17	Olg	LDR6h
**Srsf7**	1.185869802	1.10293785227163e-12	Olg	LDR6h
**mt-Co3**	1.170866956	1.08995478749077e-11	Olg	LDR6h
**Gm42413**	1.190819146	2.22787806711761e-10	Olg	LDR6h
**Calm1**	1.065078908	2.31316324562245e-09	Olg	LDR6h
**4930419G24Rik**	1.153334252	7.41822893138847e-09	Olg	LDR6h
**Cox4i1**	1.104243978	1.58709070933539e-08	Olg	LDR6h
**Cst3**	1.020278729	2.09512126372078e-07	Olg	LDR6h
**Ubb**	1.095609333	2.56266182107764e-07	Olg	LDR6h
**Tubb4a**	0.949263888	2.96205365435137e-07	Olg	LDR6h
**Gm37459**	1.106132675	4.97759806374734e-07	Olg	LDR6h
**Atp1b1**	0.991036473	2.10891841104792e-06	Olg	LDR6h
**Tmsb4x**	1.03885282	2.55940877722243e-06	Olg	LDR6h
**mt-Co2**	0.978777611	5.39005171179462e-06	Olg	LDR6h
**Slc38a2**	0.949513592	7.96973659548188e-06	Olg	LDR6h
**H3f3b**	0.927735093	1.33098512712827e-05	Olg	LDR6h
**Ckb**	0.91496414	1.90168387526215e-05	Olg	LDR6h
**Gm19522**	0.982382062	5.31482637358089e-05	Olg	LDR6h
**Trim35**	0.835977581	0.000167245	Olg	LDR6h
**Eif1**	0.92221787	0.000253743	Olg	LDR6h
**3110021N24Rik**	0.924597607	0.000298286	Olg	LDR6h
**Cox8a**	0.862832729	0.000375756	Olg	LDR6h
**Rpl13**	0.887307945	0.000513461	Olg	LDR6h
**5330438D12Rik**	0.830700562	0.000669391	Olg	LDR6h
**Cck**	0.930789545	0.000729284	Olg	LDR6h
**Aplp1**	0.784672665	0.000853148	Olg	LDR6h
**Atxn7l3b**	0.829310044	0.000854566	Olg	LDR6h
**Pla2g3**	0.886051723	0.00086475	Olg	LDR6h

**Rpl6**	0.867163948	0.000980889	Olg	LDR6h
**Tpt1**	0.881015134	0.001038255	Olg	LDR6h
**Hspa8**	0.839841811	0.001560462	Olg	LDR6h
**mt-Cytb**	0.86960595	0.001715182	Olg	LDR6h
**Gm36975**	0.676860569	0.002169497	Olg	LDR6h
**Pcsk1n**	0.811161046	0.002509108	Olg	LDR6h
**Nrgn**	0.796513487	0.002737184	Olg	LDR6h
**Rpl27a**	0.865644789	0.00397	Olg	LDR6h
**Gm13905**	0.853463184	0.004422943	Olg	LDR6h
**Gm48678**	0.805306654	0.007238307	Olg	LDR6h
**Atp6v0c**	0.742188772	0.007363508	Olg	LDR6h
**Hapln2**	0.816978147	0.00791019	Olg	LDR6h
**Rps5**	0.74347472	0.00902702	Olg	LDR6h
**Cox7c**	0.860298619	0.009093432	Olg	LDR6h
**Rpl9**	0.841185189	0.010608844	Olg	LDR6h
**Eid1**	0.775891703	0.0149515	Olg	LDR6h
**Rpl37**	0.810561671	0.01595237	Olg	LDR6h
**Rpl41**	0.864786072	0.01628655	Olg	LDR6h
**4930511M06Rik**	0.787696045	0.018153022	Olg	LDR6h
**Jund**	0.777047043	0.021200271	Olg	LDR6h
**Bmyc**	0.790349922	0.023220226	Olg	LDR6h
**Rplp0**	0.648118421	0.023655898	Olg	LDR6h
**Atp6v0b**	0.688472257	0.024099053	Olg	LDR6h
**Neat1**	0.689858789	0.024300145	Olg	LDR6h
**Sema3c**	0.785442293	0.026260372	Olg	LDR6h
**Xrcc6**	0.738250805	0.032382691	Olg	LDR6h
**Rpl37a**	0.71733665	0.032465527	Olg	LDR6h
**Sparcl1**	0.785495052	0.033299463	Olg	LDR6h
**Rpl18a**	0.690279228	0.03428736	Olg	LDR6h
**Rpl32**	0.719273194	0.035344213	Olg	LDR6h
**mt-Nd2**	0.822948765	0.038163174	Olg	LDR6h
**Cfl2**	0.73152077	0.049139473	Olg	LDR6h
**Fosb**	0.603493795	8.2861136925174e-64	Exc	LDR2h
**C1ql3**	1.062848624	1.60265217813131e-47	Exc	LDR2h
**Gm37229**	0.709367269	1.18434308463583e-44	Exc	LDR2h
**Nptx2**	1.028465501	2.84522344467821e-43	Exc	LDR2h
**Egr3**	0.931663343	1.20132541181114e-42	Exc	LDR2h
**Rnd3**	0.800618115	3.69896469724626e-34	Exc	LDR2h
**Grasp**	0.878389799	8.0070616312507e-33	Exc	LDR2h

Development • Supplementary information

**Scg2**	0.799233663	6.85949990437288e-32	Exc	LDR2h
**Gadd45g**	0.60358203	2.08097718864321e-31	Exc	LDR2h
**Ptprn**	1.389400582	3.61675603669208e-29	Exc	LDR2h
**Gm34544**	1.192223764	2.68962236932169e-28	Exc	LDR2h
**Bdnf**	1.08090958	6.04009058369159e-27	Exc	LDR2h
**Inhba**	0.794688013	1.28212164957588e-25	Exc	LDR2h
**mt-Co3**	1.432188329	6.41279428961178e-25	Exc	LDR2h
**Npas4**	1.052194345	3.70533545494586e-24	Exc	LDR2h
**1700016P03Rik**	1.521592293	4.36830679444017e-23	Exc	LDR2h
**Tiparp**	0.632322335	1.10141627407412e-22	Exc	LDR2h
**Hspa8**	0.849081568	1.38213933867634e-22	Exc	LDR2h
**Nrn1**	1.138450699	2.30528428793326e-22	Exc	LDR2h
**Nr4a1**	0.811308208	1.36204598828686e-21	Exc	LDR2h
**4933413L06Rik**	0.845803322	3.06614998639093e-17	Exc	LDR2h
**Cx3cl1**	0.78394994	5.20008584381145e-17	Exc	LDR2h
**mt-Co1**	1.408211334	7.79533062115573e-17	Exc	LDR2h
**Diras2**	0.83729797	9.2905808618069e-17	Exc	LDR2h
**Dalrd3**	0.659563591	2.76888728929501e-16	Exc	LDR2h
**Fos**	0.61886159	2.85048724776239e-16	Exc	LDR2h
**1110008P14Rik**	0.589540653	5.83860087361394e-16	Exc	LDR2h
**Cap1**	0.702262674	8.88724079493451e-15	Exc	LDR2h
**Fosl2**	0.741322971	3.25908274069743e-14	Exc	LDR2h
**Mir670hg**	1.306696366	9.73588570362088e-14	Exc	LDR2h
**mt-Nd1**	1.002455536	2.03785469871936e-13	Exc	LDR2h
**Ppme1**	1.109239951	3.89005304394852e-13	Exc	LDR2h
**Hectd2os**	0.876957687	7.97993370673275e-13	Exc	LDR2h
**Pdp1**	0.625526478	1.59837193122386e-12	Exc	LDR2h
**mt-Co2**	1.290475386	1.94236622137832e-12	Exc	LDR2h
**Tpt1**	0.699908864	1.68283502553256e-11	Exc	LDR2h
**Pabpc4**	0.711324084	5.47302487245049e-11	Exc	LDR2h
**Purb**	0.636201287	8.50690105485389e-11	Exc	LDR2h
**Ptgds**	0.750784172	3.13026918435674e-10	Exc	LDR2h
**Aldoa**	0.610004804	2.44610948105935e-09	Exc	LDR2h
**Cdh9**	0.757539606	3.52732847153241e-09	Exc	LDR2h
**Slc25a3**	0.860360116	4.42165752786393e-09	Exc	LDR2h
**Calm1**	0.593094066	1.10348805667428e-08	Exc	LDR2h
**Baz1a**	1.007465395	1.46975204529908e-08	Exc	LDR2h
**Nrsn1**	0.711868301	1.49623159437254e-08	Exc	LDR2h
**Ppp1r1a**	0.609662959	1.52784581118046e-08	Exc	LDR2h

**Tsnax**	0.726755981	2.72232477674199e-08	Exc	LDR2h
**Eif1**	0.738692438	4.03323183252554e-08	Exc	LDR2h
**Gm14636**	0.633565287	6.06788074215608e-08	Exc	LDR2h
**Eef1a1**	0.588321913	1.39956879988635e-07	Exc	LDR2h
**Rpl6**	0.75474464	2.35465736789329e-07	Exc	LDR2h
**mt-Atp6**	1.163312875	6.22708342885682e-07	Exc	LDR2h
**Rpl41**	0.633974408	6.7130902383177e-07	Exc	LDR2h
**Cox7c**	0.767747072	2.03304267480613e-06	Exc	LDR2h
**Gm15398**	1.011789237	2.13427571756551e-06	Exc	LDR2h
**Sema3c**	1.594872667	2.1344050850549e-06	Exc	LDR2h
**Rpl13**	0.592005619	4.14883212690652e-06	Exc	LDR2h
**Cox8a**	0.725760838	4.23584437464851e-06	Exc	LDR2h
**H3f3b**	0.606208738	4.43089885258534e-06	Exc	LDR2h
**Gm35188**	0.949330264	5.50893614096702e-06	Exc	LDR2h
**Nefm**	0.606027871	5.60923537771688e-06	Exc	LDR2h
**Homer1**	0.682307277	9.58368592807444e-06	Exc	LDR2h
**Atp6v0b**	0.873881848	1.14021117821984e-05	Exc	LDR2h
**Cox4i1**	0.664303365	1.83431495734996e-05	Exc	LDR2h
**Pcsk1**	0.621053146	9.99122138627817e-05	Exc	LDR2h
**1700054A03Rik**	0.740309878	0.000167943	Exc	LDR2h
**Apoe**	1.06422515	0.000228275	Exc	LDR2h
**Oprm1**	0.825814089	0.000379533	Exc	LDR2h
**1700085D07Rik**	0.737742692	0.000497969	Exc	LDR2h
**4930415C11Rik**	1.572985754	0.000598507	Exc	LDR2h
**B230216N24Rik**	0.649467818	0.000864575	Exc	LDR2h
**Tmem243**	0.596456118	0.001039854	Exc	LDR2h
**Sema3e**	0.792901626	0.001861707	Exc	LDR2h
**Gm16351**	0.660114116	0.002696723	Exc	LDR2h
**Ctnna3**	0.875529629	0.003196975	Exc	LDR2h
**Gm15520**	0.676879175	0.00321209	Exc	LDR2h
**Adam2**	0.755066565	0.003300119	Exc	LDR2h
**Egr1**	0.596064638	0.004114369	Exc	LDR2h
**Fth1**	1.322647962	0.004904437	Exc	LDR2h
**Itgav**	0.600939497	0.006002914	Exc	LDR2h
**Arc**	1.120006603	0.006578053	Exc	LDR2h
**mt-Nd4**	0.737324129	0.007138499	Exc	LDR2h
**Gm17231**	0.629888744	0.007428827	Exc	LDR2h
**mt-Cytb**	1.107546399	0.008662806	Exc	LDR2h
**Rapgef5**	0.873699683	0.00910053	Exc	LDR2h

Development • Supplementary information

**Nrd1**	0.641521834	0.013939104	Exc	LDR2h
**Gm11867**	0.757605626	0.01509276	Exc	LDR2h
**Nr4a3**	0.653137791	0.019163717	Exc	LDR2h
**Tle4**	0.599452522	0.02985926	Exc	LDR2h
**Igsf9b**	0.631249244	0.032626789	Exc	LDR2h
**Gm46367**	0.836740031	0.033217542	Exc	LDR2h
**Gm13883**	0.704031967	0.034649779	Exc	LDR2h
**Dot1l**	0.697151458	0.042820522	Exc	LDR2h
**Egr3**	2.193977452	1.68675185802779e-211	Exc	LDR30m
**Fosb**	1.143389529	9.96532099401922e-164	Exc	LDR30m
**Nr4a2**	2.171398898	4.22625042432372e-132	Exc	LDR30m
**Stk40**	1.305982805	1.84868571986701e-127	Exc	LDR30m
**Ube2ql1**	1.357390826	1.52560091503497e-125	Exc	LDR30m
**Homer1**	2.644219618	9.37721856368293e-114	Exc	LDR30m
**Arl5b**	1.807446919	1.73050685173819e-107	Exc	LDR30m
**Frmd6**	1.658810664	2.25486916262945e-106	Exc	LDR30m
**Arid3b**	0.994847633	1.73557028689161e-101	Exc	LDR30m
**Grasp**	1.036500959	1.58998953103902e-96	Exc	LDR30m
**Kdm6b**	0.960029511	2.77004323753758e-90	Exc	LDR30m
**Gm47423**	2.148149338	1.7251485975619e-86	Exc	LDR30m
**Irs2**	1.395704495	9.22816495191919e-83	Exc	LDR30m
**Nptx2**	0.697487481	3.18652058725661e-79	Exc	LDR30m
**Slc25a25**	1.419218622	3.8420102481428e-79	Exc	LDR30m
**Sik2**	1.932567147	4.98214366271315e-78	Exc	LDR30m
**Nr4a1**	1.398926438	5.02366051108938e-78	Exc	LDR30m
**Cpeb3**	1.119885675	6.07891944459182e-76	Exc	LDR30m
**Rheb**	1.574520867	3.67856711422361e-75	Exc	LDR30m
**Dusp14**	0.898875104	1.16615477077194e-72	Exc	LDR30m
**Kdm7a**	1.451994022	1.12602422664421e-69	Exc	LDR30m
**Zdbf2**	1.776755807	1.74630664647468e-68	Exc	LDR30m
**Per1**	1.449536305	7.11866467056484e-65	Exc	LDR30m
**Prag1**	0.899244368	3.90267811458879e-64	Exc	LDR30m
**Ina**	1.234724966	6.1925604617374e-63	Exc	LDR30m
**Trib1**	0.726079654	2.53626630287292e-62	Exc	LDR30m
**Hspa4**	1.241345335	2.78866120067718e-62	Exc	LDR30m
**Arhgef3**	1.623510742	4.19371080737142e-61	Exc	LDR30m
**Rcc2**	0.902568536	1.6500870742253e-59	Exc	LDR30m
**Hsph1**	0.853110669	9.92976891202758e-58	Exc	LDR30m
**Arpc2**	1.012806089	2.35780205560819e-57	Exc	LDR30m

Development • Supplementary information

**Btaf1**	1.086329185	4.45376469040645e-57	Exc	LDR30m
**Hmgcr**	1.026819647	6.63063792919288e-57	Exc	LDR30m
**Gm28294**	0.652876269	6.99157703599859e-57	Exc	LDR30m
**Skil**	0.898261739	1.22696085083778e-55	Exc	LDR30m
**Med14**	1.345644441	4.41962961953997e-55	Exc	LDR30m
**Cwc25**	0.760186688	1.24772365226502e-54	Exc	LDR30m
**Plcxd2**	1.195528536	2.89832893050701e-54	Exc	LDR30m
**Gm17231**	1.24217629	3.68681662759235e-53	Exc	LDR30m
**Ciart**	0.668291784	7.21053040291292e-52	Exc	LDR30m
**Hsd17b12**	0.716545125	9.56922949780239e-52	Exc	LDR30m
**Ankrd33b**	1.207301712	2.97506065750589e-51	Exc	LDR30m
**Npas4**	1.005876018	8.55444306303584e-51	Exc	LDR30m
**Zswim6**	1.120661997	2.7751050844509e-49	Exc	LDR30m
**Mapk4**	1.005803474	4.22476950414733e-48	Exc	LDR30m
**Bdnf**	1.324602805	8.48248560903143e-48	Exc	LDR30m
**Midn**	0.719195726	3.56756689303199e-47	Exc	LDR30m
**Ap2b1**	0.824165649	8.32200844449212e-47	Exc	LDR30m
**Lonrf1**	0.821490112	2.62380115354918e-46	Exc	LDR30m
**Mapk6**	0.668543445	2.83984878586155e-46	Exc	LDR30m
**Baiap2**	1.145003137	7.70432200474643e-46	Exc	LDR30m
**Efhd2**	0.951569136	7.92467076006221e-46	Exc	LDR30m
**Pitpna**	0.803280564	2.18436793657401e-45	Exc	LDR30m
**Osbpl8**	0.936104095	3.12793279574546e-45	Exc	LDR30m
**Camk1g**	0.586071094	1.13126747601644e-44	Exc	LDR30m
**Adora1**	0.895956253	1.23890948896728e-44	Exc	LDR30m
**Etv5**	1.435635247	1.7749509210296e-44	Exc	LDR30m
**Slc6a17**	0.851946333	2.81498516874243e-44	Exc	LDR30m
**Nap1l1**	0.866666517	1.28243294792203e-43	Exc	LDR30m
**Ctnnd1**	0.879927889	3.16471506311619e-43	Exc	LDR30m
**Clstn3**	1.153512522	6.9081458467851e-43	Exc	LDR30m
**Ctps**	0.677642921	2.32311982618025e-42	Exc	LDR30m
**Phf21b**	1.150147463	4.09394267591453e-42	Exc	LDR30m
**Cltc**	0.840610628	4.29199520834721e-42	Exc	LDR30m
**Myh9**	0.919508037	8.45653566099942e-42	Exc	LDR30m
**Tulp4**	0.949370114	9.96446162410582e-42	Exc	LDR30m
**Fosl2**	1.510191107	1.27044508115046e-41	Exc	LDR30m
**Vmp1**	0.877075769	3.07423476009752e-41	Exc	LDR30m
**Elovl5**	0.618366988	1.26090969574686e-40	Exc	LDR30m
**R3hdm2**	0.835741884	1.75775995809461e-39	Exc	LDR30m

Development • Supplementary information

**Fbxo33**	0.988224848	7.0010564031021e-39	Exc	LDR30m
**Eprs**	0.737650741	1.01192718149934e-38	Exc	LDR30m
**Synj2**	0.82472771	1.67168886194184e-38	Exc	LDR30m
**Mbp**	1.247157538	5.08241741137169e-38	Exc	LDR30m
**Txndc11**	0.763559107	5.20638519539337e-38	Exc	LDR30m
**Per2**	0.936244772	6.11703747894686e-37	Exc	LDR30m
**Gnai3**	0.763741179	1.64454975032105e-36	Exc	LDR30m
**4931406P16Rik**	0.701722412	5.8053647669299e-36	Exc	LDR30m
**Mest**	1.057277167	7.41664785224232e-36	Exc	LDR30m
**Coq10b**	1.074030185	2.13430099062237e-35	Exc	LDR30m
**1700016P03Rik**	2.959627091	3.36654134996099e-35	Exc	LDR30m
**Nr4a3**	2.670504946	8.87413950185606e-35	Exc	LDR30m
**Tiparp**	2.168880981	1.22829224786146e-34	Exc	LDR30m
**Jdp2**	1.264567302	2.94716386156141e-34	Exc	LDR30m
**Arih2**	0.749165872	4.75283555459028e-34	Exc	LDR30m
**Nrd1**	1.44532344	6.0407037315092e-34	Exc	LDR30m
**Rims4**	1.056489535	8.16041681880178e-34	Exc	LDR30m
**Ttpal**	0.588855977	8.19019682549988e-34	Exc	LDR30m
**Ivns1abp**	0.735413272	1.7731455372687e-33	Exc	LDR30m
**Por**	0.602029919	3.05316813049829e-33	Exc	LDR30m
**Ak4**	0.589512603	3.90329186536954e-33	Exc	LDR30m
**Smad1**	0.716839781	4.5561560307071e-33	Exc	LDR30m
**Fbl**	0.939813587	1.89957133742264e-32	Exc	LDR30m
**Cap1**	0.661914028	3.53334479877558e-32	Exc	LDR30m
**Mir670hg**	0.865352934	1.22141679760694e-31	Exc	LDR30m
**Scg3**	0.835119534	2.24518316647028e-31	Exc	LDR30m
**Mn1**	0.859841883	3.85205984284676e-31	Exc	LDR30m
**Smg7**	0.752565387	8.51388457273705e-31	Exc	LDR30m
**Ptprn**	1.180653908	1.29269405316481e-30	Exc	LDR30m
**1600020E01Rik**	0.707060011	4.02003027648073e-30	Exc	LDR30m
**Tmem178**	0.784120606	4.05230793335576e-30	Exc	LDR30m
**Smap2**	0.63197067	2.70174648108153e-29	Exc	LDR30m
**Rnf217**	1.084354617	2.71614327831158e-29	Exc	LDR30m
**Mxi1**	0.837529921	2.78341629912973e-29	Exc	LDR30m
**Trim9**	0.777922791	4.7647710485735e-29	Exc	LDR30m
**Cystm1**	0.589854981	6.34362764959806e-29	Exc	LDR30m
**Sidt1**	0.729241624	2.14203734995675e-28	Exc	LDR30m
**Abhd2**	0.965518937	4.25779141217965e-28	Exc	LDR30m
**Ago3**	0.850822826	4.38951760490519e-28	Exc	LDR30m

**P4ha1**	0.898911088	7.74977161285229e-28	Exc	LDR30m
**Ifrd1**	0.914291973	7.85719945977213e-28	Exc	LDR30m
**Chgb**	1.022790692	1.88222039020867e-27	Exc	LDR30m
**Atp6v0d1**	0.658047012	2.07795272821954e-27	Exc	LDR30m
**Ubtd2**	1.024687737	5.81310576685371e-27	Exc	LDR30m
**Mia3**	0.684599381	5.93977147510069e-27	Exc	LDR30m
**Sec24a**	0.594356688	8.14240309033359e-27	Exc	LDR30m
**Kif5c**	0.717656114	8.76069020856917e-27	Exc	LDR30m
**Ppp2ca**	0.613310093	9.12577505595909e-27	Exc	LDR30m
**Slc20a2**	0.757655956	1.61754844010166e-26	Exc	LDR30m
**Gng2**	0.740758784	1.2384850797564e-25	Exc	LDR30m
**Tpm3**	0.659144773	1.90437496343657e-25	Exc	LDR30m
**Ece1**	1.123912715	1.98535740775133e-25	Exc	LDR30m
**Numb**	0.601445349	2.64810152984238e-25	Exc	LDR30m
**Rgs7bp**	0.804602687	3.72504354333434e-24	Exc	LDR30m
**Nudt4**	0.909731906	5.32213911320985e-24	Exc	LDR30m
**Pip5k1a**	0.629351058	7.28059813334988e-24	Exc	LDR30m
**Cdc42ep3**	0.713258073	1.87544961017312e-23	Exc	LDR30m
**Cbarp**	0.836940083	4.15399025283031e-23	Exc	LDR30m
**Cds1**	0.637165095	7.07108030783893e-23	Exc	LDR30m
**Ndel1**	0.756647299	8.2974132093988e-23	Exc	LDR30m
**Sik1**	0.862884155	8.40759223242587e-23	Exc	LDR30m
**Zbtb11**	0.716553401	1.5925735123779e-22	Exc	LDR30m
**Sik3**	1.0423474	1.92411056557483e-22	Exc	LDR30m
**Cx3cl1**	0.775375598	5.33279120689197e-22	Exc	LDR30m
**Atp1a1**	0.793680044	5.83821887356511e-22	Exc	LDR30m
**Prickle1**	0.601707978	7.85165204307135e-22	Exc	LDR30m
**Gm13684**	0.640637314	1.12160044687093e-21	Exc	LDR30m
**Pcsk1**	2.442340364	1.32332528994288e-21	Exc	LDR30m
**Zhx2**	0.695592758	2.09743321385513e-21	Exc	LDR30m
**Acsl4**	0.835618109	2.67084532601312e-21	Exc	LDR30m
**Spred1**	1.132884354	3.20512128141777e-21	Exc	LDR30m
**Nectin1**	0.817883733	4.53334144980616e-21	Exc	LDR30m
**Ppme1**	0.730793388	6.08635895220538e-21	Exc	LDR30m
**Zfp948**	0.660007259	7.34483414293643e-21	Exc	LDR30m
**Hnrnpll**	1.015868944	1.24806214632259e-20	Exc	LDR30m
**Mast3**	0.759174412	1.25079653921361e-20	Exc	LDR30m
**Cpeb4**	1.021792108	1.41306744283445e-20	Exc	LDR30m
**Cry2**	0.836514913	1.88316020153517e-20	Exc	LDR30m

Development • Supplementary information

**Cabp1**	0.794467315	2.35024955589174e-20	Exc	LDR30m
**Nmnat2**	0.796061365	2.90963205585288e-20	Exc	LDR30m
**Usp36**	0.710535705	3.4110101895552e-20	Exc	LDR30m
**Tacc1**	0.693540943	5.29935772498844e-20	Exc	LDR30m
**Bicdl1**	0.645496926	9.41711577495774e-20	Exc	LDR30m
**Ntrk2**	1.104330428	1.01516731882972e-19	Exc	LDR30m
**Lemd3**	0.657644567	1.02315279064994e-19	Exc	LDR30m
**Galnt9**	0.905268685	1.06199557053991e-19	Exc	LDR30m
**Mgrn1**	0.663558687	1.15560722980279e-19	Exc	LDR30m
**Tbc1d9**	0.731454896	1.21774603458907e-19	Exc	LDR30m
**Csnk1a1**	0.636784153	2.03450479068589e-19	Exc	LDR30m
**Dok5**	0.732648298	3.03718896969817e-19	Exc	LDR30m
**Pmepa1**	1.008856448	5.58128355114607e-19	Exc	LDR30m
**Myo1e**	0.641744777	1.35691197749328e-18	Exc	LDR30m
**Lonp2**	0.777416837	2.94028799122278e-18	Exc	LDR30m
**Dnajb5**	0.730438944	6.12928337141561e-18	Exc	LDR30m
**Spred2**	0.879553861	8.83803045413688e-18	Exc	LDR30m
**Slc7a1**	0.594819117	1.64958804079208e-17	Exc	LDR30m
**Hspa8**	0.671732643	1.91154346952934e-17	Exc	LDR30m
**Kpna1**	0.68947496	2.35574406566392e-17	Exc	LDR30m
**Arih1**	0.765821056	2.36266552070023e-17	Exc	LDR30m
**Stx1b**	0.646600879	5.1845808930424e-17	Exc	LDR30m
**Dennd5b**	0.715087936	5.27052244227546e-17	Exc	LDR30m
**Tet3**	0.888463291	7.98295370223498e-17	Exc	LDR30m
**Crem**	0.599392177	8.81102839419593e-17	Exc	LDR30m
**Hivep2**	0.602734151	1.325940207404e-16	Exc	LDR30m
**Klf9**	0.629622224	1.3488864724979e-16	Exc	LDR30m
**Gm3294**	0.617945133	2.81219523476715e-16	Exc	LDR30m
**Ppard**	0.612461278	1.03663350819488e-15	Exc	LDR30m
**Gabbr1**	0.658271829	1.36006846044074e-15	Exc	LDR30m
**Ttbk1**	0.607775137	2.17245305571401e-15	Exc	LDR30m
**Sptbn2**	0.748381353	4.86063390220699e-15	Exc	LDR30m
**Rock2**	0.877864258	9.20783540442382e-15	Exc	LDR30m
**Ccnl1**	0.616488851	9.22379260212896e-15	Exc	LDR30m
**Pak1**	0.669337656	9.52209707125225e-15	Exc	LDR30m
**Dnajc1**	0.958339804	1.31085100316848e-14	Exc	LDR30m
**St8sia5**	0.685139803	3.22221673505778e-14	Exc	LDR30m
**Clip2**	0.790453795	8.89736927148251e-14	Exc	LDR30m
**Phyhipl**	0.654714549	9.91093688229759e-14	Exc	LDR30m

Development • Supplementary information

**Kras**	0.698910038	1.71917359251996e-13	Exc	LDR30m
**Rab6b**	0.836962969	2.13380540139364e-13	Exc	LDR30m
**Nefm**	0.618234723	3.85591709942132e-13	Exc	LDR30m
**Smarca5**	0.70835774	6.96309498190944e-13	Exc	LDR30m
**Tbc1d1**	0.798218879	8.73960355980511e-13	Exc	LDR30m
**Plekhg5**	0.598800568	9.82379990733704e-13	Exc	LDR30m
**Nrn1**	0.840147073	1.35886394012779e-12	Exc	LDR30m
**4933424G05Rik**	0.663984143	1.38732280284237e-12	Exc	LDR30m
**Gramd1b**	0.63454146	1.72492929669895e-12	Exc	LDR30m
**Fkbp1a**	0.612640451	2.40428008165062e-12	Exc	LDR30m
**Fscn1**	0.618041905	4.27724560807873e-12	Exc	LDR30m
**Lncpint**	0.777461349	4.77998546921821e-12	Exc	LDR30m
**Foxo3**	0.64134767	1.3922160908599e-11	Exc	LDR30m
**Gfod1**	0.812285044	2.01282203557054e-11	Exc	LDR30m
**Epha10**	0.606006425	4.32589559480657e-11	Exc	LDR30m
**Ago2**	0.599057682	6.15024480383554e-11	Exc	LDR30m
**Psd3**	0.815451387	7.84525417666256e-11	Exc	LDR30m
**Trak1**	0.77326331	8.62536608001022e-11	Exc	LDR30m
**Hdac5**	0.625889538	1.03081506673283e-10	Exc	LDR30m
**1700110K17Rik**	0.633280262	1.0604981281461e-10	Exc	LDR30m
**Slc25a3**	0.85139143	1.13196719766092e-10	Exc	LDR30m
**R3hdm1**	0.591448874	3.79817128284756e-10	Exc	LDR30m
**Ank**	0.689471516	9.31669437327022e-10	Exc	LDR30m
**Lrrk2**	0.688686083	1.45504303921141e-09	Exc	LDR30m
**Rab6a**	0.719929251	1.81628599538622e-09	Exc	LDR30m
**Csnk1d**	0.623518096	2.28088356588999e-09	Exc	LDR30m
**Sept7**	0.620666503	2.64784686080454e-09	Exc	LDR30m
**Maml3**	0.63098766	6.50936448540586e-09	Exc	LDR30m
**Itgav**	0.656556795	8.11583250303945e-09	Exc	LDR30m
**Cmip**	0.792045441	1.12541038987397e-07	Exc	LDR30m
**Gm48747**	0.671497682	1.45386338040307e-07	Exc	LDR30m
**Prmt8**	0.683647684	2.64411880695397e-07	Exc	LDR30m
**Agap3**	0.597065129	3.0086489259129e-07	Exc	LDR30m
**Pde4a**	0.657942903	5.9338949249965e-07	Exc	LDR30m
**Dlgap4**	0.672872933	6.190148410635e-07	Exc	LDR30m
**Rph3a**	0.692243117	1.3610605270005e-06	Exc	LDR30m
**Ddx50**	0.669516348	1.3845004566638e-06	Exc	LDR30m
**Sgsm1**	0.833369517	1.7898617527506e-06	Exc	LDR30m
**Ccn1**	0.689499327	3.94567799579083e-06	Exc	LDR30m

**Anks1**	0.587805965	9.32050360167378e-05	Exc	LDR30m
**Ski**	0.686815677	0.000101479	Exc	LDR30m
**Ndfip2**	1.223837902	0.000104462	Exc	LDR30m
**Brinp1**	0.615232995	0.000208535	Exc	LDR30m
**Pde10a**	0.709589841	0.000543912	Exc	LDR30m
**Ranbp2**	0.594207369	0.007115545	Exc	LDR30m
**Dusp1**	0.590052045	0.042843416	Exc	LDR30m
**Baz1a**	1.573518088	1.18135295319548e-220	Exc	LDR4h
**Phf21b**	1.379679869	9.49255899962911e-100	Exc	LDR4h
**Nptx2**	1.049422353	1.80020094583888e-89	Exc	LDR4h
**Homer1**	1.648419747	2.61440666116959e-89	Exc	LDR4h
**Mir670hg**	1.242187693	1.49562788320752e-87	Exc	LDR4h
**Mapk4**	1.497785054	5.78466254606349e-86	Exc	LDR4h
**Grasp**	0.808356563	4.14431952876101e-78	Exc	LDR4h
**Slc6a17**	0.87652403	1.21843386570581e-69	Exc	LDR4h
**Arhgap31**	1.221889999	8.58473828889088e-63	Exc	LDR4h
**Bdnf**	1.386605194	7.14056872573708e-51	Exc	LDR4h
**Hsd17b12**	0.628288328	4.69130543489106e-49	Exc	LDR4h
**Egr3**	1.106943024	6.93085960539724e-46	Exc	LDR4h
**Anxa11**	0.66289368	1.06254072073021e-41	Exc	LDR4h
**Hsph1**	0.616391244	1.2381954343286e-40	Exc	LDR4h
**Sik2**	1.598007035	1.50943705327272e-40	Exc	LDR4h
**Ppme1**	0.912169569	4.02055345638472e-40	Exc	LDR4h
**Rph3a**	0.95488711	4.93480772147448e-40	Exc	LDR4h
**Per2**	0.669146	6.76881191167251e-40	Exc	LDR4h
**Adgrd1**	0.640600356	1.22467661813156e-38	Exc	LDR4h
**Arid3b**	0.8213343	3.16395170503623e-38	Exc	LDR4h
**Klhl2**	0.768288939	5.89279023944588e-38	Exc	LDR4h
**Spred1**	1.079522465	1.0489723644577e-37	Exc	LDR4h
**Epha10**	0.931664176	5.93781323249222e-37	Exc	LDR4h
**Kdm6b**	0.733942681	7.46699021347673e-37	Exc	LDR4h
**Tmem178**	0.949479019	1.82379697045919e-36	Exc	LDR4h
**Pfkfb3**	0.608766352	3.2532692178913e-35	Exc	LDR4h
**Slc9a5**	0.834246764	7.02022296077056e-35	Exc	LDR4h
**Scg3**	0.757505492	1.33559588040722e-33	Exc	LDR4h
**Fmnl1**	0.826504047	2.52593249701861e-33	Exc	LDR4h
**Dot1l**	0.793434892	8.29264501001498e-33	Exc	LDR4h
**Spred2**	1.044382593	1.82998078212246e-32	Exc	LDR4h
**Osbpl8**	0.631972125	3.95713149522604e-32	Exc	LDR4h

Development • Supplementary information

**Scube1**	0.970598083	4.06649594793058e-32	Exc	LDR4h
**Numb**	0.645371291	6.05568262099811e-31	Exc	LDR4h
**Sorcs3**	1.615610205	6.38831389952747e-29	Exc	LDR4h
**Mamld1**	0.612329563	1.0683295071995e-28	Exc	LDR4h
**Prmt8**	0.909068361	2.85084388737027e-28	Exc	LDR4h
**Vmp1**	0.661912827	5.01886747073018e-28	Exc	LDR4h
**Pak6**	0.726969836	5.84423673623443e-28	Exc	LDR4h
**Ddah1**	0.795000719	7.47293154101325e-28	Exc	LDR4h
**Osbpl3**	0.835730032	1.59263504302183e-27	Exc	LDR4h
**Ifrd1**	0.659764035	2.64852766170765e-27	Exc	LDR4h
**Pcsk1**	1.201714585	4.0290136624672e-27	Exc	LDR4h
**Frmd6**	1.02046523	9.61746807685069e-26	Exc	LDR4h
**Prkg2**	0.939521469	1.09056341420943e-25	Exc	LDR4h
**2510009E07Rik**	0.705612324	1.77481056206836e-25	Exc	LDR4h
**Etv5**	0.948308828	2.21053638696784e-24	Exc	LDR4h
**Itgav**	0.954412215	6.71592737352573e-24	Exc	LDR4h
**Kdm7a**	0.713090823	9.45066712450111e-24	Exc	LDR4h
**Nrd1**	0.907971201	1.26537720472951e-23	Exc	LDR4h
**Hs3st2**	0.755716146	3.48322830538122e-23	Exc	LDR4h
**Rheb**	0.792397464	4.75553989776064e-23	Exc	LDR4h
**Cap2**	0.600360513	5.40943573110099e-23	Exc	LDR4h
**Tet3**	1.00913858	5.60917296794815e-23	Exc	LDR4h
**Gfra1**	0.661870608	1.62344883696602e-22	Exc	LDR4h
**Ntrk2**	1.164206497	2.63533551542806e-22	Exc	LDR4h
**Zbtb16**	0.963215625	1.12303742521529e-21	Exc	LDR4h
**Jdp2**	0.850629273	1.23265873965724e-21	Exc	LDR4h
**Zdbf2**	0.752488465	1.34216466736807e-21	Exc	LDR4h
**1700016P03Rik**	1.966780364	3.4813027449709e-21	Exc	LDR4h
**Prag1**	0.587195467	3.61684613797608e-21	Exc	LDR4h
**Sgsm1**	1.387928707	4.04352334603053e-21	Exc	LDR4h
**Cop1**	0.590203231	4.31359111341559e-21	Exc	LDR4h
**Sik3**	0.929793939	7.1853607867791e-21	Exc	LDR4h
**Gramd1b**	0.810520734	3.16964901932598e-20	Exc	LDR4h
**Stk40**	0.749509488	6.57723092918479e-20	Exc	LDR4h
**Prim2**	0.661789815	9.03935872117077e-20	Exc	LDR4h
**Bcor**	0.634047351	1.11748342002081e-19	Exc	LDR4h
**Tmtc2**	0.725275065	1.15026711164836e-19	Exc	LDR4h
**Gm13684**	0.765486153	2.92934063126939e-19	Exc	LDR4h
**Arhgef3**	0.767724654	5.24944885253881e-19	Exc	LDR4h

Development • Supplementary information

**Tiparp**	1.069926992	9.7189597171675e-19	Exc	LDR4h
**Pcdh15**	0.805565663	6.16490721494503e-18	Exc	LDR4h
**Trim9**	0.694679255	1.22125415364072e-17	Exc	LDR4h
**Nrp1**	0.829726261	2.16963915994644e-17	Exc	LDR4h
**P4ha1**	0.596074689	2.62545347025774e-17	Exc	LDR4h
**Zswim6**	0.80824646	4.54109781342988e-17	Exc	LDR4h
**Rgs20**	1.041600808	2.01606519865745e-16	Exc	LDR4h
**Cap1**	0.592097414	2.48724867836193e-16	Exc	LDR4h
**Akap13**	0.739147493	4.66973292430611e-16	Exc	LDR4h
**Rock2**	0.763211913	5.1508463868873e-16	Exc	LDR4h
**Ankrd33b**	0.738357439	9.45741086523875e-16	Exc	LDR4h
**Ppm1h**	0.848002824	1.27853141935898e-15	Exc	LDR4h
**Rapgef5**	0.831333631	2.03987552499688e-15	Exc	LDR4h
**Hectd2**	0.89652796	3.57734762164168e-15	Exc	LDR4h
**Dgkz**	0.654075331	6.29801112794367e-15	Exc	LDR4h
**Pdzd2**	1.037984856	7.19907305937798e-15	Exc	LDR4h
**Nrxn2**	0.760783195	7.64090373579086e-15	Exc	LDR4h
**Tsnax**	0.855861291	1.04635266688105e-14	Exc	LDR4h
**Dnajc1**	0.818193543	1.06769464730852e-14	Exc	LDR4h
**Zmiz1**	1.000457464	2.43597636730295e-14	Exc	LDR4h
**Eml5**	0.866835315	1.36011271321234e-13	Exc	LDR4h
**Gm46367**	0.773561347	1.9708775597056e-13	Exc	LDR4h
**Lrrk2**	0.741961776	6.48027837127659e-13	Exc	LDR4h
**Clstn3**	0.718257955	9.03925630566295e-13	Exc	LDR4h
**Ptprn**	1.024807018	2.43444871251405e-12	Exc	LDR4h
**Ece1**	0.742487157	1.97023827994321e-11	Exc	LDR4h
**Grb2**	0.665777168	2.74309593552459e-11	Exc	LDR4h
**Nr4a1**	0.793497583	7.84844216435957e-11	Exc	LDR4h
**Mast3**	0.651913639	9.64942123135843e-11	Exc	LDR4h
**Brinp1**	0.807956734	1.28880437444757e-10	Exc	LDR4h
**Fosl2**	0.733371503	2.08483408992112e-10	Exc	LDR4h
**Igsf9b**	0.642922278	2.99739252563859e-10	Exc	LDR4h
**Galnt9**	0.657089348	3.4224631859487e-10	Exc	LDR4h
**Nrn1**	0.747227702	7.7624938655322e-10	Exc	LDR4h
**St8sia5**	0.619415902	5.51377238835453e-09	Exc	LDR4h
**Lhfpl3**	0.662025298	5.71304607648195e-09	Exc	LDR4h
**Mbp**	0.659293409	8.76127085698438e-09	Exc	LDR4h
**Gfod1**	0.758249593	1.09392313711363e-08	Exc	LDR4h
**Rnd3**	0.737008688	3.78869951087961e-08	Exc	LDR4h

Development • Supplementary information

**Megf11**	1.459813937	2.16142664892534e-07	Exc	LDR4h
**Tmem163**	0.665165974	3.36244947088371e-07	Exc	LDR4h
**Fstl4**	0.676943171	3.53190930106107e-07	Exc	LDR4h
**Sema3e**	0.61094708	1.22553518732169e-05	Exc	LDR4h
**Elmo1**	1.427581839	2.4900690917158e-05	Exc	LDR4h
**Mical2**	0.609042264	0.000104315	Exc	LDR4h
**Gm15398**	1.319581444	0.000118862	Exc	LDR4h
**Disp3**	0.890809967	0.000410533	Exc	LDR4h
**Maml3**	1.580289401	0.002051578	Exc	LDR4h
**Nr4a3**	0.840814595	0.002273415	Exc	LDR4h
**Apoe**	0.917341214	1.80147572049933e-84	Exc	LDR6h
**Ptgds**	0.633279515	8.5405206838696e-60	Exc	LDR6h
**Fth1**	1.35488454	1.98501618111912e-51	Exc	LDR6h
**Cst3**	0.792769996	8.66817019931016e-47	Exc	LDR6h
**Pcsk1n**	0.643890099	2.88852484703408e-32	Exc	LDR6h
**mt-Co3**	1.34774865	1.39955443077169e-28	Exc	LDR6h
**Cox4i1**	0.741642525	1.67994480663864e-24	Exc	LDR6h
**B230217C12Rik**	0.589717147	6.80744722492176e-23	Exc	LDR6h
**mt-Co1**	1.466662201	1.4002182391533e-21	Exc	LDR6h
**Atxn7l3b**	0.649654506	1.88306958560297e-20	Exc	LDR6h
**Rap2b**	0.6789573	1.13403986131176e-19	Exc	LDR6h
**H3f3b**	0.967034298	4.18627335567255e-19	Exc	LDR6h
**Gm12027**	0.63711946	3.13036446926851e-17	Exc	LDR6h
**Eif1**	0.801913142	9.44946960642886e-17	Exc	LDR6h
**Slc25a4**	0.673942571	1.76693444005719e-16	Exc	LDR6h
**Gm27032**	0.98167936	1.91802216219994e-16	Exc	LDR6h
**Srsf7**	1.158029563	1.95724268417754e-16	Exc	LDR6h
**Ppia**	0.637806971	2.97135632069761e-16	Exc	LDR6h
**Cox6c**	0.654479918	3.06144923700179e-16	Exc	LDR6h
**Cox8a**	0.781992202	2.85741340246216e-14	Exc	LDR6h
**Gm32250**	0.755327579	2.00837335566648e-13	Exc	LDR6h
**Tpt1**	0.630881086	2.86530536303762e-13	Exc	LDR6h
**Rpl9**	0.593915621	1.10144809909994e-12	Exc	LDR6h
**Rps8**	0.631172356	5.62233896518662e-12	Exc	LDR6h
**Calm1**	0.614505704	9.83646959409561e-12	Exc	LDR6h
**Nrgn**	0.68581534	1.03508432501673e-11	Exc	LDR6h
**Rpl6**	0.830408698	1.19661300207451e-11	Exc	LDR6h
**mt-Co2**	1.152415985	6.86782363435168e-11	Exc	LDR6h
**Ckb**	0.641005352	9.02750615384617e-11	Exc	LDR6h

Development • Supplementary information

**Rpl13**	0.635693464	9.35859580743934e-11	Exc	LDR6h
**Tmsb4x**	0.785968945	9.67243356165951e-11	Exc	LDR6h
**Atpif1**	0.646847089	1.90262899573983e-10	Exc	LDR6h
**Dnaja2**	0.692536673	2.49936350691012e-10	Exc	LDR6h
**Mdh1**	0.627975497	3.56507440027517e-10	Exc	LDR6h
**Arpp19**	0.61217632	5.03568714755297e-10	Exc	LDR6h
**Ly6h**	0.588531759	1.29528338045788e-09	Exc	LDR6h
**Cox7b**	0.644089776	1.75235855306223e-09	Exc	LDR6h
**Arf5**	0.590018799	2.3812793417216e-09	Exc	LDR6h
**4930473D10Rik**	0.675960739	3.97264421801315e-09	Exc	LDR6h
**Sema3c**	0.893308885	1.0117331871095e-08	Exc	LDR6h
**Dact2**	0.615338912	1.18280003638248e-08	Exc	LDR6h
**Rpl41**	0.652637134	1.88228564603838e-08	Exc	LDR6h
**Slc50a1**	0.627610014	2.54319346745104e-08	Exc	LDR6h
**Trim35**	0.754527576	6.60055396943117e-08	Exc	LDR6h
**6430590A07Rik**	0.639368784	1.4230012784299e-07	Exc	LDR6h
**Nap1l5**	0.677873037	3.12175601312914e-07	Exc	LDR6h
**Tmem243**	0.639115797	3.15029284112826e-07	Exc	LDR6h
**Gm28376**	0.64938997	7.64096813670248e-06	Exc	LDR6h
**Gm15520**	0.648102446	1.40556714759186e-05	Exc	LDR6h
**Eef1a1**	0.676730828	3.03088267304917e-05	Exc	LDR6h
**Atp6v0b**	0.924912331	3.34938373211738e-05	Exc	LDR6h
**Mrps26**	0.590486234	4.88472089256247e-05	Exc	LDR6h
**Rad51ap2**	0.725550976	5.71413788069853e-05	Exc	LDR6h
**Hmgn3**	0.59549291	7.0995471826724e-05	Exc	LDR6h
**Prpf4b**	0.707377035	7.15967527939113e-05	Exc	LDR6h
**Crebzf**	0.646622382	7.70325209294541e-05	Exc	LDR6h
**Rmdn1**	0.598820493	8.6032867818882e-05	Exc	LDR6h
**A230004M16Rik**	0.585875144	9.38739420003505e-05	Exc	LDR6h
**Gm46367**	0.746000298	0.000141102	Exc	LDR6h
**Pura**	0.59158644	0.000188787	Exc	LDR6h
**BC005561**	0.591995388	0.000234068	Exc	LDR6h
**Gm35188**	0.803323132	0.000389321	Exc	LDR6h
**Ubb**	0.894792955	0.000511809	Exc	LDR6h
**P2ry14**	0.721481214	0.000822482	Exc	LDR6h
**Prelid3a**	0.610052704	0.000829822	Exc	LDR6h
**5330438D12Rik**	0.729354421	0.001241374	Exc	LDR6h
**Oprm1**	0.67555301	0.002824262	Exc	LDR6h
**Malat1**	1.30494771	0.012327923	Exc	LDR6h

Development • Supplementary information

**Cox7c**	0.9415041	0.013815742	Exc	LDR6h
**mt-Nd1**	0.930478156	0.017756695	Exc	LDR6h
**Gm43376**	0.601940094	0.038959567	Exc	LDR6h
**mt-Co3**	1.173555542	1.35552078994995e-11	Inh	LDR2h
**mt-Co1**	1.225194048	4.46268034769106e-11	Inh	LDR2h
**1700054A03Rik**	0.772437461	2.94899670103575e-09	Inh	LDR2h
**Ptprn**	0.924587433	1.99246089183089e-08	Inh	LDR2h
**Nrgn**	0.88850753	6.34578510020599e-08	Inh	LDR2h
**Brd9**	0.769822084	2.25051649884467e-07	Inh	LDR2h
**Egr1**	0.731268019	1.77035603486814e-06	Inh	LDR2h
**Dalrd3**	0.673895349	2.73091604086528e-06	Inh	LDR2h
**Atp6v0b**	0.819938898	4.53588867454782e-06	Inh	LDR2h
**Calm1**	0.66177765	1.31024462077992e-05	Inh	LDR2h
**BC018473**	0.591164773	1.90799076150823e-05	Inh	LDR2h
**Cox7c**	0.805213378	2.00375020317918e-05	Inh	LDR2h
**Slc25a3**	0.79697244	4.090548460729e-05	Inh	LDR2h
**Pabpc4**	0.725618346	0.000172585	Inh	LDR2h
**Hectd2os**	0.67356883	0.000383772	Inh	LDR2h
**Nr4a1**	0.593202626	0.000490739	Inh	LDR2h
**Mt1**	0.612253116	0.000721307	Inh	LDR2h
**Rpl6**	0.741201642	0.001090928	Inh	LDR2h
**Cox8a**	0.735768669	0.001682137	Inh	LDR2h
**Cck**	0.643388752	0.001799409	Inh	LDR2h
**Atp1a1**	0.612465832	0.003494996	Inh	LDR2h
**Rpl13**	0.692483822	0.004066515	Inh	LDR2h
**Hspa8**	0.623724038	0.006460299	Inh	LDR2h
**Gm13944**	0.659571856	0.01595795	Inh	LDR2h
**Rplp1**	0.602665324	0.018341875	Inh	LDR2h
**1700016P03Rik**	1.098763374	0.021108538	Inh	LDR2h
**Apoe**	1.074982371	0.0235365	Inh	LDR2h
**Xlr5a**	0.625992547	0.026743591	Inh	LDR2h
**Eif2s2**	0.601204472	0.027340932	Inh	LDR2h
**Nap1l5**	0.691848358	0.031647406	Inh	LDR2h
**Gm11867**	0.83484806	0.036168278	Inh	LDR2h
**Nr4a3**	2.232947373	1.46992118903268e-58	Inh	LDR30m
**Homer1**	1.999498041	1.46904603320017e-50	Inh	LDR30m
**Pcsk1**	1.428134114	6.99969237821425e-37	Inh	LDR30m
**1700016P03Rik**	2.408582149	1.30311080828445e-25	Inh	LDR30m
**Nr4a1**	1.331243247	1.08865325295597e-24	Inh	LDR30m

**Nr4a2**	1.088253639	3.61017284432029e-24	Inh	LDR30m
**Per1**	1.036422214	1.5594077642509e-18	Inh	LDR30m
**Gm47423**	1.118220091	1.91321835634383e-15	Inh	LDR30m
**Coq10b**	0.901445958	4.95894099544487e-15	Inh	LDR30m
**Mir670hg**	0.859692315	3.10015616366052e-13	Inh	LDR30m
**Gad1**	0.812637484	1.05960758501295e-12	Inh	LDR30m
**Tiparp**	0.865559951	2.45664304904282e-12	Inh	LDR30m
**Frmd6**	0.952939619	3.53295252021656e-12	Inh	LDR30m
**Slc23a2**	0.717859089	6.06857308689453e-10	Inh	LDR30m
**Fosl2**	1.259579018	1.38039426582017e-09	Inh	LDR30m
**Eepd1**	0.825281256	7.73279278030191e-09	Inh	LDR30m
**Dlx6os1**	0.742933628	1.83824125007987e-08	Inh	LDR30m
**Chgb**	0.768581361	3.61941023542881e-08	Inh	LDR30m
**Sik1**	0.668739757	4.47221908434686e-08	Inh	LDR30m
**Tent5a**	0.690841822	5.5801266731013e-08	Inh	LDR30m
**Egr3**	0.611631091	1.05527910276445e-07	Inh	LDR30m
**Arl5b**	0.719769864	2.18173008682352e-07	Inh	LDR30m
**Cort**	0.58678663	3.19639166187695e-07	Inh	LDR30m
**Stat3**	0.694300872	5.19572982673344e-07	Inh	LDR30m
**Cpeb3**	0.633312632	5.27210589627072e-07	Inh	LDR30m
**Ifrd1**	0.668264977	4.58730562097904e-06	Inh	LDR30m
**Sik2**	0.700974502	5.91010376088741e-06	Inh	LDR30m
**Fosb**	0.845905457	8.08396953278466e-06	Inh	LDR30m
**Ptprn**	0.748509131	3.03985999263452e-05	Inh	LDR30m
**Nectin2**	0.595918686	5.39876238423246e-05	Inh	LDR30m
**Sipa1l2**	0.674871769	0.000119346	Inh	LDR30m
**Ube2ql1**	0.607642211	0.000194879	Inh	LDR30m
**Rock2**	0.750646041	0.000196278	Inh	LDR30m
**Slc6a17**	0.608355342	0.000282337	Inh	LDR30m
**Slc7a8**	0.690654528	0.000369976	Inh	LDR30m
**Hspa4**	0.628168991	0.000515116	Inh	LDR30m
**Elovl5**	0.623648462	0.000515548	Inh	LDR30m
**Rgs7bp**	0.589889122	0.003211564	Inh	LDR30m
**Rims4**	0.605526782	0.005232898	Inh	LDR30m
**Npas4**	0.685216868	0.010866295	Inh	LDR30m
**Hunk**	0.58506867	0.011901195	Inh	LDR30m
**Etv5**	0.627931257	0.012712088	Inh	LDR30m
**Gm47283**	0.690325253	0.014153777	Inh	LDR30m
**Mir670hg**	1.435846544	3.60514550537787e-37	Inh	LDR4h

**1700016P03Rik**	1.389231149	4.10483574307687e-34	Inh	LDR4h
**Homer1**	1.27435421	6.19294825356707e-32	Inh	LDR4h
**Myo1e**	0.853381014	4.89636967215236e-11	Inh	LDR4h
**Alkal2**	0.687235537	6.66775296252166e-11	Inh	LDR4h
**Pex5**	0.690305433	3.98538728258946e-08	Inh	LDR4h
**Nek7**	0.649103358	7.63409442640072e-08	Inh	LDR4h
**Hunk**	0.677177634	4.14990160657555e-07	Inh	LDR4h
**St18**	0.707539077	5.91142793562737e-07	Inh	LDR4h
**Sik2**	0.694676338	1.48986366092969e-06	Inh	LDR4h
**Frmd6**	0.595543296	1.58588516855902e-05	Inh	LDR4h
**Rock2**	0.728495514	1.85846483682203e-05	Inh	LDR4h
**Slc6a17**	0.618566248	2.036858087086e-05	Inh	LDR4h
**Lgi2**	0.592404646	4.29794393089248e-05	Inh	LDR4h
**Nr4a1**	0.589770558	0.000114011	Inh	LDR4h
**Sik3**	0.620379036	0.001996411	Inh	LDR4h
**Cntnap5c**	0.627676893	0.00349045	Inh	LDR4h
**Fth1**	1.210872282	4.13061719232815e-28	Inh	LDR6h
**Apoe**	0.992137438	4.4379879486885e-18	Inh	LDR6h
**mt-Co3**	1.211157961	4.32582045393489e-15	Inh	LDR6h
**Atp5a1**	0.8850308	2.10853032661109e-12	Inh	LDR6h
**Srsf7**	1.07935436	4.33401916348342e-12	Inh	LDR6h
**H3f3b**	0.887501773	1.09178787051544e-10	Inh	LDR6h
**Cox7c**	1.001137586	2.90047511919086e-10	Inh	LDR6h
**Trim35**	0.832057497	2.21385197838241e-09	Inh	LDR6h
**Tmsb4x**	0.902124292	6.16230579257884e-09	Inh	LDR6h
**Nap1l5**	0.926160856	7.05467675339704e-09	Inh	LDR6h
**Gm35188**	1.043323425	8.55207056565931e-09	Inh	LDR6h
**Cox4i1**	0.74698815	1.28077195768394e-08	Inh	LDR6h
**Ptgds**	0.652769025	2.50505916364959e-08	Inh	LDR6h
**Ubb**	0.888984002	2.69999140337163e-08	Inh	LDR6h
**Calm1**	0.733119549	3.44753664327366e-08	Inh	LDR6h
**Cox7b**	0.827557968	4.27089028227e-08	Inh	LDR6h
**Rpl6**	0.828276145	4.31486974403708e-08	Inh	LDR6h
**Hmgn3**	0.799107428	4.80852163570795e-08	Inh	LDR6h
**Eif1**	0.776710869	6.35814166505579e-08	Inh	LDR6h
**Cox8a**	0.775851173	7.22846037897478e-08	Inh	LDR6h
**Gm27032**	0.923064448	9.1377192347575e-08	Inh	LDR6h
**Cst3**	0.691558742	3.54589227677929e-07	Inh	LDR6h
**Nrgn**	0.704922607	3.83228586381224e-07	Inh	LDR6h

Development • Supplementary information

**Dnaja2**	0.825000806	1.95217414941817e-06	Inh	LDR6h
**Gm32250**	0.78966051	2.53195358401317e-06	Inh	LDR6h
**Slc25a4**	0.751665669	2.81764618753341e-06	Inh	LDR6h
**Rpl9**	0.644388459	6.17653883369293e-06	Inh	LDR6h
**Pdcd7**	0.704579423	7.06193396918812e-06	Inh	LDR6h
**Pcsk1n**	0.659310179	1.03073721567355e-05	Inh	LDR6h
**Rps21**	0.680117435	1.62334908639387e-05	Inh	LDR6h
**Atp6v0b**	0.80037232	2.2660635756029e-05	Inh	LDR6h
**Ckb**	0.711605923	3.00281087075409e-05	Inh	LDR6h
**Nrg3os**	0.794562831	4.74642555841162e-05	Inh	LDR6h
**Ndufaf7**	0.683187704	4.7788131139951e-05	Inh	LDR6h
**Atxn7l3b**	0.602614609	0.000111702	Inh	LDR6h
**Gm15520**	0.651170699	0.000131633	Inh	LDR6h
**Prpf4b**	0.744393615	0.00013942	Inh	LDR6h
**Rps8**	0.640663125	0.000142807	Inh	LDR6h
**Shisa4**	0.668519214	0.00016684	Inh	LDR6h
**mt-Co1**	1.341496369	0.000203797	Inh	LDR6h
**Mrpl38**	0.618220488	0.000238785	Inh	LDR6h
**BC031181**	0.600682837	0.000252358	Inh	LDR6h
**Bmyc**	0.618031707	0.000289098	Inh	LDR6h
**Gm43376**	0.687336707	0.000329122	Inh	LDR6h
**Gm15614**	0.689272077	0.000329709	Inh	LDR6h
**5330438D12Rik**	0.805287327	0.000362725	Inh	LDR6h
**Ppia**	0.593863559	0.000362936	Inh	LDR6h
**Rpl13**	0.668082218	0.0003745	Inh	LDR6h
**Mdh1**	0.705134277	0.000377143	Inh	LDR6h
**Rmdn1**	0.681159588	0.00045189	Inh	LDR6h
**Rpl37a**	0.615864214	0.000622557	Inh	LDR6h
**Eid1**	0.644486876	0.000634903	Inh	LDR6h
**Gm28501**	0.682232839	0.00067538	Inh	LDR6h
**Slc25a3**	0.739007157	0.000874839	Inh	LDR6h
**Pla2g3**	0.729447373	0.001300022	Inh	LDR6h
**3110021N24Rik**	0.670094207	0.001318947	Inh	LDR6h
**Rhbdl1**	0.606446042	0.001690684	Inh	LDR6h
**P2ry14**	0.661603465	0.002005767	Inh	LDR6h
**Scn9a**	0.712855972	0.002008271	Inh	LDR6h
**Ndufa4**	0.613871513	0.002226814	Inh	LDR6h
**Sorbs2os**	0.653747209	0.002315077	Inh	LDR6h
**Malat1**	1.336188462	0.00233331	Inh	LDR6h

**Gm20642**	0.829025515	0.002588022	Inh	LDR6h
**Snhg11**	0.676993514	0.00328913	Inh	LDR6h
**Crebzf**	0.635991079	0.003370424	Inh	LDR6h
**Cox6c**	0.633019249	0.00382906	Inh	LDR6h
**Psmc6**	0.603320357	0.004205048	Inh	LDR6h
**Gm36975**	0.671092975	0.004849984	Inh	LDR6h
**Gm46367**	0.619859269	0.005105145	Inh	LDR6h
**Gsdme**	0.640405156	0.011405606	Inh	LDR6h
**4933413L06Rik**	0.609342307	0.013059217	Inh	LDR6h
**Eif2s2**	0.639751525	0.015465615	Inh	LDR6h
**Gm48678**	0.652024678	0.016258562	Inh	LDR6h
**Far1os**	0.610554921	0.017150946	Inh	LDR6h
**Lynx1**	0.600397783	0.01858824	Inh	LDR6h
**Slc38a2**	0.626159911	0.019368719	Inh	LDR6h
**Pura**	0.639636795	0.020300541	Inh	LDR6h
**Gm48321**	0.67530754	0.028961853	Inh	LDR6h
**Pnn**	0.643928575	0.034141199	Inh	LDR6h
**Tubb3**	0.677993122	0.035277078	Inh	LDR6h
**Prelid3a**	0.6597253	0.035865902	Inh	LDR6h
**Rnpc3**	0.628823795	0.039019602	Inh	LDR6h
**Ptprn**	1.106253072	1.03707330590353e-07	Grin3a	LDR2h
**Fth1**	1.288933619	3.36508561223717e-06	Grin3a	LDR2h
**mt-Co3**	0.992231855	1.20935907496319e-05	Grin3a	LDR2h
**Sema3c**	1.183015499	1.78266866367323e-05	Grin3a	LDR2h
**mt-Co1**	1.136228215	5.48334594675891e-05	Grin3a	LDR2h
**Egr1**	0.967809858	0.002346392	Grin3a	LDR2h
**4930415C11Rik**	0.981281909	0.002461312	Grin3a	LDR2h
**mt-Co2**	0.935516732	0.00295903	Grin3a	LDR2h
**Nrgn**	0.968807088	0.004000253	Grin3a	LDR2h
**Ptgds**	0.937931372	0.006528371	Grin3a	LDR2h
**Gm13944**	0.971392556	0.009058995	Grin3a	LDR2h
**mt-Nd1**	0.919446029	0.011129714	Grin3a	LDR2h
**Gabra2**	0.850244515	0.013585015	Grin3a	LDR2h
**Apoe**	0.961103594	0.037316964	Grin3a	LDR2h
**Atp6v0b**	0.844338979	0.047608634	Grin3a	LDR2h
**Homer1**	2.376797542	6.46779263456491e-52	Grin3a	LDR30m
**Gad1**	1.774461639	6.31365711014742e-25	Grin3a	LDR30m
**Pcsk1**	1.950592395	4.61080533153581e-19	Grin3a	LDR30m
**Cpeb3**	1.070558899	1.58507229989645e-14	Grin3a	LDR30m

Development • Supplementary information

**Nr4a3**	2.092506736	6.98590458686727e-13	Grin3a	LDR30m
**Frmd6**	1.356380677	7.01540686219116e-12	Grin3a	LDR30m
**Gm47423**	1.496519349	4.77438577239226e-10	Grin3a	LDR30m
**Gpt2**	1.302769066	5.11264350163047e-10	Grin3a	LDR30m
**Nr4a1**	1.544235591	8.16739497063084e-09	Grin3a	LDR30m
**Per1**	1.343484964	1.89127969586791e-08	Grin3a	LDR30m
**Rcan2**	0.994158938	3.46388749812867e-08	Grin3a	LDR30m
**Afap1**	1.02528816	2.48852813549644e-07	Grin3a	LDR30m
**Fosl2**	1.519634642	3.68009144688327e-07	Grin3a	LDR30m
**Sik2**	0.926329014	1.20981423132642e-06	Grin3a	LDR30m
**Trim9**	0.847156933	1.50366036367903e-05	Grin3a	LDR30m
**Ell2**	0.958009885	1.8624498274691e-05	Grin3a	LDR30m
**Slc7a8**	1.080202385	2.76210425579089e-05	Grin3a	LDR30m
**Coq10b**	1.129128794	4.20329161149763e-05	Grin3a	LDR30m
**Snap25**	0.721861365	4.79918322105491e-05	Grin3a	LDR30m
**Ankrd33b**	1.293380187	5.30788566365074e-05	Grin3a	LDR30m
**Tiparp**	1.143235177	0.000102617	Grin3a	LDR30m
**Slc23a2**	0.823931637	0.000243849	Grin3a	LDR30m
**Nrd1**	0.979439305	0.000550571	Grin3a	LDR30m
**Mtcl1**	0.802199905	0.00119181	Grin3a	LDR30m
**1700016P03Rik**	2.740299687	0.001325244	Grin3a	LDR30m
**Eepd1**	1.002022714	0.001785775	Grin3a	LDR30m
**Lrrc8b**	0.90186785	0.002122378	Grin3a	LDR30m
**Rims4**	0.92625227	0.005062159	Grin3a	LDR30m
**Ppargc1b**	0.952476828	0.005291925	Grin3a	LDR30m
**Zswim6**	0.860152332	0.005770503	Grin3a	LDR30m
**Syn2**	0.665382041	0.006205867	Grin3a	LDR30m
**Vegfc**	0.899089999	0.006479275	Grin3a	LDR30m
**Egr1**	0.949425749	0.009561474	Grin3a	LDR30m
**Ppm1l**	0.867503405	0.009802978	Grin3a	LDR30m
**Cort**	0.89102517	0.010973176	Grin3a	LDR30m
**Chgb**	0.862602977	0.011084981	Grin3a	LDR30m
**Rock2**	0.811765113	0.015737258	Grin3a	LDR30m
**Ptprn**	0.923658607	0.019287797	Grin3a	LDR30m
**Nectin2**	0.939794472	0.020614571	Grin3a	LDR30m
**Pak1**	0.722091988	0.026104122	Grin3a	LDR30m
**AY036118**	0.831614858	0.029316172	Grin3a	LDR30m
**Gm47283**	0.743679633	0.035879146	Grin3a	LDR30m
**Acsl4**	0.811216916	0.043434907	Grin3a	LDR30m

**Slc6a17**	0.767719431	0.047897477	Grin3a	LDR30m
**Homer1**	1.592326153	1.48588964439941e-15	Grin3a	LDR4h
**Mir670hg**	1.541755468	5.12051024686234e-10	Grin3a	LDR4h
**Sik2**	1.040695473	2.19551601236543e-07	Grin3a	LDR4h
**Gabra2**	1.080668755	2.49039314941958e-07	Grin3a	LDR4h
**Gad1**	1.152875076	6.65077559451605e-07	Grin3a	LDR4h
**Sdk1**	0.93370879	3.9665379929743e-05	Grin3a	LDR4h
**Frmd6**	0.9469057	7.81604554583401e-05	Grin3a	LDR4h
**Kcnma1**	0.790949977	0.001409812	Grin3a	LDR4h
**Alk**	1.000624725	0.001570955	Grin3a	LDR4h
**Pcsk1**	0.909131167	0.003788471	Grin3a	LDR4h
**Ptprn**	0.832825245	0.010087374	Grin3a	LDR4h
**9630028H03Rik**	0.918233598	0.012578392	Grin3a	LDR4h
**Gfod1**	0.880807776	0.018256436	Grin3a	LDR4h
**Npy**	0.883567817	0.021661949	Grin3a	LDR4h
**Lrrc8b**	0.763753073	0.022438826	Grin3a	LDR4h
**Pacsin2**	0.842800161	0.024161458	Grin3a	LDR4h
**Tenm4**	0.650955292	0.029602799	Grin3a	LDR4h
**Rock2**	0.803219226	0.0427837	Grin3a	LDR4h
**mt-Co1**	1.313048645	1.17285788767638e-11	Grin3a	LDR6h
**mt-Co3**	1.205233474	8.43137244127337e-11	Grin3a	LDR6h
**Fth1**	1.194507642	7.71022790264555e-07	Grin3a	LDR6h
**Nrg3os**	0.800037467	2.75863032613107e-05	Grin3a	LDR6h
**Srsf7**	0.963606727	7.80339242272387e-05	Grin3a	LDR6h
**mt-Co2**	0.97488072	0.000812839	Grin3a	LDR6h
**mt-Cytb**	0.977249331	0.000833345	Grin3a	LDR6h
**Prpf4b**	0.795670254	0.001368601	Grin3a	LDR6h
**Malat1**	1.423198144	0.00368174	Grin3a	LDR6h
**Snhg11**	0.656686262	0.004786635	Grin3a	LDR6h
**Trim35**	0.873902233	0.005636775	Grin3a	LDR6h
**Gm48321**	0.923916623	0.008467549	Grin3a	LDR6h
**Tmsb4x**	0.944100681	0.014430003	Grin3a	LDR6h
**P2ry14**	0.851441392	0.014893861	Grin3a	LDR6h
**Ubb**	0.989387231	0.017014735	Grin3a	LDR6h
**H3f3b**	0.959569061	0.03300506	Grin3a	LDR6h
**Pura**	0.851338913	0.046479244	Grin3a	LDR6h
**Apoe**	0.860776168	0.049334549	Grin3a	LDR6h
**mt-Co1**	1.401273108	8.68088814374959e-08	Npy	LDR2h
**mt-Co2**	1.375736985	6.75922244564307e-06	Npy	LDR2h

Development • Supplementary information

**mt-Co3**	1.221672731	2.34213303145834e-05	Npy	LDR2h
**Apoe**	1.420364078	0.002801304	Npy	LDR2h
**Homer1**	1.398131903	4.23157959649775e-06	Npy	LDR30m
**1700016P03Rik**	1.513379001	1.00722613085158e-05	Npy	LDR30m
**Dlx6os1**	0.859991938	5.28350898224877e-05	Npy	LDR30m
**Serpini1**	0.931368701	0.021760671	Npy	LDR30m
**Homer1**	0.982317526	0.006932905	Npy	LDR4h
**mt-Co1**	1.564645944	6.47841629343762e-10	Npy	LDR6h
**mt-Co2**	1.600464722	5.43025957149061e-08	Npy	LDR6h
**mt-Co3**	1.330636807	3.33005750133996e-07	Npy	LDR6h
**Fth1**	1.44204822	4.13125588733756e-05	Npy	LDR6h
**Srsf7**	1.244745224	0.000353066	Npy	LDR6h
**mt-Cytb**	1.203051752	0.024905743	Npy	LDR6h
**mt-Nd2**	1.217138291	0.038030624	Npy	LDR6h
**mt-Co1**	1.251917732	4.24043370761408e-10	Pval	LDR2h
**1700016P03Rik**	1.091801064	1.59365970933617e-08	Pval	LDR2h
**Apoe**	1.072965924	1.84608442068628e-06	Pval	LDR2h
**Egr1**	0.853895955	3.5656980114866e-05	Pval	LDR2h
**Atp6v0b**	0.849588317	6.47556393592096e-05	Pval	LDR2h
**1700054A03Rik**	0.785419585	0.000230247	Pval	LDR2h
**Ptprn**	0.864708346	0.000242968	Pval	LDR2h
**Sema3c**	1.771277875	0.000383528	Pval	LDR2h
**Brd9**	0.786106105	0.001901498	Pval	LDR2h
**Nrgn**	0.829004478	0.003077609	Pval	LDR2h
**Ptgds**	0.720849613	0.007655689	Pval	LDR2h
**Cox7c**	0.792396946	0.014929252	Pval	LDR2h
**Atp1a1**	0.74542607	0.018991692	Pval	LDR2h
**Hectd2os**	0.711318649	0.025380481	Pval	LDR2h
**Calm1**	0.646852723	0.029542447	Pval	LDR2h
**Slc25a3**	0.812695791	0.031073159	Pval	LDR2h
**Mir670hg**	1.837280962	0.035936722	Pval	LDR2h
**Pabpc4**	0.736807265	0.047463423	Pval	LDR2h
**1700016P03Rik**	1.969051294	3.03054761784053e-26	Pval	LDR30m
**Nr4a3**	1.614798159	4.4278974187357e-12	Pval	LDR30m
**Fosl2**	0.926101345	1.51850021825423e-06	Pval	LDR30m
**Nr4a1**	1.02161687	3.31146158567113e-06	Pval	LDR30m
**Rock2**	0.808634661	8.29360750594749e-05	Pval	LDR30m
**Rgs7bp**	0.665994826	0.000100276	Pval	LDR30m
**Pcsk1**	0.805660255	0.000124217	Pval	LDR30m

Development • Supplementary information

**Alkal2**	0.807944155	0.000138469	Pval	LDR30m
**Slc23a2**	0.699339368	0.000515445	Pval	LDR30m
**Homer1**	1.650775451	0.000531382	Pval	LDR30m
**Eepd1**	0.78098939	0.001313729	Pval	LDR30m
**Gm47423**	0.813597438	0.002690927	Pval	LDR30m
**Chgb**	0.807784512	0.00510301	Pval	LDR30m
**Per1**	0.749278415	0.007292188	Pval	LDR30m
**Arid5b**	0.777188175	0.014059802	Pval	LDR30m
**Plxnc1**	0.693620443	0.016043288	Pval	LDR30m
**Pex5**	0.729351554	0.019975814	Pval	LDR30m
**Nfkbiz**	0.720622174	0.020058897	Pval	LDR30m
**Mir670hg**	1.695650361	9.2891198938794e-31	Pval	LDR4h
**1700016P03Rik**	1.495285248	2.68635531836125e-18	Pval	LDR4h
**Homer1**	0.988221657	1.09737000621171e-10	Pval	LDR4h
**Rock2**	0.818949361	6.8380070513087e-08	Pval	LDR4h
**Alkal2**	0.924211952	1.26872712409106e-07	Pval	LDR4h
**Pex5**	0.85941285	1.21673673777114e-05	Pval	LDR4h
**Myo1e**	0.801926244	2.56579980194669e-05	Pval	LDR4h
**Cntnap5c**	0.764520282	0.000125555	Pval	LDR4h
**Slc6a17**	0.609725108	0.001527532	Pval	LDR4h
**Maml3**	0.720890304	0.014093391	Pval	LDR4h
**Lgi2**	0.652612857	0.015542044	Pval	LDR4h
**Sik3**	0.648876469	0.025350082	Pval	LDR4h
**Stk40**	0.695347987	0.025433978	Pval	LDR4h
**Gm35188**	1.097463053	3.22210715745734e-13	Pval	LDR6h
**Fth1**	1.169107765	6.80145979555461e-13	Pval	LDR6h
**Atp5a1**	1.002579966	1.66832295804705e-10	Pval	LDR6h
**Cox7c**	1.09798636	1.77481443543146e-08	Pval	LDR6h
**Hmgn3**	0.939587483	3.10728506067002e-07	Pval	LDR6h
**Apoe**	0.938559815	3.36772070522312e-07	Pval	LDR6h
**Prpf4b**	0.76183687	1.08863856041425e-06	Pval	LDR6h
**Trim35**	0.861616426	1.84003552265133e-06	Pval	LDR6h
**Calm1**	0.797456464	1.90205484814932e-06	Pval	LDR6h
**Dnaja2**	0.888839867	2.60479912985701e-06	Pval	LDR6h
**Sema3c**	0.869620828	4.92841470134965e-06	Pval	LDR6h
**Snhg11**	0.676853377	1.34298885648223e-05	Pval	LDR6h
**Gm32250**	0.845522368	2.34104021206107e-05	Pval	LDR6h
**Shisa4**	0.884507004	2.9616918980734e-05	Pval	LDR6h
**Ctnna3**	0.682182129	7.32491981649339e-05	Pval	LDR6h

**Gm28501**	0.872156136	8.00811936708986e-05	Pval	LDR6h
**Sorbs2os**	0.74620826	9.05189814781861e-05	Pval	LDR6h
**Cox7b**	0.848748321	0.000263221	Pval	LDR6h
**4933413L06Rik**	0.746095033	0.000468268	Pval	LDR6h
**mt-Co1**	1.341416802	0.000506831	Pval	LDR6h
**Atp6v0b**	0.820282322	0.000570552	Pval	LDR6h
**Rmdn1**	0.722993703	0.000650506	Pval	LDR6h
**Pdcd7**	0.731209012	0.001562074	Pval	LDR6h
**Scn9a**	0.737930554	0.002017605	Pval	LDR6h
**Phlda1**	0.835744492	0.002286327	Pval	LDR6h
**Gm15520**	0.736002363	0.003161879	Pval	LDR6h
**Mdh1**	0.775321244	0.003468384	Pval	LDR6h
**Slc25a3**	0.788076814	0.003499049	Pval	LDR6h
**Crebzf**	0.724460819	0.003530295	Pval	LDR6h
**Nrgn**	0.75012037	0.003666976	Pval	LDR6h
**Tmsb4x**	0.849143218	0.00367519	Pval	LDR6h
**Gm48678**	0.767146248	0.003918781	Pval	LDR6h
**Cox8a**	0.728914914	0.004525819	Pval	LDR6h
**mt-Co3**	1.183362832	0.004854369	Pval	LDR6h
**Rps21**	0.71512625	0.004892888	Pval	LDR6h
**Ubb**	0.80075796	0.005615504	Pval	LDR6h
**Malat1**	1.312386824	0.00777307	Pval	LDR6h
**Nrg3os**	0.642990949	0.008721078	Pval	LDR6h
**Bmyc**	0.739474998	0.00959358	Pval	LDR6h
**Pcsk1n**	0.715496232	0.014793274	Pval	LDR6h
**Gsdme**	0.6269244	0.014889378	Pval	LDR6h
**Gm46367**	0.73312434	0.014984525	Pval	LDR6h
**Pla2g3**	0.691218531	0.016927528	Pval	LDR6h
**Gm15614**	0.747862041	0.01926609	Pval	LDR6h
**Cox4i1**	0.669921079	0.020971163	Pval	LDR6h
**Tspyl4**	0.678246522	0.025542432	Pval	LDR6h
**Psmc6**	0.702298051	0.034819897	Pval	LDR6h
**Atxn7l3b**	0.67197484	0.035406894	Pval	LDR6h
**Gm43376**	0.738424669	0.036614272	Pval	LDR6h
**Ckb**	0.720059691	0.03820453	Pval	LDR6h
**Rbm39**	0.639811845	0.046476931	Pval	LDR6h
**3110021N24Rik**	0.627631284	0.048574055	Pval	LDR6h
**Mir670hg**	2.299222756	8.97577553149088e-12	Vip	LDR2h
**Ptprn**	1.159097903	0.00315566	Vip	LDR2h

**mt-Co3**	1.167398967	0.004446464	Vip	LDR2h
**AC149090.1**	1.276434608	0.024542907	Vip	LDR2h
**Fth1**	1.389505447	0.024990295	Vip	LDR2h
**mt-Co1**	1.159106683	0.03404771	Vip	LDR2h
**1700016P03Rik**	3.03749102	5.88837865123592e-28	Vip	LDR30m
**Homer1**	2.33633243	1.49029192653244e-25	Vip	LDR30m
**Nr4a3**	3.419165249	1.65004434372229e-16	Vip	LDR30m
**Nr4a1**	1.861919837	5.80759004758254e-10	Vip	LDR30m
**Dlx6os1**	1.169701501	1.31697791846962e-09	Vip	LDR30m
**Gad1**	1.463336668	2.16146148530203e-08	Vip	LDR30m
**Fosl2**	1.917109565	5.17637887081991e-07	Vip	LDR30m
**Gm47423**	1.620523325	8.97790180920733e-07	Vip	LDR30m
**Fam107b**	1.790691873	9.29554586871969e-07	Vip	LDR30m
**Pcsk1**	1.644572275	9.53131065018633e-06	Vip	LDR30m
**Per1**	1.56969134	1.83030847684867e-05	Vip	LDR30m
**Rab6a**	1.348098069	1.86045195419079e-05	Vip	LDR30m
**Atp2c1**	1.200997323	4.88048181104499e-05	Vip	LDR30m
**Nr4a2**	1.819472911	6.90462593632558e-05	Vip	LDR30m
**Coq10b**	1.588624333	0.00013803	Vip	LDR30m
**Fam49a**	1.181119511	0.000168401	Vip	LDR30m
**Arl5b**	1.485815868	0.000780616	Vip	LDR30m
**Fosb**	1.46731004	0.001333375	Vip	LDR30m
**Mir670hg**	1.134503875	0.001972805	Vip	LDR30m
**Rgs8**	1.183002384	0.003025858	Vip	LDR30m
**Eepd1**	1.254707011	0.00400209	Vip	LDR30m
**Crh**	1.331385234	0.0058248	Vip	LDR30m
**Sik1**	1.439312052	0.009713878	Vip	LDR30m
**Ptprn**	1.002008266	0.009846071	Vip	LDR30m
**Ube2ql1**	1.311307923	0.011947824	Vip	LDR30m
**Rgs7bp**	1.019235763	0.019268903	Vip	LDR30m
**Slc23a2**	1.09211005	0.021044044	Vip	LDR30m
**Rock2**	0.933268028	0.030928051	Vip	LDR30m
**Mir670hg**	1.629881882	2.94224205690294e-13	Vip	LDR4h
**Pcdh11x**	1.564550113	4.0259238196809e-08	Vip	LDR4h
**Htr1f**	1.520355157	1.7427377961423e-05	Vip	LDR4h
**1700016P03Rik**	1.456088342	4.66433094302911e-05	Vip	LDR4h
**9530059O14Rik**	1.145782793	9.24868309232623e-05	Vip	LDR4h
**Pde1a**	1.13163804	0.000765767	Vip	LDR4h
**Pcdh9**	0.692392535	0.000914224	Vip	LDR4h

**Hunk**	1.23298983	0.003944254	Vip	LDR4h
**Csmd2**	0.952510767	0.004388491	Vip	LDR4h
**Nrg1**	0.854523727	0.006066973	Vip	LDR4h
**Xylt1**	1.249112439	0.012347651	Vip	LDR4h
**Zswim6**	0.950055835	0.028178983	Vip	LDR4h
**mt-Co3**	1.338027559	0.001500475	Vip	LDR6h
**Bdnf**	1.477093015	1.5343191900312e-55	L23	LDR2h
**C1ql3**	1.288538237	5.82745340834629e-55	L23	LDR2h
**Rnd3**	0.833011538	8.58638289649298e-46	L23	LDR2h
**1700016P03Rik**	1.946184874	1.17720336257419e-45	L23	LDR2h
**Npas4**	1.271767257	3.83141466678813e-45	L23	LDR2h
**Nr4a2**	0.659460326	3.68794402154839e-42	L23	LDR2h
**Scg2**	0.85135281	1.70207340179985e-40	L23	LDR2h
**Mas1**	0.916228379	1.25021220477659e-39	L23	LDR2h
**Arc**	1.246047915	1.05291426225399e-37	L23	LDR2h
**Ptgs2**	0.71989745	1.99359589437406e-37	L23	LDR2h
**Nr4a1**	1.203649276	8.67885234085755e-36	L23	LDR2h
**Grasp**	1.077097633	1.55093426681318e-35	L23	LDR2h
**Tiparp**	0.878926935	7.67029392921444e-35	L23	LDR2h
**Fosb**	0.683291103	2.74859847163469e-34	L23	LDR2h
**Mir670hg**	1.259764416	9.62828585732387e-34	L23	LDR2h
**Egr4**	0.711542971	3.06922858619685e-33	L23	LDR2h
**Egr3**	0.919806991	1.33273386116028e-32	L23	LDR2h
**Inhba**	1.040037697	4.76236171108772e-32	L23	LDR2h
**Ptprn**	1.429031596	6.45659824413171e-32	L23	LDR2h
**Gm34544**	1.182014976	1.17481296375881e-31	L23	LDR2h
**Fos**	0.791002507	3.85143109769527e-31	L23	LDR2h
**Nr4a3**	0.820301088	9.96821041864553e-31	L23	LDR2h
**Gadd45g**	0.723126145	1.86242726474756e-28	L23	LDR2h
**Ppme1**	1.42768547	1.97624934021388e-27	L23	LDR2h
**Rgs4**	0.738747539	3.74485852139791e-26	L23	LDR2h
**Gm37229**	0.633222882	7.15190361442562e-26	L23	LDR2h
**Diras2**	0.941601099	1.3263231156521e-25	L23	LDR2h
**Rasl11b**	0.709996043	1.42201531987115e-25	L23	LDR2h
**Hspa8**	0.899569934	3.28469575071822e-25	L23	LDR2h
**Nrn1**	1.176142342	2.63913009558193e-23	L23	LDR2h
**Junb**	1.162659915	9.23975371653532e-22	L23	LDR2h
**mt-Co3**	1.286294184	1.52426171432678e-21	L23	LDR2h
**4933413L06Rik**	0.644039434	3.59513659243506e-20	L23	LDR2h

Development • Supplementary information

**Jund**	0.624760374	5.66426598440241e-20	L23	LDR2h
**Nrp2**	0.773810625	7.78186683578438e-20	L23	LDR2h
**Btg2**	1.031058682	7.97848411877648e-20	L23	LDR2h
**Ptgds**	0.717686345	1.34242127347751e-19	L23	LDR2h
**Dusp6**	0.594564329	8.78478365874994e-19	L23	LDR2h
**Cx3cl1**	0.889220777	2.21307540924892e-18	L23	LDR2h
**Nptx2**	1.222653464	2.5729376853987e-18	L23	LDR2h
**Pcsk1**	0.907096049	5.79517455797264e-18	L23	LDR2h
**Egr1**	0.980443173	1.16753580696216e-17	L23	LDR2h
**Tsnax**	1.238666674	1.47018015488699e-17	L23	LDR2h
**Kcnv1**	0.596939836	4.01353739855994e-17	L23	LDR2h
**Hectd2os**	0.993153222	8.90119420229365e-17	L23	LDR2h
**Fosl2**	0.964027283	9.3980293322149e-17	L23	LDR2h
**Nefl**	0.608222098	1.73816859611619e-16	L23	LDR2h
**Tpt1**	0.765598969	1.93678959748669e-16	L23	LDR2h
**1700054A03Rik**	0.691813944	4.43459797304636e-16	L23	LDR2h
**Nrsn1**	0.810902391	2.88731039904809e-15	L23	LDR2h
**Cap1**	0.769174337	7.40639805203044e-15	L23	LDR2h
**Slc25a3**	1.019590314	7.69480757081236e-15	L23	LDR2h
**mt-Co1**	1.333801116	2.78384682127131e-14	L23	LDR2h
**Fbxo33**	0.720867988	5.49629375191352e-14	L23	LDR2h
**Irs2**	0.617219092	7.54106102593126e-14	L23	LDR2h
**Jcad**	0.751262852	1.41838738612478e-13	L23	LDR2h
**Spry2**	0.643341778	2.49612691793519e-13	L23	LDR2h
**Ifrd1**	0.807908299	3.78097217799526e-13	L23	LDR2h
**Rapgef5**	1.380011569	2.99984617832899e-12	L23	LDR2h
**P4ha1**	0.663836125	3.75474735345244e-12	L23	LDR2h
**Scg3**	0.649099121	1.02351275637595e-11	L23	LDR2h
**Gm14636**	0.638537574	1.08377015827522e-11	L23	LDR2h
**Purb**	0.669523601	1.13794435116923e-11	L23	LDR2h
**Nefm**	0.771285153	1.25131057210327e-11	L23	LDR2h
**A430090L17Rik**	0.794358439	1.60159108613979e-11	L23	LDR2h
**mt-Co2**	1.175889846	3.12135608871695e-11	L23	LDR2h
**Ina**	0.593536797	4.08990240682828e-11	L23	LDR2h
**Rpl6**	0.802545879	7.91271466226967e-11	L23	LDR2h
**Homer1**	0.842435553	3.44537812683614e-10	L23	LDR2h
**Pdp1**	0.733817563	4.43679365174412e-10	L23	LDR2h
**Trpc6**	0.849944691	6.84701647481443e-10	L23	LDR2h
**Ywhag**	0.633489494	8.15768055636396e-10	L23	LDR2h

Development • Supplementary information

**Gm13684**	0.710386623	2.18740578588724e-09	L23	LDR2h
**Eif1**	0.739393721	2.57239356605506e-09	L23	LDR2h
**Lamp5**	0.810164778	3.47931839770238e-09	L23	LDR2h
**Baz1a**	1.51751328	1.11313494233574e-08	L23	LDR2h
**Rasgrp1**	0.76878871	2.00190995966928e-08	L23	LDR2h
**Nrd1**	0.92911505	2.40623678396112e-08	L23	LDR2h
**Eef1a1**	0.620099257	3.69661540873181e-08	L23	LDR2h
**Nt5dc3**	0.639308638	4.21283844644179e-08	L23	LDR2h
**Gm35188**	1.165182525	5.04579989177831e-08	L23	LDR2h
**Brinp3**	0.808500043	5.73031944334329e-08	L23	LDR2h
**Gm17202**	0.641980946	1.16367284835478e-07	L23	LDR2h
**Gm17231**	0.774523632	1.23784253047903e-07	L23	LDR2h
**Pcdh15**	0.663224598	1.2640077897851e-07	L23	LDR2h
**Ephx4**	0.628105275	1.52548043392244e-07	L23	LDR2h
**Pabpc4**	0.638418801	1.57818001378387e-07	L23	LDR2h
**Itgav**	0.865463879	1.64856489571279e-07	L23	LDR2h
**Rpl41**	0.659788584	1.99330691524865e-07	L23	LDR2h
**Mdh1**	0.588718305	2.18831533087208e-07	L23	LDR2h
**Rpl13**	0.606082774	2.48662383596708e-07	L23	LDR2h
**Gm15478**	0.683472717	2.64964287625692e-07	L23	LDR2h
**Atp1a1**	0.75214916	3.16323583275013e-07	L23	LDR2h
**Atp6v0b**	0.813357746	4.61081100755413e-07	L23	LDR2h
**Vmp1**	0.626384883	5.21245338349484e-07	L23	LDR2h
**Cox8a**	0.713664684	6.23411879442296e-07	L23	LDR2h
**Igsf9b**	0.702233664	8.62362831923437e-07	L23	LDR2h
**4930415C11Rik**	1.895101086	9.95725412872267e-07	L23	LDR2h
**Numb**	0.823906051	1.91504553493891e-06	L23	LDR2h
**Gm21798**	0.688993913	2.33711244401384e-06	L23	LDR2h
**Pak6**	0.813824216	2.56230972169144e-06	L23	LDR2h
**Oprm1**	0.814160461	4.07582825843715e-06	L23	LDR2h
**Sema3c**	1.604741556	4.79881481335088e-06	L23	LDR2h
**Arhgap5**	0.62148344	5.95968373409361e-06	L23	LDR2h
**Gm14211**	0.602697574	6.48183175076091e-06	L23	LDR2h
**mt-Atp6**	1.030118686	8.94402034162067e-06	L23	LDR2h
**Cox4i1**	0.668220659	1.10873433523995e-05	L23	LDR2h
**Gm16351**	0.786832766	1.14874914537238e-05	L23	LDR2h
**Ppp1r1a**	0.599984941	1.2265339455745e-05	L23	LDR2h
**Mapk4**	1.139475697	1.33926092188967e-05	L23	LDR2h
**Adam2**	0.980868848	1.54005576687791e-05	L23	LDR2h

Development • Supplementary information

**Gm15520**	0.732203612	3.33286125402479e-05	L23	LDR2h
**Pcdh17**	0.598212251	3.43086775477719e-05	L23	LDR2h
**Tmem243**	0.666114826	4.41862438296224e-05	L23	LDR2h
**Gm6994**	0.600924366	6.10148977746751e-05	L23	LDR2h
**Tmtc2**	0.832153304	8.5379681282338e-05	L23	LDR2h
**Pak7**	0.760727498	8.75619305503775e-05	L23	LDR2h
**Actb**	0.595711994	8.95164911633566e-05	L23	LDR2h
**Apoe**	1.02050735	0.000113749	L23	LDR2h
**Rnf144b**	0.602906193	0.000128722	L23	LDR2h
**Gnal**	0.690471853	0.000142301	L23	LDR2h
**1700085D07Rik**	0.691227857	0.000163502	L23	LDR2h
**Klhl2**	0.587197928	0.000220914	L23	LDR2h
**Ptchd4**	0.73433256	0.000315241	L23	LDR2h
**R3hdm1**	0.685462596	0.000326856	L23	LDR2h
**Gm37240**	0.741679164	0.000460861	L23	LDR2h
**Arap2**	0.778225603	0.000861886	L23	LDR2h
**Gm13883**	0.772299085	0.001009948	L23	LDR2h
**Man1a**	0.6696515	0.001209582	L23	LDR2h
**Fth1**	1.329210819	0.002067556	L23	LDR2h
**Ctnna3**	0.887670957	0.002327654	L23	LDR2h
**Ptn**	0.640771196	0.003025192	L23	LDR2h
**Dot1l**	0.748762492	0.003812849	L23	LDR2h
**Brinp1**	0.782836234	0.005241729	L23	LDR2h
**Srsf7**	0.774499729	0.007671924	L23	LDR2h
**Prmt8**	0.648859026	0.010153277	L23	LDR2h
**Slc6a17**	0.761163215	0.010696807	L23	LDR2h
**Epha10**	0.76028326	0.013140493	L23	LDR2h
**Cdh6**	0.671805646	0.024233158	L23	LDR2h
**Maml3**	1.974286251	0.030199088	L23	LDR2h
**Pde7b**	0.879576754	0.041116207	L23	LDR2h
**Nr4a3**	2.969416771	8.46667076299018e-269	L23	LDR30m
**Gm47423**	2.57749454	9.65746666705844e-232	L23	LDR30m
**Egr3**	2.039138514	5.61271756089834e-140	L23	LDR30m
**Homer1**	2.685349717	1.79133164553136e-123	L23	LDR30m
**Per1**	1.683074065	1.6940403478407e-121	L23	LDR30m
**Arl5b**	2.078295674	2.84427397065683e-119	L23	LDR30m
**Rheb**	1.833171253	1.65898022900263e-110	L23	LDR30m
**Frmd6**	1.822073225	1.97625352592935e-110	L23	LDR30m
**Sik2**	2.343162563	5.12287643536193e-108	L23	LDR30m

Development • Supplementary information

**Ube2ql1**	1.503535164	9.18862623591962e-108	L23	LDR30m
**Stk40**	1.505936679	1.05268015831599e-102	L23	LDR30m
**Irs2**	1.76841515	6.18808749299079e-100	L23	LDR30m
**Arhgef3**	2.050591886	3.31878979741569e-95	L23	LDR30m
**Slc25a25**	1.762049578	4.78145666199948e-93	L23	LDR30m
**Kdm7a**	1.93316427	1.98642934992317e-86	L23	LDR30m
**Bdnf**	1.625881583	1.55979678392004e-82	L23	LDR30m
**Nr4a1**	1.686198937	4.64133203144706e-68	L23	LDR30m
**Fosb**	0.999440303	1.66527586283971e-67	L23	LDR30m
**Arid3b**	1.154291035	1.32641837229458e-66	L23	LDR30m
**Zswim6**	1.340761455	2.61200220748501e-66	L23	LDR30m
**Plcxd2**	1.009309117	1.21427077670968e-64	L23	LDR30m
**Sik1**	1.016340162	5.65268685535256e-64	L23	LDR30m
**Grasp**	1.128846759	2.07870852625531e-63	L23	LDR30m
**Gm17231**	1.426516283	7.5414566598475e-63	L23	LDR30m
**Kdm6b**	1.045654701	7.36803227534753e-62	L23	LDR30m
**Arpc2**	1.183178903	5.52394299541263e-59	L23	LDR30m
**Npas4**	1.079070462	9.37154059084903e-59	L23	LDR30m
**Ina**	1.428642199	7.82373386158006e-58	L23	LDR30m
**Hmgcr**	1.094725225	3.6402640466589e-57	L23	LDR30m
**Lonrf1**	1.129055162	5.96256810257483e-57	L23	LDR30m
**Osbpl8**	1.297955397	9.21753313580789e-55	L23	LDR30m
**Cpeb3**	1.14556525	1.44226573391022e-54	L23	LDR30m
**Skil**	1.033469575	2.1990226076642e-54	L23	LDR30m
**Vmp1**	1.220755478	2.1227241977316e-53	L23	LDR30m
**Fbxo33**	1.356743919	7.91734408016089e-53	L23	LDR30m
**Nr4a2**	2.509357714	8.52190569320178e-53	L23	LDR30m
**Clstn3**	1.308434893	2.32223795094893e-52	L23	LDR30m
**Mapk4**	1.100255166	5.2895676356346e-52	L23	LDR30m
**Btaf1**	1.208040532	1.19746248408531e-51	L23	LDR30m
**Scg3**	1.21838024	1.41918214968639e-51	L23	LDR30m
**Mbp**	1.402875447	2.55232990403053e-51	L23	LDR30m
**Per2**	1.124195749	2.63162937996008e-51	L23	LDR30m
**Ubtd2**	1.314860262	1.01134622101048e-50	L23	LDR30m
**Fosl2**	1.702940986	2.26690918269483e-50	L23	LDR30m
**Med14**	1.504005754	3.91293304527241e-50	L23	LDR30m
**Coq10b**	1.262123241	6.42653454653406e-50	L23	LDR30m
**Hspa4**	1.244292842	8.51807135557128e-50	L23	LDR30m
**Rcc2**	1.037491589	6.02270523438969e-49	L23	LDR30m

Development • Supplementary information

**Midn**	0.810994536	8.00963294449841e-49	L23	LDR30m
**Ntrk2**	1.534163197	8.89345028307174e-48	L23	LDR30m
**Jdp2**	1.542200556	5.2821550810007e-47	L23	LDR30m
**Rnf217**	1.482829006	2.8770969768004e-46	L23	LDR30m
**Nrd1**	1.782799946	1.68814199720221e-45	L23	LDR30m
**Nudt4**	1.044085146	5.13324432169156e-44	L23	LDR30m
**Trib1**	0.739487721	6.81267572093095e-44	L23	LDR30m
**Dusp14**	0.919625313	7.31098398863303e-44	L23	LDR30m
**Ciart**	0.789841351	4.79300983664421e-43	L23	LDR30m
**Pitpna**	0.979104699	8.02772774115885e-43	L23	LDR30m
**Ap2b1**	1.076384499	8.31376027492482e-43	L23	LDR30m
**Nap1l1**	1.049707479	2.33913350606266e-42	L23	LDR30m
**Nptx2**	0.709475791	3.56171282515202e-42	L23	LDR30m
**Arih2**	0.94327684	4.00436965387786e-41	L23	LDR30m
**Baiap2**	1.187041674	1.0535480644666e-40	L23	LDR30m
**Fbl**	1.110240243	1.65484286885633e-40	L23	LDR30m
**Tulp4**	1.165709724	2.65974404000671e-40	L23	LDR30m
**Galnt9**	1.335482512	2.78024033619743e-40	L23	LDR30m
**Ankrd33b**	1.161688707	5.76305995961178e-40	L23	LDR30m
**1700016P03Rik**	3.248843499	1.20924911598228e-39	L23	LDR30m
**Efhd2**	0.853866724	7.10891270490618e-39	L23	LDR30m
**Rims4**	1.075508036	1.29413814979465e-38	L23	LDR30m
**Cwc25**	0.786858412	2.89683808053392e-38	L23	LDR30m
**Ptprn**	1.240359655	2.713248488076e-37	L23	LDR30m
**Gm28294**	0.700802826	3.27197944281593e-37	L23	LDR30m
**Synj2**	1.024744129	6.27517276042325e-37	L23	LDR30m
**Cltc**	0.956455048	1.61222182429379e-36	L23	LDR30m
**Adora1**	0.829818631	4.53559561727812e-36	L23	LDR30m
**Prag1**	0.930207664	1.37077874231827e-35	L23	LDR30m
**Hsph1**	0.831071524	3.02061978411986e-35	L23	LDR30m
**Txndc11**	0.943755597	5.57278852942792e-35	L23	LDR30m
**Cds1**	0.887986599	7.71196368417491e-35	L23	LDR30m
**Ctnnd1**	0.933829478	1.1710103635144e-34	L23	LDR30m
**Myh9**	0.893054112	1.23180601107294e-34	L23	LDR30m
**Tiparp**	2.502650667	1.74724370532455e-34	L23	LDR30m
**R3hdm2**	0.981160394	2.53889063312427e-34	L23	LDR30m
**Mn1**	0.90007157	6.33481377754288e-34	L23	LDR30m
**Gnai3**	0.98648141	8.21374624669139e-34	L23	LDR30m
**Sidt1**	0.936828323	1.96122432926781e-33	L23	LDR30m

**Ttpal**	0.73041747	2.40658941555701e-33	L23	LDR30m
**Ece1**	1.255944835	3.27137448600417e-33	L23	LDR30m
**Mest**	1.284571325	3.896268391595e-33	L23	LDR30m
**Hsd17b12**	0.739857799	4.60185884448913e-33	L23	LDR30m
**Baz1a**	0.746235446	4.99035048877501e-33	L23	LDR30m
**P4ha1**	1.044655148	1.81232293712353e-32	L23	LDR30m
**Spred1**	1.399182776	2.29403169577445e-32	L23	LDR30m
**Pcsk1**	3.038003697	3.04937123039201e-32	L23	LDR30m
**Slc20a2**	0.930516009	4.10019397594424e-32	L23	LDR30m
**Atp1a1**	1.012974664	5.84613547516795e-32	L23	LDR30m
**Sik3**	1.261009564	6.61402870017154e-32	L23	LDR30m
**Gng2**	0.935155082	1.95107800800294e-31	L23	LDR30m
**Ifrd1**	1.145793488	1.97907760209804e-31	L23	LDR30m
**Ivns1abp**	0.930662857	3.43678966511077e-31	L23	LDR30m
**Smad1**	0.966296278	4.53524617087828e-31	L23	LDR30m
**Mapk6**	0.763315707	9.69348196440441e-31	L23	LDR30m
**Numb**	0.814128873	1.97044033704574e-30	L23	LDR30m
**Eprs**	0.819907622	3.07812638735321e-30	L23	LDR30m
**Sec24a**	0.863316358	4.74828566553022e-30	L23	LDR30m
**Mxi1**	0.83892594	8.35450793270278e-30	L23	LDR30m
**Golph3**	0.759131967	1.14570246824594e-29	L23	LDR30m
**Gm13684**	1.060290278	1.77347721482366e-29	L23	LDR30m
**Ctps**	0.798161647	2.34977356819851e-29	L23	LDR30m
**Zfp948**	0.794920315	2.37253879127885e-29	L23	LDR30m
**Ago3**	0.971173619	2.90429764940598e-29	L23	LDR30m
**Lonp2**	0.970866346	3.05391836101747e-29	L23	LDR30m
**Ndel1**	0.973117435	3.37401848012237e-29	L23	LDR30m
**Cx3cl1**	0.925176024	4.12548626287789e-29	L23	LDR30m
**Slc6a17**	0.921310179	7.80735030072122e-29	L23	LDR30m
**Nmnat2**	0.896817958	1.10689359961183e-28	L23	LDR30m
**Cystm1**	0.693345572	1.74034675778046e-28	L23	LDR30m
**Por**	0.761819094	2.05804781961258e-28	L23	LDR30m
**Cap1**	0.812299939	2.19438344843907e-28	L23	LDR30m
**Ppme1**	0.985372706	2.47951180358496e-28	L23	LDR30m
**Cxadr**	0.770208689	3.14810911099354e-28	L23	LDR30m
**Rundc1**	0.695942104	3.89667300764579e-28	L23	LDR30m
**Mia3**	0.848684941	4.65904641815025e-28	L23	LDR30m
**Camk1g**	0.669962493	1.32414832061024e-27	L23	LDR30m
**Hnrnpll**	1.065736043	1.91063971902631e-27	L23	LDR30m

Development • Supplementary information

**Cpeb4**	1.243208323	6.33683151424231e-27	L23	LDR30m
**Smg7**	0.890093081	1.24500671587754e-26	L23	LDR30m
**Cdc42ep3**	0.91942111	1.84526139974185e-26	L23	LDR30m
**Dok5**	0.899411293	5.15060842220868e-26	L23	LDR30m
**Smap2**	0.769262979	5.17379745953693e-26	L23	LDR30m
**Trim9**	0.826102681	7.58107169534822e-26	L23	LDR30m
**Prkar2a**	0.724931422	8.2467064883192e-26	L23	LDR30m
**Tmem38b**	0.648947066	8.87069662744668e-26	L23	LDR30m
**Dnajb5**	0.886468228	1.06496538524518e-25	L23	LDR30m
**Egr1**	0.896599165	1.09382090001386e-25	L23	LDR30m
**Zdbf2**	2.012190546	1.49464938282152e-25	L23	LDR30m
**Tfrc**	0.787369886	1.508816609621e-25	L23	LDR30m
**4931406P16Rik**	0.814094672	1.59546984710016e-25	L23	LDR30m
**Pmepa1**	1.063076971	2.14185246374884e-25	L23	LDR30m
**Zhx2**	0.819336975	2.80951053568404e-25	L23	LDR30m
**Sptbn2**	0.960942205	3.70404026054266e-25	L23	LDR30m
**Gm3294**	0.845948634	4.06309532091496e-25	L23	LDR30m
**Egr4**	0.61602635	5.09354402095756e-25	L23	LDR30m
**Txnrd1**	0.69721475	5.73977904629639e-25	L23	LDR30m
**Pvr**	0.623848243	8.14918519950504e-25	L23	LDR30m
**Cbfb**	0.797459772	8.69330138159259e-25	L23	LDR30m
**Ephb3**	0.647496241	9.28956883085247e-25	L23	LDR30m
**Nefm**	0.864583965	9.33261971035519e-25	L23	LDR30m
**Hspa8**	0.877139946	1.08802157250075e-24	L23	LDR30m
**Lrrk2**	0.945775148	2.19087555338322e-24	L23	LDR30m
**Cbarp**	0.870203637	2.48026775580968e-24	L23	LDR30m
**Tpm3**	0.786470841	3.23696183938862e-24	L23	LDR30m
**Dennd5b**	0.93062449	4.99292712130064e-24	L23	LDR30m
**4930599N23Rik**	0.675710528	5.25421023747685e-24	L23	LDR30m
**Dusp1**	0.637830503	6.35875673950456e-24	L23	LDR30m
**4921511C10Rik**	0.73175248	8.04395614190884e-24	L23	LDR30m
**Hivep2**	0.722309056	9.50937631008445e-24	L23	LDR30m
**Myo1e**	0.693509831	1.02125072844068e-23	L23	LDR30m
**Tacc1**	0.829802118	2.33189718094428e-23	L23	LDR30m
**Mast3**	0.825771347	3.12432646907042e-23	L23	LDR30m
**Zbtb11**	0.869322794	3.94418677726612e-23	L23	LDR30m
**Dusp16**	0.63257139	4.31203403819165e-23	L23	LDR30m
**Gm10563**	0.604498877	7.03229412538146e-23	L23	LDR30m
**1600020E01Rik**	0.764882736	7.73833223578043e-23	L23	LDR30m

**Erf**	0.647535379	8.19101661790915e-23	L23	LDR30m
**Kif5c**	0.784521623	8.28530802869086e-23	L23	LDR30m
**Cry2**	0.862861041	1.41202352994993e-22	L23	LDR30m
**Hipk3**	0.752533495	1.99796280635183e-22	L23	LDR30m
**Coro1c**	0.663254322	4.10808989362558e-22	L23	LDR30m
**Ppp2ca**	0.778391704	5.44895319767553e-22	L23	LDR30m
**Lemd3**	0.831900489	6.25828249175364e-22	L23	LDR30m
**R3hdm1**	0.859222736	9.12734766163341e-22	L23	LDR30m
**Ddx3y**	0.83784352	1.48443114931712e-21	L23	LDR30m
**Gm42937**	0.630711704	2.80686874252298e-21	L23	LDR30m
**Prickle1**	0.787761902	3.85438019935145e-21	L23	LDR30m
**Dnajc1**	1.061709582	4.71107812910302e-21	L23	LDR30m
**Asap1**	0.87962856	4.91108288721757e-21	L23	LDR30m
**Etv5**	1.938926163	1.20715230368593e-20	L23	LDR30m
**Smarca5**	0.895924345	1.58347617504443e-20	L23	LDR30m
**Rab6b**	1.001498706	2.24680510963265e-20	L23	LDR30m
**Tbc1d9**	0.830909797	3.41950083076429e-20	L23	LDR30m
**Atp6v0d1**	0.744641646	3.44444861101464e-20	L23	LDR30m
**Tm9sf3**	0.704472347	4.53590450838714e-20	L23	LDR30m
**Arf4**	0.687602552	7.69406747785213e-20	L23	LDR30m
**Gpr19**	0.629564091	1.0878444024637e-19	L23	LDR30m
**Clip2**	0.87063881	1.11167912402113e-19	L23	LDR30m
**Usp36**	0.735953711	1.12510291421233e-19	L23	LDR30m
**Btg3**	0.695004441	1.66548122876639e-19	L23	LDR30m
**Abhd2**	0.902204763	1.72976975797318e-19	L23	LDR30m
**Pak1**	0.848644278	1.73484853848399e-19	L23	LDR30m
**Trak1**	0.939038581	2.71495373866767e-19	L23	LDR30m
**Sdcbp**	0.794115383	3.24342615716073e-19	L23	LDR30m
**Lncpint**	0.851135914	3.66301849990037e-19	L23	LDR30m
**Pip5k1a**	0.679697058	4.62577969208376e-19	L23	LDR30m
**Slc25a3**	1.094984207	4.71470459541868e-19	L23	LDR30m
**Prmt8**	1.002318826	7.38783271062013e-19	L23	LDR30m
**Ywhag**	0.696873387	8.36467042759622e-19	L23	LDR30m
**Kpna1**	0.74686956	8.5316266856417e-19	L23	LDR30m
**Rock2**	0.933060362	1.1762518268538e-18	L23	LDR30m
**Eif4a1**	0.631912044	2.05752191364636e-18	L23	LDR30m
**Fscn1**	0.745594098	2.05911038465593e-18	L23	LDR30m
**Sept7**	0.783419749	2.09916256888866e-18	L23	LDR30m
**Itgav**	1.05644201	3.02677680324195e-18	L23	LDR30m

Development • Supplementary information

**Acsl4**	0.966288902	3.29084867047537e-18	L23	LDR30m
**Stk38l**	0.593615742	4.23462348373538e-18	L23	LDR30m
**Nectin1**	0.753604683	8.5848339990961e-18	L23	LDR30m
**Psd3**	1.007611239	1.04108750843e-17	L23	LDR30m
**Spred2**	0.964775484	1.26958703864618e-17	L23	LDR30m
**Gramd1b**	0.771402922	1.33384891046605e-17	L23	LDR30m
**Phyhipl**	0.740665525	2.29613543458717e-17	L23	LDR30m
**Etf1**	0.591586884	2.33112015948609e-17	L23	LDR30m
**Cnnm1**	0.624015757	3.07562469262843e-17	L23	LDR30m
**Arih1**	0.859874626	3.19922316721638e-17	L23	LDR30m
**Rgs7bp**	0.831119505	5.75619658003611e-17	L23	LDR30m
**Gm42941**	0.610310433	5.92328011378633e-17	L23	LDR30m
**Stat3**	0.619245619	7.78587935264048e-17	L23	LDR30m
**Efcab6**	0.636383307	8.04464565965491e-17	L23	LDR30m
**Atp2a2**	0.683328177	9.90501546322674e-17	L23	LDR30m
**Rabgef1**	0.591655712	1.63321937332858e-16	L23	LDR30m
**Tbc1d1**	0.873102928	3.43356147591201e-16	L23	LDR30m
**Btbd8**	0.804797579	3.70931307109568e-16	L23	LDR30m
**4933424G05Rik**	0.847010096	4.34069793617256e-16	L23	LDR30m
**Ern1**	0.627295936	4.46202660719248e-16	L23	LDR30m
**1700110K17Rik**	0.737622362	5.5638408729848e-16	L23	LDR30m
**Kras**	0.917764527	7.65226160825172e-16	L23	LDR30m
**Klf9**	0.674043087	8.39293706651802e-16	L23	LDR30m
**Gabbr1**	0.670320189	1.32998660072323e-15	L23	LDR30m
**Mtmr12**	0.589052667	1.65658097343869e-15	L23	LDR30m
**Rgs4**	0.608407437	2.11214241732574e-15	L23	LDR30m
**Pde7b**	0.591760591	3.34474143968074e-15	L23	LDR30m
**Cabp1**	0.777503333	3.4905357230074e-15	L23	LDR30m
**Nrip1**	0.669684749	5.22224702012248e-15	L23	LDR30m
**Fkbp1a**	0.69873167	7.53235326322503e-15	L23	LDR30m
**Ttbk1**	0.679866936	7.60455249548157e-15	L23	LDR30m
**Rab6a**	0.804335747	9.84648291908845e-15	L23	LDR30m
**Hectd2**	0.718993099	1.49842946449363e-14	L23	LDR30m
**Fzd3**	0.640967411	1.51089883613143e-14	L23	LDR30m
**Epha10**	0.733781634	1.66211620286012e-14	L23	LDR30m
**Foxo3**	0.701263755	1.69249425914965e-14	L23	LDR30m
**Nrn1**	0.868173732	2.12927906625105e-14	L23	LDR30m
**Brinp1**	0.965438432	3.33338157851647e-14	L23	LDR30m
**Klhl2**	0.63606485	4.24597284088747e-14	L23	LDR30m

**Psme4**	0.598644193	4.37652056364977e-14	L23	LDR30m
**Dnajc21**	0.656170791	4.78128186710583e-14	L23	LDR30m
**Slc2a13**	0.780521852	5.27215042131761e-14	L23	LDR30m
**Bicdl1**	0.667005618	6.66422350686371e-14	L23	LDR30m
**Hdac5**	0.603685616	8.21059160863942e-14	L23	LDR30m
**Stx1b**	0.602706367	8.46763210153619e-14	L23	LDR30m
**Csnk1a1**	0.600338428	9.23305439925992e-14	L23	LDR30m
**Ccm2**	0.587511541	2.02796394804478e-13	L23	LDR30m
**Fmr1**	0.620553458	2.61005470119906e-13	L23	LDR30m
**Ephx4**	0.619020644	3.59877519112041e-13	L23	LDR30m
**St8sia5**	0.658803166	4.26078191963267e-13	L23	LDR30m
**Gfod1**	0.723717528	1.76930536199707e-12	L23	LDR30m
**Cmip**	0.88482438	2.14473356539398e-12	L23	LDR30m
**Mgrn1**	0.658035789	2.23624711997802e-12	L23	LDR30m
**Gclc**	0.611842615	2.43043470875622e-12	L23	LDR30m
**Samd8**	0.697568866	3.03838934595466e-12	L23	LDR30m
**Atxn7**	0.597299993	4.88737508152683e-12	L23	LDR30m
**Ago2**	0.649906924	5.43539723348348e-12	L23	LDR30m
**Csnk1d**	0.700061515	6.24840899108679e-12	L23	LDR30m
**Gm3764**	0.6526573	7.32883056199949e-12	L23	LDR30m
**Ddx50**	0.890443558	7.87591117363222e-12	L23	LDR30m
**Mon2**	0.726122031	8.75076925912894e-12	L23	LDR30m
**Ccnl1**	0.663013589	1.55049407959638e-11	L23	LDR30m
**Tet3**	0.74956173	1.59422376413599e-11	L23	LDR30m
**Top1**	0.648040429	2.49958510303642e-11	L23	LDR30m
**Cep85l**	0.694379954	3.04999628506297e-11	L23	LDR30m
**Casc4**	0.721318054	3.9883689756646e-11	L23	LDR30m
**Pde10a**	0.850648232	4.22671729834418e-11	L23	LDR30m
**Zwint**	0.671458661	9.91658754010122e-11	L23	LDR30m
**Sec14l1**	0.645511944	1.27572011971997e-10	L23	LDR30m
**Hivep1**	0.609701062	2.70688648128904e-10	L23	LDR30m
**Plekhg5**	0.600305977	2.71773023490376e-10	L23	LDR30m
**Gm48747**	0.650666277	5.3062681842446e-10	L23	LDR30m
**Glcci1**	0.790588925	8.81287667306922e-10	L23	LDR30m
**Dlgap4**	0.695438847	3.30099961785672e-09	L23	LDR30m
**Phf21b**	1.226021279	4.9205647936043e-09	L23	LDR30m
**Pak6**	0.614704537	6.55514677500026e-09	L23	LDR30m
**Ank**	0.653418763	1.08007949574195e-08	L23	LDR30m
**Syt4**	0.651704726	1.98021547313998e-08	L23	LDR30m

**Tnfaip6**	0.768772225	2.66972568214798e-08	L23	LDR30m
**Maml3**	0.639139888	5.83019877557692e-08	L23	LDR30m
**Prkce**	0.616477201	7.89179222474704e-08	L23	LDR30m
**Ythdc1**	0.621255617	8.01666471537014e-08	L23	LDR30m
**Usp9x**	0.591221646	8.5795041790707e-08	L23	LDR30m
**Bmt2**	0.717990785	2.1311147978133e-07	L23	LDR30m
**Uba6**	0.664152293	6.35649866143248e-07	L23	LDR30m
**Asap2**	0.608579814	7.44075596124365e-07	L23	LDR30m
**Anks1**	0.699234641	7.77267454669837e-07	L23	LDR30m
**Mpc1**	0.611702388	2.18045604962411e-06	L23	LDR30m
**Arc**	0.904370344	4.25883304862995e-06	L23	LDR30m
**Snap25**	0.653608185	7.19420123542103e-06	L23	LDR30m
**Ski**	0.628609088	1.01809489009905e-05	L23	LDR30m
**Ndfip2**	1.358858395	1.47190215655549e-05	L23	LDR30m
**Fnbp1l**	0.728505742	1.88713818445163e-05	L23	LDR30m
**Rph3a**	0.611747673	0.000231701	L23	LDR30m
**Fam131a**	0.589042425	0.000249593	L23	LDR30m
**Ranbp2**	0.616623233	0.000623723	L23	LDR30m
**Spry2**	0.701240515	0.000688634	L23	LDR30m
**Gm47283**	0.597975557	0.002811757	L23	LDR30m
**Chgb**	0.914400017	0.004787711	L23	LDR30m
**Baz1a**	2.24178887	1.07018167039376e-193	L23	LDR4h
**Bdnf**	1.862893119	1.22504056230701e-111	L23	LDR4h
**Phf21b**	1.829643121	1.10839370497643e-100	L23	LDR4h
**Nptx2**	1.313615782	7.90452579908703e-92	L23	LDR4h
**Homer1**	1.715821624	5.1964161518353e-89	L23	LDR4h
**Mir670hg**	1.197195497	1.40009139150507e-74	L23	LDR4h
**Scg3**	1.216650321	1.68428362065529e-72	L23	LDR4h
**Tiparp**	1.294823678	6.10107570975456e-71	L23	LDR4h
**Sik2**	2.046816933	8.87970433560745e-70	L23	LDR4h
**Mapk4**	1.649722992	9.75211027023773e-68	L23	LDR4h
**Pcsk1**	1.594502711	2.06401166259588e-64	L23	LDR4h
**Ppme1**	1.178162541	3.88941690280185e-55	L23	LDR4h
**Grasp**	0.924568741	3.33798013659184e-53	L23	LDR4h
**Spred1**	1.314655107	6.87626902087513e-53	L23	LDR4h
**Rph3a**	1.475641514	7.93701354612661e-53	L23	LDR4h
**Ntrk2**	1.627151922	2.09527738521292e-50	L23	LDR4h
**Rheb**	1.086874404	4.82764503551713e-50	L23	LDR4h
**Numb**	0.980849325	6.74163032067552e-50	L23	LDR4h

Development • Supplementary information

**Osbpl3**	1.34622526	2.14006625165741e-49	L23	LDR4h
**Frmd6**	1.321734909	2.22950255092312e-49	L23	LDR4h
**Hsd17b12**	0.873318683	2.47016732588367e-49	L23	LDR4h
**Etv5**	1.445339428	1.20789992265891e-48	L23	LDR4h
**Per2**	0.929478681	1.08212409009982e-47	L23	LDR4h
**Nr4a3**	1.03828797	5.2031254964427e-47	L23	LDR4h
**Adgrd1**	0.812157693	1.09577013317984e-46	L23	LDR4h
**Arhgap31**	1.020507293	3.77537342352837e-46	L23	LDR4h
**Nrd1**	1.236677777	2.96253071650377e-45	L23	LDR4h
**Egr3**	1.159322245	3.26996298856387e-45	L23	LDR4h
**Fmnl1**	1.010959856	7.97672698003571e-45	L23	LDR4h
**Arid3b**	1.058354004	8.8968545001307e-45	L23	LDR4h
**Prmt8**	1.351039059	1.7547918808842e-44	L23	LDR4h
**Tmtc2**	1.053301415	3.45270911111325e-44	L23	LDR4h
**Sorcs3**	1.770114428	6.79173173955144e-44	L23	LDR4h
**Tulp4**	0.895881106	2.92353934616184e-42	L23	LDR4h
**Slc6a17**	1.168929361	7.51055711504807e-42	L23	LDR4h
**Mas1**	0.814452214	7.13099034102697e-41	L23	LDR4h
**Vmp1**	0.979241978	8.05753564112589e-41	L23	LDR4h
**Arhgef3**	1.085935504	1.04470855430166e-39	L23	LDR4h
**Sik3**	1.300649834	1.35540428818269e-38	L23	LDR4h
**Kdm6b**	0.935512779	1.91931800600524e-38	L23	LDR4h
**Scube1**	1.424163094	8.44024601840885e-38	L23	LDR4h
**Asap1**	0.843861321	8.90176507198142e-38	L23	LDR4h
**Itgav**	1.321207353	2.18054933875135e-37	L23	LDR4h
**Klhl2**	0.897841521	2.36605491300869e-37	L23	LDR4h
**Epha10**	1.078538785	3.94202498708251e-37	L23	LDR4h
**Cop1**	0.887706473	4.95598262788695e-37	L23	LDR4h
**Arap2**	1.166276766	6.28407528678263e-37	L23	LDR4h
**Ptpn9**	0.837252412	1.93988115799209e-36	L23	LDR4h
**Lrrk2**	1.125715777	2.13220848963292e-36	L23	LDR4h
**Nap1l1**	0.79115272	5.25283567130762e-36	L23	LDR4h
**Gm3764**	0.833911642	9.25326440050038e-36	L23	LDR4h
**Prim2**	1.163272747	2.90433835354675e-35	L23	LDR4h
**Osbpl8**	0.895281517	5.52257619751667e-35	L23	LDR4h
**Anxa11**	0.778440234	9.22262102211189e-35	L23	LDR4h
**Gramd1b**	1.214862726	2.60245273620867e-34	L23	LDR4h
**Jcad**	0.849983058	3.33091459112311e-34	L23	LDR4h
**Zbtb16**	1.346811104	1.12384085191231e-33	L23	LDR4h

**Slc20a2**	0.748923933	2.50210663961543e-33	L23	LDR4h
**Pfkfb3**	0.745785003	2.62444257372801e-33	L23	LDR4h
**R3hdm1**	0.882038125	3.48281372731072e-33	L23	LDR4h
**Camk1g**	0.698703972	8.17198153311701e-33	L23	LDR4h
**1700016P03Rik**	2.300152646	5.50069486801269e-32	L23	LDR4h
**Ifrd1**	0.918422886	8.30334184337315e-32	L23	LDR4h
**Nt5dc3**	0.852988431	9.87312398272432e-32	L23	LDR4h
**Mapk6**	0.772832382	2.57709259326042e-31	L23	LDR4h
**Rapgef5**	1.313489851	4.34402763804254e-31	L23	LDR4h
**Cbln2**	0.691631355	2.88053571197639e-30	L23	LDR4h
**P4ha1**	0.832616361	2.01575602804615e-29	L23	LDR4h
**Atxn10**	0.864186516	7.20851846291106e-29	L23	LDR4h
**Tsnax**	1.290424589	9.31433719788194e-29	L23	LDR4h
**Stk40**	0.929242198	1.26474392621996e-28	L23	LDR4h
**Cap2**	0.771206633	1.29749531054227e-28	L23	LDR4h
**R3hdm2**	0.815259194	3.53749106109909e-28	L23	LDR4h
**Spred2**	1.098841161	6.95972065451796e-28	L23	LDR4h
**Hectd2**	1.167763345	9.61593247236772e-28	L23	LDR4h
**Pcdh15**	1.254680959	1.63664936171776e-27	L23	LDR4h
**Egln1**	0.691354124	1.80616136025228e-27	L23	LDR4h
**Hsph1**	0.704433628	2.18623260035966e-27	L23	LDR4h
**Dot1l**	0.907213241	2.2262748036419e-27	L23	LDR4h
**Rock2**	0.898815884	2.52696672126326e-27	L23	LDR4h
**Pak6**	0.95545798	3.01829812342551e-27	L23	LDR4h
**Rgs20**	1.212869855	3.19819854738305e-27	L23	LDR4h
**Zswim6**	1.060466479	4.29219942699305e-27	L23	LDR4h
**Jdp2**	1.151610059	5.49496646982598e-27	L23	LDR4h
**Prkg2**	1.149116862	9.93491714714977e-27	L23	LDR4h
**Dgkz**	0.945255604	2.91255637148421e-26	L23	LDR4h
**Dnajc1**	0.954718831	3.01514952302873e-26	L23	LDR4h
**Tmem178**	0.81393426	4.85714771924053e-26	L23	LDR4h
**Zc3h12c**	0.656645402	6.36694760722091e-26	L23	LDR4h
**Rasgrp1**	0.759029806	7.0105052752181e-26	L23	LDR4h
**2510009E07Rik**	0.9555463	7.14761629480696e-26	L23	LDR4h
**Kdm7a**	0.975369517	2.17904725996511e-25	L23	LDR4h
**Nlk**	0.82426046	2.22540954590583e-25	L23	LDR4h
**Ddah1**	0.622789266	1.35659159588821e-24	L23	LDR4h
**Hipk3**	0.674629822	2.1545854596896e-24	L23	LDR4h
**Nrsn1**	0.609555761	2.36830756296273e-24	L23	LDR4h

Development • Supplementary information

**Brinp1**	1.13198659	4.3793917562336e-24	L23	LDR4h
**Synj2**	0.802873632	4.78851141458606e-24	L23	LDR4h
**Gm46367**	1.004993033	8.39052601043924e-24	L23	LDR4h
**Ap2b1**	0.710938198	1.71768872996018e-23	L23	LDR4h
**Zdbf2**	0.806829831	4.86549575161646e-23	L23	LDR4h
**Gm3294**	0.769070715	6.80261037712447e-23	L23	LDR4h
**Mrpl48**	0.620113268	8.11476345650553e-23	L23	LDR4h
**Pdzd2**	1.250904188	1.8801918591331e-22	L23	LDR4h
**Galnt9**	1.036370569	3.0528628916154e-22	L23	LDR4h
**Akap13**	0.874704014	4.12629228577698e-22	L23	LDR4h
**Cep85l**	0.776066306	6.6003304375758e-22	L23	LDR4h
**Slc9a5**	0.840392739	9.73195649622614e-22	L23	LDR4h
**Gnb5**	0.653844035	3.46013299840527e-21	L23	LDR4h
**Inhba**	0.774063077	2.56805287819302e-20	L23	LDR4h
**Cap1**	0.723328982	3.81475593761979e-20	L23	LDR4h
**Eml5**	1.082807688	4.0480945763764e-20	L23	LDR4h
**Hivep1**	0.708243285	4.57480754999629e-20	L23	LDR4h
**Prkar2a**	0.724498624	5.12206179355033e-20	L23	LDR4h
**Per1**	0.635507044	7.286019543928e-20	L23	LDR4h
**Tacc1**	0.65472501	1.52464053257735e-19	L23	LDR4h
**Fbxo33**	0.617058422	2.90640353798938e-19	L23	LDR4h
**Bcl2**	0.609918895	9.48936698175232e-19	L23	LDR4h
**Dnajc21**	0.674647827	1.36590620214251e-18	L23	LDR4h
**Slc25a25**	0.750707851	2.46101667804902e-18	L23	LDR4h
**Samd4**	0.673240738	3.58898695715898e-18	L23	LDR4h
**Arpc2**	0.586025311	5.19521736229248e-18	L23	LDR4h
**Myo1e**	0.602876824	9.52037220874586e-18	L23	LDR4h
**Fam171a1**	0.607814855	1.50542015916447e-17	L23	LDR4h
**Actn4**	0.623365513	1.784943868424e-17	L23	LDR4h
**Ece1**	0.918812879	2.13590764814491e-17	L23	LDR4h
**Pag1**	0.732878895	2.3713505051151e-17	L23	LDR4h
**Plxdc2**	0.823585672	3.0019053778236e-17	L23	LDR4h
**Phyhipl**	0.617001059	3.14127281311223e-17	L23	LDR4h
**Trim9**	0.730662388	5.43536798198828e-17	L23	LDR4h
**Prkar1b**	0.693442723	5.61797843393993e-17	L23	LDR4h
**Clstn3**	0.850530259	6.95046423590411e-17	L23	LDR4h
**Dnajb5**	0.640626892	7.42912743289036e-17	L23	LDR4h
**Nefm**	0.778439703	8.26649304427345e-17	L23	LDR4h
**Fndc3a**	0.69867922	1.03732276712338e-16	L23	LDR4h

Development • Supplementary information

**Ubtd2**	0.750447207	1.07351374244334e-16	L23	LDR4h
**Fosl2**	0.981310311	1.68015067831886e-16	L23	LDR4h
**Unc13a**	0.63118647	1.68072782165605e-16	L23	LDR4h
**Plcl2**	0.673039443	1.83730530605364e-16	L23	LDR4h
**Slc1a2**	0.637916553	1.9012579101791e-16	L23	LDR4h
**Stard8**	0.618398221	4.77356244694801e-16	L23	LDR4h
**Tet3**	0.990155634	7.15671624338754e-16	L23	LDR4h
**Slc2a13**	1.004985349	2.19436800138536e-15	L23	LDR4h
**Mbp**	0.804115523	6.12774679643655e-15	L23	LDR4h
**Ankrd33b**	0.776717089	7.98062241853666e-15	L23	LDR4h
**Ppm1h**	0.826022254	1.00122254678988e-14	L23	LDR4h
**Grb2**	0.815142106	1.95140713188303e-14	L23	LDR4h
**Diras2**	0.597228499	2.41557051120737e-14	L23	LDR4h
**Nptxr**	0.602181527	2.6623605030762e-14	L23	LDR4h
**Rab6b**	0.634503236	3.00061978721546e-14	L23	LDR4h
**Med14**	0.668404034	3.09844599119288e-14	L23	LDR4h
**Ephx4**	0.643347347	4.74853940170095e-14	L23	LDR4h
**Bcor**	0.647205873	7.62319963825046e-14	L23	LDR4h
**Dlg1**	0.685725776	8.88698634899292e-14	L23	LDR4h
**Fam81a**	0.595637637	9.12288171379432e-14	L23	LDR4h
**Atp1a1**	0.685872729	2.08812658530692e-13	L23	LDR4h
**Dok5**	0.643462296	8.562639575152e-13	L23	LDR4h
**Nrxn2**	0.771666476	1.03084706258551e-12	L23	LDR4h
**Jarid2**	0.765323674	1.31287417763649e-12	L23	LDR4h
**Elmo1**	2.226367564	2.31756642663062e-12	L23	LDR4h
**St8sia5**	0.70652458	2.33035242291711e-12	L23	LDR4h
**Dgki**	0.729758865	4.68754352400343e-12	L23	LDR4h
**Lhfpl3**	0.846533049	6.91296704318803e-12	L23	LDR4h
**Zmiz1**	0.855782679	1.61965707422654e-11	L23	LDR4h
**Nrn1**	0.763994621	1.62369198017919e-11	L23	LDR4h
**Nol4**	0.856072975	1.63209343779013e-11	L23	LDR4h
**Mast3**	0.675414398	2.09829060234335e-11	L23	LDR4h
**Fmr1**	0.61771542	2.35574157538439e-11	L23	LDR4h
**Dock1**	0.606339322	2.35920613176083e-11	L23	LDR4h
**Cacna1a**	0.633957849	3.6836396086542e-11	L23	LDR4h
**Ext1**	0.709672458	4.03731176662531e-11	L23	LDR4h
**Prkca**	0.647182288	7.3882337332845e-11	L23	LDR4h
**Gfod1**	0.752611341	1.32468649533925e-10	L23	LDR4h
**Nectin1**	0.688098269	1.93377816764109e-10	L23	LDR4h

Development • Supplementary information

**Pmepa1**	0.70439792	2.45056926452137e-10	L23	LDR4h
**Rnd3**	0.839671217	3.58287835899754e-10	L23	LDR4h
**Epb41l1**	0.602785967	6.08166560087003e-10	L23	LDR4h
**Prag1**	0.592167115	1.66720222144732e-09	L23	LDR4h
**Garem1**	0.6364448	2.75591113821236e-09	L23	LDR4h
**Dlgap4**	0.653024568	1.31921729209829e-08	L23	LDR4h
**Igsf9b**	0.671191082	1.85403962619048e-08	L23	LDR4h
**Ptprg**	0.748277904	2.0571517434605e-08	L23	LDR4h
**Fscn1**	0.588057615	2.3714717387461e-08	L23	LDR4h
**Megf11**	1.719991677	3.24094996752767e-08	L23	LDR4h
**Nell1**	0.6413503	6.66561588415493e-08	L23	LDR4h
**Cdh4**	0.680831769	6.7347996501036e-08	L23	LDR4h
**Plxna2**	0.588602158	7.08875351335838e-08	L23	LDR4h
**Dlgap2**	0.64678614	7.32597618498507e-08	L23	LDR4h
**Pde7b**	0.610634465	8.07786100894978e-08	L23	LDR4h
**Maml3**	1.937678739	1.18136625560977e-07	L23	LDR4h
**Nr4a1**	1.202841703	1.23331248716678e-07	L23	LDR4h
**Mical2**	0.710675805	1.25288238796203e-07	L23	LDR4h
**Ndfip2**	0.606443046	2.56668060216424e-07	L23	LDR4h
**Cdh6**	0.631569325	2.86254124542061e-07	L23	LDR4h
**Gm16351**	0.632991128	4.04165067370227e-07	L23	LDR4h
**Fstl4**	0.692762954	5.88777383839531e-07	L23	LDR4h
**Gm13684**	1.149962054	8.32846690044531e-07	L23	LDR4h
**Grin2a**	0.643505958	2.02581265885917e-06	L23	LDR4h
**Sgsm1**	1.400987734	2.03935820659647e-06	L23	LDR4h
**Gm15398**	1.917158544	3.83826148754263e-06	L23	LDR4h
**Pdzrn3**	0.689768899	3.18155911996116e-05	L23	LDR4h
**Brinp3**	0.619104153	5.46836435309416e-05	L23	LDR4h
**Cmip**	0.609853181	0.000229056	L23	LDR4h
**Disp3**	1.112747107	0.0003229	L23	LDR4h
**Ptprn**	1.0934671	0.002029582	L23	LDR4h
**Ubash3b**	1.125607376	0.00284821	L23	LDR4h
**Irs2**	0.977806099	0.003432281	L23	LDR4h
**Tmem163**	0.762937132	0.004722903	L23	LDR4h
**Gm17231**	1.281023911	0.01107546	L23	LDR4h
**Plcl1**	0.977900399	0.020530491	L23	LDR4h
**Fth1**	1.376726256	1.56001713235267e-43	L23	LDR6h
**Apoe**	0.887390753	1.68385651060054e-37	L23	LDR6h
**Ptgds**	0.586542068	1.85452693031723e-27	L23	LDR6h

Development • Supplementary information

**Cst3**	0.80086753	1.64185822843675e-23	L23	LDR6h
**mt-Co3**	1.228533812	3.15989366118924e-20	L23	LDR6h
**Ubb**	0.895903266	9.67059605061703e-20	L23	LDR6h
**Gm27032**	0.915854456	2.78830393328537e-17	L23	LDR6h
**Cox4i1**	0.763494816	4.80184696238695e-17	L23	LDR6h
**Gm12027**	0.748092921	9.05664333479777e-17	L23	LDR6h
**mt-Co1**	1.409603161	1.52229589444258e-16	L23	LDR6h
**Rap2b**	0.726175043	3.74960160926209e-16	L23	LDR6h
**H3f3b**	0.974505047	2.50619036040764e-15	L23	LDR6h
**Ppia**	0.663948655	1.75930766920124e-14	L23	LDR6h
**Pcsk1n**	0.626413624	3.54779866239512e-14	L23	LDR6h
**Eif1**	0.807803746	7.50768344430907e-14	L23	LDR6h
**Srsf7**	1.171404588	1.89509022033796e-13	L23	LDR6h
**Gm32250**	0.783178317	1.94677344319864e-12	L23	LDR6h
**Cox8a**	0.756807552	2.18839249453464e-12	L23	LDR6h
**Atxn7l3b**	0.678409077	2.28580936619175e-12	L23	LDR6h
**Cox7c**	0.94982109	1.23359314405017e-11	L23	LDR6h
**Cox6c**	0.653727756	2.87592526057528e-11	L23	LDR6h
**Ddit4l**	0.595739439	4.46957131503258e-11	L23	LDR6h
**Mdh1**	0.694938044	7.12005104442432e-11	L23	LDR6h
**Tpt1**	0.643316093	4.33755101274165e-10	L23	LDR6h
**Rpl6**	0.836616399	1.07275192417852e-09	L23	LDR6h
**Gm14211**	0.59811405	1.12701000608326e-09	L23	LDR6h
**Slc25a4**	0.673763517	2.82844638287466e-09	L23	LDR6h
**Jund**	0.616936881	1.11729516733467e-08	L23	LDR6h
**Gm15520**	0.74619469	3.41172767864488e-08	L23	LDR6h
**Tmsb4x**	0.760902619	6.75795559241514e-08	L23	LDR6h
**Rpl13**	0.623715256	8.41841067564033e-08	L23	LDR6h
**Trim35**	0.830188021	1.11003212352721e-07	L23	LDR6h
**Rps8**	0.639636467	1.27030128732246e-07	L23	LDR6h
**Tmem243**	0.731452269	1.51634158639983e-07	L23	LDR6h
**Oprm1**	0.700309937	3.50673809458987e-07	L23	LDR6h
**Nap1l5**	0.643032928	5.59440206130265e-07	L23	LDR6h
**Dact2**	0.779766088	6.22231466096974e-07	L23	LDR6h
**Rpl41**	0.654516382	7.18686892316055e-07	L23	LDR6h
**Arpp19**	0.626710395	8.60485334840547e-07	L23	LDR6h
**Cox7b**	0.606936628	1.06484948448323e-06	L23	LDR6h
**Pdlim7**	0.638970902	2.27549469121144e-06	L23	LDR6h
**Atpif1**	0.600359357	2.60379635123587e-06	L23	LDR6h

**Sema3c**	0.870617345	3.25379177523891e-06	L23	LDR6h
**Rprml**	0.62703135	5.33816778799411e-06	L23	LDR6h
**Dnaja2**	0.641563327	6.69317834565219e-06	L23	LDR6h
**6430590A07Rik**	0.635199449	8.03934621542905e-06	L23	LDR6h
**Rad51ap2**	0.800127396	8.48603370043516e-06	L23	LDR6h
**Ly6h**	0.642649531	1.15068285015888e-05	L23	LDR6h
**Atp6v0b**	0.882997004	1.25390670990127e-05	L23	LDR6h
**Mrps26**	0.610222782	4.03062545706774e-05	L23	LDR6h
**P2ry14**	0.812870321	6.45459371039196e-05	L23	LDR6h
**Gm46367**	0.808460423	7.18576004645556e-05	L23	LDR6h
**A230004M16Rik**	0.608457258	9.96128790603767e-05	L23	LDR6h
**Eef1a1**	0.703100421	0.000145079	L23	LDR6h
**Nrgn**	0.631388317	0.000237062	L23	LDR6h
**Hmgn3**	0.589465216	0.000245157	L23	LDR6h
**Malat1**	1.398749006	0.000405859	L23	LDR6h
**Crebzf**	0.667108064	0.000453886	L23	LDR6h
**5330438D12Rik**	0.755687481	0.000806091	L23	LDR6h
**Prpf4b**	0.700083035	0.002164136	L23	LDR6h
**Gm35188**	0.878305154	0.002585539	L23	LDR6h
**Hectd2os**	0.609750298	0.002604521	L23	LDR6h
**mt-Atp6**	0.842366882	0.004408363	L23	LDR6h
**Slc25a3**	0.593132716	0.006531122	L23	LDR6h
**Trpc6**	0.769389696	0.009132969	L23	LDR6h
**Prelid3a**	0.599195733	0.015109642	L23	LDR6h
**Gm43376**	0.625789332	0.046467472	L23	LDR6h
**Gm37229**	0.791095752	5.2238570941879e-34	L4	LDR2h
**Ptprn**	1.607941054	5.80006765979308e-32	L4	LDR2h
**Scg2**	0.888112497	6.72229768040005e-32	L4	LDR2h
**Rnd3**	1.027246438	2.1083351189987e-31	L4	LDR2h
**Egr3**	0.962640565	5.56292916698909e-30	L4	LDR2h
**mt-Co3**	1.508186227	6.85925021091497e-28	L4	LDR2h
**Fosl2**	0.656819299	4.40679703726251e-26	L4	LDR2h
**Grasp**	0.929570413	6.83690933623402e-26	L4	LDR2h
**Hspa8**	0.916099466	1.73838519166649e-23	L4	LDR2h
**Gm12296**	0.838840006	6.64693156430727e-23	L4	LDR2h
**mt-Co1**	1.453070751	1.2557613700843e-21	L4	LDR2h
**Ptgds**	0.760547385	2.58887274459161e-21	L4	LDR2h
**Gm34544**	1.253970147	2.89448151180543e-21	L4	LDR2h
**mt-Nd1**	1.141180962	9.1423887396054e-20	L4	LDR2h

Development • Supplementary information

**Diras2**	0.795609079	1.26384609019014e-19	L4	LDR2h
**1110008P14Rik**	0.767319652	5.05285016401926e-18	L4	LDR2h
**mt-Cytb**	1.222356001	5.90265188551686e-18	L4	LDR2h
**BC018473**	0.660093632	5.21428690595334e-17	L4	LDR2h
**C1ql3**	0.672652874	1.23456395551618e-15	L4	LDR2h
**Mfap3l**	0.782656264	1.7601358505948e-14	L4	LDR2h
**mt-Co2**	1.35273029	3.70393178002679e-14	L4	LDR2h
**Ppp1r1a**	0.824413957	9.53332310563019e-14	L4	LDR2h
**1700016P03Rik**	1.165029674	4.88160919264278e-13	L4	LDR2h
**Inhba**	0.590156199	3.08794440284811e-12	L4	LDR2h
**Npas4**	0.878784869	3.66805690717429e-12	L4	LDR2h
**Pdp1**	0.680020695	7.97689093793273e-12	L4	LDR2h
**Dalrd3**	0.692580178	8.99411548783781e-12	L4	LDR2h
**Gm35188**	1.071113037	2.60006997677541e-11	L4	LDR2h
**Jund**	0.597765727	3.38331656239514e-11	L4	LDR2h
**Cox7c**	0.846853822	3.94696999426183e-11	L4	LDR2h
**Sh2d5**	0.748667171	4.10666922485179e-11	L4	LDR2h
**Dhps**	0.621270656	5.92724687860102e-11	L4	LDR2h
**Pabpc4**	0.781695623	2.22318037744007e-10	L4	LDR2h
**Ociad2**	0.654078443	2.68521721943357e-10	L4	LDR2h
**Cap1**	0.638657394	4.4190698502988e-10	L4	LDR2h
**Baz1a**	0.87233868	1.63985978894665e-09	L4	LDR2h
**Hectd2os**	0.670634795	1.80326539848782e-09	L4	LDR2h
**mt-Atp6**	1.249968877	1.90744885836985e-09	L4	LDR2h
**H3f3b**	0.728758434	3.07071474510709e-09	L4	LDR2h
**Tpt1**	0.7613137	3.87529549441352e-09	L4	LDR2h
**Fau**	0.590183294	5.99938668832298e-09	L4	LDR2h
**Gm26652**	0.592024691	7.34221716963118e-09	L4	LDR2h
**Ppme1**	1.013734053	1.03047332835033e-08	L4	LDR2h
**Klf10**	0.665717183	1.1797672070229e-08	L4	LDR2h
**Purb**	0.653942261	1.27382879698823e-08	L4	LDR2h
**Scn1b**	0.631080063	1.55621558558018e-08	L4	LDR2h
**Nptx2**	0.90740092	2.23519640320086e-08	L4	LDR2h
**Cx3cl1**	0.686430701	2.6820070758268e-08	L4	LDR2h
**Hsp90ab1**	0.59620802	9.14044165962488e-08	L4	LDR2h
**Nrn1**	0.907844768	1.02061063766127e-07	L4	LDR2h
**Slc25a4**	0.642229165	2.09646061838099e-07	L4	LDR2h
**Eif1**	0.741093871	2.34071710328127e-07	L4	LDR2h
**Rplp1**	0.614701932	3.01640810314278e-07	L4	LDR2h

**Ccsap**	0.665130123	3.80649456662899e-07	L4	LDR2h
**Sema3c**	1.637576082	6.80739537844534e-07	L4	LDR2h
**Aldoa**	0.596942137	1.11720954721573e-06	L4	LDR2h
**Nr4a1**	0.625300712	1.29031449022972e-06	L4	LDR2h
**Rps8**	0.603569837	1.48300751196926e-06	L4	LDR2h
**Rpl6**	0.768777824	2.48121783842174e-06	L4	LDR2h
**Calm1**	0.667689006	2.86887155938255e-06	L4	LDR2h
**Camk2n1**	0.652692538	5.28919972369159e-06	L4	LDR2h
**Atp6v0b**	0.971502211	7.58033052042884e-06	L4	LDR2h
**Gm14636**	0.658104974	1.42638037452379e-05	L4	LDR2h
**Cox6c**	0.604141323	3.59240715772202e-05	L4	LDR2h
**Slc25a3**	0.716311392	3.92682363192879e-05	L4	LDR2h
**Nrsn1**	0.727492735	5.05698370922e-05	L4	LDR2h
**Gm15398**	1.073452466	6.44993959133414e-05	L4	LDR2h
**Rpl41**	0.630628615	7.04633526705197e-05	L4	LDR2h
**1700085D07Rik**	0.716494592	9.88295779867217e-05	L4	LDR2h
**Rpl13**	0.60931309	0.000117414	L4	LDR2h
**Gm15520**	0.738654463	0.000134762	L4	LDR2h
**mt-Nd4**	0.801744731	0.000150594	L4	LDR2h
**Calm2**	0.626499123	0.000174235	L4	LDR2h
**Rapgef4os1**	0.775702309	0.000313969	L4	LDR2h
**Ctnna3**	0.952147418	0.000385471	L4	LDR2h
**Actb**	0.593474212	0.000443715	L4	LDR2h
**Apoe**	1.140967604	0.000509774	L4	LDR2h
**Gm17231**	0.644649078	0.000849992	L4	LDR2h
**Kcnc2**	0.717669696	0.000914122	L4	LDR2h
**Gm16351**	0.73752333	0.000937885	L4	LDR2h
**Cox4i1**	0.617732623	0.000967548	L4	LDR2h
**Dot1l**	0.727411145	0.001273447	L4	LDR2h
**Wls**	0.661289589	0.001370997	L4	LDR2h
**Megf11**	1.26164258	0.001563057	L4	LDR2h
**Gm42439**	0.759399348	0.001889584	L4	LDR2h
**Nap1l5**	0.628033152	0.002750324	L4	LDR2h
**Arhgap5**	0.613712403	0.003543672	L4	LDR2h
**Cst3**	0.870841224	0.005525961	L4	LDR2h
**Igsf9b**	0.813394318	0.005638146	L4	LDR2h
**Nrgn**	0.60950135	0.013070263	L4	LDR2h
**Gm11867**	0.856445212	0.017651591	L4	LDR2h
**4930415C11Rik**	1.558729302	0.039980782	L4	LDR2h

**Egr3**	2.513875107	2.22237333874281e-209	L4	LDR30m
**Homer1**	2.58004551	8.67536036673228e-134	L4	LDR30m
**Arl5b**	1.513515576	2.31467975749944e-97	L4	LDR30m
**Frmd6**	1.903793131	6.48439533906298e-96	L4	LDR30m
**Sik2**	2.04805523	2.43361265719473e-76	L4	LDR30m
**Phf21b**	1.449880646	5.47693869578608e-73	L4	LDR30m
**Stk40**	1.273455098	1.13713678588506e-67	L4	LDR30m
**Fosb**	1.143143529	2.01660690772555e-61	L4	LDR30m
**Zdbf2**	1.635368278	8.88640822125628e-61	L4	LDR30m
**Prag1**	1.306579406	2.66939183296025e-60	L4	LDR30m
**Plcxd2**	1.649326399	1.1388502868011e-58	L4	LDR30m
**Irs2**	1.390518654	1.47127688563274e-58	L4	LDR30m
**Med14**	1.316296342	5.3717826059226e-58	L4	LDR30m
**Grasp**	1.153085016	2.2270522239003e-57	L4	LDR30m
**Ankrd33b**	1.303054673	1.28282562338257e-55	L4	LDR30m
**Adora1**	1.161876299	2.18947690390076e-54	L4	LDR30m
**Nr4a1**	1.368777745	1.80367568474633e-53	L4	LDR30m
**Abhd2**	1.343280408	9.02174748502046e-53	L4	LDR30m
**Gm17231**	1.243970217	4.57822494382304e-51	L4	LDR30m
**Kdm7a**	1.105587386	2.32198909551424e-50	L4	LDR30m
**Arid3b**	1.006451852	2.73156272668785e-49	L4	LDR30m
**Rheb**	1.467644288	6.20071553033683e-48	L4	LDR30m
**Nrd1**	1.529525979	1.62282345562755e-47	L4	LDR30m
**Mbp**	1.393764228	4.863338781142e-47	L4	LDR30m
**Etv5**	1.444021424	5.07955803927823e-47	L4	LDR30m
**Ube2ql1**	1.124650972	1.49203147566665e-44	L4	LDR30m
**1600020E01Rik**	1.047609675	4.29460433373119e-44	L4	LDR30m
**Cpeb3**	1.075667291	1.28260585035581e-43	L4	LDR30m
**Skil**	1.010613162	3.22286073812832e-42	L4	LDR30m
**Dusp14**	1.02312171	3.29219515215076e-42	L4	LDR30m
**Arhgef3**	1.535257748	6.97865976451468e-42	L4	LDR30m
**Kdm6b**	0.929081003	1.66856755641074e-41	L4	LDR30m
**Per1**	1.33929758	3.84180480598136e-41	L4	LDR30m
**Hsph1**	1.023796119	7.82036251446528e-41	L4	LDR30m
**Slc25a25**	1.36639803	1.28482088500165e-40	L4	LDR30m
**Rcc2**	0.931557978	1.44859083485259e-40	L4	LDR30m
**Ptprn**	1.37063511	9.10533225650426e-40	L4	LDR30m
**Mir670hg**	1.152321312	9.83129953937731e-40	L4	LDR30m
**Ak4**	0.887036746	1.30762783472444e-39	L4	LDR30m

Development • Supplementary information

**Myh9**	1.164817033	1.50269473538506e-38	L4	LDR30m
**Mapk4**	1.017276408	3.28835097181026e-38	L4	LDR30m
**Zswim6**	1.174533429	2.30805535475794e-37	L4	LDR30m
**Btaf1**	0.965132247	4.38045552474606e-35	L4	LDR30m
**Nptx2**	0.748273218	3.5338824173751e-34	L4	LDR30m
**Ctps**	0.855909335	5.63329934404893e-34	L4	LDR30m
**Nr4a2**	1.856992292	8.47272704405756e-34	L4	LDR30m
**Baiap2**	1.368129131	1.5942320434411e-33	L4	LDR30m
**Nectin1**	1.213461569	6.04358772630907e-33	L4	LDR30m
**Synj2**	0.965462126	9.83655104642946e-33	L4	LDR30m
**Slc6a17**	0.903123595	3.07701094230019e-32	L4	LDR30m
**Arpc2**	0.908875433	2.21805210205665e-31	L4	LDR30m
**Spag9**	0.853180647	3.92836788746869e-31	L4	LDR30m
**Mxi1**	1.038015713	2.48018634151281e-30	L4	LDR30m
**Tet3**	1.152255665	2.82419865824521e-30	L4	LDR30m
**Ece1**	1.200324494	3.69283579079547e-30	L4	LDR30m
**Galnt9**	1.190817654	5.45233720201029e-30	L4	LDR30m
**Clstn3**	1.009682342	6.59003894514882e-30	L4	LDR30m
**Cbarp**	0.992924021	1.55863304651344e-29	L4	LDR30m
**Fosl2**	1.442784751	3.49063559911696e-29	L4	LDR30m
**Trib1**	0.62815054	5.35977308747839e-29	L4	LDR30m
**R3hdm2**	0.875812114	6.33152375877178e-29	L4	LDR30m
**Slc20a2**	0.96128072	1.20914060670348e-28	L4	LDR30m
**Gpt2**	0.811933593	1.29062362130662e-28	L4	LDR30m
**Sidt1**	0.883868757	3.22120092098188e-28	L4	LDR30m
**1700016P03Rik**	2.806753125	3.65410342812829e-28	L4	LDR30m
**Jdp2**	1.262046629	4.65514137850539e-28	L4	LDR30m
**Smap2**	0.857263696	6.8497340619153e-28	L4	LDR30m
**Numbl**	0.787241776	1.35248762429597e-27	L4	LDR30m
**Midn**	0.760984612	2.5009636795007e-27	L4	LDR30m
**Bicdl1**	0.810843608	5.55921134664513e-27	L4	LDR30m
**Ubtd2**	1.131024875	9.09195871013145e-27	L4	LDR30m
**Camk1g**	0.719928028	2.83504325858311e-26	L4	LDR30m
**Zbtb16**	1.326307825	3.08114741887457e-26	L4	LDR30m
**Mn1**	0.97383428	6.53510070976511e-26	L4	LDR30m
**Per2**	0.946102895	1.2866283453671e-25	L4	LDR30m
**Ctnnd1**	0.821824507	4.61370016177604e-25	L4	LDR30m
**Acsl4**	0.865475682	6.23187552817414e-25	L4	LDR30m
**Nab2**	0.620364328	7.25103344881141e-25	L4	LDR30m

Development • Supplementary information

**Tulp4**	0.80076528	1.63770260159133e-24	L4	LDR30m
**Nap1l1**	0.812878649	4.04981125957105e-24	L4	LDR30m
**Gnai3**	0.74494058	7.41712429770331e-24	L4	LDR30m
**Ago3**	0.81033408	1.24690554249745e-23	L4	LDR30m
**Rims4**	0.983971132	1.98455469471976e-23	L4	LDR30m
**Kif5c**	0.768727412	2.2280057514692e-23	L4	LDR30m
**Rgs7bp**	0.699288304	3.97801203696256e-23	L4	LDR30m
**Tiparp**	2.033886915	1.29432332554787e-22	L4	LDR30m
**Rock2**	1.03656475	1.4445897718163e-22	L4	LDR30m
**Gm28294**	0.663187936	1.71030156604853e-22	L4	LDR30m
**Hspa4**	0.786957379	2.52377239629736e-22	L4	LDR30m
**Smg7**	0.763617655	3.05765373063303e-22	L4	LDR30m
**Dusp5**	0.596366284	3.19996906137291e-22	L4	LDR30m
**Ank**	0.991407022	5.09163553010516e-22	L4	LDR30m
**Gfod1**	1.17851489	1.00806018764166e-21	L4	LDR30m
**Hmgcr**	0.892675374	1.6356956634791e-21	L4	LDR30m
**Plekhg5**	0.788060068	2.51746710556421e-21	L4	LDR30m
**Spred2**	0.870265943	3.00813207158018e-21	L4	LDR30m
**Pip5k1a**	0.696511219	3.25411903630856e-21	L4	LDR30m
**Tpm3**	0.734289592	4.34774639772589e-21	L4	LDR30m
**4931406P16Rik**	0.711486286	4.95182775175333e-21	L4	LDR30m
**Slc7a1**	0.792973183	5.44594799527522e-21	L4	LDR30m
**Sik3**	0.993405921	8.03637851765158e-21	L4	LDR30m
**Hsd17b12**	0.670754427	8.94233804149172e-21	L4	LDR30m
**Efhd2**	0.759535894	1.01751286009315e-20	L4	LDR30m
**Fbl**	0.690641701	1.23495270094467e-20	L4	LDR30m
**Tanc1**	0.841396115	1.57428112054976e-20	L4	LDR30m
**Cry2**	0.958825073	1.82889719980856e-20	L4	LDR30m
**Lonp2**	0.933621995	3.44641531054106e-20	L4	LDR30m
**Pitpna**	0.707906906	4.04025644060065e-20	L4	LDR30m
**Sntb2**	0.703190237	4.20381417532947e-20	L4	LDR30m
**Ifrd1**	0.839885521	5.32969856632269e-20	L4	LDR30m
**Nudt4**	1.021429454	1.13994929850117e-19	L4	LDR30m
**P4ha1**	0.846936268	1.15647179762541e-19	L4	LDR30m
**Hdac5**	0.814011982	1.29256747519615e-19	L4	LDR30m
**Csdc2**	0.65202944	1.54974778309452e-19	L4	LDR30m
**Pip4k2c**	0.699408824	4.09669952465603e-19	L4	LDR30m
**Mapk6**	0.706439808	5.85984924734865e-19	L4	LDR30m
**Mast3**	0.789946395	6.87500949874858e-19	L4	LDR30m

**Clip2**	1.038453654	9.58047569881277e-19	L4	LDR30m
**Elovl5**	0.621420838	9.60259688700966e-19	L4	LDR30m
**Txnrd1**	0.645265813	1.15770578523163e-18	L4	LDR30m
**Npas4**	0.829190059	1.78782149479853e-18	L4	LDR30m
**Mest**	0.719183048	1.96590599582387e-18	L4	LDR30m
**Ivns1abp**	0.658499814	2.45454530045932e-18	L4	LDR30m
**Coq10b**	0.948022527	2.5640418270984e-18	L4	LDR30m
**Fam81a**	0.749385053	6.1112991587165e-18	L4	LDR30m
**Cap1**	0.638525146	8.91475373319744e-18	L4	LDR30m
**Cpeb4**	0.905820287	1.25075101889483e-17	L4	LDR30m
**Dnajc1**	1.140459621	1.66249485878396e-17	L4	LDR30m
**Stx1b**	0.744416114	2.040180678589e-17	L4	LDR30m
**Basp1**	0.697092246	2.30142730898186e-17	L4	LDR30m
**Cystm1**	0.655887479	3.00247318790619e-17	L4	LDR30m
**Dnajb5**	0.769273534	3.87843701898054e-17	L4	LDR30m
**Tacc1**	0.69906888	5.04549740617789e-17	L4	LDR30m
**Tm9sf3**	0.665408502	5.92454313484537e-17	L4	LDR30m
**Arih1**	0.729457089	8.16895571369431e-17	L4	LDR30m
**Ap2b1**	0.677603604	1.10495050101319e-16	L4	LDR30m
**Rnf217**	0.729608695	1.46760492744035e-16	L4	LDR30m
**Spred1**	0.930402608	1.50212481987405e-16	L4	LDR30m
**Cabp1**	0.797795841	2.45903077067221e-16	L4	LDR30m
**Tgfbr1**	0.596074113	2.88213986551885e-16	L4	LDR30m
**Ern1**	0.636259539	3.05869107736148e-16	L4	LDR30m
**Mrpl48**	0.627880198	8.74811943712175e-16	L4	LDR30m
**Ppp2ca**	0.622982235	1.444380812109e-15	L4	LDR30m
**Ccnl1**	0.616645988	2.12074381560504e-15	L4	LDR30m
**Hivep1**	0.775969756	2.70013866553994e-15	L4	LDR30m
**Zbtb11**	0.628105297	3.28459054590351e-15	L4	LDR30m
**Eprs**	0.604415682	3.41668946923124e-15	L4	LDR30m
**Scg3**	0.59408747	3.81719260061334e-15	L4	LDR30m
**Mgrn1**	0.630242562	5.45625807591897e-15	L4	LDR30m
**Ppard**	0.690715716	5.89366604163448e-15	L4	LDR30m
**Lncpint**	0.879788996	6.20219635517634e-15	L4	LDR30m
**Lonrf1**	0.619426988	7.95233519219368e-15	L4	LDR30m
**Fzd3**	0.646112369	9.03349934520013e-15	L4	LDR30m
**Arih2**	0.651783636	1.0003806211003e-14	L4	LDR30m
**Usp28**	0.674276255	1.3377866339429e-14	L4	LDR30m
**Ttpal**	0.629323401	1.47561645465172e-14	L4	LDR30m

**St8sia5**	0.988126298	4.5158716694729e-14	L4	LDR30m
**Syt13**	0.644151018	5.69615458287487e-14	L4	LDR30m
**Prickle1**	0.590013294	6.37465215335661e-14	L4	LDR30m
**Lemd3**	0.625110322	6.43054784415627e-14	L4	LDR30m
**Ago2**	0.734738074	6.85465314537102e-14	L4	LDR30m
**Anxa11**	0.636233043	7.36833501437052e-14	L4	LDR30m
**Crem**	0.635237581	1.02982716382888e-13	L4	LDR30m
**Csnk1a1**	0.629857935	1.16139223043427e-13	L4	LDR30m
**Spock2**	0.629622162	1.50222070341761e-13	L4	LDR30m
**Zfp948**	0.592445967	1.73771099811664e-13	L4	LDR30m
**Pik3r3**	0.58675348	3.06983819058776e-13	L4	LDR30m
**Ndel1**	0.60037189	3.21595919543237e-13	L4	LDR30m
**Dennd5b**	0.693978676	4.63748638217298e-13	L4	LDR30m
**Atp6v0d1**	0.594328539	5.35428418971019e-13	L4	LDR30m
**Tbc1d1**	0.865865589	6.37168043675197e-13	L4	LDR30m
**Atp2b1**	0.598630259	1.02418116417114e-12	L4	LDR30m
**Coro1c**	0.590711475	1.23445703624252e-12	L4	LDR30m
**Kcnk12**	0.611622434	1.40009791194922e-12	L4	LDR30m
**Kpna1**	0.630975749	1.55982050410186e-12	L4	LDR30m
**Mon2**	0.587025083	1.6533579691408e-12	L4	LDR30m
**Fscn1**	0.701262921	1.67096590261524e-12	L4	LDR30m
**Rab6b**	0.874772295	1.85029921657453e-12	L4	LDR30m
**Pak1**	0.624860519	3.99534944375768e-12	L4	LDR30m
**Bcor**	0.772792721	6.99546264935646e-12	L4	LDR30m
**Pde4a**	0.747797032	7.21116079365334e-12	L4	LDR30m
**Gm47423**	1.943459554	1.18627456845243e-11	L4	LDR30m
**Eml4**	0.594631071	1.66398012152591e-11	L4	LDR30m
**Maml3**	0.88641351	2.63475180838019e-11	L4	LDR30m
**Trim9**	0.622764246	2.72307242476494e-11	L4	LDR30m
**Slc25a3**	0.76892621	3.13499412585699e-11	L4	LDR30m
**Foxo3**	0.671509016	3.29448718957314e-11	L4	LDR30m
**Chgb**	0.630866432	3.32467973392673e-11	L4	LDR30m
**Cmip**	0.912792394	3.41714308114717e-11	L4	LDR30m
**Trak1**	0.798089113	4.36183265136516e-11	L4	LDR30m
**Myo1e**	0.754269779	4.7929377793328e-11	L4	LDR30m
**Osbpl8**	0.599150457	8.07630642420549e-11	L4	LDR30m
**Rph3a**	0.806865316	1.64326942662706e-10	L4	LDR30m
**Fam13c**	0.670588757	1.96169049292167e-10	L4	LDR30m
**Dlgap4**	0.818283979	2.1191250116226e-10	L4	LDR30m

Development • Supplementary information

**Agap3**	0.665131755	2.24459424165372e-10	L4	LDR30m
**Cx3cl1**	0.697454959	2.37894406524923e-10	L4	LDR30m
**Arhgef7**	0.608843908	2.53930838803807e-10	L4	LDR30m
**Kmt2a**	0.671695388	3.10399649711905e-10	L4	LDR30m
**Klf9**	0.627454977	3.23709051039963e-10	L4	LDR30m
**Gramd1b**	0.663678331	4.22719221307826e-10	L4	LDR30m
**Dleu2**	0.675648506	5.55796394960041e-10	L4	LDR30m
**Hnrnpll**	0.749938245	6.72973700658411e-10	L4	LDR30m
**Atp1a1**	0.590933931	8.85578211544267e-10	L4	LDR30m
**Rims3**	0.723245504	1.09702922951903e-09	L4	LDR30m
**Anks1**	0.767083404	1.70747911508082e-09	L4	LDR30m
**Hspa8**	0.642635309	1.99120084153317e-09	L4	LDR30m
**Dip2a**	0.655400541	2.38773268665034e-09	L4	LDR30m
**Csnk1d**	0.710935281	3.51717121632081e-09	L4	LDR30m
**Ppme1**	0.601044831	5.39042797012235e-09	L4	LDR30m
**Tead1**	0.603444447	2.02301254756181e-08	L4	LDR30m
**Mpp7**	0.615285551	2.48410288242849e-08	L4	LDR30m
**Usp36**	0.606489458	2.55914026386931e-08	L4	LDR30m
**Psd3**	0.598596977	2.64990049460557e-08	L4	LDR30m
**Pcsk1**	2.260736137	2.66379790047529e-08	L4	LDR30m
**Osbpl3**	0.597090306	2.7807712672776e-08	L4	LDR30m
**Hivep2**	0.586044056	2.78266400176535e-08	L4	LDR30m
**Epb41l1**	0.614485441	3.1354413766148e-08	L4	LDR30m
**Sik1**	0.884109267	4.01827103229532e-08	L4	LDR30m
**Mpc1**	0.608724505	4.80613414065051e-08	L4	LDR30m
**Sipa1l2**	0.626184776	6.69213638453537e-08	L4	LDR30m
**Pkia**	0.604923721	7.13573692935053e-08	L4	LDR30m
**Ndfip2**	0.771224225	9.7086006664772e-08	L4	LDR30m
**Ddx50**	0.690565651	1.14372271448823e-07	L4	LDR30m
**Mir9-3hg**	0.590935147	1.40884017582262e-07	L4	LDR30m
**Sptbn2**	0.666941842	1.41440147692144e-07	L4	LDR30m
**Rab6a**	0.671918273	1.49348132004017e-07	L4	LDR30m
**Phyhipl**	0.629822296	1.85451961456528e-07	L4	LDR30m
**Nrn1**	0.633875328	1.23231078587353e-06	L4	LDR30m
**Rassf3**	0.677526185	1.78122286278125e-06	L4	LDR30m
**1700110K17Rik**	0.60021506	1.8147013908736e-06	L4	LDR30m
**Prkce**	0.691453904	1.82357144808131e-06	L4	LDR30m
**Prmt8**	0.597380491	2.86187921025789e-06	L4	LDR30m
**Lrrk2**	0.736252136	3.45834088190478e-06	L4	LDR30m

Development • Supplementary information

**Nr4a3**	2.270313486	4.9116706593225e-06	L4	LDR30m
**Ski**	0.727536035	8.96152235792906e-06	L4	LDR30m
**Pmepa1**	1.173386346	1.72259317441602e-05	L4	LDR30m
**Bdnf**	1.447130201	0.00054945	L4	LDR30m
**Grm4**	0.919248822	0.026300743	L4	LDR30m
**Mapk4**	1.476983931	7.91242543625161e-73	L4	LDR4h
**Homer1**	1.363168494	4.19524606885849e-65	L4	LDR4h
**Phf21b**	1.218261014	1.04337203187431e-58	L4	LDR4h
**Mir670hg**	1.348786057	1.40783459888751e-53	L4	LDR4h
**Baz1a**	1.032898559	1.14464302639632e-47	L4	LDR4h
**Nptx2**	1.019924539	2.96754280390665e-47	L4	LDR4h
**Egr3**	1.264241706	1.38092925897802e-46	L4	LDR4h
**Sik2**	1.635637663	1.04705201013453e-38	L4	LDR4h
**Slc9a5**	1.041105478	2.52959840818546e-36	L4	LDR4h
**Anxa11**	0.99168685	2.96825470782765e-36	L4	LDR4h
**Grasp**	0.906500649	7.19403215676028e-35	L4	LDR4h
**Rnd3**	1.011995255	1.37144769008343e-28	L4	LDR4h
**Prag1**	0.890211665	2.14820608587646e-28	L4	LDR4h
**Zbtb16**	1.05512215	2.23393941629979e-28	L4	LDR4h
**Slc6a17**	0.789010065	2.57863715585243e-28	L4	LDR4h
**Arid3b**	0.757796305	1.11397834354381e-27	L4	LDR4h
**Spred2**	0.969440697	2.15212780240046e-27	L4	LDR4h
**Spred1**	0.945315708	1.00832336559047e-26	L4	LDR4h
**Scube1**	0.965792375	2.24273209237204e-26	L4	LDR4h
**Igsf3**	0.722540031	9.89571373562399e-26	L4	LDR4h
**Ptprn**	1.298719769	2.77624032993905e-25	L4	LDR4h
**Fmnl1**	0.805389517	4.02742997909695e-25	L4	LDR4h
**Lhfpl3**	0.886661353	1.28927099711763e-24	L4	LDR4h
**Dot1l**	0.828334098	1.41403964190242e-24	L4	LDR4h
**Inf2**	0.760032693	2.43713086918785e-24	L4	LDR4h
**Ccdc134**	0.666887843	2.29240666169463e-23	L4	LDR4h
**Pfkfb3**	0.662773216	3.54193449542223e-23	L4	LDR4h
**Frmd6**	1.033747724	3.74311355548285e-23	L4	LDR4h
**Trpc3**	0.731771145	2.48546797499252e-22	L4	LDR4h
**Mamld1**	0.760499446	5.25152672642773e-22	L4	LDR4h
**Epha10**	0.874894871	9.10838438355828e-22	L4	LDR4h
**Klhl2**	0.796614331	2.1375610357412e-21	L4	LDR4h
**Fam13c**	0.930812356	3.04578106482279e-21	L4	LDR4h
**Ankrd33b**	0.759943226	3.87893520647319e-19	L4	LDR4h

Development • Supplementary information

**1600020E01Rik**	0.64621465	5.02444875318345e-19	L4	LDR4h
**Nrxn2**	1.053832218	6.74025958904033e-19	L4	LDR4h
**Hsph1**	0.659224255	6.7684323867412e-19	L4	LDR4h
**Ppme1**	0.881322054	8.97411400061583e-19	L4	LDR4h
**Tanc1**	0.770566661	1.68730744112859e-18	L4	LDR4h
**Galnt9**	0.812001556	1.86467829240501e-18	L4	LDR4h
**Ddah1**	0.651961737	2.78629792926631e-18	L4	LDR4h
**Nrd1**	0.86412651	2.84210844451974e-18	L4	LDR4h
**Kdm6b**	0.619883272	1.02818034999583e-17	L4	LDR4h
**Gdpd5**	0.728354568	2.69825420679977e-17	L4	LDR4h
**Antxr2**	0.814452862	9.9107019146365e-16	L4	LDR4h
**Ppm1h**	0.9561785	7.70316654307262e-15	L4	LDR4h
**Prmt8**	0.736215274	1.44617295248118e-14	L4	LDR4h
**Etv5**	0.878024699	2.46456109847808e-14	L4	LDR4h
**Rock2**	0.805507297	2.70374654655576e-14	L4	LDR4h
**Sorcs3**	2.155886044	2.93212415516469e-14	L4	LDR4h
**Abhd2**	0.651167717	3.22765229057195e-14	L4	LDR4h
**Igfn1**	0.590688335	3.36131101091467e-14	L4	LDR4h
**Rph3a**	0.716117668	4.15960416279882e-14	L4	LDR4h
**Xylt1**	0.723550085	7.16791176659461e-14	L4	LDR4h
**Gm32647**	0.69191416	8.30274625852216e-14	L4	LDR4h
**1700016P03Rik**	1.738884542	1.05928115421513e-13	L4	LDR4h
**Tet3**	0.762588263	1.27761589888297e-13	L4	LDR4h
**Zswim6**	0.74922023	1.30116684630235e-13	L4	LDR4h
**Osbpl3**	0.688549236	8.04463968831127e-13	L4	LDR4h
**Zdbf2**	0.608705468	1.02065659222769e-12	L4	LDR4h
**Cdyl2**	0.901436238	1.28100966193623e-12	L4	LDR4h
**Bcor**	0.696656217	1.40756553223851e-12	L4	LDR4h
**Pde4a**	0.61731919	1.95682728359866e-12	L4	LDR4h
**Adgrl3**	0.778496504	2.91965551674462e-12	L4	LDR4h
**Gfod1**	0.905186887	4.6471927349391e-12	L4	LDR4h
**2510009E07Rik**	0.659169935	4.73620595996842e-12	L4	LDR4h
**Plxna4**	0.597490537	5.03192953192929e-12	L4	LDR4h
**Kcnc2**	0.767938423	6.23907705701652e-12	L4	LDR4h
**Mir9-3hg**	0.743529066	9.53851322940403e-12	L4	LDR4h
**Hivep1**	0.654609939	1.49311830877438e-11	L4	LDR4h
**Jdp2**	0.65890466	1.98029034297504e-11	L4	LDR4h
**Tmem178**	0.715740408	2.55887297656884e-11	L4	LDR4h
**A730060N03Rik**	0.639401311	3.69018988641397e-11	L4	LDR4h

Development • Supplementary information

**Gm46367**	0.724146446	5.12832023962435e-11	L4	LDR4h
**Igsf9b**	0.846837804	5.50707223035631e-11	L4	LDR4h
**Pcdh15**	0.830582868	6.01280240707228e-11	L4	LDR4h
**Iqsec3**	0.592531559	2.53563546601917e-10	L4	LDR4h
**Tmtc2**	0.796379722	4.19889838641866e-10	L4	LDR4h
**Disp3**	0.684516291	5.35320193425428e-10	L4	LDR4h
**Sik3**	0.642589615	5.84140063706403e-10	L4	LDR4h
**Ece1**	0.682211783	6.42566145683959e-10	L4	LDR4h
**Mbp**	0.640468165	1.26666016040857e-09	L4	LDR4h
**Plekhh2**	0.658224824	1.43525146490975e-09	L4	LDR4h
**Galnt7**	0.612004418	2.67413903032174e-09	L4	LDR4h
**Hectd2**	0.715602765	2.83089055820125e-09	L4	LDR4h
**Megf11**	1.68726757	6.4725249763106e-09	L4	LDR4h
**Eml5**	0.716626685	7.95227772053604e-09	L4	LDR4h
**Cdh22**	0.629790102	1.44661017610208e-08	L4	LDR4h
**Nrp1**	0.635216059	2.17945966815098e-08	L4	LDR4h
**Nectin1**	0.625313004	2.42887104594807e-08	L4	LDR4h
**Mast3**	0.61758661	3.91569009699931e-08	L4	LDR4h
**Tsnax**	0.636349248	6.32444041318659e-08	L4	LDR4h
**Gsg1l**	0.598789974	8.34837799901992e-08	L4	LDR4h
**Gm16351**	0.687794029	1.26657401715069e-07	L4	LDR4h
**Ttll11**	0.607792087	1.64090618645745e-07	L4	LDR4h
**Ubtd2**	0.608090829	3.65459418356581e-07	L4	LDR4h
**Prkar1b**	0.592137109	3.84688135899227e-07	L4	LDR4h
**Pmepa1**	0.59107207	5.12201421721111e-07	L4	LDR4h
**Prkg2**	1.089627143	6.86682683001131e-07	L4	LDR4h
**Arhgef3**	0.655260681	8.00792021660671e-07	L4	LDR4h
**Tiparp**	1.049000356	1.22859553344888e-06	L4	LDR4h
**Clmp**	0.599507338	1.54232253960326e-06	L4	LDR4h
**Plcxd2**	0.599521708	1.85110756127127e-06	L4	LDR4h
**Rheb**	0.617557104	2.94502496320267e-06	L4	LDR4h
**9530059O14Rik**	0.618460712	3.06100614947389e-06	L4	LDR4h
**Enox1**	0.620868351	3.57053429095005e-06	L4	LDR4h
**Galnt18**	0.590540909	5.12293018287764e-06	L4	LDR4h
**Sgsm1**	1.44972525	5.14272320041924e-06	L4	LDR4h
**Nr4a1**	0.656112388	7.76434222053864e-06	L4	LDR4h
**Fstl4**	0.645188588	9.21877585773082e-06	L4	LDR4h
**Dnajc1**	0.660257292	2.81633568693155e-05	L4	LDR4h
**Pdzd2**	1.279922435	8.28233976883192e-05	L4	LDR4h

Development • Supplementary information

**Nrn1**	0.589116654	8.61050663039567e-05	L4	LDR4h
**Cacng2**	0.622538083	0.000123735	L4	LDR4h
**Akap13**	1.077139661	0.000326945	L4	LDR4h
**Gm15398**	1.253904814	0.000362696	L4	LDR4h
**Smad3**	0.602516294	0.00062311	L4	LDR4h
**Arhgap31**	0.74054556	0.000961314	L4	LDR4h
**Zmiz1**	1.03056784	0.000986288	L4	LDR4h
**Mical2**	0.617476387	0.001761846	L4	LDR4h
**Gm17231**	0.926528239	0.004559218	L4	LDR4h
**Fosl2**	0.612200195	0.014409231	L4	LDR4h
**Bdnf**	1.222830805	0.045416388	L4	LDR4h
**Ntrk3**	1.002386197	0.047395864	L4	LDR4h
**Apoe**	0.942638826	1.731247347325e-41	L4	LDR6h
**Fth1**	1.340555462	1.95797373949095e-39	L4	LDR6h
**mt-Co3**	1.388632335	1.15932334704344e-32	L4	LDR6h
**Ptgds**	0.744955943	8.25997213445603e-32	L4	LDR6h
**Cst3**	0.858827292	3.88012976016248e-29	L4	LDR6h
**Sema3c**	0.95856625	4.85609984821317e-27	L4	LDR6h
**mt-Co1**	1.470020906	5.47283065696318e-24	L4	LDR6h
**H3f3b**	1.086212325	1.90147267770196e-22	L4	LDR6h
**Srsf7**	1.14938922	6.96865958875701e-19	L4	LDR6h
**Pcsk1n**	0.729352921	2.68685901371011e-18	L4	LDR6h
**Eif1**	0.826534427	7.43325221311147e-17	L4	LDR6h
**Cox7c**	0.977977696	6.38370781022022e-16	L4	LDR6h
**Tpt1**	0.733629373	1.56451517699503e-15	L4	LDR6h
**Cox4i1**	0.735889098	5.69955421905079e-15	L4	LDR6h
**Slc25a4**	0.735336245	2.44874746904695e-14	L4	LDR6h
**Cox6c**	0.662325304	8.50841662415864e-14	L4	LDR6h
**Atxn7l3b**	0.674195583	9.45968474565112e-14	L4	LDR6h
**mt-Cytb**	1.044648326	5.65733181580012e-13	L4	LDR6h
**Gm27032**	0.997992942	7.98242915761036e-13	L4	LDR6h
**Rpl6**	0.834316054	1.97931700682126e-12	L4	LDR6h
**mt-Co2**	1.173636624	3.05220112944476e-12	L4	LDR6h
**Tmsb4x**	0.811692441	3.10417225799368e-12	L4	LDR6h
**Arf5**	0.632212901	9.32874015040648e-12	L4	LDR6h
**Oaz1**	0.649737627	9.48887461868141e-12	L4	LDR6h
**Atpif1**	0.702950294	1.6891551658415e-11	L4	LDR6h
**Dnaja2**	0.714577392	2.81939466289367e-10	L4	LDR6h
**Gm32250**	0.79842706	3.56002698097685e-10	L4	LDR6h

Development • Supplementary information

**Cox8a**	0.854623825	4.17321404835594e-10	L4	LDR6h
**Atp6v0c**	0.656152732	6.33228382902435e-10	L4	LDR6h
**Calm1**	0.706097286	1.61800967828337e-09	L4	LDR6h
**Rpl13**	0.653845288	1.76334346657153e-09	L4	LDR6h
**Bex2**	0.660730748	1.78276929957763e-09	L4	LDR6h
**Pdlim7**	0.653441981	1.27670740637896e-08	L4	LDR6h
**Tubb4a**	0.658037045	1.29444813607652e-08	L4	LDR6h
**Rpl9**	0.605736275	1.8069643403654e-08	L4	LDR6h
**Nrgn**	0.766578982	1.95882568987435e-08	L4	LDR6h
**B230217J21Rik**	0.593206264	3.08156508040335e-08	L4	LDR6h
**Rps8**	0.619532158	3.21643267594435e-08	L4	LDR6h
**Rpl41**	0.652808217	4.40398141478073e-08	L4	LDR6h
**Sigmar1**	0.681142939	4.6535826154432e-08	L4	LDR6h
**B230217C12Rik**	0.624031632	5.95310950026425e-08	L4	LDR6h
**Dact2**	0.7066112	6.12430471025456e-08	L4	LDR6h
**Ppia**	0.595832222	8.3568228810336e-08	L4	LDR6h
**Krt12**	0.671138378	8.89329400027051e-08	L4	LDR6h
**Ly6h**	0.602240014	9.64450851598985e-08	L4	LDR6h
**Lsm7**	0.612183873	1.52548498756191e-07	L4	LDR6h
**Cox7b**	0.653145134	1.54155061671276e-07	L4	LDR6h
**Ckb**	0.669132232	1.80732614928952e-07	L4	LDR6h
**Slc50a1**	0.593660666	1.82519116136289e-07	L4	LDR6h
**Dhps**	0.631427722	1.96334318443561e-07	L4	LDR6h
**Tmem243**	0.637020502	1.99365901314064e-07	L4	LDR6h
**BC031181**	0.610693197	2.19548632846155e-07	L4	LDR6h
**Rap2b**	0.677583851	3.03511183041967e-07	L4	LDR6h
**Rps21**	0.652118022	3.51630109938735e-07	L4	LDR6h
**mt-Atp6**	1.038103847	5.52646007678833e-07	L4	LDR6h
**Gps2**	0.60416848	1.01828414352511e-06	L4	LDR6h
**Jund**	0.589046182	2.91334040053329e-06	L4	LDR6h
**6430590A07Rik**	0.654999037	5.33392589156324e-06	L4	LDR6h
**Eef1a1**	0.652494372	1.4577552713277e-05	L4	LDR6h
**Atp6v0b**	1.032784445	1.49946728149661e-05	L4	LDR6h
**Rnf227**	0.600727828	1.55722251935578e-05	L4	LDR6h
**Cck**	0.621024893	2.21388209638788e-05	L4	LDR6h
**1110008P14Rik**	0.647493664	2.25634934518903e-05	L4	LDR6h
**Crebzf**	0.665912895	2.50135142720073e-05	L4	LDR6h
**Mrps26**	0.637227107	2.55675439133408e-05	L4	LDR6h
**Rad51ap2**	0.751888818	3.02060369145658e-05	L4	LDR6h

Development • Supplementary information

**4930473D10Rik**	0.593650324	3.64142633274861e-05	L4	LDR6h
**Gm15520**	0.620517697	4.19164241264962e-05	L4	LDR6h
**Gm35188**	0.864710161	4.42344210344672e-05	L4	LDR6h
**Mdh1**	0.610306777	6.37496543507679e-05	L4	LDR6h
**Tmem208**	0.62393368	9.08908049968177e-05	L4	LDR6h
**Nrn1**	0.94546961	9.81210460889516e-05	L4	LDR6h
**Tmed9**	0.606485419	0.000175824	L4	LDR6h
**Trim35**	0.650591338	0.000185873	L4	LDR6h
**P2ry14**	0.729080224	0.000213822	L4	LDR6h
**Prpf4b**	0.721974967	0.000341115	L4	LDR6h
**Gm28376**	0.629124695	0.000823826	L4	LDR6h
**BC005561**	0.599294851	0.001208235	L4	LDR6h
**Ndufaf7**	0.591066631	0.001356637	L4	LDR6h
**A230004M16Rik**	0.594178794	0.002201361	L4	LDR6h
**Gm26724**	0.585744691	0.002521709	L4	LDR6h
**Prelid3a**	0.653002794	0.00267657	L4	LDR6h
**Pura**	0.59053113	0.002767197	L4	LDR6h
**Rmdn1**	0.58776052	0.005830809	L4	LDR6h
**Hmgn3**	0.617337404	0.009431298	L4	LDR6h
**mt-Nd1**	0.97834513	0.01595303	L4	LDR6h
**Snhg20**	0.661064815	0.021293363	L4	LDR6h
**5330438D12Rik**	0.698781738	0.030684525	L4	LDR6h
**mt-Co3**	1.600681712	4.79239661219829e-27	L5IT	LDR2h
**mt-Co2**	1.506961439	9.74925129236493e-22	L5IT	LDR2h
**Nrn1**	1.172688633	1.39858026732903e-17	L5IT	LDR2h
**mt-Co1**	1.470974921	4.7378895911792e-15	L5IT	LDR2h
**Nptx2**	1.108873422	3.19772849656097e-14	L5IT	LDR2h
**Ptprn**	1.148210405	5.37763510494424e-14	L5IT	LDR2h
**mt-Nd1**	1.101768901	5.84404936274374e-14	L5IT	LDR2h
**Gm34544**	1.019288353	7.68954515496481e-14	L5IT	LDR2h
**1700016P03Rik**	1.152096603	1.28015564541759e-13	L5IT	LDR2h
**Diras2**	0.945681754	1.83311701947153e-13	L5IT	LDR2h
**Egr3**	0.972895892	3.91401487883677e-13	L5IT	LDR2h
**mt-Atp6**	1.319512631	2.59403378798002e-11	L5IT	LDR2h
**Npas4**	1.031857659	6.25542486708861e-10	L5IT	LDR2h
**Hectd2os**	0.855745717	1.27929549917425e-09	L5IT	LDR2h
**Klf10**	0.948763182	1.45276568481192e-07	L5IT	LDR2h
**Nr4a1**	0.77305757	2.63043694376864e-07	L5IT	LDR2h
**Oprm1**	0.991620118	5.9672619108407e-07	L5IT	LDR2h

Development • Supplementary information

**Ptgds**	0.690908112	8.96952266941169e-07	L5IT	LDR2h
**4930415C11Rik**	1.067327304	9.88421830981368e-07	L5IT	LDR2h
**Inf2**	0.78389207	1.0863132673428e-06	L5IT	LDR2h
**1700054A03Rik**	0.725252222	2.32389603787407e-06	L5IT	LDR2h
**C1ql3**	0.714130954	2.95958532634239e-06	L5IT	LDR2h
**Ubb**	0.812562724	5.28881912065367e-06	L5IT	LDR2h
**Atp6v0b**	0.914385572	8.49699272474964e-06	L5IT	LDR2h
**Baz1a**	0.611691402	9.73667390087226e-06	L5IT	LDR2h
**Bdnf**	0.633698438	1.36202752308373e-05	L5IT	LDR2h
**Jcad**	0.682460359	1.77861822231941e-05	L5IT	LDR2h
**1700085D07Rik**	0.783080464	2.4292269451296e-05	L5IT	LDR2h
**Cst3**	0.740334958	3.4829233158977e-05	L5IT	LDR2h
**Cap1**	0.683334001	4.37832631467254e-05	L5IT	LDR2h
**Hunk**	0.872047295	4.93331111139118e-05	L5IT	LDR2h
**Pabpc4**	0.680369283	8.50045587515331e-05	L5IT	LDR2h
**Dalrd3**	0.693622241	8.9705266153093e-05	L5IT	LDR2h
**Gm46367**	0.94320036	0.000110113	L5IT	LDR2h
**Fth1**	1.416864305	0.000110474	L5IT	LDR2h
**Mir670hg**	2.006777945	0.000147418	L5IT	LDR2h
**Mfap3l**	0.710733728	0.00022711	L5IT	LDR2h
**mt-Nd5**	0.620811755	0.00032291	L5IT	LDR2h
**Slc25a3**	0.783238388	0.000340807	L5IT	LDR2h
**Rps29**	0.636745585	0.000385234	L5IT	LDR2h
**Aldoa**	0.665779247	0.000389137	L5IT	LDR2h
**Eif1**	0.656677302	0.000432193	L5IT	LDR2h
**Cox4i1**	0.734612904	0.000476753	L5IT	LDR2h
**Cox7c**	0.663826475	0.000585112	L5IT	LDR2h
**Gda**	0.683687309	0.000666209	L5IT	LDR2h
**Klhl2**	0.768695482	0.000954125	L5IT	LDR2h
**mt-Nd4**	0.828656774	0.000981699	L5IT	LDR2h
**Hspa8**	0.636210689	0.001006322	L5IT	LDR2h
**Sema3c**	1.503242462	0.001103595	L5IT	LDR2h
**Calm1**	0.62268546	0.001320276	L5IT	LDR2h
**Gm14636**	0.629532801	0.001732333	L5IT	LDR2h
**Pcsk1**	0.708052161	0.001756543	L5IT	LDR2h
**Ppme1**	0.724922603	0.001880786	L5IT	LDR2h
**Phf21b**	0.648128549	0.003740626	L5IT	LDR2h
**Rpl6**	0.634069804	0.007083543	L5IT	LDR2h
**Cx3cl1**	0.636703369	0.008120134	L5IT	LDR2h

**Nrsn1**	0.629577366	0.008427524	L5IT	LDR2h
**Sertad2**	0.598539697	0.011957408	L5IT	LDR2h
**Homer1**	0.611941196	0.012567973	L5IT	LDR2h
**Ckb**	0.689298155	0.014087312	L5IT	LDR2h
**Cox8a**	0.60249718	0.027402548	L5IT	LDR2h
**4933413L06Rik**	0.601303192	0.037476982	L5IT	LDR2h
**Homer1**	2.799923323	1.59134773278228e-132	L5IT	LDR30m
**Egr3**	2.246628982	5.12011064503837e-77	L5IT	LDR30m
**Pcsk1**	1.948517668	1.55863491835425e-43	L5IT	LDR30m
**Gm47423**	1.675060905	2.49519853448035e-41	L5IT	LDR30m
**Sik2**	1.469597982	2.1373428969333e-37	L5IT	LDR30m
**Nr4a2**	1.586323454	6.66724423868205e-35	L5IT	LDR30m
**Tiparp**	1.427181537	1.58627549145682e-34	L5IT	LDR30m
**Plcxd2**	1.390936499	9.3125795083828e-34	L5IT	LDR30m
**Nr4a1**	1.331933098	3.45915250533117e-31	L5IT	LDR30m
**Rheb**	1.296078861	5.44707817459406e-30	L5IT	LDR30m
**Frmd6**	1.304313779	2.38520060700522e-27	L5IT	LDR30m
**Cpeb3**	1.103053447	8.16975720503485e-26	L5IT	LDR30m
**Slc25a25**	1.267271587	8.95934723349682e-25	L5IT	LDR30m
**Nr4a3**	2.065691371	9.4603592682321e-25	L5IT	LDR30m
**1700016P03Rik**	2.629617502	1.33103841159639e-23	L5IT	LDR30m
**Etv5**	1.368510757	5.5380158867437e-22	L5IT	LDR30m
**Per1**	1.199103775	1.58020621815932e-20	L5IT	LDR30m
**Arhgef3**	1.202617397	3.51689755212332e-20	L5IT	LDR30m
**Ube2ql1**	1.109601311	5.99338346547673e-20	L5IT	LDR30m
**Zdbf2**	1.224697464	7.40606964627779e-20	L5IT	LDR30m
**Med14**	1.066397247	7.8751698398958e-19	L5IT	LDR30m
**Arl5b**	1.03238477	2.91775191284636e-18	L5IT	LDR30m
**Mir670hg**	1.138350993	1.95568900994026e-17	L5IT	LDR30m
**Spred1**	0.994083821	1.27372268677537e-16	L5IT	LDR30m
**Mbp**	1.129114183	3.23483842255651e-16	L5IT	LDR30m
**Nptx2**	1.00052219	1.40272710378815e-15	L5IT	LDR30m
**Coq10b**	0.945874694	4.78613100826677e-15	L5IT	LDR30m
**Stk40**	0.999280512	1.10723799417436e-14	L5IT	LDR30m
**Phf21b**	0.980351329	2.35088642974313e-14	L5IT	LDR30m
**Efhd2**	0.921895803	2.65744319695049e-14	L5IT	LDR30m
**Ankrd33b**	1.019075992	2.39336471247764e-13	L5IT	LDR30m
**Gm17231**	0.883344974	2.68754106693513e-13	L5IT	LDR30m
**Fosb**	0.875007948	2.38643537760724e-12	L5IT	LDR30m

**Nrd1**	0.98193724	3.09320687399301e-12	L5IT	LDR30m
**Irs2**	0.902929011	3.38982599394832e-12	L5IT	LDR30m
**Slc6a17**	0.846021014	4.77147851825956e-12	L5IT	LDR30m
**Tbc1d9**	0.984360773	5.10411321211827e-12	L5IT	LDR30m
**P4ha1**	0.928772992	8.07292669705095e-12	L5IT	LDR30m
**Kdm6b**	0.81018149	1.25511928501917e-11	L5IT	LDR30m
**Rock2**	0.900683973	3.24676617183365e-11	L5IT	LDR30m
**Btaf1**	0.844334761	3.38242113244638e-11	L5IT	LDR30m
**Cpeb4**	0.870676418	3.62549857880116e-11	L5IT	LDR30m
**Trim9**	0.769958475	5.7269726096032e-11	L5IT	LDR30m
**R3hdm2**	0.799272385	9.10325215391978e-11	L5IT	LDR30m
**Mxi1**	0.866649494	1.78664587899835e-10	L5IT	LDR30m
**Ifrd1**	0.807047705	2.6432983136858e-10	L5IT	LDR30m
**Sik3**	1.002008905	3.37188062186372e-10	L5IT	LDR30m
**Rph3a**	0.98422359	5.56729580947789e-10	L5IT	LDR30m
**Hmgcr**	0.788169078	5.71322242940429e-10	L5IT	LDR30m
**Ina**	0.815281665	1.3339055184135e-09	L5IT	LDR30m
**Chgb**	0.869865943	1.57973031558124e-09	L5IT	LDR30m
**Baiap2**	0.859883368	2.67981712761722e-09	L5IT	LDR30m
**Pmepa1**	0.975822381	4.36103882750146e-09	L5IT	LDR30m
**Ctnnd1**	0.783603941	5.4480380969724e-09	L5IT	LDR30m
**Ece1**	1.021928397	8.78297540201435e-09	L5IT	LDR30m
**Mapk6**	0.776548438	1.27786999146307e-08	L5IT	LDR30m
**Hnrnpll**	0.959803537	1.86407354774039e-08	L5IT	LDR30m
**Fbl**	0.840678937	1.87286576137922e-08	L5IT	LDR30m
**Nectin1**	0.808726475	1.94415513890789e-08	L5IT	LDR30m
**Myh9**	0.75547499	2.63884905790944e-08	L5IT	LDR30m
**Ntrk2**	0.811775178	2.71477848617034e-08	L5IT	LDR30m
**Tbc1d1**	0.776095728	3.40974846026128e-08	L5IT	LDR30m
**Arpc2**	0.728635923	4.5358682786401e-08	L5IT	LDR30m
**Hspa4**	0.805579996	5.01425833921281e-08	L5IT	LDR30m
**Ptprn**	0.945891489	5.05524003110629e-08	L5IT	LDR30m
**Sik1**	0.623162829	5.15756299400812e-08	L5IT	LDR30m
**Trib1**	0.628419591	6.75166881963281e-08	L5IT	LDR30m
**Abhd2**	0.831316077	6.79251370875757e-08	L5IT	LDR30m
**Fosl2**	1.078946828	8.26778883731983e-08	L5IT	LDR30m
**Zbtb16**	1.011831659	1.02784665463104e-07	L5IT	LDR30m
**Tmem178**	0.891413972	1.05086856408512e-07	L5IT	LDR30m
**Npas4**	0.847688486	1.1132205382262e-07	L5IT	LDR30m

Development • Supplementary information

**Clstn3**	0.852538467	1.30557583062009e-07	L5IT	LDR30m
**Gm48747**	0.811207194	1.45874443001966e-07	L5IT	LDR30m
**Grasp**	0.657308959	1.53289845442742e-07	L5IT	LDR30m
**Bdnf**	0.790393511	2.31969337276053e-07	L5IT	LDR30m
**Spred2**	0.87061987	2.44016081551533e-07	L5IT	LDR30m
**Zswim6**	0.789566927	2.85746177609192e-07	L5IT	LDR30m
**March3**	0.702341412	2.86507683940758e-07	L5IT	LDR30m
**Jdp2**	0.823000937	3.11443058292332e-07	L5IT	LDR30m
**Psd3**	0.881100516	3.21732032801748e-07	L5IT	LDR30m
**Osbpl8**	0.750524549	3.28651751395688e-07	L5IT	LDR30m
**Cry2**	0.74239129	3.58232588946733e-07	L5IT	LDR30m
**Mest**	0.738696398	4.93897390884442e-07	L5IT	LDR30m
**Nudt4**	0.738243877	5.02036206030178e-07	L5IT	LDR30m
**Mast3**	0.835308361	5.85656180602933e-07	L5IT	LDR30m
**Tulp4**	0.781766193	7.09499719176702e-07	L5IT	LDR30m
**Per2**	0.789143278	9.86573413436057e-07	L5IT	LDR30m
**Klhl2**	0.649533972	1.42321683861571e-06	L5IT	LDR30m
**Ubtd2**	0.780269664	1.44054824648788e-06	L5IT	LDR30m
**Nap1l1**	0.662394974	2.50072020043669e-06	L5IT	LDR30m
**Fbxo33**	0.68108615	2.58492675544406e-06	L5IT	LDR30m
**Dusp14**	0.711124126	2.94872222617801e-06	L5IT	LDR30m
**Rims4**	0.807286433	7.0312960207941e-06	L5IT	LDR30m
**St8sia5**	0.710331836	8.02760110955631e-06	L5IT	LDR30m
**Cbarp**	0.744398265	1.08509075442863e-05	L5IT	LDR30m
**Numb**	0.627149526	1.12768049690504e-05	L5IT	LDR30m
**Nrn1**	0.837999758	1.18222705734719e-05	L5IT	LDR30m
**Tacc1**	0.665300382	1.26457974010412e-05	L5IT	LDR30m
**Ndfip2**	0.82303092	1.87463411633619e-05	L5IT	LDR30m
**Rab6b**	0.699977636	2.69385490751042e-05	L5IT	LDR30m
**Skil**	0.662340698	3.26482976799355e-05	L5IT	LDR30m
**Rgs7bp**	0.63206304	3.62777411550777e-05	L5IT	LDR30m
**Kdm7a**	0.691684898	3.99787208355896e-05	L5IT	LDR30m
**Dnajc1**	0.696687742	4.03969285558476e-05	L5IT	LDR30m
**Hsd17b12**	0.629877442	4.63271102722175e-05	L5IT	LDR30m
**Dleu2**	0.610859029	5.62102367324652e-05	L5IT	LDR30m
**Hsph1**	0.625585923	6.7666384130139e-05	L5IT	LDR30m
**Tgfbr1**	0.780621761	7.21365706928781e-05	L5IT	LDR30m
**Iqgap2**	0.662486122	7.25706747714054e-05	L5IT	LDR30m
**Pak1**	0.825519902	0.000106409	L5IT	LDR30m

Development • Supplementary information

**Arid3b**	0.631525705	0.000108074	L5IT	LDR30m
**Ak4**	0.625087844	0.000112942	L5IT	LDR30m
**Gabbr1**	0.6789879	0.000117342	L5IT	LDR30m
**Elovl5**	0.654116411	0.000164975	L5IT	LDR30m
**Sipa1l2**	0.632466332	0.000181614	L5IT	LDR30m
**Adora1**	0.692303231	0.000281953	L5IT	LDR30m
**Synj2**	0.635016788	0.000297982	L5IT	LDR30m
**Vmp1**	0.612568913	0.000532557	L5IT	LDR30m
**Gfod1**	0.82084995	0.000621052	L5IT	LDR30m
**Sept7**	0.652245011	0.000626728	L5IT	LDR30m
**Cx3cl1**	0.609117447	0.000637636	L5IT	LDR30m
**Myo1e**	0.636889701	0.000745606	L5IT	LDR30m
**Hivep1**	0.633686021	0.001076767	L5IT	LDR30m
**Stk10**	0.597551772	0.001870796	L5IT	LDR30m
**Mgrn1**	0.589603011	0.001871661	L5IT	LDR30m
**E330009J07Rik**	0.637541733	0.002397744	L5IT	LDR30m
**Cmip**	0.646486828	0.004336005	L5IT	LDR30m
**Mapk4**	0.743315396	0.004692188	L5IT	LDR30m
**Dlgap4**	0.589054934	0.007338801	L5IT	LDR30m
**Kcnk12**	0.586032596	0.007557918	L5IT	LDR30m
**Clip2**	0.59272452	0.012506666	L5IT	LDR30m
**Tet3**	0.597679824	0.01274167	L5IT	LDR30m
**Elmo1**	0.591061294	0.013480264	L5IT	LDR30m
**Pkia**	0.647628065	0.014023489	L5IT	LDR30m
**Ptn**	0.585096269	0.016142079	L5IT	LDR30m
**Atp1a1**	0.615490039	0.017864262	L5IT	LDR30m
**Gng2**	0.595265472	0.034378253	L5IT	LDR30m
**Homer1**	1.767907875	2.68683727561875e-75	L5IT	LDR4h
**Mir670hg**	1.762479359	4.53868180908429e-49	L5IT	LDR4h
**Phf21b**	1.45403155	6.52000176194817e-40	L5IT	LDR4h
**Rph3a**	1.073300824	1.67098567058727e-24	L5IT	LDR4h
**Egr3**	1.177106289	3.52669158919384e-21	L5IT	LDR4h
**Mapk4**	1.104052519	4.92334922691751e-21	L5IT	LDR4h
**Spred2**	1.096997725	3.4501236014167e-20	L5IT	LDR4h
**Hs3st2**	1.187010684	6.93300313242685e-20	L5IT	LDR4h
**Sik2**	1.220593363	1.12591994355808e-18	L5IT	LDR4h
**Ppm1h**	0.92155799	2.36370122857499e-17	L5IT	LDR4h
**Spred1**	0.933835757	3.31869287858087e-17	L5IT	LDR4h
**Zmiz1**	1.059545513	8.73243642277384e-17	L5IT	LDR4h

Development • Supplementary information

**Bdnf**	0.967337322	1.08970741045978e-15	L5IT	LDR4h
**Sgsm1**	1.276261369	2.03446404322074e-15	L5IT	LDR4h
**Slc6a17**	0.844467916	2.2742651267213e-15	L5IT	LDR4h
**Tmem178**	1.019122772	2.41458462918616e-15	L5IT	LDR4h
**Arhgap31**	0.951910796	2.51800921496631e-15	L5IT	LDR4h
**Nptx2**	1.041901318	3.30111662504686e-14	L5IT	LDR4h
**Tiparp**	0.924802788	4.95041689761979e-14	L5IT	LDR4h
**Ntrk2**	0.875086915	5.37355700277395e-14	L5IT	LDR4h
**1700016P03Rik**	1.734806529	1.31818767276137e-13	L5IT	LDR4h
**Pcsk1**	0.982593143	2.2482191139272e-13	L5IT	LDR4h
**Hectd2**	0.886959622	7.90073969935989e-13	L5IT	LDR4h
**Sorcs3**	1.156520471	1.33567727507424e-12	L5IT	LDR4h
**Kdm6b**	0.786391046	2.04785264307242e-11	L5IT	LDR4h
**Osbpl3**	0.883341348	4.08038618726781e-11	L5IT	LDR4h
**Mamld1**	0.7620887	4.65932428231451e-11	L5IT	LDR4h
**Scube1**	0.778880294	6.40503738603236e-11	L5IT	LDR4h
**Itgav**	0.774077162	1.06602652001767e-10	L5IT	LDR4h
**Etv5**	0.936491753	1.95547910904998e-10	L5IT	LDR4h
**Klhl2**	0.792099416	4.47879116437869e-10	L5IT	LDR4h
**Bcor**	0.80290225	7.3660856476819e-10	L5IT	LDR4h
**Rock2**	0.824306032	8.82877830161596e-10	L5IT	LDR4h
**Pawr**	0.798150856	1.4998111046201e-09	L5IT	LDR4h
**Tet3**	0.721371308	2.10356595518277e-09	L5IT	LDR4h
**Stk40**	0.752676593	6.46820325191432e-09	L5IT	LDR4h
**Baz1a**	0.774276337	1.10332462157607e-08	L5IT	LDR4h
**Nrn1**	0.835834722	1.79663231037397e-08	L5IT	LDR4h
**Pdzd2**	0.96440885	2.71617388421378e-08	L5IT	LDR4h
**Gm46367**	0.782839412	8.98410693785239e-08	L5IT	LDR4h
**Cdh11**	0.639714537	1.12151760309122e-07	L5IT	LDR4h
**Zbtb16**	0.761840489	1.15250192799952e-07	L5IT	LDR4h
**Slc9a5**	0.698109888	1.63410737819316e-07	L5IT	LDR4h
**Ppme1**	0.637546966	3.02322117982974e-07	L5IT	LDR4h
**Zdbf2**	0.681017121	4.72249086163724e-07	L5IT	LDR4h
**Hnrnpll**	0.658638965	4.96453910658732e-07	L5IT	LDR4h
**Nrxn2**	0.632862592	5.04823860123044e-07	L5IT	LDR4h
**Eml5**	0.760297112	5.67523681806177e-07	L5IT	LDR4h
**Frmd6**	0.766499446	6.22734709276917e-07	L5IT	LDR4h
**Unc13a**	0.643630903	1.02009140452699e-06	L5IT	LDR4h
**Dnajc1**	0.698346344	1.11441860139747e-06	L5IT	LDR4h

**Rheb**	0.641882671	1.40736602426589e-06	L5IT	LDR4h
**2510009E07Rik**	0.660998501	1.50745705945838e-06	L5IT	LDR4h
**Iqgap2**	0.798636009	2.1846812743017e-06	L5IT	LDR4h
**Elmo1**	0.825354073	2.42563851819664e-06	L5IT	LDR4h
**Hmgcr**	0.625764625	2.48645457209436e-06	L5IT	LDR4h
**Arhgef3**	0.634598517	5.66299932460009e-06	L5IT	LDR4h
**Dot1l**	0.613363208	7.1968664140254e-06	L5IT	LDR4h
**Mical2**	0.714100042	7.81114831297609e-06	L5IT	LDR4h
**Galnt14**	0.824249319	8.12454661683634e-06	L5IT	LDR4h
**Megf11**	1.510009978	9.49883224463952e-06	L5IT	LDR4h
**Brinp1**	0.682637076	1.1856318780998e-05	L5IT	LDR4h
**Stt3b**	0.644857367	1.33877991732801e-05	L5IT	LDR4h
**Mast3**	0.783016513	1.40578708510446e-05	L5IT	LDR4h
**Dlgap4**	0.600493609	1.44333391890237e-05	L5IT	LDR4h
**A730060N03Rik**	0.618861597	1.46292589017788e-05	L5IT	LDR4h
**Sik3**	0.718311726	1.94448776677159e-05	L5IT	LDR4h
**Ankrd33b**	0.594610699	1.99504363581199e-05	L5IT	LDR4h
**Nr4a1**	0.67197159	3.62912700931356e-05	L5IT	LDR4h
**Nrd1**	0.601193162	3.7314118033393e-05	L5IT	LDR4h
**Camk1g**	0.590045901	4.06367938458132e-05	L5IT	LDR4h
**Lrrk2**	0.62859273	4.36704124495828e-05	L5IT	LDR4h
**Prmt8**	0.622232446	4.37332606339003e-05	L5IT	LDR4h
**Clstn3**	0.639052253	4.89398202634266e-05	L5IT	LDR4h
**Gm13684**	0.611363826	5.50799999152455e-05	L5IT	LDR4h
**Sema3a**	0.6894032	6.148236569018e-05	L5IT	LDR4h
**Gmeb2**	0.601500369	7.78148671332674e-05	L5IT	LDR4h
**Rgs20**	0.745823917	8.39241559932268e-05	L5IT	LDR4h
**Inf2**	0.616801072	9.88645162198957e-05	L5IT	LDR4h
**Fstl4**	0.685323911	0.000105242	L5IT	LDR4h
**Cap2**	0.587335705	0.000109837	L5IT	LDR4h
**Nrp1**	0.725842251	0.000119154	L5IT	LDR4h
**Gdpd5**	0.626025329	0.000125504	L5IT	LDR4h
**Lingo1**	0.590727875	0.000141317	L5IT	LDR4h
**St8sia5**	0.628884312	0.000180748	L5IT	LDR4h
**Actn4**	0.587116139	0.000220414	L5IT	LDR4h
**Crtac1**	0.636610461	0.000280978	L5IT	LDR4h
**Ttll11**	0.606999348	0.000375594	L5IT	LDR4h
**Sorcs1**	0.622190607	0.000383526	L5IT	LDR4h
**Ece1**	0.625346707	0.001583457	L5IT	LDR4h

**Pcdh15**	0.600223693	0.001963735	L5IT	LDR4h
**Ptprn**	0.689370382	0.008830489	L5IT	LDR4h
**Maml3**	1.583825611	0.046132964	L5IT	LDR4h
**Fth1**	1.450381529	1.45026742707755e-32	L5IT	LDR6h
**mt-Co3**	1.469385217	2.69065905736538e-27	L5IT	LDR6h
**mt-Co2**	1.352429083	1.46501958015444e-21	L5IT	LDR6h
**mt-Co1**	1.521745432	2.86183544273744e-21	L5IT	LDR6h
**mt-Nd1**	1.046661233	5.64180523543058e-13	L5IT	LDR6h
**Oprm1**	1.021512873	8.77586769673379e-12	L5IT	LDR6h
**Srsf7**	1.088861958	3.40665244530108e-10	L5IT	LDR6h
**Ubb**	0.89800367	4.26196485770337e-10	L5IT	LDR6h
**Gm27032**	0.953945552	9.28286432330547e-10	L5IT	LDR6h
**mt-Atp6**	1.038182427	1.97113773876183e-09	L5IT	LDR6h
**Sema3c**	0.847685497	4.223638669409e-09	L5IT	LDR6h
**Dnaja2**	0.862766951	1.17926332704361e-08	L5IT	LDR6h
**H3f3b**	0.884105034	1.62255918774862e-08	L5IT	LDR6h
**Cst3**	0.799481709	2.56907357507292e-08	L5IT	LDR6h
**Rpl6**	0.919587847	4.68852255157626e-08	L5IT	LDR6h
**Gm46367**	0.99008304	6.25368386076462e-08	L5IT	LDR6h
**Gm32250**	0.82698089	1.17451726059472e-07	L5IT	LDR6h
**Ly6e**	0.815380054	1.36908420042662e-07	L5IT	LDR6h
**Nrn1**	0.965422273	1.91709388134169e-07	L5IT	LDR6h
**Cox7c**	0.848424659	6.39274006112274e-07	L5IT	LDR6h
**Diras2**	0.771707022	1.30512461892745e-06	L5IT	LDR6h
**Ckb**	0.809079697	3.15082638591979e-06	L5IT	LDR6h
**Cox4i1**	0.755245388	3.51023681793422e-06	L5IT	LDR6h
**Ift20**	0.767599691	4.62076104484063e-06	L5IT	LDR6h
**Eid1**	0.782177729	7.10884412276969e-06	L5IT	LDR6h
**Tmem91**	0.851402707	8.9289365737699e-06	L5IT	LDR6h
**Trim35**	0.822974888	1.18886903730594e-05	L5IT	LDR6h
**Cox8a**	0.729554471	1.56793755639037e-05	L5IT	LDR6h
**Calm1**	0.712558469	1.68681571615683e-05	L5IT	LDR6h
**Atp6v0b**	0.896566201	1.86961402066675e-05	L5IT	LDR6h
**Rmdn1**	0.770168447	1.95138256988065e-05	L5IT	LDR6h
**Cox7b**	0.760855313	2.20978138268382e-05	L5IT	LDR6h
**2900097C17Rik**	0.69953502	2.59139255944184e-05	L5IT	LDR6h
**Apoe**	0.690762965	2.64971575296209e-05	L5IT	LDR6h
**Kcnrg**	0.700668244	3.42891351222685e-05	L5IT	LDR6h
**Eif1**	0.715605374	6.7003011442241e-05	L5IT	LDR6h

Development • Supplementary information

**Krt12**	0.712646855	9.94918427511602e-05	L5IT	LDR6h
**Atpif1**	0.753805467	0.000112757	L5IT	LDR6h
**Slc25a4**	0.692332997	0.000119988	L5IT	LDR6h
**Rpl9**	0.657282767	0.000189123	L5IT	LDR6h
**Cox6c**	0.685405462	0.000233043	L5IT	LDR6h
**Arf5**	0.694387954	0.000242202	L5IT	LDR6h
**Gm15520**	0.769525665	0.000246673	L5IT	LDR6h
**Slc50a1**	0.68405089	0.000270916	L5IT	LDR6h
**Ppia**	0.669281604	0.000344807	L5IT	LDR6h
**Tigd2**	0.643605676	0.000363025	L5IT	LDR6h
**Rpl17**	0.615990214	0.00046759	L5IT	LDR6h
**Hectd2os**	0.664096953	0.000995792	L5IT	LDR6h
**4930587E11Rik**	0.703860252	0.001231761	L5IT	LDR6h
**Eef1a1**	0.739245873	0.001353888	L5IT	LDR6h
**6430590A07Rik**	0.646595594	0.001762228	L5IT	LDR6h
**Rps8**	0.652986363	0.002074068	L5IT	LDR6h
**Rap2b**	0.60181471	0.002368858	L5IT	LDR6h
**Inf2**	0.743603567	0.002622392	L5IT	LDR6h
**Rad51ap2**	0.676418749	0.002994103	L5IT	LDR6h
**Dtnbos**	0.760712784	0.00322409	L5IT	LDR6h
**Hmgn3**	0.678201483	0.003566325	L5IT	LDR6h
**Commd3**	0.619953355	0.003666827	L5IT	LDR6h
**Gm42439**	0.824546788	0.004363609	L5IT	LDR6h
**6330403K07Rik**	0.631730239	0.004514567	L5IT	LDR6h
**Shisa4**	1.303903274	0.00456906	L5IT	LDR6h
**Crebzf**	0.679175784	0.004703453	L5IT	LDR6h
**Rpl13**	0.634997873	0.00567757	L5IT	LDR6h
**Aldoa**	0.601428895	0.005802662	L5IT	LDR6h
**Tmsb4x**	0.690988976	0.006882832	L5IT	LDR6h
**Rprml**	0.695951682	0.007035186	L5IT	LDR6h
**Hagh**	0.654209329	0.007117758	L5IT	LDR6h
**Pabpc4**	0.668920033	0.007238523	L5IT	LDR6h
**5330438D12Rik**	0.731295662	0.007542474	L5IT	LDR6h
**Pdlim7**	0.611492686	0.011075088	L5IT	LDR6h
**BC005561**	0.629599573	0.011607669	L5IT	LDR6h
**Bex2**	0.624900945	0.011921793	L5IT	LDR6h
**Ssbp4**	0.605724194	0.015351185	L5IT	LDR6h
**Pura**	0.643743167	0.019934905	L5IT	LDR6h
**Cep78**	0.6023055	0.021550258	L5IT	LDR6h

**Pcsk1n**	0.585290697	0.02739558	L5IT	LDR6h
**B3galt2**	0.591141806	0.028272225	L5IT	LDR6h
**Gm28198**	0.588250294	0.029058772	L5IT	LDR6h
**Malat1**	1.223842368	0.038091092	L5IT	LDR6h
**Cck**	0.586312309	0.040986546	L5IT	LDR6h
**Hmgcr**	0.629545543	0.048026691	L5IT	LDR6h
**Sema3c**	1.714747441	2.8034911369455e-10	L5NP	LDR2h
**mt-Co3**	1.339583063	2.73427218352232e-09	L5NP	LDR2h
**mt-Co2**	1.233992755	2.31178501975442e-08	L5NP	LDR2h
**mt-Nd1**	1.013156306	0.000313395	L5NP	LDR2h
**Ptprn**	1.015388344	0.000717208	L5NP	LDR2h
**Zfpm2**	1.087287917	0.00078594	L5NP	LDR2h
**Nrgn**	1.01468699	0.003981443	L5NP	LDR2h
**Gm11867**	0.951945431	0.014409418	L5NP	LDR2h
**Cck**	0.942176421	0.016399813	L5NP	LDR2h
**mt-Cytb**	0.921435155	0.016804763	L5NP	LDR2h
**Calm1**	0.759879064	0.025457702	L5NP	LDR2h
**Lmo4**	0.791981188	0.028856676	L5NP	LDR2h
**Atp6v0b**	0.893768659	0.040750917	L5NP	LDR2h
**Eif1**	0.872758764	0.041387716	L5NP	LDR2h
**Ptgds**	0.833541253	0.045311835	L5NP	LDR2h
**Nr4a3**	1.833796567	2.97222624758674e-16	L5NP	LDR30m
**Pcsk1**	1.590844997	3.51500682523215e-14	L5NP	LDR30m
**1700016P03Rik**	2.323521257	4.0849870789659e-07	L5NP	LDR30m
**Egr3**	1.420705717	4.7765586344071e-07	L5NP	LDR30m
**Per1**	1.401028672	6.41182531673245e-07	L5NP	LDR30m
**Homer1**	2.050942329	1.27109386964226e-06	L5NP	LDR30m
**Etv5**	1.234491766	3.14594184679459e-06	L5NP	LDR30m
**Tiparp**	1.301668179	1.07756596926339e-05	L5NP	LDR30m
**Sik2**	1.078764334	1.54596446012979e-05	L5NP	LDR30m
**Zdbf2**	1.036948328	0.00166447	L5NP	LDR30m
**Chgb**	0.873752851	0.020132943	L5NP	LDR30m
**Ece1**	0.989028634	0.024498915	L5NP	LDR30m
**Homer1**	1.141279037	9.53446452246529e-09	L5NP	LDR4h
**1700016P03Rik**	1.19553462	8.37114328799779e-05	L5NP	LDR4h
**Pcsk1**	1.006705477	0.000287726	L5NP	LDR4h
**Sik2**	0.775566266	0.006522788	L5NP	LDR4h
**Fth1**	1.530258422	3.11740807905051e-12	L5NP	LDR6h
**mt-Co3**	1.310499575	1.40141496772039e-10	L5NP	LDR6h

**Malat1**	1.060600748	5.15306964543041e-07	L5NP	LDR6h
**Srsf7**	1.040716146	3.27925752965613e-06	L5NP	LDR6h
**mt-Co2**	1.07168733	9.56892724219616e-06	L5NP	LDR6h
**Eif1**	1.063290454	0.000626574	L5NP	LDR6h
**Shisa4**	0.947991664	0.009011461	L5NP	LDR6h
**Nrg3os**	0.699482759	0.012031641	L5NP	LDR6h
**Prpf4b**	0.786262832	0.01702044	L5NP	LDR6h
**Sema3c**	0.878542569	0.027502892	L5NP	LDR6h
**mt-Nd1**	0.912543764	0.048264432	L5NP	LDR6h
**1700016P03Rik**	1.375693658	4.26386965353832e-20	L5PT	LDR2h
**mt-Co3**	1.355940894	2.26946004469233e-19	L5PT	LDR2h
**Ptprn**	1.234070452	3.26179726651326e-19	L5PT	LDR2h
**mt-Co1**	1.193504331	1.95819038371833e-11	L5PT	LDR2h
**Apoe**	1.025872554	2.05548162491235e-10	L5PT	LDR2h
**mt-Cytb**	1.010470686	1.88329174438332e-09	L5PT	LDR2h
**Nr4a3**	0.824779234	1.98035391271901e-08	L5PT	LDR2h
**mt-Co2**	1.095790794	3.48091448186986e-08	L5PT	LDR2h
**C1ql3**	0.831232289	4.57829428837473e-07	L5PT	LDR2h
**Diras2**	0.822888699	2.05850650465385e-06	L5PT	LDR2h
**mt-Nd1**	0.813882127	1.19479498840568e-05	L5PT	LDR2h
**Gm11867**	0.662757215	1.80986430479608e-05	L5PT	LDR2h
**Ptgds**	0.656869071	2.15027338320397e-05	L5PT	LDR2h
**Baz1a**	0.741459481	3.09226078885293e-05	L5PT	LDR2h
**Pabpc4**	0.790861723	5.02985409959498e-05	L5PT	LDR2h
**Arc**	0.710261167	5.84447379505371e-05	L5PT	LDR2h
**Gm37229**	0.602359821	0.000102973	L5PT	LDR2h
**Atp6v0b**	0.788821753	0.000353655	L5PT	LDR2h
**Bdnf**	0.629569832	0.000696836	L5PT	LDR2h
**mt-Nd4**	0.748787303	0.001124291	L5PT	LDR2h
**mt-Nd2**	0.847745499	0.0013794	L5PT	LDR2h
**Pcsk1**	0.706414132	0.001384182	L5PT	LDR2h
**Hspa8**	0.677699191	0.001496091	L5PT	LDR2h
**Ahi1**	0.58705304	0.00297661	L5PT	LDR2h
**Nrsn1**	0.643753338	0.004066976	L5PT	LDR2h
**Npas4**	0.646502342	0.006535748	L5PT	LDR2h
**Cox7c**	0.727590287	0.008025169	L5PT	LDR2h
**1110008P14Rik**	0.608898596	0.011016014	L5PT	LDR2h
**Homer1**	0.592532274	0.014056871	L5PT	LDR2h
**1700054A03Rik**	0.602828464	0.015975395	L5PT	LDR2h

**B3galt2**	0.732829474	0.016472823	L5PT	LDR2h
**Prpf4b**	0.601462659	0.041281916	L5PT	LDR2h
**Gm11290**	0.585638049	0.048494969	L5PT	LDR2h
**1700016P03Rik**	2.712601391	8.7408927350623e-87	L5PT	LDR30m
**Homer1**	2.209683993	1.53442568326137e-86	L5PT	LDR30m
**Nr4a3**	2.510258573	9.95668428704275e-72	L5PT	LDR30m
**Pcsk1**	2.043655657	7.65804100877708e-48	L5PT	LDR30m
**Gm47423**	1.67469901	1.05766621968097e-42	L5PT	LDR30m
**Etv5**	1.542119642	5.39499238304231e-36	L5PT	LDR30m
**Pdlim1**	1.526832833	8.98943197493046e-32	L5PT	LDR30m
**Zdbf2**	1.641774247	2.7809231375494e-31	L5PT	LDR30m
**Arhgef3**	1.369556718	6.41935700992958e-27	L5PT	LDR30m
**Frmd6**	1.34484066	1.28761798431166e-25	L5PT	LDR30m
**Ube2ql1**	1.294965904	3.08855666701521e-24	L5PT	LDR30m
**Rheb**	1.345505426	8.68717128214707e-24	L5PT	LDR30m
**Sik2**	1.390017265	1.02044301268988e-23	L5PT	LDR30m
**Slc25a25**	1.270650421	1.04017012199245e-23	L5PT	LDR30m
**Arl5b**	1.338846501	4.62356324712824e-22	L5PT	LDR30m
**Per1**	1.218189774	5.20851101053794e-21	L5PT	LDR30m
**Hspa4**	1.171664026	7.44451532224266e-21	L5PT	LDR30m
**Egr3**	1.273089997	7.95530963096224e-21	L5PT	LDR30m
**Rnf217**	1.194684786	1.22090492893444e-20	L5PT	LDR30m
**Plcxd2**	1.256314026	1.45624377332705e-19	L5PT	LDR30m
**Bdnf**	1.176444029	1.76918020618682e-19	L5PT	LDR30m
**Ina**	1.174648756	4.99911714386274e-18	L5PT	LDR30m
**Cpeb3**	0.929563973	8.52035276307469e-18	L5PT	LDR30m
**Fosl2**	1.190573082	2.13790493478456e-17	L5PT	LDR30m
**Nr4a1**	1.065092997	2.73856258046588e-17	L5PT	LDR30m
**Hmgcr**	1.078321554	8.73035870325483e-16	L5PT	LDR30m
**Osbpl8**	0.962246067	1.17760493722192e-15	L5PT	LDR30m
**Nr4a2**	1.685082859	1.17061029477255e-14	L5PT	LDR30m
**Ndfip2**	1.310521778	3.43852008983662e-14	L5PT	LDR30m
**Rgs7bp**	0.850045841	6.25238257292447e-13	L5PT	LDR30m
**Rims4**	1.058407386	1.09753336045509e-12	L5PT	LDR30m
**Irs2**	1.032192478	1.68650584421967e-12	L5PT	LDR30m
**Tiparp**	1.746914234	4.3389608010544e-12	L5PT	LDR30m
**Ntrk2**	0.643850136	4.05845310361217e-11	L5PT	LDR30m
**Coq10b**	0.92906686	1.05577795949138e-10	L5PT	LDR30m
**Trim9**	0.725180217	1.06382819405765e-10	L5PT	LDR30m

Development • Supplementary information

**Fbxo33**	0.920523598	1.20532247869284e-10	L5PT	LDR30m
**Clstn3**	0.938978284	2.71326505166525e-10	L5PT	LDR30m
**Ptprn**	0.982773455	3.04956258849013e-10	L5PT	LDR30m
**4931406P16Rik**	0.865766743	4.6077883690337e-10	L5PT	LDR30m
**Elovl5**	0.887928756	1.0097579327449e-09	L5PT	LDR30m
**Arpc2**	0.846094678	1.66752686134095e-09	L5PT	LDR30m
**Nrd1**	0.867661459	1.93197163494289e-09	L5PT	LDR30m
**Rcan2**	0.955190055	2.33317853973407e-09	L5PT	LDR30m
**Btaf1**	0.780056145	2.74497382504845e-09	L5PT	LDR30m
**Vmp1**	0.769075276	3.54890725450535e-09	L5PT	LDR30m
**Fosb**	0.783059734	3.89375996117476e-09	L5PT	LDR30m
**Prkar2a**	0.862537994	4.31420430158892e-09	L5PT	LDR30m
**R3hdm2**	0.676184552	4.33321305949823e-09	L5PT	LDR30m
**Ankrd33b**	0.93075618	4.52719174105277e-09	L5PT	LDR30m
**Kdm7a**	0.847645059	4.58721019905686e-09	L5PT	LDR30m
**Scg3**	0.977181112	4.81652225992914e-09	L5PT	LDR30m
**Stk40**	0.878411857	6.98202884773841e-09	L5PT	LDR30m
**Rabgef1**	0.854709192	8.46008168116186e-09	L5PT	LDR30m
**Atp1a1**	0.822886819	8.87542837969024e-09	L5PT	LDR30m
**Ago3**	0.793017022	1.07132014871828e-08	L5PT	LDR30m
**Kdm6b**	0.807721861	3.09358396546041e-08	L5PT	LDR30m
**Chgb**	0.822304233	6.33425956141799e-08	L5PT	LDR30m
**P4ha1**	0.870868907	7.30111359652183e-08	L5PT	LDR30m
**Spred1**	0.954013137	1.20736255320815e-07	L5PT	LDR30m
**Nap1l1**	0.763343045	1.61898159386168e-07	L5PT	LDR30m
**Eprs**	0.811053591	1.63516143090753e-07	L5PT	LDR30m
**Cwc25**	0.779796644	5.70507862059103e-07	L5PT	LDR30m
**Nt5dc3**	0.728865136	6.33951877075961e-07	L5PT	LDR30m
**Lonrf1**	0.790185149	6.55870339983544e-07	L5PT	LDR30m
**Zc3h12c**	0.763369434	7.34783036253129e-07	L5PT	LDR30m
**Cabp1**	0.660296332	1.04029864163311e-06	L5PT	LDR30m
**Bcl6**	0.792563958	1.15405114537914e-06	L5PT	LDR30m
**Ciart**	0.716080904	1.30423813725315e-06	L5PT	LDR30m
**Per2**	0.801758036	1.31148792389391e-06	L5PT	LDR30m
**Sept7**	0.804970531	1.35659569537058e-06	L5PT	LDR30m
**Sik1**	0.65773737	2.34746560593728e-06	L5PT	LDR30m
**Med14**	0.787745147	2.41237473628689e-06	L5PT	LDR30m
**Hnrnpll**	0.753553583	2.70866927021918e-06	L5PT	LDR30m
**Gm3294**	0.751342886	3.61187992080204e-06	L5PT	LDR30m

Development • Supplementary information

**Tulp4**	0.78590649	4.28140735421721e-06	L5PT	LDR30m
**Mia3**	0.726384921	7.94228435996734e-06	L5PT	LDR30m
**Cdc42ep3**	0.83159949	8.37893872980756e-06	L5PT	LDR30m
**Fbl**	0.747979427	9.56209759545365e-06	L5PT	LDR30m
**Mapk4**	0.84810162	1.05997981825037e-05	L5PT	LDR30m
**Arc**	0.708914202	1.16168670819008e-05	L5PT	LDR30m
**Zswim6**	0.751111797	2.2219573869103e-05	L5PT	LDR30m
**Gm17231**	0.752372217	2.57827892416994e-05	L5PT	LDR30m
**Ifrd1**	0.704490389	2.61652836635773e-05	L5PT	LDR30m
**Cnnm1**	0.692109427	3.67324495131081e-05	L5PT	LDR30m
**Grasp**	0.701773257	3.78515094661565e-05	L5PT	LDR30m
**Npas4**	0.700536085	3.81479850638317e-05	L5PT	LDR30m
**Kpna1**	0.739859979	4.21965759589882e-05	L5PT	LDR30m
**Sik3**	0.728738818	4.4326707537698e-05	L5PT	LDR30m
**Grhl1**	0.744253548	4.62205732906548e-05	L5PT	LDR30m
**Rcc2**	0.751327646	5.15397395563329e-05	L5PT	LDR30m
**Sgsm1**	0.735808115	5.35600875504501e-05	L5PT	LDR30m
**Pak1**	0.818668047	6.970084830628e-05	L5PT	LDR30m
**Slc7a8**	0.702303478	7.11520990937634e-05	L5PT	LDR30m
**Mast3**	0.74299331	8.41297179335056e-05	L5PT	LDR30m
**Ipmk**	0.686101814	8.80875228764432e-05	L5PT	LDR30m
**Cpeb4**	0.705711138	9.43531696060292e-05	L5PT	LDR30m
**Ap2b1**	0.598515827	0.000100432	L5PT	LDR30m
**Myh9**	0.689937747	0.000107203	L5PT	LDR30m
**Trib1**	0.620784039	0.000119692	L5PT	LDR30m
**Hsd17b12**	0.662429379	0.000168501	L5PT	LDR30m
**Zbtb11**	0.641272298	0.000186903	L5PT	LDR30m
**Klhl2**	0.591463616	0.000211588	L5PT	LDR30m
**Gm28294**	0.598279934	0.00022315	L5PT	LDR30m
**Gm48747**	0.681414114	0.000225254	L5PT	LDR30m
**Hsph1**	0.74109037	0.000300596	L5PT	LDR30m
**Dock1**	0.661822036	0.000337855	L5PT	LDR30m
**Gm15398**	0.774471662	0.000370362	L5PT	LDR30m
**Cds1**	0.62059123	0.000388075	L5PT	LDR30m
**Dnajc1**	0.656801525	0.000492138	L5PT	LDR30m
**Basp1**	0.601036564	0.000525001	L5PT	LDR30m
**Mest**	0.637636748	0.000561163	L5PT	LDR30m
**Ivns1abp**	0.661661957	0.000636137	L5PT	LDR30m
**Peli1**	0.725072409	0.000652127	L5PT	LDR30m

Development • Supplementary information

**Ece1**	0.689049951	0.000938768	L5PT	LDR30m
**Phf21b**	0.644348388	0.001037212	L5PT	LDR30m
**Usp36**	0.667835394	0.001072506	L5PT	LDR30m
**Arih2**	0.661630298	0.001162392	L5PT	LDR30m
**Stk38l**	0.670854532	0.001268376	L5PT	LDR30m
**Insyn2a**	0.632868409	0.001277928	L5PT	LDR30m
**Atp2a2**	0.596335376	0.001279186	L5PT	LDR30m
**Cbarp**	0.646478571	0.002283173	L5PT	LDR30m
**Fam91a1**	0.639229699	0.002464688	L5PT	LDR30m
**Kif5c**	0.59688935	0.003123935	L5PT	LDR30m
**Pvr**	0.646829593	0.003464243	L5PT	LDR30m
**Lemd3**	0.609293802	0.003525281	L5PT	LDR30m
**Jdp2**	0.664486336	0.010672107	L5PT	LDR30m
**Mbp**	0.665322405	0.011037671	L5PT	LDR30m
**Lrrc28**	0.587899555	0.014135809	L5PT	LDR30m
**Phyhipl**	0.602084557	0.015923328	L5PT	LDR30m
**Mn1**	0.609550951	0.028337512	L5PT	LDR30m
**Homer1**	1.612454619	1.81160302791858e-70	L5PT	LDR4h
**1700016P03Rik**	1.948284884	3.67332336501551e-52	L5PT	LDR4h
**Pcsk1**	1.367901211	1.2627814515595e-25	L5PT	LDR4h
**Sgsm1**	1.314580359	4.08240263453636e-23	L5PT	LDR4h
**Baz1a**	1.225967559	1.15284505088207e-20	L5PT	LDR4h
**Rgs20**	1.287186531	1.27221145571851e-20	L5PT	LDR4h
**Etv5**	1.084978544	1.51031169312433e-19	L5PT	LDR4h
**Mapk4**	1.08041695	2.87930400323257e-17	L5PT	LDR4h
**Nr4a3**	1.089294358	1.02634528074104e-16	L5PT	LDR4h
**Sik2**	1.112489706	1.54077706936137e-16	L5PT	LDR4h
**Bdnf**	1.033421264	5.26489737841776e-15	L5PT	LDR4h
**Pdlim1**	1.096793031	2.15597440055907e-13	L5PT	LDR4h
**Tiparp**	1.00041231	2.29205959452473e-13	L5PT	LDR4h
**Osbpl3**	0.93162048	2.68252677611365e-13	L5PT	LDR4h
**Ntrk2**	0.728951916	3.397108167243e-13	L5PT	LDR4h
**Zdbf2**	1.014314741	1.11611574361572e-12	L5PT	LDR4h
**Rph3a**	0.939204959	1.55266250788733e-12	L5PT	LDR4h
**Spred1**	1.027701736	1.11450960930481e-11	L5PT	LDR4h
**Osbpl8**	0.729323906	2.99924685712689e-11	L5PT	LDR4h
**Phf21b**	0.913247263	4.89406219999749e-11	L5PT	LDR4h
**Tmem163**	0.790161115	8.52013850127175e-11	L5PT	LDR4h
**Osbpl6**	0.70288639	1.01080754635985e-10	L5PT	LDR4h

**R3hdm1**	0.640615794	1.11977322617649e-09	L5PT	LDR4h
**Tet3**	0.865484325	1.14489646787007e-09	L5PT	LDR4h
**Mir670hg**	0.86517637	1.76999700677033e-09	L5PT	LDR4h
**Rcan2**	0.828367337	2.54307281301171e-09	L5PT	LDR4h
**Sik3**	0.767089125	1.16888957163506e-08	L5PT	LDR4h
**Frmd6**	0.783303847	3.3318931959327e-08	L5PT	LDR4h
**Sorbs1**	0.71752701	6.0819190744973e-08	L5PT	LDR4h
**Ipmk**	0.778353005	6.82595852223938e-08	L5PT	LDR4h
**Rps6ka3**	0.626949993	2.1526168278104e-07	L5PT	LDR4h
**Numb**	0.686973997	3.22350003139824e-07	L5PT	LDR4h
**Epha10**	0.65326078	4.25712921028838e-07	L5PT	LDR4h
**Dnajc1**	0.673727573	5.38552795560172e-07	L5PT	LDR4h
**Spred2**	0.696255997	6.21031710732427e-07	L5PT	LDR4h
**Ago3**	0.641812156	8.39978483393637e-07	L5PT	LDR4h
**Irs2**	0.778007516	1.01873856024151e-06	L5PT	LDR4h
**Ankrd33b**	0.755143502	1.20337309227099e-06	L5PT	LDR4h
**Hmgcr**	0.730934005	1.53937803688703e-06	L5PT	LDR4h
**Klhl2**	0.589398664	1.9537274867277e-06	L5PT	LDR4h
**Gm46367**	0.782841756	2.10492805374783e-06	L5PT	LDR4h
**Zswim6**	0.603018416	2.40967407019369e-06	L5PT	LDR4h
**Slc6a17**	0.609182665	6.83550834909848e-06	L5PT	LDR4h
**Lrrk2**	0.648221199	7.81814953849845e-06	L5PT	LDR4h
**Fndc3a**	0.715337761	9.13518328292523e-06	L5PT	LDR4h
**Nap1l1**	0.63363831	1.89542766532109e-05	L5PT	LDR4h
**Unc13a**	0.594838452	3.46108360855442e-05	L5PT	LDR4h
**Per2**	0.647713991	4.35223088389702e-05	L5PT	LDR4h
**Arhgap31**	0.652123834	4.43425921091333e-05	L5PT	LDR4h
**Rock2**	0.590718184	0.000104597	L5PT	LDR4h
**Etl4**	0.660974261	0.000194153	L5PT	LDR4h
**Ppme1**	0.605905663	0.000198406	L5PT	LDR4h
**Wwtr1**	0.68689187	0.000243603	L5PT	LDR4h
**Hnrnpll**	0.595760481	0.000267141	L5PT	LDR4h
**Clstn3**	0.66522897	0.000625346	L5PT	LDR4h
**Hsph1**	0.605399043	0.000703339	L5PT	LDR4h
**Eml5**	0.622466596	0.000933523	L5PT	LDR4h
**Cop1**	0.59834172	0.001134084	L5PT	LDR4h
**Lrrc28**	0.619846425	0.001446471	L5PT	LDR4h
**March11**	0.601507223	0.001889386	L5PT	LDR4h
**Siah3**	0.616923884	0.00250719	L5PT	LDR4h

**Mast3**	0.631119836	0.003617826	L5PT	LDR4h
**Chst8**	0.614210493	0.006335923	L5PT	LDR4h
**Lmo7**	0.6224643	0.00769796	L5PT	LDR4h
**Scg3**	0.618384148	0.012945996	L5PT	LDR4h
**Slc25a25**	0.588478781	0.018244657	L5PT	LDR4h
**Gm15398**	1.173701202	0.022219959	L5PT	LDR4h
**Plcl1**	0.597907148	0.046627291	L5PT	LDR4h
**mt-Co3**	1.421787012	4.00589251812728e-28	L5PT	LDR6h
**mt-Co1**	1.373366858	1.42277032291148e-20	L5PT	LDR6h
**Fth1**	1.20919603	1.70747384167356e-17	L5PT	LDR6h
**Srsf7**	1.23366562	2.18135773271606e-17	L5PT	LDR6h
**mt-Cytb**	1.142013561	3.58183773707315e-14	L5PT	LDR6h
**Gm27032**	0.986112248	1.26712970587673e-08	L5PT	LDR6h
**Apoe**	0.85915297	1.31776300035567e-08	L5PT	LDR6h
**mt-Nd1**	0.875880942	2.2448714617313e-07	L5PT	LDR6h
**Cox7c**	0.926638552	2.11436692810783e-06	L5PT	LDR6h
**Gm15563**	0.897818859	8.23633445371317e-06	L5PT	LDR6h
**Atp6v0b**	0.826905852	8.34216270089591e-06	L5PT	LDR6h
**H3f3b**	0.821995551	1.3429547604108e-05	L5PT	LDR6h
**Tigd2**	0.733495816	5.10005555808102e-05	L5PT	LDR6h
**B3galt2**	0.795001648	6.54502587000845e-05	L5PT	LDR6h
**Trim35**	0.778727872	8.80914631696922e-05	L5PT	LDR6h
**Gm35188**	0.884140192	0.00012954	L5PT	LDR6h
**4933413L06Rik**	0.834607045	0.000166711	L5PT	LDR6h
**Ubb**	0.716649752	0.000176329	L5PT	LDR6h
**Eif1**	0.756051263	0.00019154	L5PT	LDR6h
**Tmsb4x**	0.785472046	0.000195457	L5PT	LDR6h
**5330438D12Rik**	0.804385002	0.000254825	L5PT	LDR6h
**Nrg3os**	0.724757713	0.000513273	L5PT	LDR6h
**Gm32250**	0.773418213	0.000654614	L5PT	LDR6h
**Rpl6**	0.730791151	0.000701407	L5PT	LDR6h
**Mdh1**	0.714349152	0.001308116	L5PT	LDR6h
**Nap1l5**	0.713196458	0.001542173	L5PT	LDR6h
**Gm46367**	0.739270093	0.001579954	L5PT	LDR6h
**Atpif1**	0.703577304	0.001958718	L5PT	LDR6h
**mt-Nd4**	0.739222626	0.002123325	L5PT	LDR6h
**Arf5**	0.647342959	0.002252632	L5PT	LDR6h
**C1ql3**	0.769015218	0.002332611	L5PT	LDR6h
**Sema3c**	0.689557441	0.004763539	L5PT	LDR6h

Development • Supplementary information

**Mrpl38**	0.640666854	0.006054906	L5PT	LDR6h
**Rbm39**	0.658378758	0.006236601	L5PT	LDR6h
**Prpf4b**	0.643695007	0.006588971	L5PT	LDR6h
**Eef1a1**	0.690040033	0.006646482	L5PT	LDR6h
**Cox8a**	0.673445221	0.007287325	L5PT	LDR6h
**Gm48678**	0.73399006	0.008032189	L5PT	LDR6h
**Slc50a1**	0.642958863	0.008279131	L5PT	LDR6h
**Pet100**	0.632595163	0.010069223	L5PT	LDR6h
**Slc16a11**	0.6691112	0.01027946	L5PT	LDR6h
**Nrgn**	0.634790115	0.012117354	L5PT	LDR6h
**Gm20275**	0.655370717	0.013599003	L5PT	LDR6h
**Malat1**	1.193629656	0.014681259	L5PT	LDR6h
**Pcsk1n**	0.586934327	0.021847149	L5PT	LDR6h
**Ppia**	0.612247475	0.02321517	L5PT	LDR6h
**Cox4i1**	0.625820275	0.025200544	L5PT	LDR6h
**Bmyc**	0.646185733	0.025492317	L5PT	LDR6h
**Dnaja2**	0.644896816	0.026947606	L5PT	LDR6h
**Crebzf**	0.653367864	0.03294366	L5PT	LDR6h
**Ndufa10**	0.648134566	0.037382375	L5PT	LDR6h
**Atxn7l3b**	0.606492109	0.041996141	L5PT	LDR6h
**1110008P14Rik**	0.640987467	0.045874855	L5PT	LDR6h
**Nrn1**	1.588324391	2.09985755259955e-58	L6a	LDR2h
**Ccn1**	1.210165357	1.49942033190221e-45	L6a	LDR2h
**Egr3**	1.192037407	2.58109969491499e-45	L6a	LDR2h
**Gm34544**	1.368970661	8.1711975493247e-36	L6a	LDR2h
**Ptprn**	1.332304362	5.80606420400738e-32	L6a	LDR2h
**Nptx2**	1.102856114	3.13832249834844e-29	L6a	LDR2h
**Npas4**	1.245815903	5.4122343317527e-27	L6a	LDR2h
**Fosb**	0.814623163	2.04706866516481e-25	L6a	LDR2h
**Egr4**	0.787715325	7.30948568153872e-25	L6a	LDR2h
**mt-Co1**	1.446573012	1.36879931841503e-23	L6a	LDR2h
**mt-Co3**	1.451792306	2.02058059384181e-23	L6a	LDR2h
**Gm21798**	0.940110791	2.55025790391807e-20	L6a	LDR2h
**Cx3cl1**	0.969758629	5.60327420472238e-20	L6a	LDR2h
**Rnd3**	0.832823532	2.38886142758764e-19	L6a	LDR2h
**Fos**	0.767475451	1.18390831856134e-18	L6a	LDR2h
**Grasp**	0.793317083	2.90812411283483e-18	L6a	LDR2h
**Fosl2**	0.664529499	1.97258347858872e-17	L6a	LDR2h
**Hspa8**	0.877677503	9.63877846559687e-17	L6a	LDR2h

Development • Supplementary information

**Gadd45b**	0.649983182	1.11816583662978e-16	L6a	LDR2h
**Nefl**	0.818359395	1.36851941157481e-16	L6a	LDR2h
**Pcsk1**	0.850877997	1.45916722770366e-16	L6a	LDR2h
**mt-Co2**	1.279903003	6.00223784736796e-16	L6a	LDR2h
**Gm37229**	0.811077905	1.14357541568079e-15	L6a	LDR2h
**4933413L06Rik**	1.11561716	4.44236861517336e-15	L6a	LDR2h
**P4ha1**	0.876421576	5.00258456867968e-15	L6a	LDR2h
**Scg2**	0.663864837	5.76466491345593e-15	L6a	LDR2h
**Nefm**	0.830543443	1.08304094890707e-14	L6a	LDR2h
**Inhba**	0.881245859	1.14576849430461e-14	L6a	LDR2h
**Homer1**	1.018566366	1.33616174699514e-14	L6a	LDR2h
**Ina**	0.793830484	4.9363703791719e-14	L6a	LDR2h
**Gm4128**	0.7491242	7.36243719831244e-14	L6a	LDR2h
**Tiparp**	0.630469572	7.71513789099498e-14	L6a	LDR2h
**Nr4a1**	0.837104285	9.19879034504996e-14	L6a	LDR2h
**Nr4a2**	0.762710816	1.07920931854976e-13	L6a	LDR2h
**Cap1**	0.773993626	9.17873073142098e-13	L6a	LDR2h
**mt-Nd1**	0.981421616	1.54584926533075e-12	L6a	LDR2h
**Fbxo33**	0.712755516	1.47926923881204e-11	L6a	LDR2h
**Slc25a3**	0.93043052	1.85530206480477e-11	L6a	LDR2h
**Dalrd3**	0.711406749	2.34311658042062e-10	L6a	LDR2h
**Rpl41**	0.702384203	1.02574759386491e-09	L6a	LDR2h
**Nrsn1**	0.727591908	1.51188504841357e-09	L6a	LDR2h
**Spry2**	0.607564549	2.15781093384926e-09	L6a	LDR2h
**Arpp19**	0.667108409	2.90550348596462e-09	L6a	LDR2h
**Ppme1**	0.905096293	3.91013284386014e-09	L6a	LDR2h
**Eif1**	0.869676532	4.05359270802816e-09	L6a	LDR2h
**1700016P03Rik**	1.731819976	4.390034428368e-09	L6a	LDR2h
**Jund**	0.625910643	7.89971321441879e-09	L6a	LDR2h
**Elmo1**	0.932897702	2.30296629831371e-08	L6a	LDR2h
**Sema3c**	1.6924672	2.48734064956664e-08	L6a	LDR2h
**Aldoa**	0.665794514	5.01604023210115e-08	L6a	LDR2h
**Hsp90ab1**	0.622854064	1.23431710685081e-07	L6a	LDR2h
**Nap1l5**	0.732175968	1.59825024679029e-07	L6a	LDR2h
**Cst3**	0.751049895	1.60536843268329e-07	L6a	LDR2h
**H3f3b**	0.709096315	2.09202334344161e-07	L6a	LDR2h
**Gm26652**	0.586823827	3.1307547630068e-07	L6a	LDR2h
**Calm1**	0.65776727	3.46494793021858e-07	L6a	LDR2h
**mt-Atp6**	1.096749354	5.93973534100464e-07	L6a	LDR2h

Development • Supplementary information

**Purb**	0.644786144	7.27279538180849e-07	L6a	LDR2h
**Tpt1**	0.692713097	8.72492046500013e-07	L6a	LDR2h
**Cox4i1**	0.778515261	1.20646830388925e-06	L6a	LDR2h
**Rpl6**	0.748605371	1.70896498632306e-06	L6a	LDR2h
**Cnot3**	0.611680684	3.04619334425109e-06	L6a	LDR2h
**Eef1a1**	0.605448721	6.96478611659035e-06	L6a	LDR2h
**Pabpc4**	0.65814108	9.51756020517664e-06	L6a	LDR2h
**Ctnna3**	0.959715324	1.0640137478778e-05	L6a	LDR2h
**Rpl38**	0.605099733	1.13584088061845e-05	L6a	LDR2h
**Cox8a**	0.71213624	1.26943119680128e-05	L6a	LDR2h
**Nt5dc3**	0.629103326	1.31160160140115e-05	L6a	LDR2h
**Arl5b**	0.585235541	2.58131842384015e-05	L6a	LDR2h
**Itgav**	0.708131	2.99471251200328e-05	L6a	LDR2h
**Tmem243**	0.692800912	4.10574627573112e-05	L6a	LDR2h
**Actb**	0.648148156	4.83797767290124e-05	L6a	LDR2h
**Gm14636**	0.694536487	4.86066701652477e-05	L6a	LDR2h
**Atp6v0b**	0.832165178	6.1903459695217e-05	L6a	LDR2h
**Vamp2**	0.618386703	6.59829115991033e-05	L6a	LDR2h
**Kcnk2**	0.890308769	7.96877556044996e-05	L6a	LDR2h
**Pnn**	0.657867427	8.77166150506448e-05	L6a	LDR2h
**Mapk6**	0.589785482	9.92439018211907e-05	L6a	LDR2h
**Brd9**	0.62524738	0.000124894	L6a	LDR2h
**Ywhag**	0.635407336	0.000144894	L6a	LDR2h
**Tle4**	0.721136903	0.000174569	L6a	LDR2h
**Sema3a**	0.889508671	0.000190548	L6a	LDR2h
**Snap25**	0.609051964	0.00033006	L6a	LDR2h
**Cox6c**	0.605826965	0.000389374	L6a	LDR2h
**R3hdm1**	0.646925539	0.000544666	L6a	LDR2h
**4930415C11Rik**	1.524111703	0.000819673	L6a	LDR2h
**Rgs20**	0.6628215	0.00089732	L6a	LDR2h
**Mir670hg**	1.32229798	0.00095755	L6a	LDR2h
**Rpl13**	0.607088285	0.001031081	L6a	LDR2h
**Diras2**	0.663866361	0.001291787	L6a	LDR2h
**Gadd45g**	0.867330132	0.001362029	L6a	LDR2h
**Dot1l**	0.73897422	0.001492427	L6a	LDR2h
**mt-Cytb**	1.172183911	0.001567022	L6a	LDR2h
**Epha10**	0.796284732	0.00170857	L6a	LDR2h
**Actn4**	0.630252228	0.001727945	L6a	LDR2h
**Cntn3**	0.69836167	0.001763459	L6a	LDR2h

**Arc**	1.277134925	0.001790786	L6a	LDR2h
**mt-Nd4**	0.667843983	0.003040487	L6a	LDR2h
**Oprm1**	0.839133509	0.003305068	L6a	LDR2h
**Hcn1**	0.835630562	0.004374284	L6a	LDR2h
**A230004M16Rik**	0.651376632	0.0058271	L6a	LDR2h
**1700054A03Rik**	0.778997251	0.024797165	L6a	LDR2h
**Ptchd4**	0.643956365	0.027104116	L6a	LDR2h
**Atf6**	0.611135388	0.027414491	L6a	LDR2h
**Ndfip2**	0.702376372	0.029047997	L6a	LDR2h
**Egr3**	2.337298399	1.06985452107313e-107	L6a	LDR30m
**Homer1**	2.833266062	1.76242004729293e-95	L6a	LDR30m
**Tiparp**	2.227848131	1.28186081297087e-84	L6a	LDR30m
**Hspa4**	1.954711672	2.34002964144483e-80	L6a	LDR30m
**Arl5b**	2.196400002	2.10631183558774e-76	L6a	LDR30m
**Pcsk1**	2.367474677	7.60102417938463e-75	L6a	LDR30m
**Zdbf2**	1.919375949	9.7533549921941e-67	L6a	LDR30m
**Fosb**	1.667097825	1.33491690698718e-65	L6a	LDR30m
**Gm47423**	2.124778798	1.21633160668946e-57	L6a	LDR30m
**Fosl2**	1.538674159	2.58373478773534e-51	L6a	LDR30m
**Ina**	1.756280317	1.84643333348863e-50	L6a	LDR30m
**Stk40**	1.419533413	5.2940337282604e-49	L6a	LDR30m
**Rheb**	1.658884388	6.87614800031084e-48	L6a	LDR30m
**Ankrd33b**	1.365378675	4.21356129426422e-45	L6a	LDR30m
**Npas4**	1.287227137	7.671501227881e-45	L6a	LDR30m
**Nr4a1**	1.365001888	1.92100136541873e-44	L6a	LDR30m
**Clstn3**	1.408954316	6.00784762117538e-44	L6a	LDR30m
**Sik2**	1.52518628	3.96815115536726e-43	L6a	LDR30m
**Chgb**	1.701214811	2.12785050381338e-41	L6a	LDR30m
**Cpeb3**	1.207544944	3.20760715887359e-41	L6a	LDR30m
**Med14**	1.469101372	5.04977800808342e-41	L6a	LDR30m
**Nr4a2**	2.722176586	9.98411389741844e-41	L6a	LDR30m
**Efhd2**	1.347632482	2.30427585050319e-40	L6a	LDR30m
**Frmd6**	1.388501684	2.53138758145044e-40	L6a	LDR30m
**Ube2ql1**	1.266100239	8.39177970550878e-38	L6a	LDR30m
**Per1**	1.471233134	3.01070729376068e-37	L6a	LDR30m
**Cltc**	1.238961365	8.74040633078613e-36	L6a	LDR30m
**Btaf1**	1.228000298	1.83650768951372e-33	L6a	LDR30m
**Baiap2**	1.094934708	6.73022919541599e-33	L6a	LDR30m
**Cwc25**	1.108066906	1.49133344952734e-32	L6a	LDR30m

Development • Supplementary information

**Gm17231**	1.341694043	7.17275890140454e-32	L6a	LDR30m
**Dusp14**	1.083009537	2.84162797144305e-31	L6a	LDR30m
**Mest**	1.087330771	3.92303132876828e-31	L6a	LDR30m
**1700016P03Rik**	3.065633647	1.31879137092413e-29	L6a	LDR30m
**Grasp**	1.030112796	1.6212670918898e-29	L6a	LDR30m
**Nrn1**	1.267204178	6.68759623747565e-29	L6a	LDR30m
**Rcc2**	0.985820292	1.21024709941501e-28	L6a	LDR30m
**Ctnnd1**	1.080019321	1.60201278359532e-28	L6a	LDR30m
**Arhgef3**	1.363036489	4.98391900884018e-28	L6a	LDR30m
**Kdm7a**	1.171728733	5.35752843688591e-28	L6a	LDR30m
**Kdm6b**	1.022609074	9.66097174446582e-28	L6a	LDR30m
**Trib1**	0.924820331	1.0457536061911e-27	L6a	LDR30m
**Coq10b**	1.141040319	2.10292833062675e-27	L6a	LDR30m
**Arid3b**	1.043119755	2.35579141625722e-27	L6a	LDR30m
**Gadd45b**	0.866648286	1.56836896978718e-26	L6a	LDR30m
**Hmgcr**	1.103231347	2.58751568957481e-26	L6a	LDR30m
**Ptprn**	1.139276081	2.27091254358753e-25	L6a	LDR30m
**Myh9**	0.992461159	1.01515598392987e-23	L6a	LDR30m
**Ciart**	0.865516092	1.48775699927645e-23	L6a	LDR30m
**Usp36**	1.025665403	1.50691100078925e-23	L6a	LDR30m
**Kcnk1**	0.93514129	1.57006197241542e-23	L6a	LDR30m
**Arpc2**	1.119191869	2.50357946227307e-23	L6a	LDR30m
**Nmnat2**	1.187538243	4.59576710856992e-23	L6a	LDR30m
**Hsph1**	0.896042568	2.85433232942119e-22	L6a	LDR30m
**Irs2**	0.955552446	3.88809475140725e-22	L6a	LDR30m
**Ece1**	1.241215752	4.34925910721261e-22	L6a	LDR30m
**Tmem178**	0.975139669	4.55547624250677e-22	L6a	LDR30m
**Nudt4**	0.96198015	4.87682103765185e-22	L6a	LDR30m
**Eprs**	0.980560416	9.33359672477859e-22	L6a	LDR30m
**Mn1**	0.939856955	9.49096596209561e-22	L6a	LDR30m
**Nrd1**	1.206571352	4.47200654548258e-21	L6a	LDR30m
**Rims4**	1.212917927	7.96695764880365e-21	L6a	LDR30m
**Ntrk2**	1.011805014	1.15364670107477e-20	L6a	LDR30m
**Fbl**	1.068608174	1.42676771365963e-20	L6a	LDR30m
**Trim9**	0.854504174	3.01121098331567e-20	L6a	LDR30m
**Gm17501**	0.843291605	5.90053835218466e-20	L6a	LDR30m
**Cpeb4**	1.002067363	7.62739514963379e-20	L6a	LDR30m
**Nap1l1**	0.864846973	9.80727538141948e-20	L6a	LDR30m
**Rcan2**	1.176822863	1.33101342413321e-19	L6a	LDR30m

Development • Supplementary information

**Pitpna**	0.852672852	2.77443506924113e-19	L6a	LDR30m
**Egr4**	0.688590393	6.04364258565512e-19	L6a	LDR30m
**Stat3**	0.870179311	6.1840796238146e-19	L6a	LDR30m
**Slc25a25**	0.983078474	7.87081362021008e-19	L6a	LDR30m
**Tulp4**	0.899653561	8.19114872366807e-19	L6a	LDR30m
**Etv5**	1.020609233	1.16227115197271e-18	L6a	LDR30m
**Emd**	0.687399937	2.25008780042465e-18	L6a	LDR30m
**Adora1**	0.924623334	5.51581712410794e-18	L6a	LDR30m
**Gm10563**	0.776127928	5.6370045768406e-18	L6a	LDR30m
**Fbxo33**	0.974643771	8.84414793403588e-18	L6a	LDR30m
**Spen**	0.833828409	1.28686341469282e-17	L6a	LDR30m
**Ap2b1**	0.816754386	1.38138381179397e-17	L6a	LDR30m
**Errfi1**	0.933067405	2.04680218139863e-17	L6a	LDR30m
**Cabp1**	0.921273217	2.09992996239632e-17	L6a	LDR30m
**Rnf217**	1.034584592	3.76495469040731e-17	L6a	LDR30m
**Smarca5**	0.935901061	4.3345064857857e-17	L6a	LDR30m
**Mgrn1**	0.879663101	5.15548747452286e-17	L6a	LDR30m
**Gm28294**	0.700103243	5.68376704357258e-17	L6a	LDR30m
**Nr4a3**	2.908303167	7.23176967331269e-17	L6a	LDR30m
**Slc2a1**	0.685787229	1.06703543383771e-16	L6a	LDR30m
**Ivns1abp**	0.78025489	1.98686374564859e-16	L6a	LDR30m
**Midn**	0.764062093	2.05539798489031e-16	L6a	LDR30m
**Csnk1a1**	0.889404964	2.18595123364254e-16	L6a	LDR30m
**Hnrnpll**	1.161355283	2.18851496894782e-16	L6a	LDR30m
**Atp6v0d1**	0.834812075	2.3154432457803e-16	L6a	LDR30m
**Txndc11**	0.834046126	2.84302022903783e-16	L6a	LDR30m
**Ndel1**	0.823370343	3.25592315011808e-16	L6a	LDR30m
**Stx1b**	0.832836391	5.01789256277189e-16	L6a	LDR30m
**Spred2**	0.890650335	5.25415714345466e-16	L6a	LDR30m
**Hsd17b12**	0.781433687	8.86312656477365e-16	L6a	LDR30m
**Vmp1**	0.818598538	8.91138548378429e-16	L6a	LDR30m
**Skil**	0.800118753	2.09736515616809e-15	L6a	LDR30m
**R3hdm2**	0.756672066	4.09409465899355e-15	L6a	LDR30m
**Ppard**	0.778728	5.00975764427841e-15	L6a	LDR30m
**Slc6a17**	0.818768236	5.24940882244237e-15	L6a	LDR30m
**Acsl4**	0.808963934	5.98741507139575e-15	L6a	LDR30m
**Plk3**	0.660360776	7.4616696890913e-15	L6a	LDR30m
**Tbc1d9**	0.806410144	7.83889501811399e-15	L6a	LDR30m
**Rgs7bp**	0.818033148	1.01501735874665e-14	L6a	LDR30m

Development • Supplementary information

**Rell1**	0.959096926	1.08687411818861e-14	L6a	LDR30m
**Kif5c**	0.762305773	1.3289550415726e-14	L6a	LDR30m
**Spred1**	1.013663815	2.56804622425838e-14	L6a	LDR30m
**Zswim6**	1.086017958	2.88741311900122e-14	L6a	LDR30m
**Jdp2**	1.06198307	2.92478003625533e-14	L6a	LDR30m
**Wdr1**	0.766282745	3.67900132359109e-14	L6a	LDR30m
**Sik3**	1.008890263	4.38288345859256e-14	L6a	LDR30m
**Arih1**	0.940070666	5.01910992259453e-14	L6a	LDR30m
**Mia3**	0.719941141	6.45209228085872e-14	L6a	LDR30m
**Gng2**	0.750826028	9.04559781909341e-14	L6a	LDR30m
**Gm6225**	0.665205071	9.17425273183584e-14	L6a	LDR30m
**Nptx2**	0.597084785	2.30004896045353e-13	L6a	LDR30m
**Slc7a5**	0.650595895	2.52552726892078e-13	L6a	LDR30m
**Arih2**	0.748283241	2.5760837399641e-13	L6a	LDR30m
**Gclc**	0.774479749	3.17794784077826e-13	L6a	LDR30m
**Slc7a8**	0.710708903	5.50096277911732e-13	L6a	LDR30m
**Agap3**	0.886208019	5.5078693308777e-13	L6a	LDR30m
**Atxn7**	0.741942045	6.35929136032742e-13	L6a	LDR30m
**Kpna1**	0.797569056	6.64027769254064e-13	L6a	LDR30m
**Cx3cl1**	0.786759094	9.58732815579415e-13	L6a	LDR30m
**Tet3**	0.934616109	1.00343739839379e-12	L6a	LDR30m
**Siah2**	0.628900803	1.47542061288472e-12	L6a	LDR30m
**Ccnl1**	0.785079489	1.68504626282229e-12	L6a	LDR30m
**Sez6l2**	0.772596203	2.70492084975455e-12	L6a	LDR30m
**Gnai3**	0.673914633	3.1607420625801e-12	L6a	LDR30m
**Mbp**	0.884053996	3.49617690745719e-12	L6a	LDR30m
**Ago3**	0.935854481	4.83016866534773e-12	L6a	LDR30m
**Smg7**	0.754871866	7.06396351061098e-12	L6a	LDR30m
**Elovl5**	0.651190802	8.9819358484001e-12	L6a	LDR30m
**Iqgap1**	0.699947095	1.15547721603585e-11	L6a	LDR30m
**Cacng3**	0.838212976	1.60419804292438e-11	L6a	LDR30m
**Bicdl1**	0.700589206	1.79592489240527e-11	L6a	LDR30m
**Cry2**	0.834436943	2.10669254648762e-11	L6a	LDR30m
**Cdc42ep3**	0.604122727	2.32894286037477e-11	L6a	LDR30m
**Gak**	0.774181609	2.7452750730687e-11	L6a	LDR30m
**Slc25a3**	0.985795838	3.34234657099384e-11	L6a	LDR30m
**Gabbr1**	0.779376848	4.11071991457578e-11	L6a	LDR30m
**Psd3**	0.979655197	4.31343499605952e-11	L6a	LDR30m
**Ppp1cc**	0.666035048	5.08240093520038e-11	L6a	LDR30m

Development • Supplementary information

**Dusp1**	0.638291324	6.39126456097744e-11	L6a	LDR30m
**Zbtb11**	0.762929655	6.64830909844155e-11	L6a	LDR30m
**E330009J07Rik**	0.677371869	1.23681620924199e-10	L6a	LDR30m
**Lncpint**	0.790410079	2.46504726274605e-10	L6a	LDR30m
**Ttbk1**	0.708277345	2.52766539686316e-10	L6a	LDR30m
**Per2**	0.723750535	3.06416355345646e-10	L6a	LDR30m
**Arf4**	0.696147729	3.16699404827147e-10	L6a	LDR30m
**Bdnf**	0.648348999	3.28729639936912e-10	L6a	LDR30m
**P4ha1**	0.79453965	4.02452426463122e-10	L6a	LDR30m
**Mir670hg**	0.830924105	4.87476165268195e-10	L6a	LDR30m
**Dcun1d3**	0.670675008	5.62676365419397e-10	L6a	LDR30m
**Gmeb2**	0.616066457	6.02372261153069e-10	L6a	LDR30m
**Cbarp**	0.781003289	7.88576486672671e-10	L6a	LDR30m
**Cdk11b**	0.677860049	7.88663644128281e-10	L6a	LDR30m
**Crem**	0.674312524	8.78283828816783e-10	L6a	LDR30m
**Pdlim1**	0.777231841	1.01835179561072e-09	L6a	LDR30m
**Mxi1**	0.731590654	1.06464908779757e-09	L6a	LDR30m
**Tacc1**	0.669374916	1.07473567928772e-09	L6a	LDR30m
**Abhd2**	0.792354014	1.09711595839501e-09	L6a	LDR30m
**Mast3**	0.71455564	1.36134456104947e-09	L6a	LDR30m
**Nup98**	0.64461917	1.51612868770053e-09	L6a	LDR30m
**Plat**	0.686036913	1.96837127242678e-09	L6a	LDR30m
**St8sia5**	0.643963166	2.00129324648302e-09	L6a	LDR30m
**Tbc1d1**	0.759523618	2.29712129910512e-09	L6a	LDR30m
**4921511C10Rik**	0.597064477	2.46980661645128e-09	L6a	LDR30m
**Atp1a1**	0.855890118	2.62130196159957e-09	L6a	LDR30m
**Dnajc1**	0.893896278	4.06526476238424e-09	L6a	LDR30m
**Pgm2**	0.672502862	4.35426833833701e-09	L6a	LDR30m
**Pik3r3**	0.687881014	4.555344237586e-09	L6a	LDR30m
**Cyp51**	0.62497981	5.02606040397752e-09	L6a	LDR30m
**Sec14l1**	0.732451405	5.30601366990616e-09	L6a	LDR30m
**Pmepa1**	0.819953611	5.6782258631104e-09	L6a	LDR30m
**Fkbp1a**	0.732714298	7.44110205583538e-09	L6a	LDR30m
**Dnajb5**	0.702793273	9.39287571186912e-09	L6a	LDR30m
**Foxo3**	0.771532322	9.68507079731789e-09	L6a	LDR30m
**Tpm3**	0.628938397	1.1568867427181e-08	L6a	LDR30m
**Zhx2**	0.771929002	1.31866121719838e-08	L6a	LDR30m
**Spag9**	0.650059707	1.5003004756807e-08	L6a	LDR30m
**Zfp948**	0.710071826	1.56436293406783e-08	L6a	LDR30m

**Dnaja1**	0.586503163	2.14333708719727e-08	L6a	LDR30m
**Rundc1**	0.597669254	2.46902693509075e-08	L6a	LDR30m
**Atp2a2**	0.597760966	3.23979419526756e-08	L6a	LDR30m
**Mpp2**	0.632741371	4.15539578722334e-08	L6a	LDR30m
**Mamld1**	0.630208308	4.21642208988809e-08	L6a	LDR30m
**Slc2a3**	0.66189967	4.76205229501052e-08	L6a	LDR30m
**Acss1**	0.657884515	5.40993480467728e-08	L6a	LDR30m
**Rab6a**	0.900303265	5.70633994643514e-08	L6a	LDR30m
**Ifrd1**	0.642577718	5.77832310622851e-08	L6a	LDR30m
**Kras**	0.63236362	5.93807367001945e-08	L6a	LDR30m
**Ski**	0.874454022	7.07022326636082e-08	L6a	LDR30m
**Hipk3**	0.620306563	8.32689462824717e-08	L6a	LDR30m
**Phyhipl**	0.604938639	8.40967318919185e-08	L6a	LDR30m
**Dpy19l3**	0.650738489	1.26421267772156e-07	L6a	LDR30m
**Klf9**	0.62890072	1.32625323769429e-07	L6a	LDR30m
**Mbnl1**	0.67864095	1.51422047286526e-07	L6a	LDR30m
**Unc45a**	0.629847113	2.18986414906269e-07	L6a	LDR30m
**Ranbp2**	0.909650677	3.52362446422895e-07	L6a	LDR30m
**Sptbn2**	0.658214086	4.15097838681373e-07	L6a	LDR30m
**Usp9x**	0.649005348	4.27431470097062e-07	L6a	LDR30m
**Rab6b**	0.736346277	4.47683405752741e-07	L6a	LDR30m
**Ubl3**	0.638583369	5.18336956754656e-07	L6a	LDR30m
**Pip5k1a**	0.605616469	5.4175467969816e-07	L6a	LDR30m
**Grm4**	0.674413781	6.25470305415739e-07	L6a	LDR30m
**Epha10**	0.637047246	8.13883279923561e-07	L6a	LDR30m
**Sgsm1**	0.84088988	8.91772800564061e-07	L6a	LDR30m
**Mef2d**	0.586879534	9.67064440574118e-07	L6a	LDR30m
**BC005537**	0.630805874	1.34009922349266e-06	L6a	LDR30m
**Efr3b**	0.686677955	1.8683011013037e-06	L6a	LDR30m
**Slc12a2**	0.607466943	2.29389930203716e-06	L6a	LDR30m
**Trak1**	0.722817147	2.34584511752573e-06	L6a	LDR30m
**Ubtd2**	0.640973732	2.42595576641217e-06	L6a	LDR30m
**Syt4**	0.686956611	3.13344115351635e-06	L6a	LDR30m
**Fbrsl1**	0.587159913	3.45542042666248e-06	L6a	LDR30m
**Arid5b**	0.637942843	4.1452008835535e-06	L6a	LDR30m
**Ddx3y**	0.666673694	6.34167562520727e-06	L6a	LDR30m
**Vps37b**	0.607328264	9.92050131556363e-06	L6a	LDR30m
**Gm48747**	0.723208483	1.83363877763802e-05	L6a	LDR30m
**Sdcbp**	0.62217131	1.86657306649398e-05	L6a	LDR30m

**Ptpn12**	0.5989849	2.25812079709084e-05	L6a	LDR30m
**Cmip**	0.73880639	3.98312059859203e-05	L6a	LDR30m
**Rock2**	0.695514928	4.70430294210778e-05	L6a	LDR30m
**Ndfip2**	1.536766108	5.7673790371554e-05	L6a	LDR30m
**Fam131a**	0.669575392	6.61960084300457e-05	L6a	LDR30m
**Csnk1d**	0.61904045	0.000181444	L6a	LDR30m
**Sik1**	0.834295236	0.000196366	L6a	LDR30m
**Tnfrsf21**	0.617656842	0.000236176	L6a	LDR30m
**1700110K17Rik**	0.635828895	0.000248984	L6a	LDR30m
**Ywhag**	0.601987611	0.00025701	L6a	LDR30m
**Pde4a**	0.64237116	0.000258804	L6a	LDR30m
**Hdac5**	0.618200248	0.000338427	L6a	LDR30m
**Dok5**	0.658707755	0.000523296	L6a	LDR30m
**Snap25**	0.627910169	0.000654755	L6a	LDR30m
**Brinp1**	0.610254497	0.001455929	L6a	LDR30m
**Ccn1**	1.179403349	0.001568903	L6a	LDR30m
**Osbpl8**	0.603418755	0.001794612	L6a	LDR30m
**Sept7**	0.597384473	0.002176756	L6a	LDR30m
**Gfod1**	0.596202965	0.015979734	L6a	LDR30m
**Hs3st2**	0.664934086	0.027622081	L6a	LDR30m
**Homer1**	1.995007013	2.2737954948317e-93	L6a	LDR4h
**Arhgap31**	1.65508221	7.90142981996944e-70	L6a	LDR4h
**Gfra1**	1.275373627	7.91857049246406e-46	L6a	LDR4h
**Tet3**	1.332497589	1.98068373348119e-38	L6a	LDR4h
**Pcsk1**	1.243666143	5.65976110259599e-38	L6a	LDR4h
**Epha10**	1.200764928	4.05132262463509e-35	L6a	LDR4h
**Mir670hg**	1.173746597	7.58105490734449e-33	L6a	LDR4h
**Tmem178**	1.050918589	4.55250135848632e-32	L6a	LDR4h
**Tiparp**	0.915711161	1.7441782972503e-30	L6a	LDR4h
**Egr3**	1.064662216	2.10763323901361e-29	L6a	LDR4h
**Sgsm1**	1.601280515	5.36244712376477e-29	L6a	LDR4h
**Ddah1**	1.07188091	2.5081970676585e-28	L6a	LDR4h
**Ntrk2**	1.085184441	1.62012718935287e-27	L6a	LDR4h
**Trim9**	0.918207872	7.39820465694726e-26	L6a	LDR4h
**Mapk4**	1.35219605	8.27139945434751e-26	L6a	LDR4h
**Spred2**	1.15750842	4.65629986696167e-25	L6a	LDR4h
**Baz1a**	0.879541711	1.57624142816831e-24	L6a	LDR4h
**Nrn1**	1.112464373	3.81561164008757e-24	L6a	LDR4h
**R3hdm1**	0.8290384	4.44842625895709e-24	L6a	LDR4h

**Vmp1**	0.878754956	1.79118732160508e-23	L6a	LDR4h
**Jdp2**	1.053939886	7.04558591797082e-23	L6a	LDR4h
**Spred1**	0.948149021	2.79854976176491e-21	L6a	LDR4h
**Car10**	0.920875611	3.3517970435735e-21	L6a	LDR4h
**Actn4**	0.942327851	4.33329190881189e-21	L6a	LDR4h
**Rcan2**	1.057898185	2.88727400668967e-20	L6a	LDR4h
**Nptx2**	0.75689738	3.22026350663086e-20	L6a	LDR4h
**Itgav**	0.811597315	5.32866087894083e-20	L6a	LDR4h
**Brinp1**	0.932465063	8.11511257563069e-20	L6a	LDR4h
**Rgs20**	1.027921628	4.44411862189679e-19	L6a	LDR4h
**Grasp**	0.73169916	6.89498971679091e-19	L6a	LDR4h
**Dnajc1**	0.948939431	1.66149179767875e-18	L6a	LDR4h
**Fmnl1**	0.897645111	2.78638663391563e-17	L6a	LDR4h
**Mamld1**	0.75304208	4.42623582519405e-17	L6a	LDR4h
**Gmeb2**	0.734176509	6.53727811185373e-17	L6a	LDR4h
**Ppm1h**	1.013273353	8.232902626521e-16	L6a	LDR4h
**Ppme1**	0.695284697	8.66523114881694e-16	L6a	LDR4h
**Nrp1**	0.890034118	8.87046981590193e-16	L6a	LDR4h
**Dot1l**	0.707265091	1.0214949885444e-15	L6a	LDR4h
**Stk40**	0.864749167	1.42390591062628e-15	L6a	LDR4h
**Phf21b**	0.744915869	1.62121900413203e-15	L6a	LDR4h
**Eml5**	0.972364028	2.66753387066617e-15	L6a	LDR4h
**Slc6a17**	0.711521725	3.37533240338448e-15	L6a	LDR4h
**Gm32647**	0.869681092	5.20824020220297e-15	L6a	LDR4h
**Sik2**	1.05597607	8.58694932611466e-15	L6a	LDR4h
**Kdm7a**	0.728520689	9.15238912863111e-15	L6a	LDR4h
**Ago3**	0.653215326	1.07614652610247e-14	L6a	LDR4h
**Zmiz1**	1.146091251	1.20594225250736e-14	L6a	LDR4h
**Pak6**	0.701057895	2.96065735073118e-14	L6a	LDR4h
**Nrxn2**	0.751366018	4.28154300962181e-14	L6a	LDR4h
**Slc9a5**	0.6608965	5.0823168900042e-14	L6a	LDR4h
**Gm17501**	0.654207361	7.83276562069584e-14	L6a	LDR4h
**1700016P03Rik**	2.099648084	8.76994309579759e-14	L6a	LDR4h
**Grin2a**	0.747821605	1.54798700532539e-13	L6a	LDR4h
**Rps6ka3**	0.740885676	1.97067495267006e-13	L6a	LDR4h
**Cap2**	0.67106331	5.5984179304087e-13	L6a	LDR4h
**Kdm6b**	0.683171213	1.0483706774019e-12	L6a	LDR4h
**Clstn3**	0.922267549	1.37172235813336e-12	L6a	LDR4h
**Arid3b**	0.768589321	1.65697817149209e-12	L6a	LDR4h

**Mapk6**	0.707275455	3.94579461517381e-12	L6a	LDR4h
**Zdbf2**	0.888916133	8.00071696527173e-12	L6a	LDR4h
**P4ha1**	0.705273629	9.33921641229242e-12	L6a	LDR4h
**Osbpl8**	0.665474555	1.0556237700031e-11	L6a	LDR4h
**Jmjd1c**	0.587086139	1.14275113206143e-11	L6a	LDR4h
**Stx4a**	0.595357904	1.61122124447826e-11	L6a	LDR4h
**Efr3a**	0.766251983	2.37015419570616e-11	L6a	LDR4h
**Hnrnpll**	0.601512484	4.1373324937332e-11	L6a	LDR4h
**Klhl2**	0.623028805	4.26755522479978e-11	L6a	LDR4h
**Sik3**	0.901472205	4.63928437738454e-11	L6a	LDR4h
**Sema3e**	0.967240386	4.78712228538484e-11	L6a	LDR4h
**Nrd1**	0.713972697	1.02537292659485e-10	L6a	LDR4h
**Rheb**	0.718836278	3.1036309133805e-10	L6a	LDR4h
**Scube1**	0.711622489	4.87037471562489e-10	L6a	LDR4h
**Grb2**	0.690004901	5.55457392784861e-10	L6a	LDR4h
**Med14**	0.652748805	8.11088350112937e-10	L6a	LDR4h
**Hectd2**	0.708353822	9.48942443762206e-10	L6a	LDR4h
**Gm4128**	0.663075438	1.0824503736022e-09	L6a	LDR4h
**Psd3**	0.605197606	1.14843656063553e-09	L6a	LDR4h
**Ppard**	0.731730999	1.19560246294158e-09	L6a	LDR4h
**Ece1**	0.851016539	3.88292323313269e-09	L6a	LDR4h
**Sorcs1**	0.671032113	4.29089770237653e-09	L6a	LDR4h
**Arhgef7**	0.720495383	6.56114598324468e-09	L6a	LDR4h
**Ndfip2**	0.780501213	1.10639162392349e-08	L6a	LDR4h
**Hs3st2**	0.730588426	1.5496177797849e-08	L6a	LDR4h
**Gabra2**	0.638386804	1.76286071778532e-08	L6a	LDR4h
**Rnd3**	0.653735989	3.29790841499345e-08	L6a	LDR4h
**Nmnat2**	0.755477905	3.48721111689891e-08	L6a	LDR4h
**Nr4a1**	0.715958021	4.04883986693453e-08	L6a	LDR4h
**Mast3**	0.748661266	5.32634185892184e-08	L6a	LDR4h
**Gm46367**	0.686199885	6.72766245887705e-08	L6a	LDR4h
**Ankrd33b**	0.749152925	6.84333635661384e-08	L6a	LDR4h
**Ptprn**	0.868036559	1.09890268844549e-07	L6a	LDR4h
**Fkbp1a**	0.662944805	3.37218440185179e-07	L6a	LDR4h
**Dok5**	0.590768329	3.7957543491755e-07	L6a	LDR4h
**Frmd6**	0.74021195	7.09369032164295e-07	L6a	LDR4h
**Myh9**	0.621829445	2.27054248881275e-06	L6a	LDR4h
**Zbtb16**	0.654983984	2.47997203916316e-06	L6a	LDR4h
**Zswim6**	0.717822672	3.19189265921984e-06	L6a	LDR4h

Development • Supplementary information

**Gfod1**	0.666619965	6.32117971956174e-06	L6a	LDR4h
**Pdlim1**	0.602149041	2.89440668064118e-05	L6a	LDR4h
**Slc2a13**	0.616727955	3.40163211162923e-05	L6a	LDR4h
**Per1**	0.59405039	7.28484111584912e-05	L6a	LDR4h
**Cmip**	0.602394339	0.012015981	L6a	LDR4h
**Fth1**	1.328194401	6.64790233864865e-36	L6a	LDR6h
**mt-Co1**	1.510470015	1.89652655913266e-32	L6a	LDR6h
**Apoe**	1.015349051	2.27622361748497e-30	L6a	LDR6h
**mt-Co3**	1.39871008	4.13463336558545e-28	L6a	LDR6h
**Sema3c**	0.89650087	3.62410212123673e-23	L6a	LDR6h
**Srsf7**	1.118288745	3.24684527437222e-16	L6a	LDR6h
**Gm27032**	1.081938866	8.52775504860944e-16	L6a	LDR6h
**Gm28376**	1.026300057	2.81073944331127e-15	L6a	LDR6h
**mt-Co2**	1.168201407	2.98379585283525e-15	L6a	LDR6h
**mt-Cytb**	1.088359401	1.65841846091293e-14	L6a	LDR6h
**Cst3**	0.769779214	1.77890464472341e-14	L6a	LDR6h
**Tmsb4x**	0.866243172	3.31127688010896e-14	L6a	LDR6h
**Eif1**	0.812817148	2.47130571942008e-12	L6a	LDR6h
**mt-Nd1**	0.953100031	4.98835331754195e-12	L6a	LDR6h
**B230217C12Rik**	0.634467779	5.35931094448057e-12	L6a	LDR6h
**H3f3b**	0.867359659	1.12955550853353e-11	L6a	LDR6h
**Cox8a**	0.779650028	1.80846180289801e-11	L6a	LDR6h
**Ubb**	0.884316287	1.23891751200413e-10	L6a	LDR6h
**Cox4i1**	0.734691146	2.07532248093426e-10	L6a	LDR6h
**Rpl41**	0.738870457	5.53963039091337e-10	L6a	LDR6h
**Atp5a1**	0.735598075	7.76757528297297e-10	L6a	LDR6h
**Rpl6**	0.847024082	9.40151018812528e-10	L6a	LDR6h
**Rap2b**	0.689196716	3.58674877862997e-09	L6a	LDR6h
**Ndufb9**	0.597573058	1.2917669498565e-08	L6a	LDR6h
**Cck**	0.601505791	2.10436048404746e-08	L6a	LDR6h
**Gm46367**	0.804865457	5.49454435751685e-08	L6a	LDR6h
**Rprml**	0.822584794	6.20282604642169e-08	L6a	LDR6h
**Nrgn**	0.710705976	8.17416677571e-08	L6a	LDR6h
**Tigd2**	0.594948233	1.82521953265142e-07	L6a	LDR6h
**Rps21**	0.702230456	2.63851671470339e-07	L6a	LDR6h
**Rpl13**	0.665047289	2.7451023224107e-07	L6a	LDR6h
**Ppia**	0.682491066	5.2570326792575e-07	L6a	LDR6h
**Dnaja2**	0.718821724	8.10645099082055e-07	L6a	LDR6h
**Slc50a1**	0.751770922	1.0243640588158e-06	L6a	LDR6h

Development • Supplementary information

**Slc25a4**	0.618887313	1.31545006206049e-06	L6a	LDR6h
**Hagh**	0.618621111	1.45809722484697e-06	L6a	LDR6h
**B3galt2**	0.630297156	1.74400433551904e-06	L6a	LDR6h
**mt-Atp6**	0.93175771	2.59333687231299e-06	L6a	LDR6h
**Gm32250**	0.666669599	6.26856936127952e-06	L6a	LDR6h
**Bmyc**	0.598407374	1.02940145979898e-05	L6a	LDR6h
**Calm1**	0.633676815	1.05067825075092e-05	L6a	LDR6h
**Cox6c**	0.594259129	1.21657487371235e-05	L6a	LDR6h
**Arpp19**	0.615307695	1.24017833032827e-05	L6a	LDR6h
**Ndufaf7**	0.637284868	1.25733275778762e-05	L6a	LDR6h
**Tmem208**	0.615289719	1.43118030146238e-05	L6a	LDR6h
**Cox7b**	0.689195392	1.92847626134467e-05	L6a	LDR6h
**Ckb**	0.678931341	2.93449064098287e-05	L6a	LDR6h
**Prelid3a**	0.6405935	4.99462535435053e-05	L6a	LDR6h
**Tmem243**	0.635269231	6.75202409375039e-05	L6a	LDR6h
**Oaz1**	0.599049742	6.75596289125012e-05	L6a	LDR6h
**Rpl9**	0.593246167	7.03811343490283e-05	L6a	LDR6h
**Atp6v0b**	0.855491741	8.23122507838692e-05	L6a	LDR6h
**Tpt1**	0.589709487	9.85000211584221e-05	L6a	LDR6h
**Rmdn1**	0.634906708	0.000109079	L6a	LDR6h
**Prpf4b**	0.728002284	0.000115084	L6a	LDR6h
**Tmed9**	0.586376937	0.000115314	L6a	LDR6h
**Trim35**	0.709819748	0.000125345	L6a	LDR6h
**6430590A07Rik**	0.644477997	0.000135649	L6a	LDR6h
**1110008P14Rik**	0.600049213	0.000139329	L6a	LDR6h
**5330438D12Rik**	0.7412841	0.000144392	L6a	LDR6h
**Arf5**	0.59508901	0.000161323	L6a	LDR6h
**Atpif1**	0.601993766	0.000290051	L6a	LDR6h
**Nap1l5**	0.608082563	0.000476412	L6a	LDR6h
**Slc25a3**	0.669058392	0.00047805	L6a	LDR6h
**Dynll1**	0.593863739	0.000523094	L6a	LDR6h
**Dalrd3**	0.604019827	0.000561552	L6a	LDR6h
**Emc3**	0.591732637	0.000702972	L6a	LDR6h
**Pnn**	0.667216733	0.000985972	L6a	LDR6h
**Malat1**	1.288981442	0.001541011	L6a	LDR6h
**1700019D03Rik**	0.586168503	0.00164784	L6a	LDR6h
**Ndfip2**	0.668980474	0.001771348	L6a	LDR6h
**Mdh1**	0.594089881	0.001841782	L6a	LDR6h
**Eef1a1**	0.617663612	0.00222206	L6a	LDR6h

Development • Supplementary information

**Kcnk2**	0.813994155	0.003224431	L6a	LDR6h
**Gm43376**	0.679530764	0.007669693	L6a	LDR6h
**Gm36975**	0.656356442	0.010358986	L6a	LDR6h
**Rbm39**	0.590881143	0.016591719	L6a	LDR6h
**Ptgds**	0.701258702	0.018033907	L6a	LDR6h
**Nrg3os**	0.797257521	0.018575855	L6a	LDR6h
**Oprm1**	0.70001339	0.025875366	L6a	LDR6h
**Ndufaf4**	0.615445102	0.037348876	L6a	LDR6h
**Gm48678**	0.643387743	0.0383855	L6a	LDR6h
**AC149090.1**	1.599932841	6.34758608851115e-13	L6b	LDR2h
**Sema3c**	1.821005593	3.46463244052766e-06	L6b	LDR2h
**Maml3**	1.259906968	7.97110202790641e-06	L6b	LDR2h
**Ptprn**	1.04731176	0.000174364	L6b	LDR2h
**4930415C11Rik**	1.348295042	0.000318671	L6b	LDR2h
**Homer1**	0.928907099	0.001391646	L6b	LDR2h
**Cx3cl1**	1.205309613	0.004520607	L6b	LDR2h
**mt-Co3**	1.222511305	0.005842438	L6b	LDR2h
**Apoe**	1.30319581	0.012598559	L6b	LDR2h
**Hunk**	1.232789798	0.032532691	L6b	LDR2h
**Homer1**	2.717709797	8.09528698315578e-72	L6b	LDR30m
**1700016P03Rik**	2.565658628	7.32171158296314e-22	L6b	LDR30m
**Pcsk1**	2.057234059	3.26291977389814e-12	L6b	LDR30m
**Gm47423**	1.82541633	7.31826080195499e-10	L6b	LDR30m
**Ntrk2**	1.023903708	8.27117535445502e-10	L6b	LDR30m
**Tmem178**	1.24214908	1.7131218832632e-07	L6b	LDR30m
**Cpeb3**	1.112896197	3.3537563453859e-07	L6b	LDR30m
**Nr4a3**	2.782717816	5.43474227537522e-07	L6b	LDR30m
**Arhgef3**	1.265031711	2.83242860954775e-06	L6b	LDR30m
**Zdbf2**	1.745385423	6.18929521077673e-06	L6b	LDR30m
**Btaf1**	1.219435602	7.36393359728974e-06	L6b	LDR30m
**Rock2**	1.034869	1.30358141413669e-05	L6b	LDR30m
**Arl5b**	1.55419671	1.49278695670349e-05	L6b	LDR30m
**Nr4a2**	1.244537479	1.79372597708548e-05	L6b	LDR30m
**Hnrnpll**	1.281038788	2.51845031965255e-05	L6b	LDR30m
**Ube2ql1**	1.46183076	2.62026244729859e-05	L6b	LDR30m
**Egr3**	2.347415453	4.18992704103081e-05	L6b	LDR30m
**Nr4a1**	1.37689884	4.49409005800932e-05	L6b	LDR30m
**Ankrd33b**	1.300788316	7.33657416538955e-05	L6b	LDR30m
**Chst11**	1.105787784	9.61924477447764e-05	L6b	LDR30m

**Sv2c**	1.394183752	0.00028511	L6b	LDR30m
**Psd3**	0.938509351	0.000299831	L6b	LDR30m
**Tet3**	1.207035977	0.00061819	L6b	LDR30m
**Baiap2**	1.013429348	0.001684791	L6b	LDR30m
**Rheb**	1.172392979	0.001720945	L6b	LDR30m
**Nrn1**	1.295907069	0.003246186	L6b	LDR30m
**Gfod1**	0.943202806	0.003688874	L6b	LDR30m
**Pik3r3**	1.024663334	0.004562913	L6b	LDR30m
**Pdlim1**	1.213843005	0.008676504	L6b	LDR30m
**Pde10a**	0.745862114	0.009125075	L6b	LDR30m
**Stk40**	1.172786951	0.009778039	L6b	LDR30m
**Trim9**	0.812442744	0.010509782	L6b	LDR30m
**Kif5c**	0.902386577	0.012145999	L6b	LDR30m
**Ndfip2**	1.093360123	0.014606871	L6b	LDR30m
**Cpeb4**	0.933102902	0.015412723	L6b	LDR30m
**Coq10b**	1.254602296	0.01783007	L6b	LDR30m
**Rph3a**	1.099426364	0.022625053	L6b	LDR30m
**Nrd1**	0.958129703	0.030504527	L6b	LDR30m
**Slc25a25**	1.256361916	0.032090323	L6b	LDR30m
**Sik2**	0.944683584	0.037126946	L6b	LDR30m
**Homer1**	1.857461748	6.62453441162193e-28	L6b	LDR4h
**Tmem178**	1.344073006	3.47684237667852e-08	L6b	LDR4h
**Ntrk2**	0.752674362	1.14310229825296e-05	L6b	LDR4h
**Airn**	1.529554983	0.000126312	L6b	LDR4h
**1700016P03Rik**	1.435989542	0.00051812	L6b	LDR4h
**Gm15398**	1.227320835	0.001853472	L6b	LDR4h
**Grin2a**	0.771574046	0.006116988	L6b	LDR4h
**Gfod1**	0.855726753	0.023209007	L6b	LDR4h
**Pcdh15**	0.730807071	0.038355693	L6b	LDR4h
**Sgsm1**	0.947693075	0.047275511	L6b	LDR4h
**Malat1**	1.150821659	0.015025801	L6b	LDR6h
**mt-Co1**	1.222375652	0.023611879	L6b	LDR6h
**C1ql3**	1.603290193	2.21109997516504e-35	L6IT	LDR2h
**Nrn1**	1.50501523	1.23438574578744e-34	L6IT	LDR2h
**1700016P03Rik**	1.318572447	1.4815412575821e-27	L6IT	LDR2h
**Adgrd1**	1.278615477	2.90731226229125e-25	L6IT	LDR2h
**4933413L06Rik**	1.087632187	3.35732000809653e-22	L6IT	LDR2h
**Nptx2**	1.127993616	4.01629129450984e-22	L6IT	LDR2h
**Ptprn**	1.123806152	1.93123125700227e-19	L6IT	LDR2h

Development • Supplementary information

**Bdnf**	1.16632385	4.98644893022394e-19	L6IT	LDR2h
**Gm37229**	0.929314232	6.6534445554117e-17	L6IT	LDR2h
**Mir670hg**	1.255371624	8.73619190483412e-17	L6IT	LDR2h
**Fosl2**	0.915211058	3.89748396219418e-16	L6IT	LDR2h
**mt-Co3**	1.417097225	9.83251549176329e-16	L6IT	LDR2h
**Inhba**	1.008552796	1.23051643953831e-15	L6IT	LDR2h
**mt-Co1**	1.391561379	8.06843594491718e-15	L6IT	LDR2h
**Ppme1**	1.176051613	4.53340485119905e-14	L6IT	LDR2h
**Npas4**	0.86592497	2.04408901983368e-13	L6IT	LDR2h
**Car12**	0.901697505	2.16955825426312e-13	L6IT	LDR2h
**mt-Nd1**	1.042979317	4.89312571991483e-13	L6IT	LDR2h
**Ptgds**	0.817335223	5.69780640793072e-13	L6IT	LDR2h
**Gm15261**	0.826061156	2.266365243383e-12	L6IT	LDR2h
**Scg2**	0.992591988	1.0079700653737e-11	L6IT	LDR2h
**Arc**	1.005979123	2.14938192332937e-11	L6IT	LDR2h
**1700054A03Rik**	0.8218999	5.1603647597267e-11	L6IT	LDR2h
**Pabpc4**	0.854358824	7.46047754305182e-11	L6IT	LDR2h
**Cdh9**	1.123189449	8.46211646845527e-11	L6IT	LDR2h
**Grasp**	0.86642779	1.14134042716448e-10	L6IT	LDR2h
**Egr3**	0.766970546	1.16518020629239e-10	L6IT	LDR2h
**Gadd45g**	0.682840404	1.56320762883961e-10	L6IT	LDR2h
**Cap1**	0.807602743	2.44441698725727e-10	L6IT	LDR2h
**Gm49127**	0.88709783	5.42025932810808e-10	L6IT	LDR2h
**Baz1a**	0.681303077	6.04971503266183e-10	L6IT	LDR2h
**Cx3cl1**	0.721288924	1.12646435717473e-08	L6IT	LDR2h
**Oprm1**	0.957945112	1.90653387673049e-08	L6IT	LDR2h
**Slc25a3**	0.899808671	2.00760000463882e-08	L6IT	LDR2h
**Brinp1**	0.99183555	7.58642996371089e-07	L6IT	LDR2h
**Rps29**	0.709043104	7.61914373897664e-07	L6IT	LDR2h
**Tpt1**	0.65994678	1.27189663321098e-06	L6IT	LDR2h
**Gnal**	0.782313724	2.32705435623778e-06	L6IT	LDR2h
**Atp6v0b**	0.86431541	3.26214437314056e-06	L6IT	LDR2h
**Eef1a1**	0.678242303	3.78256250772929e-06	L6IT	LDR2h
**Rps16**	0.614919932	3.89860488904133e-06	L6IT	LDR2h
**Dalrd3**	0.700724438	5.32309899247347e-06	L6IT	LDR2h
**Mas1**	0.649078879	6.55638464639517e-06	L6IT	LDR2h
**Ctnna3**	0.990485795	6.89762871678764e-06	L6IT	LDR2h
**Purb**	0.652203692	7.49663959884733e-06	L6IT	LDR2h
**Gm14636**	0.707585299	1.56370480594442e-05	L6IT	LDR2h

Development • Supplementary information

**Aldoa**	0.693357153	1.56753502225222e-05	L6IT	LDR2h
**Nrep**	0.690425927	1.746155676885e-05	L6IT	LDR2h
**Fbxo33**	0.611243267	2.22787076653965e-05	L6IT	LDR2h
**Rpl38**	0.644970628	2.41324022351423e-05	L6IT	LDR2h
**Hspa8**	0.77046203	2.4968255375027e-05	L6IT	LDR2h
**Itgav**	0.832942073	3.25246705720345e-05	L6IT	LDR2h
**Rapgef5**	0.98109267	5.13565593943801e-05	L6IT	LDR2h
**Rps8**	0.620014417	5.71126197237628e-05	L6IT	LDR2h
**Gm13684**	0.59506103	6.37642906803319e-05	L6IT	LDR2h
**Arhgap6**	0.644560642	6.70796307544058e-05	L6IT	LDR2h
**Calm1**	0.620677426	9.45954705247624e-05	L6IT	LDR2h
**Hcn1**	1.046215974	9.53550455867158e-05	L6IT	LDR2h
**Jcad**	0.669761907	0.000107319	L6IT	LDR2h
**Diras2**	0.761248861	0.000111621	L6IT	LDR2h
**Gm4128**	0.697769414	0.000122474	L6IT	LDR2h
**Ppp1r1a**	0.703305759	0.000152367	L6IT	LDR2h
**Nr4a3**	0.639741189	0.000176262	L6IT	LDR2h
**Arhgap31**	0.79306201	0.000223545	L6IT	LDR2h
**Gm15520**	0.680547493	0.000329642	L6IT	LDR2h
**Gm6994**	0.691188195	0.000372894	L6IT	LDR2h
**Rpl6**	0.757141012	0.000414295	L6IT	LDR2h
**Pdp1**	0.594851618	0.000473525	L6IT	LDR2h
**Plk2**	0.649748591	0.000603414	L6IT	LDR2h
**Ifrd1**	0.594636566	0.000735051	L6IT	LDR2h
**Gm21798**	0.616173395	0.000879665	L6IT	LDR2h
**Daglb**	0.669062585	0.000903723	L6IT	LDR2h
**Plxna4os3**	0.638197016	0.001097576	L6IT	LDR2h
**Ltbp1**	0.648787302	0.001117131	L6IT	LDR2h
**Klhl2**	0.685437117	0.00137486	L6IT	LDR2h
**Cox8a**	0.684956948	0.002953263	L6IT	LDR2h
**Cox6c**	0.632773821	0.002988465	L6IT	LDR2h
**Crebzf**	0.591410492	0.003112119	L6IT	LDR2h
**Apoe**	1.059019032	0.003198976	L6IT	LDR2h
**1700085D07Rik**	0.723018796	0.005580892	L6IT	LDR2h
**Ier5**	0.59249561	0.005711685	L6IT	LDR2h
**Cox4i1**	0.65320246	0.006042991	L6IT	LDR2h
**Sema3c**	1.455791109	0.006166854	L6IT	LDR2h
**Gm15398**	0.86283839	0.006779002	L6IT	LDR2h
**Eif1**	0.640836499	0.007261805	L6IT	LDR2h

**mt-Co2**	1.307342001	0.008708888	L6IT	LDR2h
**Phf21b**	0.618057897	0.008769448	L6IT	LDR2h
**Gm34544**	1.099404864	0.011044149	L6IT	LDR2h
**Grb2**	0.682890732	0.014175959	L6IT	LDR2h
**Fth1**	1.326387874	0.017715283	L6IT	LDR2h
**4930415C11Rik**	1.446103995	0.019175546	L6IT	LDR2h
**Sema3a**	0.623289645	0.041467335	L6IT	LDR2h
**Sema3e**	0.731497959	0.043349778	L6IT	LDR2h
**Pcsk1n**	0.594729919	0.045139557	L6IT	LDR2h
**1700016P03Rik**	2.906200876	2.12702609951609e-131	L6IT	LDR30m
**Nr4a3**	2.725909639	1.13696814966666e-92	L6IT	LDR30m
**Egr3**	2.150807867	2.07004972589916e-77	L6IT	LDR30m
**Sik2**	1.687353458	9.10260013196671e-64	L6IT	LDR30m
**Ube2ql1**	1.634485418	2.81419688116293e-51	L6IT	LDR30m
**Arl5b**	1.832914344	2.87874646558597e-46	L6IT	LDR30m
**Fosl2**	1.686436983	9.55034735155943e-46	L6IT	LDR30m
**Zdbf2**	1.70891384	1.11402708212979e-44	L6IT	LDR30m
**Cpeb3**	1.277554803	3.02432830137856e-42	L6IT	LDR30m
**Arhgef3**	1.615610327	1.4233930456307e-40	L6IT	LDR30m
**Frmd6**	1.311130646	2.27250510920177e-40	L6IT	LDR30m
**Rheb**	1.453965709	2.08028454432845e-37	L6IT	LDR30m
**Stk40**	1.276282402	9.08372631893346e-34	L6IT	LDR30m
**Nr4a1**	1.278844773	1.25931995554185e-33	L6IT	LDR30m
**Homer1**	2.70238826	2.67545107028415e-33	L6IT	LDR30m
**Per1**	1.335744143	2.1141091821236e-32	L6IT	LDR30m
**Ankrd33b**	1.21063725	2.97188378941789e-31	L6IT	LDR30m
**Med14**	1.364885775	1.76288647072859e-30	L6IT	LDR30m
**Hmgcr**	1.271447447	4.1584663424263e-29	L6IT	LDR30m
**Bdnf**	1.361971858	8.83544239707108e-29	L6IT	LDR30m
**Tmem178**	1.138635811	2.87688248202015e-28	L6IT	LDR30m
**Fosb**	1.226558972	1.47476562911478e-26	L6IT	LDR30m
**Irs2**	1.338020117	2.56139404392291e-26	L6IT	LDR30m
**Mbp**	1.291397842	1.38925238042344e-25	L6IT	LDR30m
**Tiparp**	2.18674147	8.28454191079866e-25	L6IT	LDR30m
**Kdm7a**	1.303843599	9.99514638662324e-25	L6IT	LDR30m
**Hspa4**	1.181714266	1.90768157669784e-24	L6IT	LDR30m
**Slc6a17**	1.010474097	1.16837355246556e-23	L6IT	LDR30m
**Jdp2**	1.399093471	1.24403291983131e-23	L6IT	LDR30m
**Hnrnpll**	1.368506333	2.59217952619162e-23	L6IT	LDR30m

**Tbc1d9**	1.113692713	9.19409229687532e-23	L6IT	LDR30m
**Slc25a25**	1.165843733	1.23724429983154e-22	L6IT	LDR30m
**Gm17231**	1.065448247	6.43464618940604e-22	L6IT	LDR30m
**Baiap2**	1.142321944	6.93827983631047e-22	L6IT	LDR30m
**Mest**	1.278703135	1.09003264023487e-21	L6IT	LDR30m
**Ntrk2**	1.159907815	3.23418726930905e-21	L6IT	LDR30m
**Rims4**	1.146770492	7.98742895677878e-21	L6IT	LDR30m
**Nrn1**	1.226001081	4.78745748196229e-20	L6IT	LDR30m
**Spred1**	1.227770962	4.93931608609747e-20	L6IT	LDR30m
**Ndfip2**	1.421172468	1.38574729755609e-19	L6IT	LDR30m
**P4ha1**	1.000376972	1.61149413530178e-19	L6IT	LDR30m
**Kdm6b**	0.995738189	1.98390081074679e-19	L6IT	LDR30m
**Btaf1**	1.070907535	3.29368117904044e-19	L6IT	LDR30m
**Npas4**	1.080032313	3.30943244070271e-19	L6IT	LDR30m
**Etv5**	1.118834333	8.27407324005526e-19	L6IT	LDR30m
**Tulp4**	0.989775264	1.22901043899619e-18	L6IT	LDR30m
**Pcsk1**	2.052064062	1.33341504358715e-18	L6IT	LDR30m
**Rgs7bp**	0.910169606	1.85603840382773e-18	L6IT	LDR30m
**Kcnmb4**	0.939250842	1.91668667578454e-18	L6IT	LDR30m
**Txndc11**	0.927439394	3.31691682852215e-18	L6IT	LDR30m
**Grasp**	1.047791159	1.48375618088182e-17	L6IT	LDR30m
**Arid3b**	0.970949216	1.49962604275133e-17	L6IT	LDR30m
**Fbxo33**	0.999097248	1.53053602709907e-17	L6IT	LDR30m
**R3hdm2**	0.827713325	2.72744153296591e-17	L6IT	LDR30m
**Ctnnd1**	0.915143836	5.5590005588412e-17	L6IT	LDR30m
**Prickle1**	0.682179862	1.4403269055585e-16	L6IT	LDR30m
**Efhd2**	1.00369829	2.1021581779986e-16	L6IT	LDR30m
**Nmnat2**	0.856814863	2.63883171011882e-16	L6IT	LDR30m
**Sik1**	0.821680957	4.51917828176693e-16	L6IT	LDR30m
**Por**	0.939582636	4.54896653394789e-16	L6IT	LDR30m
**Nap1l1**	0.924425559	4.72766297766496e-16	L6IT	LDR30m
**Mir670hg**	0.866533892	8.62691879261529e-16	L6IT	LDR30m
**Fbl**	0.964121404	9.37865557082148e-16	L6IT	LDR30m
**Pmepa1**	1.01960009	1.13800845743351e-15	L6IT	LDR30m
**Arpc2**	0.881267786	1.20901566883841e-15	L6IT	LDR30m
**Trim9**	0.827959859	2.13951582362753e-15	L6IT	LDR30m
**Clstn3**	0.988519592	2.90399683789952e-15	L6IT	LDR30m
**Adora1**	0.926236312	3.18973383822862e-15	L6IT	LDR30m
**Tet3**	1.098885043	3.51772444239196e-15	L6IT	LDR30m

Development • Supplementary information

**Cltc**	0.843151266	6.66045900963932e-15	L6IT	LDR30m
**Phf21b**	1.198925831	1.14582685514137e-14	L6IT	LDR30m
**Per2**	0.99486058	1.29426396499074e-14	L6IT	LDR30m
**Zswim6**	0.97468076	1.59485611891173e-14	L6IT	LDR30m
**Nrd1**	1.164417386	2.88844571674639e-14	L6IT	LDR30m
**Coq10b**	0.917863226	4.41890226249173e-14	L6IT	LDR30m
**Cwc25**	0.868655612	4.54239564519661e-14	L6IT	LDR30m
**Gm47423**	2.07414155	4.88364168400368e-14	L6IT	LDR30m
**Ago3**	0.787440458	5.65215094310231e-14	L6IT	LDR30m
**Ap2b1**	0.808058356	5.98677959783863e-14	L6IT	LDR30m
**Cpeb4**	0.96181098	1.39277212198174e-13	L6IT	LDR30m
**Sik3**	0.994868821	1.50253019979003e-13	L6IT	LDR30m
**Psd3**	0.946797966	2.37462644316219e-13	L6IT	LDR30m
**Ifrd1**	0.956046404	6.69325464422959e-13	L6IT	LDR30m
**Mapk4**	0.970217154	7.73347368192465e-13	L6IT	LDR30m
**Vmp1**	0.84303943	7.90143755021569e-13	L6IT	LDR30m
**Cry2**	0.839984463	1.43445864480249e-12	L6IT	LDR30m
**Gadd45b**	0.694584045	1.58098387818077e-12	L6IT	LDR30m
**Tnfaip6**	0.752631007	2.56625771172915e-12	L6IT	LDR30m
**Ptprn**	0.972987846	3.20693741545138e-12	L6IT	LDR30m
**Dusp14**	0.867426241	5.26973264383134e-12	L6IT	LDR30m
**Zhx2**	0.82547181	5.31520378401897e-12	L6IT	LDR30m
**Galnt9**	0.994863309	5.49316256050777e-12	L6IT	LDR30m
**Lonrf1**	0.853438615	7.2424537945612e-12	L6IT	LDR30m
**Hivep2**	0.661449986	9.07471707292502e-12	L6IT	LDR30m
**Acsl4**	0.843753744	9.51487666441148e-12	L6IT	LDR30m
**Skil**	0.857031192	1.08494806592996e-11	L6IT	LDR30m
**Rock2**	0.858245592	1.21824075983409e-11	L6IT	LDR30m
**Rcc2**	0.765557456	1.58679265282282e-11	L6IT	LDR30m
**Slc2a1**	0.721222084	2.9529696950191e-11	L6IT	LDR30m
**Trib1**	0.680589667	3.00909574343308e-11	L6IT	LDR30m
**Abhd2**	0.90787469	4.09451434527027e-11	L6IT	LDR30m
**Egr4**	0.685811211	4.32251787299669e-11	L6IT	LDR30m
**Synj2**	0.797677574	4.88079619540457e-11	L6IT	LDR30m
**Mapk6**	0.752598944	5.24980392457394e-11	L6IT	LDR30m
**Cabp1**	0.793400081	9.17235121166496e-11	L6IT	LDR30m
**Midn**	0.723041669	9.96443033238835e-11	L6IT	LDR30m
**Pitpna**	0.694332667	1.67777580961001e-10	L6IT	LDR30m
**Ak4**	0.783698657	2.03020351334474e-10	L6IT	LDR30m

Development • Supplementary information

**Klf9**	0.800362303	3.75703227280705e-10	L6IT	LDR30m
**Nr4a2**	1.904710665	3.8197101746103e-10	L6IT	LDR30m
**Hsph1**	0.722251401	6.71348029240603e-10	L6IT	LDR30m
**Hsd17b12**	0.755466518	9.54527569276702e-10	L6IT	LDR30m
**Tbc1d1**	0.827724226	1.42203281744191e-09	L6IT	LDR30m
**Osbpl8**	0.850907828	2.25726431840158e-09	L6IT	LDR30m
**Rab6a**	0.744805039	3.08663775440108e-09	L6IT	LDR30m
**Crem**	0.72955019	3.72366471178633e-09	L6IT	LDR30m
**Cyp51**	0.729881167	5.41001380418325e-09	L6IT	LDR30m
**Mn1**	0.853562346	5.67112054041835e-09	L6IT	LDR30m
**Ppme1**	0.750501122	6.06071354034316e-09	L6IT	LDR30m
**Ciart**	0.642565832	7.89885889187986e-09	L6IT	LDR30m
**Dennd5b**	0.738252758	8.08451582527551e-09	L6IT	LDR30m
**Spred2**	0.951388725	1.12026234307973e-08	L6IT	LDR30m
**Arih1**	0.632905898	1.13362469527187e-08	L6IT	LDR30m
**Ina**	0.679218059	1.30472874347859e-08	L6IT	LDR30m
**Mxi1**	0.779872987	1.49866546693908e-08	L6IT	LDR30m
**Nptx2**	0.605873387	1.59109696748191e-08	L6IT	LDR30m
**Arhgap31**	0.888598894	1.90501383510773e-08	L6IT	LDR30m
**Cx3cl1**	0.747483306	2.00398801307067e-08	L6IT	LDR30m
**Hs3st2**	0.657645789	2.19487214602316e-08	L6IT	LDR30m
**Rab6b**	0.848505567	2.53656071730582e-08	L6IT	LDR30m
**Kif5c**	0.642258571	3.395906902008e-08	L6IT	LDR30m
**Fkbp1a**	0.733859161	3.44226885724209e-08	L6IT	LDR30m
**Ndel1**	0.69760413	3.93850189920091e-08	L6IT	LDR30m
**Nudt4**	0.737242447	3.95922612232238e-08	L6IT	LDR30m
**Hspa8**	0.774065513	4.60589917734926e-08	L6IT	LDR30m
**Phyhipl**	0.788397812	4.83324552832641e-08	L6IT	LDR30m
**Cdc42ep3**	0.714284354	5.89314238087526e-08	L6IT	LDR30m
**Gfod1**	0.829171232	6.58434631164914e-08	L6IT	LDR30m
**Atp1a1**	0.770623254	6.88682832473971e-08	L6IT	LDR30m
**Cap1**	0.705489834	7.02415347232718e-08	L6IT	LDR30m
**Cnnm1**	0.646632835	7.32769044244403e-08	L6IT	LDR30m
**Ubtd2**	0.796994691	9.43827753847254e-08	L6IT	LDR30m
**Csnk1a1**	0.64997822	9.52851866172522e-08	L6IT	LDR30m
**Basp1**	0.619005263	9.58224392047881e-08	L6IT	LDR30m
**Cacng3**	0.641447152	1.6599345671453e-07	L6IT	LDR30m
**Ank**	0.680471744	1.77757324204547e-07	L6IT	LDR30m
**Dok5**	0.784975584	2.01039572713e-07	L6IT	LDR30m

**Rnf217**	0.887466358	2.02624729958874e-07	L6IT	LDR30m
**Usp36**	0.665927844	2.72075586768373e-07	L6IT	LDR30m
**Gabbr1**	0.768952995	2.83060330130007e-07	L6IT	LDR30m
**Cbarp**	0.753829915	3.41316774462099e-07	L6IT	LDR30m
**Lncpint**	0.750500739	3.76111387410609e-07	L6IT	LDR30m
**Gnai3**	0.636347773	3.88317052830976e-07	L6IT	LDR30m
**Rph3a**	0.881141201	5.05040578668151e-07	L6IT	LDR30m
**Arih2**	0.667893843	5.26805463121598e-07	L6IT	LDR30m
**Lonp2**	0.679239104	5.44942264809506e-07	L6IT	LDR30m
**Tpm3**	0.620855111	5.63514623018818e-07	L6IT	LDR30m
**Pip5k1a**	0.592557743	5.70535342314031e-07	L6IT	LDR30m
**Lemd3**	0.658787779	7.21778945785034e-07	L6IT	LDR30m
**Mamld1**	0.65095938	9.44874509812818e-07	L6IT	LDR30m
**1700110K17Rik**	0.633675413	1.03027896119169e-06	L6IT	LDR30m
**Ccn1**	0.664131662	1.23751348203048e-06	L6IT	LDR30m
**Map3k14**	0.681922393	1.31074712123296e-06	L6IT	LDR30m
**Gm28294**	0.612769713	1.43243646674547e-06	L6IT	LDR30m
**Mast3**	0.696119873	1.66206673461409e-06	L6IT	LDR30m
**Ece1**	0.877867973	1.74569629801509e-06	L6IT	LDR30m
**Gpr19**	0.653567618	1.84555426064028e-06	L6IT	LDR30m
**Arf4**	0.641757081	2.59467466783495e-06	L6IT	LDR30m
**Cbfb**	0.644336421	2.6653192281629e-06	L6IT	LDR30m
**Adgrd1**	0.686858708	2.68289246903808e-06	L6IT	LDR30m
**Sept7**	0.763320797	2.75327112183079e-06	L6IT	LDR30m
**Atp6v0d1**	0.602271091	2.75772277014062e-06	L6IT	LDR30m
**Gm48747**	0.7027807	2.79875159015867e-06	L6IT	LDR30m
**Grhl1**	0.601368921	5.38969275339905e-06	L6IT	LDR30m
**Arc**	0.730532214	7.35969863923635e-06	L6IT	LDR30m
**Hrh1**	0.617696698	7.55796925604762e-06	L6IT	LDR30m
**Smg7**	0.609257136	7.66722072694216e-06	L6IT	LDR30m
**Myh9**	0.654815049	7.70478229449616e-06	L6IT	LDR30m
**Dlgap4**	0.768631191	8.75567008715041e-06	L6IT	LDR30m
**Eprs**	0.647696087	9.46575779593574e-06	L6IT	LDR30m
**Stx1b**	0.6527412	1.34415042690267e-05	L6IT	LDR30m
**Dnajc1**	0.719338787	1.45361790177754e-05	L6IT	LDR30m
**Tacc1**	0.596110119	1.46096132095448e-05	L6IT	LDR30m
**Hmgcs1**	0.62674084	1.46592661562865e-05	L6IT	LDR30m
**Btbd8**	0.627440172	1.73413315849602e-05	L6IT	LDR30m
**Atp2a2**	0.599216629	1.82195199890012e-05	L6IT	LDR30m

Development • Supplementary information

**Slc25a3**	0.717146927	1.84801820366293e-05	L6IT	LDR30m
**Kitl**	0.759632382	2.07134485735308e-05	L6IT	LDR30m
**Kpna1**	0.645224572	2.23089078845504e-05	L6IT	LDR30m
**Gramd1b**	0.711157818	2.47307604163168e-05	L6IT	LDR30m
**Smarca5**	0.707498796	2.4886526521171e-05	L6IT	LDR30m
**Chgb**	0.606026794	2.8052255450981e-05	L6IT	LDR30m
**Slc20a2**	0.644459382	2.80648834178856e-05	L6IT	LDR30m
**Scg3**	0.59212365	7.17675207974294e-05	L6IT	LDR30m
**Elovl5**	0.585706001	7.43311697122111e-05	L6IT	LDR30m
**Sec14l1**	0.636679751	0.00012285	L6IT	LDR30m
**Sptbn2**	0.663494488	0.00023304	L6IT	LDR30m
**Zfp948**	0.633082509	0.000287669	L6IT	LDR30m
**Kmt2a**	0.653722831	0.000430333	L6IT	LDR30m
**Dnajb5**	0.588576709	0.000545515	L6IT	LDR30m
**Gng2**	0.597124849	0.000800087	L6IT	LDR30m
**Arid5b**	0.601896045	0.000867887	L6IT	LDR30m
**Pde4a**	0.729933802	0.001312587	L6IT	LDR30m
**Epb41l1**	0.595314086	0.001408569	L6IT	LDR30m
**Hdac5**	0.611518587	0.004142813	L6IT	LDR30m
**Gfra1**	1.107188697	0.00455159	L6IT	LDR30m
**Sgsm1**	0.698269344	0.004756827	L6IT	LDR30m
**Clip2**	0.601201969	0.010236256	L6IT	LDR30m
**Cmip**	0.618397409	0.048942536	L6IT	LDR30m
**Arhgap31**	1.794418475	5.90984331036815e-64	L6IT	LDR4h
**1700016P03Rik**	1.664856322	9.62529280360295e-57	L6IT	LDR4h
**Homer1**	1.611159231	3.85862752234116e-49	L6IT	LDR4h
**Tmem178**	1.343555183	5.49608704902619e-37	L6IT	LDR4h
**Phf21b**	1.274761249	9.26056995411816e-37	L6IT	LDR4h
**Mapk4**	1.499706077	9.56039623843496e-35	L6IT	LDR4h
**Bdnf**	1.427687914	8.64222163282982e-33	L6IT	LDR4h
**Sik2**	1.382478096	3.16464991451378e-31	L6IT	LDR4h
**Nrp1**	1.323621675	1.92609382897227e-28	L6IT	LDR4h
**Nptx2**	1.041149169	4.07900424128969e-26	L6IT	LDR4h
**Spred2**	1.256638074	5.05749255073291e-26	L6IT	LDR4h
**Tet3**	1.27900334	5.91021115456037e-26	L6IT	LDR4h
**Rph3a**	1.152012982	8.35688487198496e-26	L6IT	LDR4h
**Brinp1**	1.058488678	4.1265010312408e-24	L6IT	LDR4h
**Adgrd1**	1.237894301	4.45432782808259e-24	L6IT	LDR4h
**Pcsk1**	1.053271184	5.99746529598197e-24	L6IT	LDR4h

Development • Supplementary information

**Mir670hg**	0.920319099	7.34513108708424e-22	L6IT	LDR4h
**Hs3st2**	0.981181538	1.25679026581359e-21	L6IT	LDR4h
**Spred1**	1.092010615	1.87808262805293e-20	L6IT	LDR4h
**Ntrk2**	1.081076701	2.6303657004652e-20	L6IT	LDR4h
**Sorcs3**	1.224327749	7.26440899909272e-20	L6IT	LDR4h
**Slc6a17**	0.890844719	8.74245668705051e-20	L6IT	LDR4h
**Tiparp**	0.892803114	2.5948508577175e-19	L6IT	LDR4h
**Sgsm1**	1.23281083	1.2927805728885e-18	L6IT	LDR4h
**Nrn1**	1.070458353	1.7703803696226e-18	L6IT	LDR4h
**Car12**	0.918723335	1.29451448881405e-17	L6IT	LDR4h
**Baz1a**	0.973887842	5.29633565222145e-17	L6IT	LDR4h
**Ddah1**	0.884773723	6.20231582958724e-17	L6IT	LDR4h
**Egr3**	0.94388926	1.18570459787956e-15	L6IT	LDR4h
**Slc9a5**	0.869401623	1.5080994698355e-14	L6IT	LDR4h
**Gramd1b**	0.983798447	2.75002490511227e-14	L6IT	LDR4h
**Sik3**	1.015246982	5.22140131520468e-14	L6IT	LDR4h
**Fosl2**	0.84262094	8.97869554009584e-14	L6IT	LDR4h
**Itgav**	1.007548334	1.41121757227142e-13	L6IT	LDR4h
**Fmnl1**	0.805790365	2.65756527974199e-13	L6IT	LDR4h
**Epb41l1**	0.776360999	3.53200726822499e-13	L6IT	LDR4h
**Grb2**	0.90965229	1.22184760018996e-12	L6IT	LDR4h
**Osbpl3**	0.785420293	1.46966511483499e-12	L6IT	LDR4h
**Kdm7a**	0.852258647	2.61750855621152e-12	L6IT	LDR4h
**Pawr**	0.658000542	3.48600027341265e-12	L6IT	LDR4h
**Rheb**	0.837327099	5.59603861030289e-12	L6IT	LDR4h
**Bcor**	0.749340315	6.15433978352698e-12	L6IT	LDR4h
**Dnajc1**	0.83462078	6.69112692343579e-12	L6IT	LDR4h
**Dot1l**	0.84230098	6.9570845455512e-12	L6IT	LDR4h
**Scube1**	0.919435558	8.10416765572737e-12	L6IT	LDR4h
**Tmtc2**	0.81523906	8.82611098065891e-12	L6IT	LDR4h
**Jarid2**	0.908170233	1.4869415214277e-11	L6IT	LDR4h
**Hnrnpll**	0.684604822	1.72091930690562e-11	L6IT	LDR4h
**Kdm6b**	0.722866467	2.89881803273922e-11	L6IT	LDR4h
**Sema5b**	0.760019793	6.99310847027551e-11	L6IT	LDR4h
**Rock2**	0.758034761	9.48096651359284e-11	L6IT	LDR4h
**Zbtb16**	0.845975518	1.49275836616746e-10	L6IT	LDR4h
**Ltbp1**	0.738853932	1.64688966001137e-10	L6IT	LDR4h
**Dok5**	0.684607744	2.32279486371404e-10	L6IT	LDR4h
**Klhl2**	0.698401911	3.92867963933475e-10	L6IT	LDR4h

Development • Supplementary information

**Grasp**	0.762194489	8.22583942610177e-10	L6IT	LDR4h
**Hectd2**	0.918697342	9.42509859748189e-10	L6IT	LDR4h
**Fstl4**	0.93681639	2.70793286375435e-09	L6IT	LDR4h
**Arid3b**	0.803080183	4.07498993890495e-09	L6IT	LDR4h
**Mapk6**	0.677410592	4.13370406353138e-09	L6IT	LDR4h
**Trim9**	0.645387214	4.8131915540579e-09	L6IT	LDR4h
**Clmp**	0.748131807	5.55364371681886e-09	L6IT	LDR4h
**Sorcs1**	0.736409223	6.76465360095817e-09	L6IT	LDR4h
**Ppm1h**	0.722837702	7.56207351068468e-09	L6IT	LDR4h
**Arhgef3**	0.728743604	1.07531254908903e-08	L6IT	LDR4h
**Ppme1**	0.70629785	1.21456168461502e-08	L6IT	LDR4h
**Ifrd1**	0.641358232	1.55962599775411e-08	L6IT	LDR4h
**Vmp1**	0.587109963	2.37599414650171e-08	L6IT	LDR4h
**Igsf9b**	0.812945821	2.45923623943007e-08	L6IT	LDR4h
**Dgki**	0.737679252	2.70980893654717e-08	L6IT	LDR4h
**Pld5**	0.760073979	3.59994832146303e-08	L6IT	LDR4h
**Por**	0.673664719	3.91572436241024e-08	L6IT	LDR4h
**Ankrd33b**	0.710106416	3.94433209921814e-08	L6IT	LDR4h
**Per2**	0.656952371	6.82760081257919e-08	L6IT	LDR4h
**Nrxn2**	0.808304489	1.02303493238858e-07	L6IT	LDR4h
**Rapgef5**	0.752084314	1.14947797114331e-07	L6IT	LDR4h
**Adgrl3**	0.631433402	1.35943307196089e-07	L6IT	LDR4h
**Gm13684**	0.72785804	1.36130708162835e-07	L6IT	LDR4h
**Actn4**	0.641566316	1.66677658307314e-07	L6IT	LDR4h
**Cap1**	0.619677478	1.67793146039114e-07	L6IT	LDR4h
**Unc13a**	0.58508871	2.21411285195349e-07	L6IT	LDR4h
**Zswim6**	0.710743821	2.9981677752402e-07	L6IT	LDR4h
**Prmt8**	0.749790495	3.08491078303446e-07	L6IT	LDR4h
**Jdp2**	0.791854579	3.14909615844358e-07	L6IT	LDR4h
**Ccdc88c**	0.602954559	3.54353351992157e-07	L6IT	LDR4h
**Nrd1**	0.641130638	7.12786726634765e-07	L6IT	LDR4h
**Hmgcr**	0.603198859	7.72414349373924e-07	L6IT	LDR4h
**2510009E07Rik**	0.607943729	1.10547210827604e-06	L6IT	LDR4h
**Stk40**	0.651471214	1.26881239787177e-06	L6IT	LDR4h
**Srgap1**	0.693442953	1.343954473192e-06	L6IT	LDR4h
**Gfra1**	1.347706534	1.48242635352489e-06	L6IT	LDR4h
**Rcan2**	0.731456708	3.04859886861044e-06	L6IT	LDR4h
**Etv5**	0.636556086	3.94090409510695e-06	L6IT	LDR4h
**Frmd6**	0.589900824	4.07320374338964e-06	L6IT	LDR4h

Development • Supplementary information

**Inhba**	0.682833457	4.30788144001892e-06	L6IT	LDR4h
**Gfod1**	0.777467552	5.19801527094128e-06	L6IT	LDR4h
**Galnt9**	0.709701674	6.71752838456731e-06	L6IT	LDR4h
**Spock3**	0.591725263	1.03205279374185e-05	L6IT	LDR4h
**Lhfpl3**	0.734734369	1.45088086987496e-05	L6IT	LDR4h
**Eml5**	0.703876308	1.54562481902424e-05	L6IT	LDR4h
**Pcdh15**	0.664526956	1.68472086871542e-05	L6IT	LDR4h
**Disp3**	1.331072962	1.98677830978713e-05	L6IT	LDR4h
**Dlgap2**	0.618321967	2.46102584231757e-05	L6IT	LDR4h
**Nptxr**	0.614026324	2.63947858562102e-05	L6IT	LDR4h
**Grin2a**	0.61334638	2.75446015901655e-05	L6IT	LDR4h
**Arhgap10**	0.590882119	2.83071993332163e-05	L6IT	LDR4h
**Fkbp1a**	0.62615319	5.087296518168e-05	L6IT	LDR4h
**Tgfbr1**	0.615349712	6.94102471650015e-05	L6IT	LDR4h
**Megf11**	1.378702593	9.85593142311327e-05	L6IT	LDR4h
**Ntrk3**	0.675647677	0.000105167	L6IT	LDR4h
**Mast3**	0.63191975	0.000141074	L6IT	LDR4h
**Prkg2**	0.586176033	0.000157112	L6IT	LDR4h
**Mbp**	0.629912166	0.000172458	L6IT	LDR4h
**Ptprn**	0.673582042	0.000305888	L6IT	LDR4h
**Dlgap4**	0.634865569	0.000531093	L6IT	LDR4h
**Ubash3b**	1.425481787	0.001414936	L6IT	LDR4h
**Ece1**	0.590142203	0.001605148	L6IT	LDR4h
**Scg2**	0.590306124	0.002140166	L6IT	LDR4h
**Zmiz1**	1.299624728	0.003552442	L6IT	LDR4h
**Galnt14**	0.618374755	0.003608351	L6IT	LDR4h
**Gm15398**	1.434181745	0.00894406	L6IT	LDR4h
**Maml3**	1.310379815	0.012737548	L6IT	LDR4h
**Fth1**	1.370798067	1.1826147081891e-33	L6IT	LDR6h
**mt-Co1**	1.51377962	6.58288264185478e-25	L6IT	LDR6h
**mt-Co3**	1.362505796	3.65939560539258e-18	L6IT	LDR6h
**Srsf7**	1.201687774	4.33934796219285e-18	L6IT	LDR6h
**Apoe**	0.971154583	1.60547172586267e-16	L6IT	LDR6h
**mt-Nd1**	1.029071923	1.13282118075992e-14	L6IT	LDR6h
**Gm27032**	0.980501278	6.64559061628623e-12	L6IT	LDR6h
**Sema3c**	0.914359291	1.01797813587399e-09	L6IT	LDR6h
**H3f3b**	0.899759178	3.788089560081e-09	L6IT	LDR6h
**Cst3**	0.75640257	6.19337334196173e-09	L6IT	LDR6h
**Cox6c**	0.773349384	1.93659693550231e-08	L6IT	LDR6h

Development • Supplementary information

**Rprml**	0.832485937	2.97711133433924e-08	L6IT	LDR6h
**Rpl9**	0.702868269	5.21886085518599e-08	L6IT	LDR6h
**Rps8**	0.744132403	9.20847435676044e-08	L6IT	LDR6h
**Gm12027**	0.794719828	9.21410262832892e-08	L6IT	LDR6h
**Pcsk1n**	0.693456578	1.0904994618727e-07	L6IT	LDR6h
**Trim35**	0.790716031	1.1534137767841e-07	L6IT	LDR6h
**Cox8a**	0.786775416	2.50740865281848e-07	L6IT	LDR6h
**Cox4i1**	0.759047143	3.46191592983935e-07	L6IT	LDR6h
**Atp6v0b**	0.944245151	5.51188028348407e-07	L6IT	LDR6h
**Ubb**	0.873765592	9.07918349218565e-07	L6IT	LDR6h
**Gm15520**	0.728262914	1.58729085692543e-06	L6IT	LDR6h
**Rpl6**	0.837586217	1.91411550318136e-06	L6IT	LDR6h
**Oprm1**	0.800086941	2.05000857264314e-06	L6IT	LDR6h
**Tmsb4x**	0.780122347	2.7790860185926e-06	L6IT	LDR6h
**Rap2b**	0.662757305	6.81422327026894e-06	L6IT	LDR6h
**Pabpc4**	0.749810858	1.07867120559016e-05	L6IT	LDR6h
**Dnaja2**	0.691468971	1.79746518218942e-05	L6IT	LDR6h
**Slc25a4**	0.667138871	4.70488709797662e-05	L6IT	LDR6h
**Eif1**	0.696303939	5.04279465877191e-05	L6IT	LDR6h
**Rps29**	0.608460487	5.41818260913481e-05	L6IT	LDR6h
**Prpf4b**	0.75469738	6.44960670274355e-05	L6IT	LDR6h
**Gm28376**	0.689692727	6.82471950926444e-05	L6IT	LDR6h
**Atp6v0c**	0.640520778	7.8839559228061e-05	L6IT	LDR6h
**Ctnna3**	0.686660703	8.01744493113441e-05	L6IT	LDR6h
**Ppia**	0.633843645	9.67845901521072e-05	L6IT	LDR6h
**Gm32250**	0.669811869	9.95515024834977e-05	L6IT	LDR6h
**Nrgn**	0.656883834	0.00012578	L6IT	LDR6h
**Mrps26**	0.638198967	0.000130581	L6IT	LDR6h
**Atxn7l3b**	0.598304341	0.000146491	L6IT	LDR6h
**Eef1a1**	0.706496349	0.000173081	L6IT	LDR6h
**Arpp19**	0.649407296	0.000214423	L6IT	LDR6h
**Gm35188**	0.784122071	0.000254006	L6IT	LDR6h
**Gm46367**	0.694915642	0.000403326	L6IT	LDR6h
**Dalrd3**	0.697778306	0.000403721	L6IT	LDR6h
**Gm26724**	0.623012415	0.000404186	L6IT	LDR6h
**Junb**	0.73708409	0.000444919	L6IT	LDR6h
**Eid1**	0.620783418	0.000583243	L6IT	LDR6h
**Cox7b**	0.64038321	0.00065231	L6IT	LDR6h
**Dact2**	0.591908698	0.000687673	L6IT	LDR6h

Development • Supplementary information

**Crebzf**	0.661186848	0.0011061	L6IT	LDR6h
**Rpl37a**	0.592628495	0.001159059	L6IT	LDR6h
**Calm1**	0.612385935	0.00126995	L6IT	LDR6h
**Rpl13**	0.665676657	0.001322648	L6IT	LDR6h
**Ckb**	0.669504731	0.001490599	L6IT	LDR6h
**Bmyc**	0.614391566	0.00297785	L6IT	LDR6h
**Mdh1**	0.591693216	0.003090782	L6IT	LDR6h
**Rpl41**	0.658664223	0.003565661	L6IT	LDR6h
**Vti1b**	0.6582592	0.00376129	L6IT	LDR6h
**Hmgn3**	0.671721588	0.003803	L6IT	LDR6h
**Ly6h**	0.587996842	0.005508824	L6IT	LDR6h
**Gm43376**	0.605023023	0.007947527	L6IT	LDR6h
**BC005561**	0.627849814	0.008477834	L6IT	LDR6h
**Rad51ap2**	0.649246179	0.014246204	L6IT	LDR6h
**Atpif1**	0.598275006	0.016306349	L6IT	LDR6h
**mt-Co2**	1.190237698	0.018221313	L6IT	LDR6h
**Pura**	0.603560271	0.02026185	L6IT	LDR6h
**Gm16351**	0.698294419	0.022839958	L6IT	LDR6h
**5330438D12Rik**	0.678019325	0.023029409	L6IT	LDR6h
**Malat1**	1.20216934	0.037117124	L6IT	LDR6h

Development • Supplementary information

Table S3. Differentially expressed genes in each cell type compared to NR.

	avg.Log2FC	p_val_adj	Cell type	Timepoint
**Tnc**	0.88953706	0.0034713	Ast	LDR
**Itpkb**	0.96654058	0.00958694	Ast	LDR2h
**Pdzd2**	1.45536037	0.02518461	Ast	LDR2h
**AW047730**	-0.6932837	0.00050207	Ast	LDR30m
**Aff1**	0.814765	0.00340434	Ast	LDR30m
**Aff4**	0.95491621	7.23E-08	Ast	LDR30m
**Ago2**	1.42394255	8.48E-15	Ast	LDR30m
**Ankrd13c**	1.05549043	3.97E-05	Ast	LDR30m
**Arhgap31**	1.46949921	7.15E-17	Ast	LDR30m
**Arid5a**	1.60563355	4.92E-12	Ast	LDR30m
**Atxn7**	0.68673953	0.00107335	Ast	LDR30m
**Baiap2**	1.35506446	8.78E-05	Ast	LDR30m
**Bmp2k**	0.62715249	0.01003754	Ast	LDR30m
**Bpnt1**	2.15438354	3.60E-17	Ast	LDR30m
**Cables1**	1.70728572	1.88E-10	Ast	LDR30m
**Cacnb2**	1.02848331	0.0244387	Ast	LDR30m
**Ccnl1**	1.24418937	1.62E-14	Ast	LDR30m
**Cdh20**	0.65343509	0.00607296	Ast	LDR30m
**Cerk**	1.99812924	1.33E-31	Ast	LDR30m
**Chp1**	1.10802159	1.56E-07	Ast	LDR30m
**Coq10b**	2.27818216	1.47E-31	Ast	LDR30m
**Cpe**	0.75009305	2.29E-10	Ast	LDR30m
**Crem**	1.2654486	9.80E-07	Ast	LDR30m
**Csdc2**	1.10533937	0.00118523	Ast	LDR30m
**Ddah1**	0.80619128	0.00033231	Ast	LDR30m
**Dhrs3**	1.71479638	3.67E-15	Ast	LDR30m
**Dio2**	1.12645306	4.18E-05	Ast	LDR30m
**Diras2**	1.33950494	0.03349871	Ast	LDR30m
**Dkk3**	1.1473788	3.05E-08	Ast	LDR30m
**Dleu2**	0.76153607	0.00090542	Ast	LDR30m
**Elovl5**	1.34301722	1.76E-12	Ast	LDR30m
**Emd**	2.253012	2.91E-22	Ast	LDR30m
**Eno1**	-0.6419477	0.00518757	Ast	LDR30m
**Epas1**	1.7413499	5.24E-39	Ast	LDR30m

**Eprs**	2.65799326	5.17E-60	Ast	LDR30m
**Ercc5**	1.05943956	0.02109137	Ast	LDR30m
**Ezr**	1.08210474	0.00065932	Ast	LDR30m
**Fam13c**	0.95823262	1.85E-08	Ast	LDR30m
**Fam20a**	0.98005427	0.000364	Ast	LDR30m
**Fgfr1**	0.80804444	0.00156521	Ast	LDR30m
**Fos**	2.14407608	9.75E-12	Ast	LDR30m
**Fosb**	1.55230987	0.00143066	Ast	LDR30m
**Fosl2**	2.55896861	5.51E-19	Ast	LDR30m
**Foxo1**	1.45453348	6.71E-11	Ast	LDR30m
**Frmd6**	1.55710472	1.35E-14	Ast	LDR30m
**Gcnt4**	1.10800309	0.00864848	Ast	LDR30m
**Gem**	2.47227065	1.71E-20	Ast	LDR30m
**Gm47423**	1.99282718	0.01754543	Ast	LDR30m
**Gm49380**	1.94013126	7.91E-07	Ast	LDR30m
**Gm6225**	1.77143813	1.75E-10	Ast	LDR30m
**Hspa4**	1.23515155	1.83E-07	Ast	LDR30m
**Htra1**	1.11355297	5.63E-10	Ast	LDR30m
**Ifrd1**	1.39730047	2.96E-14	Ast	LDR30m
**Irs2**	2.36054276	6.29E-30	Ast	LDR30m
**Isy1**	1.42204214	1.57E-05	Ast	LDR30m
**Jdp2**	1.17359079	0.00211561	Ast	LDR30m
**Jmy**	0.9366668	0.04956156	Ast	LDR30m
**Kcnj10**	-0.6949445	0.00143596	Ast	LDR30m
**Kdm5b**	0.91167646	2.32E-05	Ast	LDR30m
**Klf4**	1.51254488	0.00185311	Ast	LDR30m
**Lncpint**	0.90562798	0.03395408	Ast	LDR30m
**Man1b1**	1.50597252	2.47E-07	Ast	LDR30m
**Map3k19**	1.98820142	6.57E-11	Ast	LDR30m
**Mapkapk2**	1.24683383	4.86E-05	Ast	LDR30m
**Mbd2**	0.75395778	0.04810292	Ast	LDR30m
**Mbnl1**	0.65253424	0.01622104	Ast	LDR30m
**Mef2d**	0.96627962	0.00260773	Ast	LDR30m
**Mest**	1.40732975	0.01766414	Ast	LDR30m
**Mia3**	1.08992403	0.00021758	Ast	LDR30m
**Mlc1**	0.86015393	0.04760249	Ast	LDR30m
**Mtss2**	-0.7972427	7.75E-08	Ast	LDR30m
**Mxi1**	1.22456903	7.86E-08	Ast	LDR30m
**Nr4a1**	2.30304174	0.00034009	Ast	LDR30m

Development • Supplementary information

**Nr4a2**	3.23040444	2.93E-35	Ast	LDR30m
**Nr4a3**	3.49030359	4.75E-05	Ast	LDR30m
**Ntrk2**	1.41302383	2.25E-18	Ast	LDR30m
**Olfml2a**	1.3991126	0.0013285	Ast	LDR30m
**Palmd**	1.19659407	0.00354094	Ast	LDR30m
**Paqr8**	0.74185688	0.00721638	Ast	LDR30m
**Pcdh10**	-0.7176107	5.63E-05	Ast	LDR30m
**Pcgf5**	1.0037884	4.06E-07	Ast	LDR30m
**Pcsk1**	1.54078021	0.02085924	Ast	LDR30m
**Per1**	1.92862233	2.39E-13	Ast	LDR30m
**Plekhg5**	1.41680328	0.01123823	Ast	LDR30m
**Pmepa1**	1.42218811	0.00702125	Ast	LDR30m
**Prdx6**	-0.9063442	0.00136451	Ast	LDR30m
**Ptbp1**	1.51132258	2.53E-06	Ast	LDR30m
**Rasa2**	0.66505256	0.00912171	Ast	LDR30m
**Rfx4**	1.76033339	4.04E-63	Ast	LDR30m
**Rheb**	1.13877451	1.83E-08	Ast	LDR30m
**Sfpq**	0.63488612	0.0400977	Ast	LDR30m
**Sik1**	2.50163036	0.03098246	Ast	LDR30m
**Sik2**	1.38313778	5.16E-14	Ast	LDR30m
**Sik3**	0.6690308	0.02845777	Ast	LDR30m
**Ski**	1.1260388	0.00023185	Ast	LDR30m
**Slc3a2**	1.4996182	8.04E-13	Ast	LDR30m
**Slc7a5**	1.76869114	5.98E-10	Ast	LDR30m
**Slco1c1**	0.88897931	0.02728624	Ast	LDR30m
**Sox9**	-0.9153292	0.00135081	Ast	LDR30m
**Tle1**	1.15880547	1.87E-07	Ast	LDR30m
**Tle3**	1.30226249	0.00438973	Ast	LDR30m
**Tpm3**	1.12690566	6.50E-06	Ast	LDR30m
**Trib2**	-0.6279918	0.00724289	Ast	LDR30m
**Trmt61b**	1.48999871	5.69E-08	Ast	LDR30m
**Tubgcp3**	1.31690323	1.97E-05	Ast	LDR30m
**Txndc11**	1.28795968	8.38E-12	Ast	LDR30m
**Unc45a**	1.41537486	2.49E-06	Ast	LDR30m
**Usp2**	2.61829266	5.18E-44	Ast	LDR30m
**Usp53**	1.30263605	0.00052064	Ast	LDR30m
**Vps37b**	1.44210186	4.19E-06	Ast	LDR30m
**Zbtb38**	0.90439288	0.00981968	Ast	LDR30m
**Zdbf2**	1.40533875	0.01163212	Ast	LDR30m

Development • Supplementary information

**Zfp516**	0.86745987	0.00014788	Ast	LDR30m
**Zhx2**	1.11267089	4.49E-14	Ast	LDR30m
**Zswim6**	1.98044472	2.56E-13	Ast	LDR30m
**Cabp1**	1.42151954	3.84E-05	Ast	LDR4h
**Crim1**	0.75548825	0.04176135	Ast	LDR4h
**Epb41l1**	1.18694654	0.03447743	Ast	LDR4h
**Epha5**	0.72511851	0.00906749	Ast	LDR4h
**Galnt9**	1.56760572	0.00145307	Ast	LDR4h
**Hs3st2**	1.6213937	0.00016997	Ast	LDR4h
**Iqgap2**	1.45166164	9.08E-05	Ast	LDR4h
**Itpkb**	0.9984977	0.00112483	Ast	LDR4h
**Maml3**	1.53278494	1.26E-05	Ast	LDR4h
**Mpped2**	0.73441876	0.00011024	Ast	LDR4h
**Pde2a**	1.21703085	0.00083252	Ast	LDR4h
**Pdzd2**	1.30707838	4.07E-07	Ast	LDR4h
**Ppm1h**	1.11570603	7.60E-05	Ast	LDR4h
**Sgsm1**	1.59778413	0.00031097	Ast	LDR4h
**Sik2**	1.08772122	7.28E-06	Ast	LDR4h
**Sorbs1**	0.77819703	0.00936339	Ast	LDR4h
**Sorcs1**	1.36247555	0.0001521	Ast	LDR4h
**Sorcs3**	1.64248524	3.94E-08	Ast	LDR4h
**Tmeff2**	0.60019268	0.00155664	Grin3a	LDR
**Mir670hg**	2.2006019	0.00039412	Grin3a	LDR2h
**Afap1**	1.02345152	1.59E-07	Grin3a	LDR30m
**Ago2**	1.11412521	0.00101866	Grin3a	LDR30m
**Ankrd33b**	1.35054159	0.02247267	Grin3a	LDR30m
**Arid5b**	1.12474851	7.39E-06	Grin3a	LDR30m
**Baiap2**	1.44455752	0.02418306	Grin3a	LDR30m
**Bcl2l11**	1.46602774	0.00516651	Grin3a	LDR30m
**Cep112**	1.10082906	0.00128645	Grin3a	LDR30m
**Cpeb3**	1.04368464	6.74E-12	Grin3a	LDR30m
**Cry2**	1.11597403	0.01218026	Grin3a	LDR30m
**Eepd1**	1.34638527	2.09E-05	Grin3a	LDR30m
**Ell2**	0.95201977	1.93E-05	Grin3a	LDR30m
**Fosb**	2.05273925	6.82E-06	Grin3a	LDR30m
**Fosl2**	2.17809324	2.90E-12	Grin3a	LDR30m
**Frmd6**	1.29536594	9.14E-07	Grin3a	LDR30m
**Gad1**	1.56740353	9.19E-15	Grin3a	LDR30m
**Gm47423**	1.34964511	2.32E-07	Grin3a	LDR30m

**Gpt2**	1.51423859	5.26E-13	Grin3a	LDR30m
**Homer1**	1.78420933	0.00047721	Grin3a	LDR30m
**Laptm4b**	1.18456003	0.00346816	Grin3a	LDR30m
**Lncpint**	0.78166877	1.25E-08	Grin3a	LDR30m
**Mbnl2**	0.65029011	0.03368738	Grin3a	LDR30m
**Mtcl1**	1.04969151	1.22E-09	Grin3a	LDR30m
**Nectin2**	1.3726538	0.03789592	Grin3a	LDR30m
**Nr4a1**	1.55525947	1.45E-05	Grin3a	LDR30m
**Nr4a2**	2.40652361	1.07E-10	Grin3a	LDR30m
**Ntrk2**	0.61494138	9.46E-06	Grin3a	LDR30m
**Osbpl8**	0.90169494	1.05E-05	Grin3a	LDR30m
**Pak1**	0.76205429	0.00659246	Grin3a	LDR30m
**Per1**	1.6418156	0.0001335	Grin3a	LDR30m
**Plxnc1**	1.20749336	3.84E-05	Grin3a	LDR30m
**Ppm1l**	1.05249084	4.64E-06	Grin3a	LDR30m
**Prkg2**	1.02301633	0.0285744	Grin3a	LDR30m
**Rcan2**	1.09402901	6.06E-09	Grin3a	LDR30m
**Sik1**	1.51830651	0.01862138	Grin3a	LDR30m
**Sik3**	0.74808456	0.00077638	Grin3a	LDR30m
**Slc23a2**	0.84805448	3.06E-05	Grin3a	LDR30m
**Slc2a13**	0.68662281	0.04788661	Grin3a	LDR30m
**Slc7a8**	1.31693375	8.01E-09	Grin3a	LDR30m
**Snap25**	0.64762806	0.01187459	Grin3a	LDR30m
**Syn2**	0.65070779	0.00132759	Grin3a	LDR30m
**Trim9**	0.8092846	2.46E-06	Grin3a	LDR30m
**Vegfc**	1.3862461	1.32E-08	Grin3a	LDR30m
**Zswim6**	0.92091358	0.00635192	Grin3a	LDR30m
**Alk**	0.93914718	3.84E-07	Grin3a	LDR4h
**Bach2**	0.79261701	0.03609563	Grin3a	LDR4h
**Bcas1**	1.61101741	0.01582434	Grin3a	LDR4h
**D7Ertd443e**	1.62174252	0.0475943	Grin3a	LDR4h
**Gabra2**	0.82419488	0.01514794	Grin3a	LDR4h
**Gad1**	0.94581697	0.02142732	Grin3a	LDR4h
**Gm32647**	0.8204658	0.00404689	Grin3a	LDR4h
**Klhl2**	0.87548409	0.01592351	Grin3a	LDR4h
**Mbp**	1.53670988	1.64E-11	Grin3a	LDR4h
**Mir670hg**	1.78187374	1.06E-11	Grin3a	LDR4h
**Mobp**	1.74334718	1.46E-06	Grin3a	LDR4h
**Mog**	1.4675288	0.03430056	Grin3a	LDR4h

Development • Supplementary information

**Ninj2**	1.57146195	0.04998757	Grin3a	LDR4h
**Pcdh15**	0.7120717	0.00628323	Grin3a	LDR4h
**Sik3**	0.74619968	0.00014952	Grin3a	LDR4h
**Tenm4**	0.74273772	1.95E-05	Grin3a	LDR4h
**9530026P05Rik**	1.0583547	0.00169061	L23	LDR
**Ablim3**	0.910327	0.04965945	L23	LDR
**Adamts17**	0.9764864	2.26E-06	L23	LDR
**Adcyap1**	0.64220053	0.04696433	L23	LDR
**Agmat**	0.71102359	2.14E-07	L23	LDR
**Aldh2**	1.00902303	0.00079615	L23	LDR
**Apbb1ip**	1.06499774	3.85E-06	L23	LDR
**Bdnf**	-0.8761792	0.00012597	L23	LDR
**Bnc2**	1.12055505	1.93E-07	L23	LDR
**Cacna1h**	0.8925154	0.03897479	L23	LDR
**Cacnb2**	0.65039415	2.75E-06	L23	LDR
**Cdkl4**	0.87219095	0.01342244	L23	LDR
**Chst9**	1.22555656	3.28E-09	L23	LDR
**Clmn**	1.12413986	2.44E-09	L23	LDR
**Crim1**	0.856572	2.53E-12	L23	LDR
**Dcc**	0.59934491	5.42E-05	L23	LDR
**Dgkg**	0.75903784	5.63E-05	L23	LDR
**Dkk2**	0.76757879	0.0044764	L23	LDR
**Dock2**	0.70307251	0.0020465	L23	LDR
**Eda**	0.72023596	0.00354995	L23	LDR
**Evc**	0.98324255	0.00638772	L23	LDR
**Evc2**	0.83384403	0.03732535	L23	LDR
**F730043M19Rik**	1.10003259	6.76E-24	L23	LDR
**Fam126a**	1.0805267	5.96E-09	L23	LDR
**Fam214a**	0.7495259	0.03307232	L23	LDR
**Flt3**	0.93918027	0.01212856	L23	LDR
**Fndc1**	1.11230345	0.00013449	L23	LDR
**Fstl1**	0.90319107	0.0003832	L23	LDR
**Fzd10os**	0.71755352	3.40E-06	L23	LDR
**Galnt16**	0.89340637	0.00425539	L23	LDR
**Gipc2**	1.04502739	0.00020337	L23	LDR
**Gm10649**	0.85106944	3.32E-05	L23	LDR
**Gm15155**	0.69253953	0.0070147	L23	LDR
**Gm15398**	-1.0694443	3.47E-05	L23	LDR
**Gm1604a**	1.21860101	1.01E-15	L23	LDR

Development • Supplementary information

**Gm1604b**	1.08759925	0.00111285	L23	LDR
**Gm16070**	1.02318463	0.0025809	L23	LDR
**Gm17276**	0.77092649	0.00967825	L23	LDR
**Gm34667**	1.06461446	0.00042152	L23	LDR
**Gm45323**	1.04443813	0.03328879	L23	LDR
**Gm45341**	1.06064015	0.0076588	L23	LDR
**Gm49969**	0.87250561	0.00028236	L23	LDR
**Gm50445**	0.77986795	0.01878501	L23	LDR
**Gpc3**	1.09573964	4.77E-05	L23	LDR
**Gpc5**	0.74293934	0.00619955	L23	LDR
**Grb14**	0.67658072	6.49E-05	L23	LDR
**Hlf**	1.03877732	3.12E-05	L23	LDR
**Itga8**	0.88316278	0.00056674	L23	LDR
**Kcnip2**	0.83686895	0.01091815	L23	LDR
**Kcnq4**	0.99678797	0.00022507	L23	LDR
**Kctd8**	1.29254951	1.03E-05	L23	LDR
**L3mbtl4**	1.02409376	2.65E-11	L23	LDR
**Laptm4b**	1.00241416	2.57E-06	L23	LDR
**Lgi3**	0.86785117	0.00031133	L23	LDR
**Lzts3**	0.6291725	0.01787845	L23	LDR
**Mapk3**	1.02338612	0.0001102	L23	LDR
**Matn2**	1.23014912	1.77E-05	L23	LDR
**Mei4**	1.20470564	8.29E-15	L23	LDR
**Mpp6**	0.61402864	4.12E-06	L23	LDR
**Mpzl1**	0.89906644	0.01025467	L23	LDR
**Mylk3**	0.69092208	0.01120203	L23	LDR
**N4bp2**	1.01740417	6.95E-05	L23	LDR
**Nell1**	-0.6031246	0.01144449	L23	LDR
**Nr3c2**	0.92358039	0.00038133	L23	LDR
**Nr4a1**	-0.7003106	1.14E-06	L23	LDR
**Nrn1**	-0.7179414	0.0018059	L23	LDR
**Otof**	1.10424769	0.00013712	L23	LDR
**Parvb**	1.02850831	0.04623197	L23	LDR
**Pcdh15**	-0.7828479	0.00113886	L23	LDR
**Pdcd4**	0.63663957	2.80E-06	L23	LDR
**Prcd**	1.00834123	0.00983351	L23	LDR
**Proser2**	0.7508288	0.04150334	L23	LDR
**Psrc1**	0.96441187	0.03669116	L23	LDR
**Rarg**	1.00977506	0.0025779	L23	LDR

**Rgma**	1.02822901	0.01831038	L23	LDR
**Sema6a**	0.9158938	2.98E-05	L23	LDR
**Sh3bp4**	1.02233396	0.00147071	L23	LDR
**Sh3gl3**	0.88206228	0.00560488	L23	LDR
**Slco2a1**	0.68825513	0.03052536	L23	LDR
**Sncaip**	0.98769687	3.90E-08	L23	LDR
**Sorbs2os**	-0.6667984	0.01470122	L23	LDR
**Spa17**	0.60931698	0.00010515	L23	LDR
**Spata16**	0.63876038	0.04642562	L23	LDR
**Tgfbr3**	1.07667435	0.01408795	L23	LDR
**Tmem117**	0.92306829	0.0001639	L23	LDR
**Tmem150c**	1.09523966	2.36E-07	L23	LDR
**Tspan11**	0.59179792	6.50E-06	L23	LDR
**Vegfc**	0.88500473	0.02609374	L23	LDR
**Vtcn1**	0.79541179	0.00165224	L23	LDR
**Vwc2l**	1.16505597	4.27E-06	L23	LDR
**Wipf2**	0.5858263	0.0174552	L23	LDR
**Wscd1**	0.99422613	0.04970074	L23	LDR
**Zbtb7c**	0.96225157	0.00394328	L23	LDR
**4930519K11Rik**	1.09297425	0.00044645	L23	LDR2h
**5830416I19Rik**	1.13966122	0.02368601	L23	LDR2h
**A430090L17Rik**	1.00954162	0.02058893	L23	LDR2h
**Ap1s3**	0.88290563	0.02563813	L23	LDR2h
**Bach2os**	1.078221	0.0116804	L23	LDR2h
**Chrm2**	1.06473976	0.0067877	L23	LDR2h
**Cited2**	1.22589376	0.00603827	L23	LDR2h
**Gadd45b**	1.14329913	0.00965602	L23	LDR2h
**Gm14636**	0.8324019	0.01874902	L23	LDR2h
**Gnal**	0.99712966	7.69E-05	L23	LDR2h
**Grp**	1.03496725	0.03160578	L23	LDR2h
**Gucy1a1**	0.78033031	0.04508822	L23	LDR2h
**Htr1b**	1.06662795	0.03057176	L23	LDR2h
**Inhba**	1.06313974	1.42E-08	L23	LDR2h
**Man1a**	0.91931165	0.00337938	L23	LDR2h
**Nrsn1**	0.95827492	0.00023336	L23	LDR2h
**Pcdh10**	0.59277112	0.02507189	L23	LDR2h
**Ppme1**	0.96541542	0.01370235	L23	LDR2h
**Prim2**	1.21499774	0.0001694	L23	LDR2h
**Prkg2**	1.18946872	0.00470394	L23	LDR2h

Development • Supplementary information

**Ptgs2**	1.18818169	4.44E-05	L23	LDR2h
**Rapgef5**	0.94908594	4.05E-06	L23	LDR2h
**Tbr1**	0.88972467	0.01878189	L23	LDR2h
**Tmem163**	1.1172082	0.03444784	L23	LDR2h
**Tmtc2**	0.82782093	0.03736717	L23	LDR2h
**1600020E01Rik**	1.19559708	3.57E-27	L23	LDR30m
**1700110K17Rik**	1.01358348	0.02199816	L23	LDR30m
**1810055G02Rik**	0.92665078	0.00143982	L23	LDR30m
**2810455O05Rik**	0.99029866	0.00030307	L23	LDR30m
**4930412C18Rik**	0.70995492	0.03633743	L23	LDR30m
**4930486I03Rik**	0.79657204	0.02817143	L23	LDR30m
**4930599N23Rik**	1.17085358	0.00035679	L23	LDR30m
**4931406P16Rik**	0.75388201	0.00168551	L23	LDR30m
**4931413K12Rik**	1.01753177	0.01008707	L23	LDR30m
**9130017K11Rik**	0.7331032	0.02946191	L23	LDR30m
**9530026P05Rik**	1.02984073	0.01791901	L23	LDR30m
**Abhd2**	0.90565991	0.00043734	L23	LDR30m
**Acly**	0.94742114	1.40E-07	L23	LDR30m
**Acsl4**	1.06757963	1.41E-11	L23	LDR30m
**Acss1**	1.06484824	9.61E-08	L23	LDR30m
**Adamts17**	1.13803164	2.26E-09	L23	LDR30m
**Adgra1**	0.81025419	0.00086857	L23	LDR30m
**Adnp**	0.8333376	0.00011771	L23	LDR30m
**Adora1**	1.27602345	1.80E-19	L23	LDR30m
**Aff1**	0.99193359	9.12E-05	L23	LDR30m
**Aff4**	0.68212928	4.62E-08	L23	LDR30m
**Aftph**	0.60845261	0.00021903	L23	LDR30m
**Agap2**	0.80323411	2.01E-05	L23	LDR30m
**Agap3**	1.00303829	2.02E-09	L23	LDR30m
**Agmat**	0.70917007	9.84E-05	L23	LDR30m
**Ago2**	1.18012454	4.62E-29	L23	LDR30m
**Ago3**	0.93931882	2.74E-08	L23	LDR30m
**Ak4**	1.06076773	3.23E-19	L23	LDR30m
**Aldh18a1**	0.90205198	0.00204392	L23	LDR30m
**Amigo3**	1.0610785	0.00207122	L23	LDR30m
**Ank**	1.02880109	3.97E-10	L23	LDR30m
**Ankfn1**	1.12067776	0.04186633	L23	LDR30m
**Ankrd17**	0.60952125	6.10E-06	L23	LDR30m
**Ankrd33b**	1.17536815	1.60E-19	L23	LDR30m

Development • Supplementary information

**Anks1**	1.42267742	8.44E-28	L23	LDR30m
**Ap2b1**	0.94847569	2.82E-08	L23	LDR30m
**Arf4**	0.93767216	1.24E-05	L23	LDR30m
**Arhgap1**	0.95187415	3.72E-15	L23	LDR30m
**Arhgef3**	1.77890257	3.92E-05	L23	LDR30m
**Arhgef7**	0.68994125	2.87E-06	L23	LDR30m
**Arid3b**	1.12014829	0.00032442	L23	LDR30m
**Arid5a**	1.20378689	2.01E-12	L23	LDR30m
**Arid5b**	0.94042482	6.69E-09	L23	LDR30m
**Arih1**	1.01694515	4.00E-20	L23	LDR30m
**Arih2**	1.1849371	6.08E-45	L23	LDR30m
**Arl13b**	0.87065228	0.00054997	L23	LDR30m
**Arl5b**	2.1645175	3.39E-07	L23	LDR30m
**Arpc2**	1.20314165	2.06E-11	L23	LDR30m
**Arpc3**	0.78728843	0.00279572	L23	LDR30m
**Atg2b**	0.76621043	0.01905962	L23	LDR30m
**Atl2**	0.89937566	2.37E-12	L23	LDR30m
**Atp11a**	0.87717189	1.95E-06	L23	LDR30m
**Atp6v0d1**	0.99249323	5.49E-12	L23	LDR30m
**Atp7a**	1.08684498	5.37E-13	L23	LDR30m
**Atxn7**	0.80955218	9.70E-09	L23	LDR30m
**Azin1**	0.70496227	0.0023949	L23	LDR30m
**BC005537**	0.68777731	8.52E-06	L23	LDR30m
**BC049715**	1.0426417	0.0248035	L23	LDR30m
**Bach2**	0.80188615	0.01230358	L23	LDR30m
**Bag2**	1.00692375	0.00108805	L23	LDR30m
**Baiap2**	1.49451208	1.06E-33	L23	LDR30m
**Bcl6**	1.28263778	1.33E-14	L23	LDR30m
**Bdp1**	0.62000337	0.00100669	L23	LDR30m
**Bicdl1**	0.86766028	1.75E-07	L23	LDR30m
**Blvrb**	1.02774912	0.00769896	L23	LDR30m
**Bnc2**	1.1267969	2.18E-06	L23	LDR30m
**Bpnt1**	0.88988443	5.48E-06	L23	LDR30m
**Braf**	0.68803861	1.90E-06	L23	LDR30m
**Brd4**	0.63037837	0.00517144	L23	LDR30m
**Btaf1**	1.284365	7.13E-31	L23	LDR30m
**Btbd8**	0.84663831	1.51E-07	L23	LDR30m
**Btbd9**	0.63902334	0.00367649	L23	LDR30m
**Btg3**	1.04871225	0.00505261	L23	LDR30m

Development • Supplementary information

**Cabp1**	0.79507873	1.34E-12	L23	LDR30m
**Cacnb2**	0.59825659	0.00160728	L23	LDR30m
**Calu**	0.86515755	0.0007584	L23	LDR30m
**Camsap2**	0.65259801	9.64E-09	L23	LDR30m
**Cbarp**	1.11120426	8.51E-09	L23	LDR30m
**Cbfb**	1.23120165	8.10E-12	L23	LDR30m
**Cbln2**	0.97748826	0.00090469	L23	LDR30m
**Ccdc136**	0.94334265	4.24E-07	L23	LDR30m
**Ccdc6**	0.71492743	0.00111052	L23	LDR30m
**Ccm2**	1.33130345	2.73E-27	L23	LDR30m
**Ccnl1**	0.83589505	4.12E-08	L23	LDR30m
**Cdc14b**	0.76447468	0.00463116	L23	LDR30m
**Cdc27**	0.74575374	0.0002364	L23	LDR30m
**Cdc42ep3**	1.01825752	5.92E-09	L23	LDR30m
**Cdk13**	0.68985942	2.48E-07	L23	LDR30m
**Cds1**	1.04030481	3.04E-14	L23	LDR30m
**Celf6**	0.92887892	3.92E-05	L23	LDR30m
**Cenpa**	1.0660847	0.00048358	L23	LDR30m
**Cep120**	0.76569762	4.46E-05	L23	LDR30m
**Cfap36**	0.64951479	5.42E-07	L23	LDR30m
**Chd1**	0.70164521	9.15E-06	L23	LDR30m
**Chd2**	0.6853334	4.80E-07	L23	LDR30m
**Chgb**	1.05919489	3.02E-05	L23	LDR30m
**Chp1**	0.77182027	3.20E-09	L23	LDR30m
**Chst9**	0.99487832	5.07E-06	L23	LDR30m
**Ciapin1**	0.91729319	2.52E-11	L23	LDR30m
**Ciart**	1.42618918	3.76E-13	L23	LDR30m
**Clip2**	1.16953708	2.61E-21	L23	LDR30m
**Clmn**	0.91357159	0.00497191	L23	LDR30m
**Clstn3**	1.31629663	1.00E-17	L23	LDR30m
**Cltc**	1.12356712	6.12E-14	L23	LDR30m
**Cmip**	1.09301239	9.86E-19	L23	LDR30m
**Cnih3**	1.08667719	0.03640722	L23	LDR30m
**Cnnm1**	0.73436017	2.20E-08	L23	LDR30m
**Coq10b**	1.33885058	4.74E-11	L23	LDR30m
**Coro1c**	1.1133618	1.67E-08	L23	LDR30m
**Coro2a**	0.75114829	0.03166837	L23	LDR30m
**Cpeb3**	1.11291938	6.90E-10	L23	LDR30m
**Cpeb4**	1.19479596	3.51E-10	L23	LDR30m

Development • Supplementary information

**Cpne7**	0.9219092	0.00244757	L23	LDR30m
**Crem**	1.02177157	1.28E-07	L23	LDR30m
**Crim1**	0.73801519	9.77E-13	L23	LDR30m
**Cry1**	1.09284018	4.24E-05	L23	LDR30m
**Cry2**	1.11896029	9.50E-18	L23	LDR30m
**Csde1**	0.61784582	0.00022482	L23	LDR30m
**Cse1l**	0.80461348	0.00263058	L23	LDR30m
**Csnk1a1**	0.74857202	7.69E-09	L23	LDR30m
**Csnk1d**	1.03628553	7.23E-12	L23	LDR30m
**Csrnp2**	0.8697992	3.49E-05	L23	LDR30m
**Ctdp1**	0.93550422	3.39E-06	L23	LDR30m
**Ctnnb1**	0.73131818	0.00457384	L23	LDR30m
**Ctnnd1**	0.88895943	3.13E-11	L23	LDR30m
**Ctps**	1.3047635	8.12E-28	L23	LDR30m
**Cwc25**	1.24159183	1.88E-27	L23	LDR30m
**Cxadr**	0.93389665	1.00E-10	L23	LDR30m
**Cxcl12**	0.68506884	0.02476467	L23	LDR30m
**Cyfip2**	0.65108943	0.00026917	L23	LDR30m
**Cystm1**	1.02658966	4.66E-16	L23	LDR30m
**D030028A08Rik**	1.05811403	1.31E-12	L23	LDR30m
**D5Ertd579e**	0.68674062	6.61E-10	L23	LDR30m
**D730045A05Rik**	1.01641653	0.00230847	L23	LDR30m
**D930015M05Rik**	1.00835244	1.35E-08	L23	LDR30m
**Ddx27**	0.78165778	0.00468346	L23	LDR30m
**Ddx3y**	1.22233899	3.51E-16	L23	LDR30m
**Dennd5b**	0.79494592	9.76E-09	L23	LDR30m
**Dgkg**	0.68006063	0.00135762	L23	LDR30m
**Diaph1**	0.87396361	5.20E-09	L23	LDR30m
**Dkk2**	0.76307876	0.04142358	L23	LDR30m
**Dlg4**	0.62638897	0.00986608	L23	LDR30m
**Dlgap4**	0.92563178	6.41E-11	L23	LDR30m
**Dnaja1**	0.82530807	0.00049016	L23	LDR30m
**Dnajc1**	0.87004011	2.94E-08	L23	LDR30m
**Dnm3**	0.67726794	0.012738	L23	LDR30m
**Dok5**	0.83318268	0.00292288	L23	LDR30m
**Dpy19l3**	0.79846238	0.0001834	L23	LDR30m
**Dpysl5**	1.17070621	1.85E-10	L23	LDR30m
**Dusp14**	0.90902665	9.00E-09	L23	LDR30m
**Dusp16**	1.22434585	6.03E-18	L23	LDR30m

Development • Supplementary information

**Dyrk1a**	0.69551262	0.00201048	L23	LDR30m
**E330009J07Rik**	0.79632764	1.11E-09	L23	LDR30m
**Ece1**	1.28474627	1.55E-23	L23	LDR30m
**Eef1akmt4**	1.0483982	0.00151717	L23	LDR30m
**Efcab6**	0.85821737	2.34E-09	L23	LDR30m
**Efhd2**	1.05178499	0.0001228	L23	LDR30m
**Egln2**	1.19739696	5.11E-10	L23	LDR30m
**Egr3**	2.0489859	0.00874821	L23	LDR30m
**Eif2ak3**	0.87237433	3.79E-06	L23	LDR30m
**Eif4a1**	0.98651941	1.79E-05	L23	LDR30m
**Eif4g1**	0.63331259	0.01147304	L23	LDR30m
**Elf2**	0.66651359	0.03293867	L23	LDR30m
**Elk4**	0.82001152	0.02020674	L23	LDR30m
**Ell2**	0.6629955	5.51E-05	L23	LDR30m
**Elovl4**	0.88965637	0.00214685	L23	LDR30m
**Elovl5**	1.32467003	2.90E-21	L23	LDR30m
**Elp5**	0.95359288	1.25E-12	L23	LDR30m
**Eml4**	0.64868947	2.50E-22	L23	LDR30m
**Epb41l1**	0.74765851	7.25E-07	L23	LDR30m
**Epha4**	0.59130893	9.22E-08	L23	LDR30m
**Ephb3**	1.13249984	8.36E-09	L23	LDR30m
**Eprs**	1.22633129	1.83E-31	L23	LDR30m
**Ercc5**	0.77616142	0.00013277	L23	LDR30m
**Erf**	1.04426335	1.74E-10	L23	LDR30m
**Ern1**	1.23266087	1.26E-25	L23	LDR30m
**Ero1l**	0.82396025	0.00012015	L23	LDR30m
**Etf1**	0.9895771	2.82E-16	L23	LDR30m
**Etv6**	0.65647819	0.00104904	L23	LDR30m
**F730043M19Rik**	0.8407631	0.00011596	L23	LDR30m
**Fam107b**	1.12460803	3.79E-11	L23	LDR30m
**Fam189a2**	0.96048243	0.00026256	L23	LDR30m
**Fam219a**	0.59554073	0.00081085	L23	LDR30m
**Fam71e1**	0.72092833	0.00712924	L23	LDR30m
**Fam91a1**	1.03305562	6.31E-12	L23	LDR30m
**Fat4**	0.89041684	1.01E-05	L23	LDR30m
**Fbl**	1.42309423	4.27E-26	L23	LDR30m
**Fbrsl1**	0.8559649	3.63E-13	L23	LDR30m
**Fbxo33**	1.30479784	1.92E-13	L23	LDR30m
**Fbxo42**	0.66205774	0.01912723	L23	LDR30m

**Fkbp1a**	0.81764586	0.00027154	L23	LDR30m
**Flrt2**	0.82479099	0.00051641	L23	LDR30m
**Fmr1**	0.70863413	0.00207462	L23	LDR30m
**Fnbp1l**	0.74089024	0.0082181	L23	LDR30m
**Foxo3**	1.11594877	1.88E-08	L23	LDR30m
**Frmd6**	1.62245519	1.20E-21	L23	LDR30m
**Frzb**	0.69285699	0.00277323	L23	LDR30m
**Fzd10os**	0.71967807	0.00277939	L23	LDR30m
**Fzd3**	0.79048451	9.26E-08	L23	LDR30m
**Gabbr1**	0.8736847	1.73E-16	L23	LDR30m
**Gadd45b**	1.08492029	0.02021207	L23	LDR30m
**Gak**	0.94486322	1.02E-10	L23	LDR30m
**Gclc**	0.96642239	1.24E-11	L23	LDR30m
**Gda**	-0.610422	0.01796021	L23	LDR30m
**Gdap2**	0.95715728	2.14E-14	L23	LDR30m
**Gfod1**	0.83947479	1.89E-18	L23	LDR30m
**Glcci1**	0.85636024	8.56E-11	L23	LDR30m
**Gls2**	0.86121424	0.00014981	L23	LDR30m
**Gm1043**	0.64505359	0.00155059	L23	LDR30m
**Gm12940**	0.72883076	4.61E-07	L23	LDR30m
**Gm13012**	1.07683661	0.00049519	L23	LDR30m
**Gm15638**	1.0959593	2.30E-06	L23	LDR30m
**Gm15860**	0.59908158	0.02171214	L23	LDR30m
**Gm1604a**	1.18233259	1.53E-08	L23	LDR30m
**Gm17491**	1.02440066	0.00087308	L23	LDR30m
**Gm2164**	0.7978498	1.71E-05	L23	LDR30m
**Gm28791**	1.00781105	0.0050396	L23	LDR30m
**Gm3294**	0.76598061	0.00573629	L23	LDR30m
**Gm38190**	0.97020215	0.02347653	L23	LDR30m
**Gm39326**	0.82158192	0.00016302	L23	LDR30m
**Gm42937**	1.22552038	8.38E-08	L23	LDR30m
**Gm42941**	1.14690685	2.64E-07	L23	LDR30m
**Gm43569**	1.05966096	0.00642603	L23	LDR30m
**Gm45321**	1.07518811	3.86E-05	L23	LDR30m
**Gm45323**	1.07920988	1.20E-05	L23	LDR30m
**Gm47423**	1.88935299	0.03608337	L23	LDR30m
**Gm47644**	1.05337486	0.01771249	L23	LDR30m
**Gm47938**	1.02850207	0.00519149	L23	LDR30m
**Gm50445**	0.75661079	0.00226202	L23	LDR30m

Development • Supplementary information

**Gm6225**	1.18190856	2.15E-07	L23	LDR30m
**Gm7467**	0.69500748	0.00205945	L23	LDR30m
**Gmeb1**	0.67591077	2.18E-05	L23	LDR30m
**Gmeb2**	0.77942303	5.73E-06	L23	LDR30m
**Gnai3**	1.27267504	2.23E-15	L23	LDR30m
**Gnao1**	0.5910778	0.02899945	L23	LDR30m
**Gng2**	0.95707608	1.66E-18	L23	LDR30m
**Gng4**	0.96346642	0.00040542	L23	LDR30m
**Gnl1**	0.96340437	0.00290252	L23	LDR30m
**Golph3**	1.0444606	5.80E-09	L23	LDR30m
**Got1**	0.75703371	3.60E-07	L23	LDR30m
**Gpr161**	1.15173827	0.00067642	L23	LDR30m
**Gpr19**	1.20003614	1.98E-25	L23	LDR30m
**Gpt2**	0.97003935	2.10E-19	L23	LDR30m
**Grhl1**	1.27663754	1.98E-16	L23	LDR30m
**Grm4**	1.06284504	0.00012792	L23	LDR30m
**Gtpbp4**	0.70009029	0.00386823	L23	LDR30m
**Gxylt1**	0.82903249	1.18E-05	L23	LDR30m
**H13**	0.73135491	0.00021759	L23	LDR30m
**Hcfc2**	0.76206962	3.88E-06	L23	LDR30m
**Hdac5**	1.08855922	5.70E-13	L23	LDR30m
**Heatr3**	0.64729225	0.01299166	L23	LDR30m
**Heca**	0.96395777	3.22E-14	L23	LDR30m
**Hmgcr**	1.03368802	0.01275203	L23	LDR30m
**Hmgxb3**	0.78202151	3.96E-09	L23	LDR30m
**Hnrnpll**	1.10356167	9.70E-07	L23	LDR30m
**Homer1**	1.85416626	1.96E-16	L23	LDR30m
**Hs3st2**	1.27805082	5.85E-06	L23	LDR30m
**Hsd17b12**	0.9201729	0.00669962	L23	LDR30m
**Hspa14**	0.87935468	0.00543639	L23	LDR30m
**Hspa4**	1.27294126	3.77E-20	L23	LDR30m
**Hspa4l**	0.79479808	4.26E-07	L23	LDR30m
**Hsph1**	1.07933257	0.00055352	L23	LDR30m
**Igf1r**	0.61146333	0.01015494	L23	LDR30m
**Ilf3**	0.82873958	0.00596897	L23	LDR30m
**Ilrun**	0.80446537	0.0058194	L23	LDR30m
**Ina**	1.5690638	8.37E-11	L23	LDR30m
**Insyn2a**	0.93050433	0.02391989	L23	LDR30m
**Ippk**	0.80753323	0.00127861	L23	LDR30m

Development • Supplementary information

**Iqgap1**	0.85965065	2.47E-05	L23	LDR30m
**Isy1**	0.85158887	0.0018839	L23	LDR30m
**Ivns1abp**	1.15894612	4.72E-20	L23	LDR30m
**Jade2**	0.82504033	0.02315583	L23	LDR30m
**Jak2**	0.66879665	0.00344981	L23	LDR30m
**Jdp2**	1.54443491	8.23E-15	L23	LDR30m
**Jrkl**	0.66959685	0.01631703	L23	LDR30m
**Kcnip2**	0.74487611	0.01156514	L23	LDR30m
**Kctd1**	0.75131691	6.50E-20	L23	LDR30m
**Kctd8**	1.47485909	8.99E-10	L23	LDR30m
**Kdm3a**	0.68426239	6.69E-05	L23	LDR30m
**Kdm6b**	1.54457946	0.0009367	L23	LDR30m
**Kdm6bos**	1.12778607	9.07E-07	L23	LDR30m
**Kdm7a**	1.71258046	2.92E-26	L23	LDR30m
**Khdrbs1**	0.61704286	5.21E-07	L23	LDR30m
**Kif18a**	1.0604213	0.00174117	L23	LDR30m
**Kif5c**	0.64958615	2.54E-07	L23	LDR30m
**Kitl**	0.88003307	4.46E-09	L23	LDR30m
**Kmt2a**	0.74218718	1.27E-07	L23	LDR30m
**Kpna1**	0.87846171	1.22E-09	L23	LDR30m
**Kras**	1.19223469	9.92E-18	L23	LDR30m
**L3mbtl4**	0.96187787	5.55E-10	L23	LDR30m
**Lemd3**	1.19565526	2.81E-15	L23	LDR30m
**Lmbr1l**	1.02950661	1.55E-06	L23	LDR30m
**Lncpint**	1.11569191	8.83E-25	L23	LDR30m
**Lnx2**	0.95571972	0.04394229	L23	LDR30m
**Lonp2**	1.30231449	2.26E-42	L23	LDR30m
**Lonrf1**	1.42035618	2.75E-32	L23	LDR30m
**Lonrf3**	1.07703147	1.33E-05	L23	LDR30m
**Lrrc28**	0.70786369	0.00013997	L23	LDR30m
**Lrrc8a**	0.96225684	0.00136303	L23	LDR30m
**Lrrk2**	0.8886014	3.71E-20	L23	LDR30m
**Lzts3**	0.66000575	5.62E-06	L23	LDR30m
**Mal2**	0.84318646	0.00025273	L23	LDR30m
**Maml1**	0.9566749	1.52E-05	L23	LDR30m
**Mamld1**	0.72942703	0.00283186	L23	LDR30m
**Map3k14**	1.08548834	4.61E-07	L23	LDR30m
**Map9**	0.84779192	3.52E-05	L23	LDR30m
**Mapk1ip1l**	0.65562778	0.00966907	L23	LDR30m

Development • Supplementary information

**Mapk4**	0.72346599	1.88E-09	L23	LDR30m
**Mapre1**	0.73500198	1.24E-05	L23	LDR30m
**Mapre2**	0.61028056	0.00052809	L23	LDR30m
**11-Mar**	0.59696412	0.0008259	L23	LDR30m
**7-Mar**	0.64682532	0.02934882	L23	LDR30m
**Mark3**	0.6093475	7.37E-11	L23	LDR30m
**Mb21d2**	0.84772315	0.04195923	L23	LDR30m
**Mbnl1**	0.78687034	1.66E-14	L23	LDR30m
**Mbnl2**	0.72092833	2.44E-16	L23	LDR30m
**Mbp**	1.46399484	9.80E-12	L23	LDR30m
**Med13**	0.59988957	0.00730406	L23	LDR30m
**Med14**	1.61058812	2.17E-25	L23	LDR30m
**Medag**	0.77849522	0.01821754	L23	LDR30m
**Mef2d**	0.81353782	2.24E-09	L23	LDR30m
**Mei4**	0.94726003	0.0034573	L23	LDR30m
**Mest**	1.44423388	5.85E-10	L23	LDR30m
**Mfhas1**	0.71927333	7.64E-09	L23	LDR30m
**Mgrn1**	1.08200932	3.29E-31	L23	LDR30m
**Mia3**	1.11965387	2.81E-15	L23	LDR30m
**Mindy3**	0.71161641	0.00088872	L23	LDR30m
**Minpp1**	0.74413475	0.00440527	L23	LDR30m
**Mknk2**	1.06695658	2.55E-05	L23	LDR30m
**Mlxip**	0.82955551	1.87E-11	L23	LDR30m
**Mn1**	1.32553056	1.08E-16	L23	LDR30m
**Mnt**	1.01152189	3.21E-08	L23	LDR30m
**Mon2**	0.86632717	3.36E-08	L23	LDR30m
**Mpc1**	0.83474058	0.03969011	L23	LDR30m
**Mpp2**	0.80582417	4.02E-05	L23	LDR30m
**Mrpl48**	0.78707007	0.00525528	L23	LDR30m
**Msrb3**	0.66243247	0.00611633	L23	LDR30m
**Mtf1**	0.91768209	2.37E-11	L23	LDR30m
**Mthfr**	0.82320344	6.69E-05	L23	LDR30m
**Mtmr12**	0.73462013	6.49E-13	L23	LDR30m
**Mtmr6**	0.64020316	0.04790554	L23	LDR30m
**Mxi1**	1.07086046	3.78E-15	L23	LDR30m
**Myh9**	1.19560121	3.06E-09	L23	LDR30m
**Mylip**	0.79476446	0.00569215	L23	LDR30m
**Myo1e**	1.03232835	0.00130378	L23	LDR30m
**N4bp2**	0.98296829	0.00342936	L23	LDR30m

**Naa60**	0.71792653	0.00277056	L23	LDR30m
**Nap1l1**	1.16091108	2.84E-28	L23	LDR30m
**Ndel1**	1.10233322	1.37E-12	L23	LDR30m
**Neat1**	1.04955431	0.03237231	L23	LDR30m
**Nectin1**	1.05831249	0.00350455	L23	LDR30m
**Nfkb1**	0.62384752	1.18E-06	L23	LDR30m
**Nlgn2**	0.70446062	0.02830057	L23	LDR30m
**Nmnat2**	1.07699955	6.98E-21	L23	LDR30m
**Nolc1**	1.08233378	8.10E-08	L23	LDR30m
**Nop53**	1.0177976	0.00616771	L23	LDR30m
**Nos1**	1.09239996	0.00134878	L23	LDR30m
**Npnt**	0.68699806	2.87E-07	L23	LDR30m
**Nr1d2**	0.78823858	3.51E-07	L23	LDR30m
**Nr4a1**	0.98588833	0.01673054	L23	LDR30m
**Nr4a2**	2.93787971	0.00608612	L23	LDR30m
**Nr4a3**	3.03748941	0.02987454	L23	LDR30m
**Nrd1**	1.58420612	5.17E-12	L23	LDR30m
**Nrip1**	0.87658204	8.57E-12	L23	LDR30m
**Nrip3**	0.91913647	1.37E-05	L23	LDR30m
**Ntrk2**	1.07716483	6.70E-21	L23	LDR30m
**Nudt3**	0.67350931	0.00036584	L23	LDR30m
**Nudt4**	1.24695258	4.44E-14	L23	LDR30m
**Numbl**	1.00165504	2.89E-19	L23	LDR30m
**Nup98**	0.74933965	1.06E-10	L23	LDR30m
**Opa3**	0.93564901	0.00010554	L23	LDR30m
**Orai1**	1.0537893	0.01762	L23	LDR30m
**Osbpl11**	0.77867201	2.05E-05	L23	LDR30m
**Osbpl8**	1.05437484	3.72E-36	L23	LDR30m
**Otof**	0.97263512	0.00010704	L23	LDR30m
**Pak1**	0.99152048	1.11E-07	L23	LDR30m
**Parm1**	1.08566902	0.00305444	L23	LDR30m
**Pcsk1**	2.39129029	5.80E-05	L23	LDR30m
**Pcyt1a**	0.80920671	0.0061432	L23	LDR30m
**Pea15a**	0.60467378	0.00623084	L23	LDR30m
**Peli1**	0.64672688	0.00632476	L23	LDR30m
**Per1**	1.74603994	0.00506889	L23	LDR30m
**Per2**	1.30161419	1.51E-09	L23	LDR30m
**Pfkfb4**	0.65898266	0.0042042	L23	LDR30m
**Piga**	1.05360238	0.00061926	L23	LDR30m

Development • Supplementary information

**Pik3c2a**	0.76136869	4.98E-08	L23	LDR30m
**Pip5k1a**	0.76518803	5.42E-12	L23	LDR30m
**Pisd**	0.61243947	0.0001073	L23	LDR30m
**Pitpna**	1.08130158	2.57E-27	L23	LDR30m
**Pitpnc1**	0.96168455	0.0207692	L23	LDR30m
**Plagl1**	0.99314904	5.00E-08	L23	LDR30m
**Plat**	0.86095207	0.00667354	L23	LDR30m
**Plcd1**	1.32484343	1.03E-22	L23	LDR30m
**Plcxd2**	1.51404562	4.09E-12	L23	LDR30m
**Plekhg5**	0.73746718	5.17E-06	L23	LDR30m
**Plk3**	1.18837872	7.08E-10	L23	LDR30m
**Plxnc1**	0.78996722	2.16E-07	L23	LDR30m
**Por**	1.24088704	2.58E-22	L23	LDR30m
**Pou6f1**	0.77615619	4.48E-11	L23	LDR30m
**Ppard**	1.10189972	1.06E-06	L23	LDR30m
**Ppfia1**	0.69163087	8.06E-09	L23	LDR30m
**Pphln1**	0.78160902	0.03508428	L23	LDR30m
**Ppm1d**	0.89198602	0.00011865	L23	LDR30m
**Ppp1cc**	0.83218188	0.00030691	L23	LDR30m
**Ppp1r11**	0.95948379	0.035499	L23	LDR30m
**Ppp2ca**	0.97166693	8.35E-09	L23	LDR30m
**Ppp2r1b**	0.93298212	0.02656205	L23	LDR30m
**Ppp6c**	0.61002045	0.02381615	L23	LDR30m
**Pprc1**	1.01292523	3.14E-07	L23	LDR30m
**Pptc7**	0.75454268	5.26E-05	L23	LDR30m
**Prag1**	0.92604506	3.54E-07	L23	LDR30m
**Prdm2**	0.65844333	1.96E-05	L23	LDR30m
**Prim2**	0.99948299	4.92E-08	L23	LDR30m
**Prkag1**	0.74549455	0.00026221	L23	LDR30m
**Prkar2a**	1.0116692	5.35E-12	L23	LDR30m
**Prkce**	0.6472873	0.0209978	L23	LDR30m
**Prkx**	0.91435042	0.00043667	L23	LDR30m
**Psd3**	0.84189348	3.11E-06	L23	LDR30m
**Psen1**	0.87337142	1.09E-10	L23	LDR30m
**Psme4**	0.64833002	7.20E-08	L23	LDR30m
**Ptges3**	0.78225614	0.00154818	L23	LDR30m
**Ptk2b**	0.62259076	1.10E-07	L23	LDR30m
**Ptprm**	1.46543173	0.04306653	L23	LDR30m
**Pvr**	1.28551915	5.69E-15	L23	LDR30m

**Pygb**	0.86050734	5.46E-07	L23	LDR30m
**Qk**	0.77581811	0.0064301	L23	LDR30m
**R3hdm2**	0.63959987	5.84E-06	L23	LDR30m
**Rab10**	0.58866879	1.93E-05	L23	LDR30m
**Rab3b**	1.17502672	3.77E-12	L23	LDR30m
**Rab43**	0.67345747	0.00892716	L23	LDR30m
**Rab6a**	0.7640106	0.00050967	L23	LDR30m
**Rab6b**	1.03226106	1.15E-09	L23	LDR30m
**Rab7**	0.73255379	3.69E-07	L23	LDR30m
**Rabgef1**	1.01066286	1.82E-13	L23	LDR30m
**Rap1b**	0.81757451	1.61E-05	L23	LDR30m
**Rapgef2**	0.79462134	2.36E-09	L23	LDR30m
**Rapgef6**	0.7198957	4.77E-14	L23	LDR30m
**Rbms2**	1.16758182	6.44E-14	L23	LDR30m
**Rcc2**	1.51722125	9.05E-34	L23	LDR30m
**Rcor1**	0.70993081	1.96E-11	L23	LDR30m
**Rflnb**	0.66910514	0.00015601	L23	LDR30m
**Rgs7bp**	0.76122044	2.66E-13	L23	LDR30m
**Rheb**	1.54983428	0.0114597	L23	LDR30m
**Rhot2**	1.070315	4.25E-13	L23	LDR30m
**Rims3**	1.04536821	9.81E-21	L23	LDR30m
**Rims4**	1.26746584	1.11E-15	L23	LDR30m
**Rnf19a**	0.74029725	7.78E-12	L23	LDR30m
**Rnf20**	0.84176778	5.42E-08	L23	LDR30m
**Rnf217**	1.77615512	1.35E-38	L23	LDR30m
**Rnf38**	0.59093493	0.00872082	L23	LDR30m
**Rock2**	0.75688884	4.24E-05	L23	LDR30m
**Rspry1**	0.63095669	0.00015022	L23	LDR30m
**Rtn4rl2**	0.94107926	0.00010769	L23	LDR30m
**Rundc1**	0.9845547	0.00077692	L23	LDR30m
**Rybp**	0.79077508	0.00433272	L23	LDR30m
**Safb2**	0.7544543	0.00082283	L23	LDR30m
**Samd4b**	0.73151315	0.00243275	L23	LDR30m
**Samd8**	1.01490961	1.88E-08	L23	LDR30m
**Sap130**	0.62361084	0.00019141	L23	LDR30m
**Sash1**	0.89279689	0.03927694	L23	LDR30m
**Scarb2**	0.7010159	0.01124129	L23	LDR30m
**Scg3**	0.89212713	0.02730525	L23	LDR30m
**Scyl2**	0.76799615	0.00613058	L23	LDR30m

Development • Supplementary information

**Sdcbp**	0.85491967	0.02909779	L23	LDR30m
**Sec24a**	1.16080939	1.20E-14	L23	LDR30m
**Sema6a**	0.88037369	1.34E-05	L23	LDR30m
**Senp2**	0.7175246	4.37E-09	L23	LDR30m
**7-Sep**	0.80464959	1.74E-11	L23	LDR30m
**9-Sep**	0.97068754	0.01230355	L23	LDR30m
**Serpini1**	0.59718746	0.00299197	L23	LDR30m
**Setd7**	0.88888211	5.80E-06	L23	LDR30m
**Sf1**	0.63096341	7.89E-06	L23	LDR30m
**Sft2d1**	0.85725414	6.14E-05	L23	LDR30m
**Sgsm1**	0.62483981	0.03294236	L23	LDR30m
**Sh3gl3**	0.79716721	0.00216598	L23	LDR30m
**Shc4**	1.15679293	0.00041264	L23	LDR30m
**Shkbp1**	0.83907925	0.00102796	L23	LDR30m
**Shoc2**	0.65940711	1.27E-06	L23	LDR30m
**Siah2**	1.23976402	1.52E-10	L23	LDR30m
**Siah3**	1.01976001	0.00157411	L23	LDR30m
**Sidt1**	1.09835183	1.10E-16	L23	LDR30m
**Sik2**	2.11569479	2.64E-24	L23	LDR30m
**Sik3**	1.21199951	6.88E-16	L23	LDR30m
**Ski**	1.1674202	3.98E-08	L23	LDR30m
**Skil**	1.17150065	4.68E-06	L23	LDR30m
**Slc1a1**	0.79384088	1.62E-06	L23	LDR30m
**Slc20a2**	0.95423504	3.65E-08	L23	LDR30m
**Slc25a25**	1.60631734	6.11E-13	L23	LDR30m
**Slc2a1**	1.16096798	4.40E-06	L23	LDR30m
**Slc2a3**	0.64574406	0.00082759	L23	LDR30m
**Slc35e1**	0.98172663	0.03588141	L23	LDR30m
**Slc45a4**	0.89849538	0.04339166	L23	LDR30m
**Slc4a7**	0.70246261	1.08E-06	L23	LDR30m
**Slc6a17**	0.70865	4.74E-07	L23	LDR30m
**Slc7a1**	1.16558692	3.95E-11	L23	LDR30m
**Slk**	0.63310343	0.02521548	L23	LDR30m
**Smad1**	1.4835407	5.94E-16	L23	LDR30m
**Smap2**	0.75606016	1.22E-06	L23	LDR30m
**Smarca5**	1.03710365	4.94E-09	L23	LDR30m
**Smg7**	1.09847631	6.02E-23	L23	LDR30m
**Snx25**	0.81695202	4.03E-11	L23	LDR30m
**Snx4**	0.72635868	0.00207131	L23	LDR30m

Development • Supplementary information

**Socs7**	0.59117839	6.90E-05	L23	LDR30m
**Sorcs2**	0.6053589	0.00061291	L23	LDR30m
**Spata16**	0.59060022	0.00013809	L23	LDR30m
**Spen**	0.88917728	7.11E-11	L23	LDR30m
**Spock2**	1.04879828	1.04E-20	L23	LDR30m
**Spred2**	0.80325255	1.01E-06	L23	LDR30m
**Sptbn2**	1.03717985	2.24E-08	L23	LDR30m
**Sptbn4**	0.58851104	0.00033089	L23	LDR30m
**Spty2d1**	1.0424001	0.0001405	L23	LDR30m
**Srrm4**	0.94363308	1.85E-12	L23	LDR30m
**St8sia5**	0.6701203	6.52E-07	L23	LDR30m
**Stat3**	1.12696697	1.26E-15	L23	LDR30m
**Stau1**	0.79916632	1.72E-11	L23	LDR30m
**Stk10**	0.97181477	1.79E-09	L23	LDR30m
**Stk24**	0.78884836	4.24E-05	L23	LDR30m
**Stk35**	0.84810158	0.00468478	L23	LDR30m
**Stk38l**	0.95884545	2.35E-09	L23	LDR30m
**Stk4**	0.78822998	5.24E-13	L23	LDR30m
**Stk40**	1.59522337	2.64E-15	L23	LDR30m
**Stx1b**	0.91912125	1.74E-09	L23	LDR30m
**Suco**	0.96494179	3.66E-06	L23	LDR30m
**Sufu**	0.76704553	0.01778797	L23	LDR30m
**Sult2b1**	0.9238908	0.00058602	L23	LDR30m
**Supt6**	0.65849798	0.0001732	L23	LDR30m
**Susd6**	0.6420511	0.02808655	L23	LDR30m
**Synj2**	1.07081391	6.65E-13	L23	LDR30m
**Syt4**	0.94682619	0.00342356	L23	LDR30m
**Taf1**	0.79372062	2.65E-15	L23	LDR30m
**Tbc1d1**	1.33056422	4.29E-23	L23	LDR30m
**Tbc1d16**	1.12265075	0.00920639	L23	LDR30m
**Tbc1d9**	0.90147291	1.62E-08	L23	LDR30m
**Tchh**	0.74268082	0.01633632	L23	LDR30m
**Tet2**	0.64778583	0.00065031	L23	LDR30m
**Tet3**	0.77901534	4.41E-06	L23	LDR30m
**Tfdp1**	0.69308455	0.01211447	L23	LDR30m
**Tfrc**	0.86460506	4.46E-09	L23	LDR30m
**Thbs3**	0.9201648	0.0001376	L23	LDR30m
**Thegl**	1.11795541	0.00020659	L23	LDR30m
**Tm9sf3**	0.9271235	7.79E-07	L23	LDR30m

Development • Supplementary information

**Tmem117**	0.82494773	0.0082763	L23	LDR30m
**Tmem150c**	0.89812941	0.00038812	L23	LDR30m
**Tmem38b**	1.11154608	1.94E-12	L23	LDR30m
**Tnfrsf21**	0.95129764	0.00065801	L23	LDR30m
**Tnfrsf23**	1.02562807	0.02654544	L23	LDR30m
**Tnks1bp1**	0.76018761	1.42E-05	L23	LDR30m
**Tollip**	0.99622597	0.00022181	L23	LDR30m
**Tomm40**	0.69549047	6.88E-05	L23	LDR30m
**Top1**	0.72272314	4.15E-11	L23	LDR30m
**Tor1aip1**	0.8307137	7.73E-06	L23	LDR30m
**Tpm3**	1.06458861	1.75E-30	L23	LDR30m
**Trak1**	1.27603286	1.20E-25	L23	LDR30m
**Trim71**	1.10727555	0.00026455	L23	LDR30m
**Trim9**	0.79486339	4.60E-19	L23	LDR30m
**Tsc22d2**	0.81182264	0.00118454	L23	LDR30m
**Tshz2**	1.26757632	8.04E-06	L23	LDR30m
**Tspan31**	1.03370114	0.01988055	L23	LDR30m
**Ttbk1**	1.10648447	2.07E-07	L23	LDR30m
**Ttc28**	1.21772012	4.00E-11	L23	LDR30m
**Ttpal**	1.17134092	4.01E-14	L23	LDR30m
**Tulp4**	1.14215304	3.33E-17	L23	LDR30m
**Tut7**	0.61213966	0.01000071	L23	LDR30m
**Txndc11**	1.19376216	2.94E-38	L23	LDR30m
**Txndc12**	0.94978745	1.13E-08	L23	LDR30m
**Txnrd1**	1.36567905	1.32E-26	L23	LDR30m
**Uba6**	0.95948119	0.00037763	L23	LDR30m
**Ubald1**	1.0505896	0.0005001	L23	LDR30m
**Ube2b**	0.64544774	1.51E-09	L23	LDR30m
**Ube2f**	0.6295904	0.01003182	L23	LDR30m
**Ube2h**	0.69386801	6.27E-05	L23	LDR30m
**Ube2ql1**	1.70692565	3.86E-13	L23	LDR30m
**Ubl3**	0.74494265	1.80E-05	L23	LDR30m
**Ubn1**	0.59968237	5.10E-05	L23	LDR30m
**Ubqln1**	0.74163591	0.00082552	L23	LDR30m
**Ubtd2**	1.15718227	2.25E-07	L23	LDR30m
**Ulk1**	0.90654653	0.01599924	L23	LDR30m
**Unc45a**	1.01127716	2.15E-07	L23	LDR30m
**Usp25**	0.59734794	1.01E-05	L23	LDR30m
**Usp36**	1.12238684	6.63E-20	L23	LDR30m

Development • Supplementary information

**Usp38**	0.99157059	1.55E-15	L23	LDR30m
**Usp9x**	0.76222791	3.12E-08	L23	LDR30m
**Vcl**	0.76416946	0.00053332	L23	LDR30m
**Vmp1**	0.89632185	2.77E-08	L23	LDR30m
**Vwc2l**	0.8938408	0.00108006	L23	LDR30m
**Wdfy2**	0.82886972	4.85E-10	L23	LDR30m
**Wdr1**	1.04553587	6.71E-06	L23	LDR30m
**Wdr26**	0.73557172	2.68E-05	L23	LDR30m
**Wdr44**	0.75432447	9.06E-05	L23	LDR30m
**Wdr45b**	0.94002514	6.86E-13	L23	LDR30m
**Ybx3**	0.70360205	1.08E-07	L23	LDR30m
**Ywhab**	0.77954459	0.00010566	L23	LDR30m
**Ywhaz**	0.62878451	0.00093558	L23	LDR30m
**Zbed5**	0.72150806	0.03389034	L23	LDR30m
**Zbtb11**	1.10964743	5.88E-21	L23	LDR30m
**Zbtb16**	0.82866606	0.04890159	L23	LDR30m
**Zbtb2**	0.83848273	0.00046923	L23	LDR30m
**Zbtb4**	0.92619193	1.37E-14	L23	LDR30m
**Zbtb7c**	0.81855897	0.01236395	L23	LDR30m
**Zc3h12c**	0.80395956	1.56E-07	L23	LDR30m
**Zdbf2**	2.07122016	2.17E-05	L23	LDR30m
**Zfp184**	1.07891596	0.01088799	L23	LDR30m
**Zfp426**	0.79204854	0.00365195	L23	LDR30m
**Zfp516**	1.12520014	8.18E-09	L23	LDR30m
**Zfp948**	1.10146133	4.21E-06	L23	LDR30m
**Zhx2**	1.16799543	7.73E-19	L23	LDR30m
**Zkscan3**	0.63036689	7.18E-05	L23	LDR30m
**Zmym5**	0.62332908	0.01685316	L23	LDR30m
**Zswim6**	1.34865536	2.83E-23	L23	LDR30m
**Zwint**	0.78047155	6.14E-06	L23	LDR30m
**1600020E01Rik**	0.9518051	1.04E-11	L23	LDR4h
**1810030O07Rik**	0.86930054	0.00075119	L23	LDR4h
**2510009E07Rik**	1.05274472	6.28E-08	L23	LDR4h
**4930519K11Rik**	0.99851126	0.00693234	L23	LDR4h
**A730060N03Rik**	0.96492382	0.0003364	L23	LDR4h
**Acsl5**	0.77247818	0.00012173	L23	LDR4h
**Adgrd1**	1.13631847	6.40E-08	L23	LDR4h
**Adora1**	0.88390674	0.00128997	L23	LDR4h
**Agap2**	0.62889729	0.0117908	L23	LDR4h

**Ago2**	0.72942592	5.94E-09	L23	LDR4h
**Akap13**	0.96241881	0.0010846	L23	LDR4h
**Ank1**	0.78691282	0.03888066	L23	LDR4h
**Ankrd33b**	0.79039653	4.45E-08	L23	LDR4h
**Anks1**	0.77164494	2.08E-07	L23	LDR4h
**Anxa11**	1.19326561	5.97E-07	L23	LDR4h
**Arap2**	1.05666345	2.18E-08	L23	LDR4h
**Arhgef7**	0.76025508	1.37E-09	L23	LDR4h
**Arid3b**	1.02421126	0.00253007	L23	LDR4h
**Arih2**	0.63973273	0.00017636	L23	LDR4h
**Atp11a**	0.64413867	0.03040898	L23	LDR4h
**Atxn10**	0.81488475	1.11E-18	L23	LDR4h
**Azin1**	0.62176202	0.00411642	L23	LDR4h
**B4galt1**	0.92438764	0.00164397	L23	LDR4h
**Baiap2**	0.69902497	4.92E-06	L23	LDR4h
**Baz1a**	1.89223105	3.69E-07	L23	LDR4h
**Bcl2**	0.67782069	0.01412079	L23	LDR4h
**Bcl6**	1.03896957	0.00049005	L23	LDR4h
**Bcl9**	0.60711856	0.0002694	L23	LDR4h
**Bcor**	0.81346343	2.36E-06	L23	LDR4h
**Brinp1**	0.67672325	4.68E-12	L23	LDR4h
**Cbarp**	0.73858256	0.04891342	L23	LDR4h
**Cbfa2t3**	0.7318429	0.04992214	L23	LDR4h
**Cbfb**	0.94003443	0.00051023	L23	LDR4h
**Cbln2**	1.24828011	2.45E-16	L23	LDR4h
**Ccdc134**	1.05024963	0.00027772	L23	LDR4h
**Ccdc6**	0.84245122	4.09E-08	L23	LDR4h
**Cdh22**	0.94030458	9.62E-06	L23	LDR4h
**Cdk13**	0.63297932	4.68E-18	L23	LDR4h
**Cds1**	0.6165289	0.00072067	L23	LDR4h
**Cdyl**	0.78745391	0.00058536	L23	LDR4h
**Cecr2**	1.0058913	0.00074385	L23	LDR4h
**Celf6**	0.8910824	0.00012222	L23	LDR4h
**Cep85l**	0.70640766	0.00017284	L23	LDR4h
**Chd7**	1.00408888	0.0002525	L23	LDR4h
**Chrm2**	1.15733464	4.18E-06	L23	LDR4h
**Chst8**	1.08251818	0.00038242	L23	LDR4h
**Cinp**	0.70300984	0.04223724	L23	LDR4h
**Clcn5**	0.69872176	0.02899552	L23	LDR4h

Development • Supplementary information

**Clip2**	0.66629366	0.00079031	L23	LDR4h
**Clstn3**	0.858392	3.55E-05	L23	LDR4h
**Cmip**	0.81804119	1.98E-08	L23	LDR4h
**Cntnap5c**	0.7783844	0.00410325	L23	LDR4h
**Col28a1**	0.68179279	0.03950292	L23	LDR4h
**Cop1**	0.84985658	1.12E-19	L23	LDR4h
**Cramp1l**	0.58793855	1.28E-09	L23	LDR4h
**Csnk1a1**	0.6242885	3.36E-08	L23	LDR4h
**Csrnp1**	1.09609516	0.0055841	L23	LDR4h
**Ctps**	0.80752986	0.00046989	L23	LDR4h
**D730045A05Rik**	1.10909701	1.14E-05	L23	LDR4h
**Ddah1**	1.03759038	0.00044635	L23	LDR4h
**Dek**	0.7236675	0.00035017	L23	LDR4h
**Dgki**	0.70648725	5.25E-07	L23	LDR4h
**Dgkz**	0.8180168	4.38E-11	L23	LDR4h
**Diaph3**	1.00196213	0.00035719	L23	LDR4h
**Disp3**	1.45195983	1.44E-14	L23	LDR4h
**Dlg1**	0.61100218	4.33E-13	L23	LDR4h
**Dlgap2**	0.58692705	0.0001312	L23	LDR4h
**Dlgap4**	0.8832175	4.95E-11	L23	LDR4h
**Dnajc1**	0.76304936	1.74E-10	L23	LDR4h
**Dot1l**	0.98968587	1.03E-11	L23	LDR4h
**Dpy19l3**	0.86317109	1.71E-07	L23	LDR4h
**E130304I02Rik**	0.98084211	3.84E-07	L23	LDR4h
**Ece1**	0.94761431	7.67E-12	L23	LDR4h
**Egln1**	0.70767396	1.15E-21	L23	LDR4h
**Ehd3**	0.87278648	0.01344841	L23	LDR4h
**Eif3h**	0.5974441	0.00514039	L23	LDR4h
**Elf2**	0.79798767	3.91E-12	L23	LDR4h
**Elmo1**	2.04180822	6.40E-06	L23	LDR4h
**Eml5**	1.03314247	1.12E-10	L23	LDR4h
**Entpd7**	0.80057473	4.85E-07	L23	LDR4h
**Epb41l1**	0.92493967	7.49E-15	L23	LDR4h
**Epha10**	0.75055701	5.30E-06	L23	LDR4h
**Eprs**	0.94805893	1.49E-19	L23	LDR4h
**Evl**	0.58580052	0.00355292	L23	LDR4h
**Fam129b**	0.87610604	0.00019438	L23	LDR4h
**Fam171a1**	0.68373794	8.49E-09	L23	LDR4h
**Fbrsl1**	0.66821502	7.68E-10	L23	LDR4h

Development • Supplementary information

**Fkbp1a**	0.67393942	0.02838285	L23	LDR4h
**Fmnl1**	1.21058234	1.04E-09	L23	LDR4h
**Fmr1**	0.7057961	0.002101	L23	LDR4h
**Fndc3a**	0.910032	1.07E-12	L23	LDR4h
**Frmd6**	1.12211688	3.25E-10	L23	LDR4h
**Fstl4**	0.7745392	0.0021175	L23	LDR4h
**Furin**	1.01419773	0.00015348	L23	LDR4h
**Galnt7**	0.79934726	0.0005609	L23	LDR4h
**Gap43**	0.60100146	0.02806001	L23	LDR4h
**Gdpd5**	1.00132527	0.0025431	L23	LDR4h
**Gfod1**	0.86836861	4.82E-15	L23	LDR4h
**Gm15477**	0.98621524	0.00247899	L23	LDR4h
**Gm2164**	1.09223234	1.05E-16	L23	LDR4h
**Gm3294**	0.68910269	0.01020998	L23	LDR4h
**Gm38642**	0.60690503	0.00050216	L23	LDR4h
**Gm45323**	1.06211474	0.00316874	L23	LDR4h
**Gm49959**	1.06142712	0.0007956	L23	LDR4h
**Gm6225**	0.92948351	0.01614501	L23	LDR4h
**Gmeb2**	0.66593857	0.0001327	L23	LDR4h
**Gnb5**	0.80658862	0.00062046	L23	LDR4h
**Gramd1a**	0.68610146	9.44E-05	L23	LDR4h
**Gramd1b**	1.01683381	1.45E-17	L23	LDR4h
**Gramd4**	0.7982981	4.67E-08	L23	LDR4h
**Grb2**	1.04126083	1.19E-09	L23	LDR4h
**Grik3**	1.10794205	0.00422585	L23	LDR4h
**Grm4**	1.03239329	0.00044882	L23	LDR4h
**Heatr5a**	0.85997378	0.00022853	L23	LDR4h
**Hectd2**	0.98366742	5.00E-05	L23	LDR4h
**Homer1**	0.88463817	0.00175128	L23	LDR4h
**Hs3st2**	1.0466301	0.00320485	L23	LDR4h
**Hs6st2**	0.7209508	7.38E-12	L23	LDR4h
**Hsd17b12**	1.05363379	2.44E-07	L23	LDR4h
**Hsph1**	0.95269468	0.0089004	L23	LDR4h
**Htr1b**	1.05468025	0.00139327	L23	LDR4h
**Igsf3**	1.19674437	9.30E-06	L23	LDR4h
**Inhba**	0.79716512	0.00083606	L23	LDR4h
**Inpp5a**	0.72885437	8.44E-09	L23	LDR4h
**Insyn2a**	0.92515956	0.00313396	L23	LDR4h
**Ipcef1**	0.64786268	0.00476392	L23	LDR4h

Development • Supplementary information

**Ipmk**	0.87271564	0.00644795	L23	LDR4h
**Ipo5**	0.63970549	0.0163809	L23	LDR4h
**Itgav**	0.96407018	3.17E-10	L23	LDR4h
**Jak1**	0.58976259	9.77E-08	L23	LDR4h
**Jak2**	0.60573211	7.79E-06	L23	LDR4h
**Jarid2**	0.83638912	1.08E-09	L23	LDR4h
**Jcad**	0.66920957	0.01554628	L23	LDR4h
**Jdp2**	1.15384441	9.08E-08	L23	LDR4h
**Kcnip2**	0.76925993	3.45E-05	L23	LDR4h
**Kdm6b**	1.43443754	0.01196127	L23	LDR4h
**Kdm7a**	0.7547857	0.00053132	L23	LDR4h
**Klf5**	0.90855238	0.00587364	L23	LDR4h
**Kmt2a**	0.60682474	7.67E-05	L23	LDR4h
**Lhfpl3**	0.65662274	0.00030544	L23	LDR4h
**Lingo1**	0.58816594	0.00209287	L23	LDR4h
**Lncpint**	0.84845713	5.83E-15	L23	LDR4h
**Lpp**	0.81113175	8.64E-05	L23	LDR4h
**Lrrk2**	1.06854203	8.28E-35	L23	LDR4h
**Maf**	0.84665778	0.01236001	L23	LDR4h
**Maml2**	1.06262157	0.00276145	L23	LDR4h
**Mamld1**	0.82723094	1.09E-07	L23	LDR4h
**Man1a**	0.95662766	8.23E-07	L23	LDR4h
**Map2k3**	0.82545076	0.00683736	L23	LDR4h
**Map3k14**	0.97775206	0.00052382	L23	LDR4h
**Mapk4**	1.27293381	1.99E-27	L23	LDR4h
**Mapk6**	0.94448429	0.03042338	L23	LDR4h
**Mark4**	0.64189339	0.02408747	L23	LDR4h
**Mbp**	0.86523492	0.01332955	L23	LDR4h
**Med13**	0.62848812	2.78E-05	L23	LDR4h
**Med14**	0.7749864	6.83E-05	L23	LDR4h
**Megf11**	1.66596864	1.63E-11	L23	LDR4h
**Mia3**	0.66696469	0.00024642	L23	LDR4h
**Mical2**	0.78470734	0.02603216	L23	LDR4h
**Mrpl48**	0.87069351	2.35E-06	L23	LDR4h
**Mtss1**	0.97027326	6.14E-05	L23	LDR4h
**Myo1e**	0.94169535	0.02383974	L23	LDR4h
**Myo9b**	0.63435429	7.44E-08	L23	LDR4h
**Naa25**	0.64260916	0.00018069	L23	LDR4h
**Nap1l1**	0.90235632	5.70E-22	L23	LDR4h

Development • Supplementary information

**Nav1**	0.66577667	0.00088952	L23	LDR4h
**Neat1**	1.19740228	3.02E-10	L23	LDR4h
**Nectin1**	0.99280608	0.0213017	L23	LDR4h
**Neto1**	0.71660889	5.39E-07	L23	LDR4h
**Nfatc1**	1.06397699	1.45E-05	L23	LDR4h
**Nfia**	0.63165455	0.04636661	L23	LDR4h
**Nfkb1**	0.61508196	6.05E-10	L23	LDR4h
**Nmnat2**	0.70902179	2.78E-09	L23	LDR4h
**Npnt**	0.69998224	0.00264435	L23	LDR4h
**Nptx2**	1.1413275	1.02E-05	L23	LDR4h
**Nrd1**	1.03808395	2.88E-06	L23	LDR4h
**Nrsn1**	0.75692829	1.65E-05	L23	LDR4h
**Nrxn2**	0.92322538	3.16E-16	L23	LDR4h
**Nsun2**	0.605131	3.24E-06	L23	LDR4h
**Nt5dc3**	0.94665004	2.45E-09	L23	LDR4h
**Ntrk2**	1.17015356	2.49E-27	L23	LDR4h
**Ntrk3**	0.59746756	0.00073804	L23	LDR4h
**Nuak1**	0.58929262	0.00132493	L23	LDR4h
**Nudt3**	0.63089779	0.00046661	L23	LDR4h
**Osbpl11**	0.76699064	1.81E-08	L23	LDR4h
**Osbpl3**	1.08711003	3.14E-08	L23	LDR4h
**Osbpl8**	0.65170096	5.55E-20	L23	LDR4h
**Pag1**	0.90343313	3.61E-12	L23	LDR4h
**Pak6**	0.82137328	2.06E-05	L23	LDR4h
**Pawr**	1.10262945	8.15E-08	L23	LDR4h
**Pdgfa**	0.90630638	0.00087701	L23	LDR4h
**Peak1**	0.68836403	0.00444383	L23	LDR4h
**Peli1**	0.75578471	3.37E-13	L23	LDR4h
**Per2**	1.10689712	2.27E-07	L23	LDR4h
**Pgap1**	0.75282247	1.62E-07	L23	LDR4h
**Phf21b**	1.50018231	1.17E-09	L23	LDR4h
**Pip5k1b**	0.75219886	1.41E-09	L23	LDR4h
**Plcd1**	0.99080781	0.00131944	L23	LDR4h
**Plce1**	1.03616398	0.00531698	L23	LDR4h
**Plcl1**	0.79985135	0.00018857	L23	LDR4h
**Pld5**	1.28452054	3.73E-07	L23	LDR4h
**Plk3**	1.02652846	0.01485696	L23	LDR4h
**Plxna2**	0.59109192	0.00038967	L23	LDR4h
**Plxnc1**	0.69356976	5.95E-07	L23	LDR4h

Development • Supplementary information

**Por**	0.94502722	1.20E-06	L23	LDR4h
**Pou6f1**	0.67858775	6.98E-11	L23	LDR4h
**Ppard**	0.83479302	0.01974346	L23	LDR4h
**Ppm1h**	0.75238858	1.00E-05	L23	LDR4h
**Ppme1**	0.71589249	0.02919583	L23	LDR4h
**Ppp1r16b**	0.77044784	0.00207781	L23	LDR4h
**Ppp1r37**	0.63511898	2.65E-06	L23	LDR4h
**Ppp2r1b**	0.9226144	0.00472805	L23	LDR4h
**Ppp2r5a**	0.64242966	0.0002442	L23	LDR4h
**Prim2**	1.60665299	1.68E-23	L23	LDR4h
**Prkar2a**	1.0112364	1.15E-10	L23	LDR4h
**Prkcb**	0.59752312	0.00462334	L23	LDR4h
**Prkce**	0.59713198	0.03042551	L23	LDR4h
**Prkg2**	1.22806116	2.58E-08	L23	LDR4h
**Prmt8**	0.85634769	4.92E-12	L23	LDR4h
**Ptch1**	0.67784277	0.00086038	L23	LDR4h
**Ptprg**	0.67633042	0.00010677	L23	LDR4h
**Rab15**	0.69682979	6.87E-10	L23	LDR4h
**Rab6b**	0.66526559	2.99E-05	L23	LDR4h
**Rai1**	0.60839387	1.11E-06	L23	LDR4h
**Ranbp2**	0.6298465	0.00801818	L23	LDR4h
**Rapgef5**	0.88256422	5.22E-16	L23	LDR4h
**Rbm11**	0.71388265	0.0119114	L23	LDR4h
**Rcor1**	0.62573982	1.02E-06	L23	LDR4h
**Retreg1**	1.04163096	1.20E-24	L23	LDR4h
**Rgs20**	1.10400982	0.00113297	L23	LDR4h
**Rhoq**	1.00939283	8.09E-12	L23	LDR4h
**Rhot2**	0.78119043	0.00133934	L23	LDR4h
**Rims3**	0.77609126	2.63E-11	L23	LDR4h
**Rnf217**	0.80303892	5.48E-07	L23	LDR4h
**Rock2**	0.72264437	1.06E-07	L23	LDR4h
**Rph3a**	1.07191892	3.22E-08	L23	LDR4h
**Rps6ka2**	0.64849779	6.11E-06	L23	LDR4h
**Samd4**	0.91982426	1.19E-06	L23	LDR4h
**Sbk1**	1.1242618	0.00124146	L23	LDR4h
**Scg3**	0.89039721	0.00096817	L23	LDR4h
**Scube1**	1.25829824	4.77E-07	L23	LDR4h
**Sema3f**	1.03062797	0.01334846	L23	LDR4h
**Sgsm1**	1.19030371	7.76E-16	L23	LDR4h

Development • Supplementary information

**Sh3pxd2b**	0.75182602	0.00349505	L23	LDR4h
**Sik2**	1.81934916	4.31E-18	L23	LDR4h
**Sik3**	1.25163979	2.24E-21	L23	LDR4h
**Slc20a2**	0.77264296	1.02E-06	L23	LDR4h
**Slc2a13**	0.75598895	3.98E-06	L23	LDR4h
**Slc38a1**	0.62254429	0.00290256	L23	LDR4h
**Slc4a7**	0.63724921	6.44E-07	L23	LDR4h
**Slc6a17**	0.95626918	2.25E-16	L23	LDR4h
**Slc9a5**	1.1790441	1.55E-20	L23	LDR4h
**Slco3a1**	1.00772918	0.02195455	L23	LDR4h
**Slk**	0.60590409	0.00022947	L23	LDR4h
**Smad3**	0.68732861	0.00243206	L23	LDR4h
**Smc1a**	0.71391164	0.0007767	L23	LDR4h
**Smg7**	0.68648686	5.72E-10	L23	LDR4h
**Snx25**	0.84983087	7.45E-18	L23	LDR4h
**Sorcs3**	1.16587006	1.59E-14	L23	LDR4h
**Spa17**	0.59203729	0.00135382	L23	LDR4h
**Spata5**	0.63840019	7.53E-14	L23	LDR4h
**Spred2**	0.93731822	3.13E-14	L23	LDR4h
**St8sia5**	0.71784172	7.32E-07	L23	LDR4h
**Stk10**	0.92158327	4.36E-13	L23	LDR4h
**Stk40**	1.01852889	0.0017927	L23	LDR4h
**Stx1b**	0.75226965	7.40E-05	L23	LDR4h
**Stx4a**	0.87847185	5.90E-05	L23	LDR4h
**Sv2c**	1.09697276	0.00231352	L23	LDR4h
**Synj2**	0.84894341	9.65E-08	L23	LDR4h
**Tanc1**	0.80459885	0.00060938	L23	LDR4h
**Tbc1d1**	0.89404371	2.16E-13	L23	LDR4h
**Tdg**	0.61271237	0.02607113	L23	LDR4h
**Tet3**	1.01960924	7.64E-11	L23	LDR4h
**Tex2**	0.6671775	0.00017394	L23	LDR4h
**Tjp2**	0.95587623	2.71E-08	L23	LDR4h
**Tle4**	0.67920576	2.85E-05	L23	LDR4h
**Tmem163**	1.40273451	2.67E-14	L23	LDR4h
**Tmem178**	1.039296	2.52E-28	L23	LDR4h
**Tmtc2**	1.04896904	2.44E-22	L23	LDR4h
**Tnfrsf9**	0.65146511	0.04834422	L23	LDR4h
**Tnks1bp1**	0.75605947	6.32E-08	L23	LDR4h
**Tpm3**	0.79829772	7.00E-18	L23	LDR4h

**Trak1**	0.76619141	1.45E-05	L23	LDR4h
**Trim9**	0.6994231	6.91E-14	L23	LDR4h
**Trpc3**	0.87188363	0.01016492	L23	LDR4h
**Tspan14**	0.62680391	0.04239962	L23	LDR4h
**Tulp4**	0.87232442	2.64E-14	L23	LDR4h
**Ube2f**	0.62064334	4.85E-05	L23	LDR4h
**Ubtf**	0.67062648	0.00013197	L23	LDR4h
**Upf1**	0.78810327	0.03366604	L23	LDR4h
**Vmp1**	0.65480835	7.59E-05	L23	LDR4h
**Wdfy1**	0.61546069	1.52E-05	L23	LDR4h
**Wdfy2**	0.68543614	8.86E-05	L23	LDR4h
**Xpo1**	0.74243948	1.31E-07	L23	LDR4h
**Zbtb16**	0.95368008	1.08E-10	L23	LDR4h
**Zc3h12c**	0.89299105	3.28E-20	L23	LDR4h
**Zdhhc14**	0.932363	0.00418801	L23	LDR4h
**Zfc3h1**	0.66356477	0.00014165	L23	LDR4h
**Zhx2**	0.73223062	1.06E-05	L23	LDR4h
**Zmiz1**	1.11922411	3.46E-08	L23	LDR4h
**Zswim6**	1.06836039	6.41E-13	L23	LDR4h
**Gm30094**	1.13039232	0.01184019	L23	LDR6h
**Ntrk2**	-0.6488939	1.66E-06	L23	LDR6h
**Sgsm1**	-0.6513208	0.00015831	L23	LDR6h
**Tspan11**	0.62778198	0.0285748	L23	LDR6h
**Ablim3**	0.8547268	0.00530704	L4	LDR
**Adamtsl1**	1.01663086	7.32E-05	L4	LDR
**Baz1a**	0.64670481	0.00144394	L4	LDR
**Clmn**	0.91130342	0.01767024	L4	LDR
**Crim1**	0.62841786	0.04185864	L4	LDR
**Kctd8**	1.05187312	6.14E-06	L4	LDR
**Mgat5b**	0.77742867	0.00476003	L4	LDR
**Ptprm**	1.03466641	2.15E-09	L4	LDR
**Rasal1**	0.79277663	0.00309385	L4	LDR
**Sash1**	0.83514521	0.00135432	L4	LDR
**Sgcd**	0.90835806	9.02E-06	L4	LDR
**Tmem150c**	0.81059344	0.00023474	L4	LDR
**Vwc2l**	0.79175899	0.00117713	L4	LDR
**A4galt**	1.09047255	0.02533679	L4	LDR2h
**Antxr2**	1.66093888	1.00E-09	L4	LDR2h
**Arhgap5**	0.63814891	0.01672534	L4	LDR2h

Development • Supplementary information

**Bach2os**	1.06506207	0.00149091	L4	LDR2h
**Baz1a**	1.51904349	6.95E-08	L4	LDR2h
**Diras2**	0.75721171	0.00615538	L4	LDR2h
**Dot1l**	0.8455882	0.00884344	L4	LDR2h
**Fat1**	1.01444938	0.00289508	L4	LDR2h
**Gadd45g**	1.22647551	2.19E-05	L4	LDR2h
**Gm10309**	1.24989444	4.75E-13	L4	LDR2h
**Gm26652**	0.87752323	0.0011892	L4	LDR2h
**Gnal**	1.03066962	1.30E-08	L4	LDR2h
**Inhba**	0.8698749	0.00081225	L4	LDR2h
**Megf11**	1.77767236	0.00559884	L4	LDR2h
**Mir670hg**	1.53851619	1.43E-07	L4	LDR2h
**Nrsn1**	0.70521363	0.0103033	L4	LDR2h
**Pdgfc**	1.0596782	0.04105018	L4	LDR2h
**Ptchd4**	0.6934789	0.00771849	L4	LDR2h
**Ptgs2**	1.1705576	0.00109388	L4	LDR2h
**Rhbdf1**	1.13336175	0.00169158	L4	LDR2h
**Slc4a10**	0.64460186	0.04174703	L4	LDR2h
**Smoc2**	0.9983609	0.0013113	L4	LDR2h
**1600020E01Rik**	1.39637195	1.45E-32	L4	LDR30m
**1700016P03Rik**	2.12453583	0.0121381	L4	LDR30m
**2810455O05Rik**	1.01958508	4.58E-05	L4	LDR30m
**2900026A02Rik**	0.7535499	0.00433429	L4	LDR30m
**4931406P16Rik**	0.81598031	3.15E-05	L4	LDR30m
**5031439G07Rik**	0.63460115	0.0096857	L4	LDR30m
**Abca5**	0.59830878	6.92E-05	L4	LDR30m
**Abhd2**	1.34823406	2.68E-18	L4	LDR30m
**Abr**	0.62348771	0.01133609	L4	LDR30m
**Acsl4**	1.03448531	5.61E-10	L4	LDR30m
**Acss1**	1.03044541	1.93E-13	L4	LDR30m
**Adamts17**	0.6707828	0.00201972	L4	LDR30m
**Adamtsl1**	0.97883116	0.00389528	L4	LDR30m
**Adar**	0.7065106	0.00405704	L4	LDR30m
**Adgrb2**	0.65797846	3.14E-09	L4	LDR30m
**Adnp**	0.72544862	0.0123071	L4	LDR30m
**Adora1**	1.53405676	4.23E-28	L4	LDR30m
**Adra1d**	1.06364451	8.35E-07	L4	LDR30m
**Aftph**	0.59479084	0.00213636	L4	LDR30m
**Agap2**	0.80808195	0.00041793	L4	LDR30m

Development • Supplementary information

**Agap3**	1.04480163	9.41E-12	L4	LDR30m
**Ago2**	1.13911909	2.79E-18	L4	LDR30m
**Ago3**	0.82223452	1.83E-11	L4	LDR30m
**Ajap1**	1.14432369	2.04E-12	L4	LDR30m
**Ak4**	1.46433681	3.41E-25	L4	LDR30m
**Aldh18a1**	0.96510174	0.00019685	L4	LDR30m
**Ank**	1.25300835	1.41E-19	L4	LDR30m
**Ankrd17**	0.67625955	1.60E-08	L4	LDR30m
**Ankrd33b**	1.32672226	8.39E-21	L4	LDR30m
**Anks1**	1.38709202	2.53E-21	L4	LDR30m
**Ap2b1**	0.7768341	2.56E-11	L4	LDR30m
**Arf3**	0.74776618	4.53E-07	L4	LDR30m
**Arhgap1**	0.89518302	6.92E-10	L4	LDR30m
**Arhgap23**	0.88262046	2.55E-08	L4	LDR30m
**Arhgef3**	1.44781566	1.13E-07	L4	LDR30m
**Arhgef7**	0.81437825	4.50E-11	L4	LDR30m
**Arid1a**	0.61566755	0.01760991	L4	LDR30m
**Arid3b**	1.09222486	5.03E-07	L4	LDR30m
**Arid5a**	1.06424548	0.00819516	L4	LDR30m
**Arih1**	0.83917801	1.75E-17	L4	LDR30m
**Arih2**	0.89515813	1.01E-11	L4	LDR30m
**Arl5b**	1.69597281	1.89E-17	L4	LDR30m
**Arpc2**	0.98815473	4.34E-12	L4	LDR30m
**Atl2**	0.73083034	2.24E-05	L4	LDR30m
**Atp11a**	0.72497512	0.00422254	L4	LDR30m
**Atp6v0d1**	0.84413424	3.06E-11	L4	LDR30m
**Atp6v0e**	0.64868249	0.03316786	L4	LDR30m
**Atp7a**	0.84665224	0.00012716	L4	LDR30m
**Atxn7**	0.63473286	0.00133276	L4	LDR30m
**Bag2**	1.05596593	0.00025823	L4	LDR30m
**Baiap2**	1.6373857	1.97E-18	L4	LDR30m
**Bcar1**	0.90344363	5.28E-06	L4	LDR30m
**Bcl9l**	1.01006666	0.00540536	L4	LDR30m
**Bcor**	1.1903023	1.53E-09	L4	LDR30m
**Bicdl1**	1.02508552	4.35E-08	L4	LDR30m
**Bmp2k**	0.95838753	0.00610049	L4	LDR30m
**Btaf1**	1.00179272	3.01E-12	L4	LDR30m
**C230057M02Rik**	0.63028612	0.00539533	L4	LDR30m
**Cabp1**	0.81653508	5.41E-11	L4	LDR30m

Development • Supplementary information

**Camk1g**	1.11619184	0.00788905	L4	LDR30m
**Caskin1**	0.73552918	0.04091137	L4	LDR30m
**Cbarp**	1.267292	2.33E-13	L4	LDR30m
**Cbfa2t3**	1.15186956	3.39E-05	L4	LDR30m
**Cbfb**	1.02487484	5.97E-09	L4	LDR30m
**Ccdc136**	0.60011712	0.00053018	L4	LDR30m
**Ccdc6**	0.74845231	1.49E-06	L4	LDR30m
**Ccm2**	1.12274798	1.36E-15	L4	LDR30m
**Ccnl1**	0.84683962	1.94E-18	L4	LDR30m
**Cdk13**	0.61980426	6.99E-07	L4	LDR30m
**Celf6**	0.95829659	5.58E-05	L4	LDR30m
**Cerk**	0.86208047	0.00276295	L4	LDR30m
**Chd1**	0.60282415	2.66E-05	L4	LDR30m
**Chp1**	0.67656431	9.18E-07	L4	LDR30m
**Ciart**	1.14917447	0.0009468	L4	LDR30m
**Clip2**	1.28461237	2.68E-18	L4	LDR30m
**Clstn3**	1.07651536	9.83E-12	L4	LDR30m
**Cltc**	0.78097417	1.30E-06	L4	LDR30m
**Cmip**	1.14927618	7.13E-17	L4	LDR30m
**Cnnm1**	0.79360195	1.48E-05	L4	LDR30m
**Coch**	0.63579498	0.00762339	L4	LDR30m
**Coq10b**	0.99372724	0.0026079	L4	LDR30m
**Coro1c**	1.08407347	1.27E-09	L4	LDR30m
**Coro2a**	0.73810802	0.01345521	L4	LDR30m
**Cpeb3**	1.103174	2.74E-08	L4	LDR30m
**Cpeb4**	0.9914853	6.98E-07	L4	LDR30m
**Crem**	1.00520401	2.71E-08	L4	LDR30m
**Crim1**	0.69102166	6.29E-05	L4	LDR30m
**Cry1**	0.96761717	0.01003094	L4	LDR30m
**Cry2**	1.11182122	1.16E-13	L4	LDR30m
**Csdc2**	0.97161844	0.0007906	L4	LDR30m
**Csnk1a1**	0.81548544	1.65E-13	L4	LDR30m
**Csnk1d**	1.06638698	3.71E-07	L4	LDR30m
**Csrp1**	1.10495607	5.30E-07	L4	LDR30m
**Ctnnb1**	0.86329097	8.61E-06	L4	LDR30m
**Ctnnd1**	0.81886392	3.44E-06	L4	LDR30m
**Ctps**	1.28223841	4.06E-19	L4	LDR30m
**Cux2**	0.65894063	0.00556867	L4	LDR30m
**Cwc25**	1.0015743	8.15E-07	L4	LDR30m

Development • Supplementary information

**Cxadr**	0.69786555	0.03933485	L4	LDR30m
**Cystm1**	1.06514185	4.76E-12	L4	LDR30m
**D5Ertd579e**	0.59779885	3.41E-05	L4	LDR30m
**D730045A05Rik**	1.04643298	0.00063571	L4	LDR30m
**D930015M05Rik**	0.9617623	0.04383708	L4	LDR30m
**Dagla**	0.69529841	1.93E-05	L4	LDR30m
**Dapk1**	0.59419748	0.0002547	L4	LDR30m
**Dennd5b**	0.69132981	1.80E-06	L4	LDR30m
**Dexi**	0.74310526	0.00109705	L4	LDR30m
**Dgkh**	0.6720214	5.63E-08	L4	LDR30m
**Diaph1**	0.90961497	2.42E-12	L4	LDR30m
**Dip2a**	0.92051529	2.15E-20	L4	LDR30m
**Dleu2**	0.66155753	0.00257598	L4	LDR30m
**Dlg4**	0.71171434	0.01251733	L4	LDR30m
**Dlgap4**	1.06671377	3.67E-12	L4	LDR30m
**Dnajc1**	1.18360877	8.35E-25	L4	LDR30m
**Dpysl5**	1.11509063	0.04282935	L4	LDR30m
**Dusp14**	1.19652902	2.63E-10	L4	LDR30m
**Dyrk1a**	0.72224248	0.00179135	L4	LDR30m
**E330009J07Rik**	0.80910328	2.03E-14	L4	LDR30m
**Ece1**	1.35659118	3.49E-15	L4	LDR30m
**Efcab14**	0.67828766	4.50E-08	L4	LDR30m
**Efcab6**	0.71064883	0.00050293	L4	LDR30m
**Efhd2**	1.05331804	1.86E-06	L4	LDR30m
**Efr3b**	0.61346074	0.00030808	L4	LDR30m
**Eif2ak3**	1.03215856	6.34E-07	L4	LDR30m
**Elmsan1**	0.94190978	0.00012647	L4	LDR30m
**Elovl5**	1.29789983	4.70E-18	L4	LDR30m
**Elp5**	0.77920393	0.01904064	L4	LDR30m
**Eml4**	0.72162591	6.00E-16	L4	LDR30m
**Epb41l1**	0.81138138	7.98E-09	L4	LDR30m
**Ephb2**	0.67689191	0.03984558	L4	LDR30m
**Eprs**	0.976244	5.03E-18	L4	LDR30m
**Erf**	1.09665546	2.58E-07	L4	LDR30m
**Ergic1**	0.59830106	0.00493905	L4	LDR30m
**Ern1**	1.10090692	7.82E-15	L4	LDR30m
**Etf1**	0.86530846	4.94E-06	L4	LDR30m
**Fam13c**	0.76744613	4.96E-06	L4	LDR30m
**Fam219a**	0.61557413	0.00033897	L4	LDR30m

Development • Supplementary information

**Fam81a**	0.84292608	2.78E-13	L4	LDR30m
**Fam91a1**	0.74569177	0.02849939	L4	LDR30m
**Fbl**	1.04827307	6.53E-17	L4	LDR30m
**Fbrsl1**	0.77550459	1.02E-06	L4	LDR30m
**Fbxo42**	0.68965372	0.001328	L4	LDR30m
**Fnbp1l**	0.75098759	0.00023756	L4	LDR30m
**Foxo3**	0.95433089	4.55E-05	L4	LDR30m
**Frmd6**	1.78401934	2.27E-16	L4	LDR30m
**Fxyd5**	0.71598771	0.02744366	L4	LDR30m
**Fzd3**	0.72417326	2.31E-05	L4	LDR30m
**Gabbr1**	0.76271122	3.02E-08	L4	LDR30m
**Gadd45g**	1.12997557	0.00049462	L4	LDR30m
**Gak**	0.78219406	6.37E-07	L4	LDR30m
**Galnt9**	1.423215	4.01E-08	L4	LDR30m
**Gclc**	0.62641926	0.02410325	L4	LDR30m
**Gfod1**	1.26528106	1.87E-29	L4	LDR30m
**Gm12940**	0.73206595	7.54E-05	L4	LDR30m
**Gm13872**	0.91709309	0.01748808	L4	LDR30m
**Gm15638**	1.2639238	4.69E-11	L4	LDR30m
**Gm27017**	0.92701516	0.00650989	L4	LDR30m
**Gm36371**	0.60986093	0.04242286	L4	LDR30m
**Gm39326**	0.903713	3.04E-06	L4	LDR30m
**Gm42937**	1.09587323	0.0348093	L4	LDR30m
**Gm6225**	1.0339555	3.34E-05	L4	LDR30m
**Gmeb1**	0.59326324	0.01577826	L4	LDR30m
**Gmeb2**	0.85016517	2.51E-10	L4	LDR30m
**Gnai3**	1.06062888	5.89E-09	L4	LDR30m
**Gnao1**	0.59069856	0.04298529	L4	LDR30m
**Gnb5**	0.63735729	0.00131993	L4	LDR30m
**Gng2**	0.81197033	6.22E-13	L4	LDR30m
**Gng4**	1.13092628	1.80E-08	L4	LDR30m
**Got1**	0.66027267	0.02428428	L4	LDR30m
**Gpi1**	0.67944128	0.01459802	L4	LDR30m
**Gpr19**	0.96580548	6.95E-13	L4	LDR30m
**Gpt2**	1.30626571	2.18E-34	L4	LDR30m
**Gramd1a**	0.77328235	0.00669214	L4	LDR30m
**Gramd1b**	0.75146084	8.21E-09	L4	LDR30m
**Grhl1**	0.96224325	0.02877273	L4	LDR30m
**Grm4**	1.46075012	1.74E-12	L4	LDR30m

**H13**	0.63925471	0.00516042	L4	LDR30m
**Hcfc2**	0.78001015	8.34E-10	L4	LDR30m
**Hdac5**	1.25221703	1.31E-14	L4	LDR30m
**Heca**	0.791416	0.00054631	L4	LDR30m
**Hivep1**	0.97596061	2.57E-07	L4	LDR30m
**Hmgxb3**	0.80140911	0.00012273	L4	LDR30m
**Hnrnpll**	1.02674767	2.91E-07	L4	LDR30m
**Homer1**	2.00424938	1.14E-13	L4	LDR30m
**Hrh1**	0.81260872	0.00029708	L4	LDR30m
**Hsd11b1**	1.12033619	6.59E-10	L4	LDR30m
**Hsd17b12**	0.8763433	8.06E-11	L4	LDR30m
**Hsf1**	0.73991994	0.04466839	L4	LDR30m
**Hspa4**	0.8536149	3.67E-08	L4	LDR30m
**Hsph1**	1.26588318	3.51E-11	L4	LDR30m
**Ifrd1**	0.82541434	0.01494169	L4	LDR30m
**Ilrun**	0.71797827	0.00018835	L4	LDR30m
**Insyn2a**	1.01955403	0.00739441	L4	LDR30m
**Ipo5**	0.71007528	3.98E-05	L4	LDR30m
**Iqgap1**	0.76982881	0.00733752	L4	LDR30m
**Ism1**	1.04963974	0.0003554	L4	LDR30m
**Ivns1abp**	0.86315218	2.26E-20	L4	LDR30m
**Jak1**	0.59828189	0.00037801	L4	LDR30m
**Jdp2**	1.34319214	1.58E-12	L4	LDR30m
**Kcnb1**	0.65110356	7.95E-11	L4	LDR30m
**Kcnip2**	1.02654747	0.01565446	L4	LDR30m
**Kcnk12**	0.90285291	0.03861806	L4	LDR30m
**Kctd8**	1.29570892	3.33E-13	L4	LDR30m
**Kdm6b**	1.53067188	9.19E-19	L4	LDR30m
**Kdm6bos**	1.08060885	0.00512078	L4	LDR30m
**Kdm7a**	1.1739093	3.92E-40	L4	LDR30m
**Kif5c**	0.69810399	8.39E-10	L4	LDR30m
**Kmt2a**	0.80092218	6.82E-06	L4	LDR30m
**Kpna1**	0.7464686	0.00123544	L4	LDR30m
**Kras**	0.79566757	4.66E-13	L4	LDR30m
**Lamc1**	0.73910601	7.12E-06	L4	LDR30m
**Lemd3**	0.90514993	9.55E-09	L4	LDR30m
**Lmna**	0.95082918	0.0001065	L4	LDR30m
**Lmtk3**	0.60716433	0.00032115	L4	LDR30m
**Lncpint**	1.14209254	2.73E-20	L4	LDR30m

Development • Supplementary information

**Lonp2**	1.19378855	2.48E-26	L4	LDR30m
**Lonrf1**	0.99860134	4.14E-19	L4	LDR30m
**Lrch1**	0.71164938	0.00013028	L4	LDR30m
**Lrrc75a**	0.91769458	0.01855583	L4	LDR30m
**Lrrc8a**	0.9199305	0.00794407	L4	LDR30m
**Lrrk2**	0.91780739	3.24E-11	L4	LDR30m
**Lzts3**	0.63536023	0.00160763	L4	LDR30m
**Map1b**	0.65423876	1.22E-08	L4	LDR30m
**Map3k13**	0.67764174	0.02941064	L4	LDR30m
**Map3k14**	0.95505076	2.09E-06	L4	LDR30m
**Mapk4**	0.89075386	6.98E-10	L4	LDR30m
**Mapk6**	0.90391665	1.66E-06	L4	LDR30m
**Mapkapk2**	1.07110408	6.20E-08	L4	LDR30m
**11-Mar**	0.71809293	4.84E-07	L4	LDR30m
**3-Mar**	0.92683833	0.00076907	L4	LDR30m
**7-Mar**	0.64008235	0.00989844	L4	LDR30m
**Mbp**	1.4946903	3.02E-10	L4	LDR30m
**Med14**	1.44460855	4.32E-26	L4	LDR30m
**Mef2d**	0.94535078	2.63E-11	L4	LDR30m
**Mest**	1.00862574	1.05E-07	L4	LDR30m
**Mgrn1**	0.91826682	1.69E-23	L4	LDR30m
**Mia3**	0.76574412	9.46E-07	L4	LDR30m
**Mir670hg**	1.42958683	1.33E-21	L4	LDR30m
**Mknk2**	1.09050673	1.85E-05	L4	LDR30m
**Mlxip**	0.78501996	2.61E-12	L4	LDR30m
**Mn1**	1.44282337	2.42E-14	L4	LDR30m
**Mnt**	1.13296881	7.95E-09	L4	LDR30m
**Mon2**	0.82312807	8.53E-13	L4	LDR30m
**Mpc1**	0.79685331	0.00492461	L4	LDR30m
**Mpp2**	0.87443553	1.23E-06	L4	LDR30m
**Mpp7**	0.80351758	0.00131294	L4	LDR30m
**Mrpl48**	0.91465513	8.70E-07	L4	LDR30m
**Mtcl1**	0.74637762	8.80E-05	L4	LDR30m
**Mtf1**	0.78592853	0.01691774	L4	LDR30m
**Mthfd1l**	0.69050863	0.00011682	L4	LDR30m
**Mtmr12**	0.71798891	1.68E-05	L4	LDR30m
**Mxi1**	1.08816322	4.13E-07	L4	LDR30m
**Myh10**	0.70376793	0.00228922	L4	LDR30m
**Myh9**	1.53190252	9.32E-17	L4	LDR30m

Development • Supplementary information

**Myo1e**	1.37532565	2.35E-11	L4	LDR30m
**Naa25**	0.72229874	0.01299306	L4	LDR30m
**Nab1**	1.00062109	3.27E-12	L4	LDR30m
**Nap1l1**	0.97071723	5.61E-22	L4	LDR30m
**Nav1**	0.59952055	0.00231057	L4	LDR30m
**Ncor2**	0.85469735	0.01236533	L4	LDR30m
**Ndel1**	0.74350919	0.00650903	L4	LDR30m
**Neat1**	1.04944881	0.01051928	L4	LDR30m
**Nectin1**	1.53698128	0.00171514	L4	LDR30m
**Nmnat2**	0.85321635	1.84E-10	L4	LDR30m
**Nolc1**	0.93496761	0.00121366	L4	LDR30m
**Noxred1**	1.04960893	0.00079462	L4	LDR30m
**Nr1d2**	0.60946441	0.00040687	L4	LDR30m
**Nr4a1**	1.07940291	1.86E-05	L4	LDR30m
**Nr4a2**	2.49523571	1.94E-05	L4	LDR30m
**Nrd1**	1.37527547	2.63E-19	L4	LDR30m
**Ntrk2**	0.89055168	5.74E-14	L4	LDR30m
**Nuak1**	0.69364322	1.19E-12	L4	LDR30m
**Nudt3**	0.6605728	1.49E-05	L4	LDR30m
**Nudt4**	1.08169072	2.66E-05	L4	LDR30m
**Numbl**	1.15463354	1.63E-15	L4	LDR30m
**Olah**	1.10337307	0.00065575	L4	LDR30m
**Olfm2**	0.83247482	9.83E-11	L4	LDR30m
**Osbpl8**	0.72705192	2.01E-08	L4	LDR30m
**Oxct1**	0.64459272	0.00012676	L4	LDR30m
**P4hb**	1.04400747	1.60E-07	L4	LDR30m
**Paf1**	0.91649346	0.00996615	L4	LDR30m
**Pak1**	0.68888678	2.55E-09	L4	LDR30m
**Pcdh10**	-0.5905296	5.97E-06	L4	LDR30m
**Pcdh17**	0.95357325	6.27E-06	L4	LDR30m
**Pcsk1**	1.86215811	0.00140808	L4	LDR30m
**Pde4a**	0.80585621	0.00023328	L4	LDR30m
**Pdlim1**	1.0616549	0.01585762	L4	LDR30m
**Per2**	1.36127605	3.57E-13	L4	LDR30m
**Pgbd5**	0.69850676	0.00542833	L4	LDR30m
**Phf21b**	1.42030401	1.89E-11	L4	LDR30m
**Pik3r3**	0.74126134	0.00890098	L4	LDR30m
**Pip4k2c**	0.90309046	0.00125719	L4	LDR30m
**Pip5k1a**	0.79037359	5.86E-15	L4	LDR30m

Development • Supplementary information

**Pip5k1c**	0.63775	0.00015578	L4	LDR30m
**Pisd**	0.83569413	1.95E-16	L4	LDR30m
**Pitpna**	0.79112629	6.04E-10	L4	LDR30m
**Plcd1**	1.18278594	1.27E-08	L4	LDR30m
**Plekhb2**	0.73895171	0.03709733	L4	LDR30m
**Plekhg5**	0.97228236	3.97E-09	L4	LDR30m
**Pmepa1**	1.20132607	0.00010347	L4	LDR30m
**Por**	0.88103462	8.86E-07	L4	LDR30m
**Pou6f1**	0.75036007	1.74E-06	L4	LDR30m
**Ppard**	1.09184676	2.83E-10	L4	LDR30m
**Ppargc1b**	0.61658425	0.0427725	L4	LDR30m
**Ppfia1**	0.61671655	0.01139195	L4	LDR30m
**Ppm1h**	0.60160834	0.00946975	L4	LDR30m
**Ppp1cc**	0.86901855	2.49E-07	L4	LDR30m
**Ppp1r37**	0.67968122	0.00449567	L4	LDR30m
**Ppp2ca**	0.86449255	4.75E-06	L4	LDR30m
**Ppp2r1b**	1.1327552	1.10E-08	L4	LDR30m
**Ppp2r2c**	0.72618666	8.08E-05	L4	LDR30m
**Ppp2r5a**	0.7441716	6.18E-06	L4	LDR30m
**Pptc7**	0.76367882	0.02657668	L4	LDR30m
**Prim2**	0.70044854	0.03099827	L4	LDR30m
**Prkar2a**	0.78586095	0.00149284	L4	LDR30m
**Prkce**	0.88882151	1.79E-05	L4	LDR30m
**Psd3**	0.60572395	0.00196231	L4	LDR30m
**Ptk2b**	0.64239892	1.85E-05	L4	LDR30m
**Ptp4a2**	0.72891883	1.13E-06	L4	LDR30m
**Ptpn12**	0.60916297	0.01870594	L4	LDR30m
**Ptprj**	0.67246111	1.80E-09	L4	LDR30m
**Ptprm**	1.03644262	1.07E-07	L4	LDR30m
**Pvr**	1.17355384	3.03E-11	L4	LDR30m
**R3hdm2**	0.77885895	1.93E-14	L4	LDR30m
**Rab3b**	1.22661626	2.08E-17	L4	LDR30m
**Rab40c**	0.77002228	0.03377858	L4	LDR30m
**Rab6b**	0.97658577	9.11E-11	L4	LDR30m
**Rab7**	0.63602695	1.34E-05	L4	LDR30m
**Rabgef1**	0.73699731	6.30E-05	L4	LDR30m
**Rai1**	0.75007791	3.78E-06	L4	LDR30m
**Rangap1**	0.68567488	0.00774017	L4	LDR30m
**Rap1b**	0.85808327	1.27E-10	L4	LDR30m

**Rassf5**	1.17900135	5.74E-11	L4	LDR30m
**Rbbp7**	0.84126445	0.00031361	L4	LDR30m
**Rbm11**	0.68046011	0.00949163	L4	LDR30m
**Rbms2**	1.07089156	9.18E-06	L4	LDR30m
**Rcc2**	1.34043407	2.34E-22	L4	LDR30m
**Rcor1**	0.68257477	3.78E-07	L4	LDR30m
**Relb**	1.13824326	0.00141833	L4	LDR30m
**Rgs7bp**	0.78584196	5.85E-13	L4	LDR30m
**Rheb**	1.40832641	1.03E-06	L4	LDR30m
**Rhot2**	0.81405967	0.00037288	L4	LDR30m
**Rims3**	1.06161106	1.60E-13	L4	LDR30m
**Rims4**	1.14668344	2.46E-07	L4	LDR30m
**Ripor1**	0.78720897	0.00885502	L4	LDR30m
**Rnf19a**	0.62465932	3.46E-06	L4	LDR30m
**Rnf217**	1.13142136	5.27E-27	L4	LDR30m
**Rock2**	0.92675702	9.94E-07	L4	LDR30m
**Rph3a**	0.72841128	0.00046277	L4	LDR30m
**Rundc1**	0.91215243	0.0053149	L4	LDR30m
**Rybp**	0.88029695	0.00023539	L4	LDR30m
**Safb2**	0.66257457	0.00832311	L4	LDR30m
**Samd8**	0.75815725	2.35E-08	L4	LDR30m
**Sash1**	0.77412197	0.0007612	L4	LDR30m
**Sbk1**	1.23837912	9.47E-07	L4	LDR30m
**Scg3**	0.85577732	0.00173599	L4	LDR30m
**Sdk2**	0.93792843	6.46E-05	L4	LDR30m
**Sec14l1**	0.69811944	0.01194946	L4	LDR30m
**Sec24a**	0.82555593	3.74E-06	L4	LDR30m
**9-Sep**	0.94800212	0.00034855	L4	LDR30m
**Serinc3**	0.59860568	0.04934848	L4	LDR30m
**Serpine2**	0.93577479	8.81E-05	L4	LDR30m
**Setd7**	0.65850629	0.02269592	L4	LDR30m
**Sez6l**	0.72323952	0.00976024	L4	LDR30m
**Sft2d1**	0.79673247	0.0065398	L4	LDR30m
**Sfxn3**	0.71805801	0.04486337	L4	LDR30m
**Sgcd**	0.6270061	0.00552209	L4	LDR30m
**Sgsm1**	1.12007346	3.38E-05	L4	LDR30m
**Sh2b3**	0.94384492	0.04291113	L4	LDR30m
**Sh3gl3**	0.70645753	0.01632187	L4	LDR30m
**Sh3pxd2b**	0.99001172	7.15E-09	L4	LDR30m

**Shank3**	0.58881085	4.72E-06	L4	LDR30m
**Shc4**	1.08902384	0.00368543	L4	LDR30m
**Siah2**	1.02907602	4.48E-07	L4	LDR30m
**Sidt1**	0.96404761	8.46E-11	L4	LDR30m
**Sik2**	1.96378024	3.88E-25	L4	LDR30m
**Sik3**	1.04814146	6.50E-12	L4	LDR30m
**Ski**	1.22451217	3.52E-08	L4	LDR30m
**Skil**	1.24620848	1.12E-05	L4	LDR30m
**Slc20a2**	1.1272476	1.73E-10	L4	LDR30m
**Slc24a4**	0.76950796	0.00483189	L4	LDR30m
**Slc25a25**	1.43573625	7.74E-07	L4	LDR30m
**Slc38a1**	0.65987397	0.00250963	L4	LDR30m
**Slc6a17**	0.96139934	3.13E-23	L4	LDR30m
**Slc7a1**	1.19884195	4.55E-14	L4	LDR30m
**Smad1**	1.10363832	5.49E-07	L4	LDR30m
**Smap2**	0.88795179	1.94E-09	L4	LDR30m
**Smg7**	0.93000804	4.02E-18	L4	LDR30m
**Sntb2**	0.86305537	1.20E-10	L4	LDR30m
**Snx25**	0.7726994	0.00010042	L4	LDR30m
**Socs7**	0.71221828	4.28E-06	L4	LDR30m
**Spag9**	0.78909263	2.94E-14	L4	LDR30m
**Spen**	0.73977918	7.77E-06	L4	LDR30m
**Spock2**	0.84456023	1.14E-09	L4	LDR30m
**Spred2**	0.87202928	4.85E-14	L4	LDR30m
**Sptbn2**	0.94322213	3.58E-05	L4	LDR30m
**Srrm4**	0.883544	3.60E-07	L4	LDR30m
**St8sia5**	1.24103073	9.39E-15	L4	LDR30m
**Stat3**	0.90717507	0.0141823	L4	LDR30m
**Stau1**	0.74818575	0.00172006	L4	LDR30m
**Stim2**	0.62428131	0.00241168	L4	LDR30m
**Stk10**	0.96106915	1.59E-09	L4	LDR30m
**Stk38l**	0.76758514	0.00190962	L4	LDR30m
**Stk40**	1.54566025	1.02E-14	L4	LDR30m
**Stx1b**	0.97584309	1.36E-10	L4	LDR30m
**Sult2b1**	1.07879307	6.63E-05	L4	LDR30m
**Sult4a1**	0.70765669	0.00128697	L4	LDR30m
**Supt6**	0.67749643	0.04750844	L4	LDR30m
**Susd6**	0.63571845	9.61E-05	L4	LDR30m
**Synj2**	1.2453181	4.64E-25	L4	LDR30m

Development • Supplementary information

**Syt13**	0.78344622	2.64E-05	L4	LDR30m
**Tacc1**	0.62928637	5.28E-06	L4	LDR30m
**Taf1**	0.60283365	0.00158638	L4	LDR30m
**Tanc1**	0.9061325	1.48E-08	L4	LDR30m
**Tbc1d1**	1.2232162	3.83E-22	L4	LDR30m
**Tbc1d16**	1.19603589	3.51E-09	L4	LDR30m
**Tead1**	0.895993	2.69E-05	L4	LDR30m
**Tet3**	1.23711502	7.88E-22	L4	LDR30m
**Tex2**	0.78700057	2.36E-07	L4	LDR30m
**Tfdp1**	0.67314317	0.03553527	L4	LDR30m
**Tgfbr1**	1.02626853	4.76E-05	L4	LDR30m
**Tm9sf3**	0.89282354	8.87E-12	L4	LDR30m
**Tmc7**	0.86825964	0.01051151	L4	LDR30m
**Tmem178**	0.66645475	1.55E-08	L4	LDR30m
**Tmem38b**	0.94266908	0.00209166	L4	LDR30m
**Tnfaip6**	1.15401563	5.33E-05	L4	LDR30m
**Tnks1bp1**	0.78968826	0.03937615	L4	LDR30m
**Top1**	0.64530851	2.86E-06	L4	LDR30m
**Tpm3**	0.88280151	6.66E-20	L4	LDR30m
**Trak1**	1.09844821	4.42E-14	L4	LDR30m
**Trim71**	1.13912399	1.25E-05	L4	LDR30m
**Trim9**	0.74385531	3.02E-14	L4	LDR30m
**Trp53inp1**	0.66954736	0.03787224	L4	LDR30m
**Ttbk1**	0.96352437	5.78E-09	L4	LDR30m
**Ttc28**	1.18900251	1.25E-12	L4	LDR30m
**Ttpal**	0.91931159	1.44E-07	L4	LDR30m
**Tulp4**	0.84512115	4.12E-17	L4	LDR30m
**Txndc11**	0.81041003	7.53E-15	L4	LDR30m
**Txndc12**	0.75781562	3.21E-06	L4	LDR30m
**Txnrd1**	1.30193641	9.25E-35	L4	LDR30m
**Uba6**	0.75896483	0.03818275	L4	LDR30m
**Ube2f**	0.62649043	0.00306172	L4	LDR30m
**Ube2ql1**	1.40195114	8.31E-14	L4	LDR30m
**Ubl3**	0.72258342	2.17E-06	L4	LDR30m
**Ubqln1**	0.69095806	0.00593661	L4	LDR30m
**Ubtd2**	1.19668779	5.35E-08	L4	LDR30m
**Ubtf**	0.86534428	3.82E-08	L4	LDR30m
**Unc45a**	0.89868737	5.34E-06	L4	LDR30m
**Usp28**	0.88279542	0.00073595	L4	LDR30m

Development • Supplementary information

**Usp36**	1.04695191	5.64E-08	L4	LDR30m
**Usp9x**	0.70986766	1.93E-09	L4	LDR30m
**Vcl**	0.62793706	0.02296064	L4	LDR30m
**Wdfy2**	0.70355321	9.05E-06	L4	LDR30m
**Wdr1**	1.09058014	5.16E-08	L4	LDR30m
**Wdr26**	0.64103383	0.02104368	L4	LDR30m
**Wdr45b**	0.72902635	1.34E-07	L4	LDR30m
**Whrn**	0.70433479	0.04348471	L4	LDR30m
**Ybx3**	0.76156049	4.63E-05	L4	LDR30m
**Ywhaz**	0.60460213	0.01846516	L4	LDR30m
**Zbtb11**	0.82878983	1.46E-14	L4	LDR30m
**Zbtb16**	1.16278016	4.90E-10	L4	LDR30m
**Zbtb2**	0.84093293	0.01514985	L4	LDR30m
**Zbtb4**	0.84982722	3.09E-06	L4	LDR30m
**Zdbf2**	1.70509891	0.000128	L4	LDR30m
**Zdhhc14**	0.81411182	0.02701049	L4	LDR30m
**Zfp516**	1.07130404	2.45E-10	L4	LDR30m
**Zfp948**	0.90841579	0.0002524	L4	LDR30m
**Zhx2**	1.06588301	9.58E-10	L4	LDR30m
**Zswim6**	1.20271933	7.24E-12	L4	LDR30m
**Zwint**	0.71155664	2.05E-05	L4	LDR30m
**1600020E01Rik**	0.99497692	1.16E-15	L4	LDR4h
**2510009E07Rik**	0.6392287	0.00019445	L4	LDR4h
**4930519K11Rik**	1.06534064	0.00067721	L4	LDR4h
**5031439G07Rik**	0.62919453	0.00304125	L4	LDR4h
**6430548M08Rik**	0.74721051	0.003681	L4	LDR4h
**9130017K11Rik**	0.62206207	0.04806927	L4	LDR4h
**9530059O14Rik**	0.79533127	0.00085773	L4	LDR4h
**A4galt**	1.13014234	5.72E-05	L4	LDR4h
**A730060N03Rik**	1.01268298	0.01671947	L4	LDR4h
**A830018L16Rik**	-0.5968187	0.00036428	L4	LDR4h
**Abhd2**	0.65612137	0.02557465	L4	LDR4h
**Actn1**	0.70579714	1.19E-08	L4	LDR4h
**Adam19**	0.72831794	0.04632269	L4	LDR4h
**Adamts19**	1.04242455	0.00062707	L4	LDR4h
**Adgrb2**	0.60191194	8.32E-08	L4	LDR4h
**Adgrd1**	1.0916235	0.00033575	L4	LDR4h
**Adgrl3**	0.69879518	7.20E-08	L4	LDR4h
**Agfg2**	0.59927472	0.00784015	L4	LDR4h

Development • Supplementary information

**Ago2**	0.58563371	0.04046848	L4	LDR4h
**Akap13**	1.13831373	0.0039157	L4	LDR4h
**Akirin1**	0.71597978	0.00065542	L4	LDR4h
**Ankrd33b**	0.78361081	2.11E-06	L4	LDR4h
**Anks1**	0.80490699	3.58E-06	L4	LDR4h
**Antxr2**	1.6275932	3.95E-19	L4	LDR4h
**Anxa11**	1.20374575	0.00019753	L4	LDR4h
**Arhgap1**	0.70680193	0.00038994	L4	LDR4h
**Arhgap10**	0.58593449	0.0208904	L4	LDR4h
**Arhgef7**	0.71235343	3.01E-08	L4	LDR4h
**Arid3b**	0.84356931	0.04469608	L4	LDR4h
**B4galt1**	1.13362437	0.00334288	L4	LDR4h
**Baiap2**	0.80551198	0.00066339	L4	LDR4h
**Baz1a**	1.67960336	4.07E-19	L4	LDR4h
**Bcor**	1.11416579	1.77E-09	L4	LDR4h
**Btbd9**	0.67276318	0.00341965	L4	LDR4h
**Cacng2**	0.81106469	9.39E-07	L4	LDR4h
**Caln1**	0.71407398	0.02818229	L4	LDR4h
**Car10**	0.67418265	1.38E-07	L4	LDR4h
**Cbfa2t3**	1.02554684	0.00689556	L4	LDR4h
**Cbln2**	1.0225853	0.00957806	L4	LDR4h
**Ccdc134**	0.98473855	0.0273445	L4	LDR4h
**Ccdc6**	0.8228003	7.45E-15	L4	LDR4h
**Ccdc88c**	0.81110735	0.00400366	L4	LDR4h
**Ccnf**	1.06038118	0.00331528	L4	LDR4h
**Cdh23**	1.22876119	2.77E-12	L4	LDR4h
**Chst11**	0.7690261	0.00605857	L4	LDR4h
**Clip2**	0.67111877	0.00014545	L4	LDR4h
**Clmp**	0.71019399	0.0408322	L4	LDR4h
**Cmip**	0.7789083	1.84E-09	L4	LDR4h
**Col4a2**	0.81619374	0.00303781	L4	LDR4h
**Col5a1**	1.0138377	0.00190494	L4	LDR4h
**Cop1**	0.63933353	3.27E-12	L4	LDR4h
**Crim1**	0.66591583	0.00020635	L4	LDR4h
**Crtac1**	0.85517707	0.00010321	L4	LDR4h
**Csnk1a1**	0.69060503	4.85E-10	L4	LDR4h
**Cxxc5**	0.78342055	0.0203963	L4	LDR4h
**D730045A05Rik**	1.13486063	8.55E-07	L4	LDR4h
**Dagla**	0.68836942	2.67E-08	L4	LDR4h

Development • Supplementary information

**Dclre1c**	0.63491214	0.00341253	L4	LDR4h
**Ddah1**	1.24976024	3.20E-09	L4	LDR4h
**Dgkz**	0.6265965	0.00055983	L4	LDR4h
**Dip2c**	0.64240244	0.00206706	L4	LDR4h
**Disp3**	1.26754866	3.34E-07	L4	LDR4h
**Dlg4**	0.68818174	0.02936701	L4	LDR4h
**Dlgap2**	0.74351643	0.00016189	L4	LDR4h
**Dlgap4**	0.82341552	2.04E-07	L4	LDR4h
**Dnajc1**	0.70340644	9.52E-10	L4	LDR4h
**Dner**	0.86993302	1.49E-05	L4	LDR4h
**Dot1l**	0.94651115	3.55E-22	L4	LDR4h
**Dpy19l3**	1.06468987	0.00253161	L4	LDR4h
**Dsg2**	0.64876014	0.00084129	L4	LDR4h
**Dvl1**	0.62965039	0.02929475	L4	LDR4h
**Ece1**	0.83847847	0.00021353	L4	LDR4h
**Efr3b**	0.68912719	3.89E-07	L4	LDR4h
**Egln1**	0.69773763	0.0010674	L4	LDR4h
**Eml5**	0.67384761	2.66E-06	L4	LDR4h
**Enox1**	0.74281831	0.00012054	L4	LDR4h
**Epb41l1**	0.73889727	5.81E-12	L4	LDR4h
**Epha10**	0.6187852	6.10E-05	L4	LDR4h
**Fam129b**	1.12756828	7.33E-07	L4	LDR4h
**Fam13c**	1.02766973	1.90E-14	L4	LDR4h
**Fam171a1**	0.78380509	1.35E-11	L4	LDR4h
**Fat1**	0.97871442	2.83E-07	L4	LDR4h
**Fbrsl1**	0.60902654	0.00034303	L4	LDR4h
**Fmnl1**	0.92619905	0.00145567	L4	LDR4h
**Fndc3a**	0.60942085	0.00034171	L4	LDR4h
**Fndc3b**	0.64755551	0.02739087	L4	LDR4h
**Frmd6**	0.91397394	0.02313672	L4	LDR4h
**Fstl4**	0.73437279	0.00995466	L4	LDR4h
**Galnt18**	0.80686161	0.00047109	L4	LDR4h
**Galnt7**	0.87853689	0.02799823	L4	LDR4h
**Galnt9**	1.04439891	0.00257478	L4	LDR4h
**Gap43**	0.72017316	0.04756151	L4	LDR4h
**Garem1**	0.59207049	0.00605873	L4	LDR4h
**Gdpd5**	1.10966624	1.77E-15	L4	LDR4h
**Gfod1**	0.99195306	1.19E-17	L4	LDR4h
**Gm10419**	0.62782457	7.61E-14	L4	LDR4h

**Gm15397**	1.24321521	6.13E-06	L4	LDR4h
**Gm15398**	1.21707909	0.00016522	L4	LDR4h
**Gm15477**	1.07720242	6.14E-08	L4	LDR4h
**Gm2164**	0.98089563	1.31E-08	L4	LDR4h
**Gm32647**	1.06486971	2.54E-05	L4	LDR4h
**Gm36736**	1.00428219	0.02308705	L4	LDR4h
**Gmeb2**	0.7282382	1.53E-06	L4	LDR4h
**Gnb5**	0.59981665	0.00298941	L4	LDR4h
**Gosr2**	0.85761565	0.01510707	L4	LDR4h
**Gramd1b**	0.61393465	2.26E-07	L4	LDR4h
**Gramd4**	0.84139085	4.13E-11	L4	LDR4h
**Grb2**	0.82104144	4.61E-05	L4	LDR4h
**Grik4**	0.96335026	0.0002473	L4	LDR4h
**Grm1**	0.98357491	7.36E-08	L4	LDR4h
**Grm4**	0.95617902	0.00019652	L4	LDR4h
**Gsg1l**	0.92162955	5.19E-08	L4	LDR4h
**Gxylt2**	1.04737723	0.02486976	L4	LDR4h
**Hcfc2**	0.6492145	1.14E-05	L4	LDR4h
**Hectd2**	0.74480346	0.0099797	L4	LDR4h
**Hivep1**	0.85460079	3.85E-05	L4	LDR4h
**Hnrnpll**	0.75215277	8.98E-05	L4	LDR4h
**Hs3st2**	0.85966325	3.88E-05	L4	LDR4h
**Hsd17b12**	0.71635714	1.34E-05	L4	LDR4h
**Hsph1**	0.90131131	0.00237701	L4	LDR4h
**Igf1**	1.10096738	0.00156804	L4	LDR4h
**Igsf9b**	0.72323102	5.14E-06	L4	LDR4h
**Inpp4b**	1.10871017	9.58E-07	L4	LDR4h
**Inpp5a**	0.70914849	0.0381726	L4	LDR4h
**Ipo5**	0.70788167	1.46E-06	L4	LDR4h
**Iqsec3**	0.75311305	0.00192236	L4	LDR4h
**Jarid2**	0.71803136	2.05E-06	L4	LDR4h
**Jdp2**	0.74005017	0.03007628	L4	LDR4h
**Kcnd3**	0.82936177	0.02389963	L4	LDR4h
**Kcnip2**	1.1799115	7.48E-05	L4	LDR4h
**Kcnip3**	0.5882586	0.00166056	L4	LDR4h
**Kdm6b**	1.22147415	9.82E-08	L4	LDR4h
**Kirrel3**	1.13986145	3.08E-06	L4	LDR4h
**Klhl2**	0.72579396	1.18E-14	L4	LDR4h
**Lhfpl3**	0.79062883	6.58E-09	L4	LDR4h

Development • Supplementary information

**Lingo1**	0.70426548	3.02E-08	L4	LDR4h
**Lncpint**	0.78737395	2.35E-11	L4	LDR4h
**Lrfn1**	0.77246802	0.0062535	L4	LDR4h
**Lrrk2**	0.75658806	2.01E-10	L4	LDR4h
**Maf**	0.92956353	0.00018121	L4	LDR4h
**Mamld1**	0.96218904	6.72E-05	L4	LDR4h
**Mapk4**	1.35046138	1.63E-24	L4	LDR4h
**Mapkapk2**	0.88629923	0.01855645	L4	LDR4h
**11-Mar**	0.58979924	0.00260864	L4	LDR4h
**Megf11**	2.20329735	5.36E-08	L4	LDR4h
**Mical2**	0.76625381	0.03724706	L4	LDR4h
**Mir670hg**	1.62605158	5.79E-29	L4	LDR4h
**Mir9-3hg**	0.82090005	0.00059224	L4	LDR4h
**Mmp17**	0.86819767	2.71E-05	L4	LDR4h
**Mpp3**	0.74624876	0.0068858	L4	LDR4h
**Mrpl48**	0.83450172	1.45E-05	L4	LDR4h
**Mthfd1l**	0.68065178	1.67E-06	L4	LDR4h
**Myh9**	0.93842239	0.00085019	L4	LDR4h
**Myo9b**	0.62126397	7.30E-10	L4	LDR4h
**Naa25**	0.6844741	0.01536171	L4	LDR4h
**Nap1l1**	0.64272262	2.32E-08	L4	LDR4h
**Nck2**	0.62049237	0.00048159	L4	LDR4h
**Neto1**	0.71714876	0.00033877	L4	LDR4h
**Noct**	0.75466493	0.00670735	L4	LDR4h
**Nrd1**	0.709876	0.00040687	L4	LDR4h
**Nrp1**	1.40601899	0.0480854	L4	LDR4h
**Nrxn2**	1.24434316	1.80E-13	L4	LDR4h
**Nsun2**	0.65773962	0.00088168	L4	LDR4h
**Ntrk2**	0.93127696	7.01E-18	L4	LDR4h
**Ntrk3**	0.85997995	0.00016476	L4	LDR4h
**Osbpl10**	0.72713708	0.02467868	L4	LDR4h
**Osbpl3**	0.64439225	0.00059436	L4	LDR4h
**Pawr**	1.12370834	4.05E-08	L4	LDR4h
**Paxip1**	0.72415248	0.0401516	L4	LDR4h
**Pcdh15**	1.11205124	4.57E-07	L4	LDR4h
**Pde2a**	0.8035504	0.00219682	L4	LDR4h
**Pde4a**	0.67537837	0.00140377	L4	LDR4h
**Pdgfc**	0.99880217	0.0053447	L4	LDR4h
**Pdzd2**	1.47677603	6.55E-06	L4	LDR4h

Development • Supplementary information

**Peak1**	0.96904289	0.00495622	L4	LDR4h
**Per2**	0.99581274	4.52E-06	L4	LDR4h
**Pgap1**	0.6046123	0.00185308	L4	LDR4h
**Phf21b**	1.18868437	2.68E-07	L4	LDR4h
**Pip5k1b**	0.69856704	0.01037111	L4	LDR4h
**Pisd**	0.60968721	0.00428926	L4	LDR4h
**Pitpnm3**	0.64424686	0.000191	L4	LDR4h
**Plekhg5**	0.70036636	0.00220269	L4	LDR4h
**Plxna4**	0.70946644	4.06E-07	L4	LDR4h
**Ppargc1b**	0.61111451	0.00104499	L4	LDR4h
**Ppm1e**	0.65903063	3.95E-07	L4	LDR4h
**Ppm1h**	1.0916488	2.04E-12	L4	LDR4h
**Ppp1r16b**	0.71720305	1.68E-06	L4	LDR4h
**Ppp1r37**	0.83232287	5.38E-11	L4	LDR4h
**Ppp2r1b**	0.93816651	0.01046248	L4	LDR4h
**Ppp2r5a**	0.60536087	0.02327076	L4	LDR4h
**Prim2**	0.90102583	5.65E-08	L4	LDR4h
**Prkce**	0.71937738	0.01477464	L4	LDR4h
**Prmt8**	0.65839017	0.00273815	L4	LDR4h
**Ptch1**	0.74854751	0.00202619	L4	LDR4h
**Ptk2**	0.59192256	7.37E-08	L4	LDR4h
**Ptprg**	0.64035766	1.89E-05	L4	LDR4h
**Rab15**	0.70425002	4.46E-07	L4	LDR4h
**Rai1**	0.62161418	0.00025583	L4	LDR4h
**Rapgef5**	0.71005157	4.36E-05	L4	LDR4h
**Rhbdf1**	1.10148847	1.17E-06	L4	LDR4h
**Rhoq**	1.01801865	3.74E-08	L4	LDR4h
**Rilpl1**	0.70746347	0.00032384	L4	LDR4h
**Rims3**	0.68673513	1.33E-05	L4	LDR4h
**Rnf166**	0.71207411	2.41E-05	L4	LDR4h
**Rock2**	0.69569957	0.00471065	L4	LDR4h
**Rph3a**	0.63766363	1.31E-05	L4	LDR4h
**Rps6ka2**	0.67445398	0.00085906	L4	LDR4h
**Rps6ka3**	0.59458495	7.98E-06	L4	LDR4h
**Rtn4rl1**	0.77177757	0.03234391	L4	LDR4h
**Scube1**	0.75438081	8.63E-05	L4	LDR4h
**Sec14l2**	0.97828595	1.93E-05	L4	LDR4h
**Sema5a**	1.04479534	0.00069932	L4	LDR4h
**Sema7a**	0.66305972	0.00671083	L4	LDR4h

Development • Supplementary information

**Sgsm1**	1.5324459	8.86E-13	L4	LDR4h
**Sh3bp1**	0.92142489	0.01027419	L4	LDR4h
**Sh3pxd2b**	0.93373677	6.97E-07	L4	LDR4h
**Shank3**	0.59197663	5.02E-07	L4	LDR4h
**Sik2**	1.55136267	1.26E-13	L4	LDR4h
**Sik3**	0.69732516	3.07E-05	L4	LDR4h
**Slc6a17**	0.84728581	1.66E-20	L4	LDR4h
**Slc6a8**	0.82699946	0.00173979	L4	LDR4h
**Slc9a5**	1.339047	1.24E-12	L4	LDR4h
**Slco3a1**	1.04433005	0.04719382	L4	LDR4h
**Smad3**	1.22391999	0.00020584	L4	LDR4h
**Sntb2**	0.71547988	6.99E-07	L4	LDR4h
**Snx25**	0.6887345	0.02171916	L4	LDR4h
**Sorcs3**	1.92068385	2.14E-13	L4	LDR4h
**Spred2**	0.97120404	5.13E-20	L4	LDR4h
**Sptb**	0.69433657	0.00022998	L4	LDR4h
**Sptssb**	0.59158645	0.01882154	L4	LDR4h
**Ssbp3**	0.83700906	0.00224	L4	LDR4h
**St8sia5**	1.13551257	5.69E-11	L4	LDR4h
**Stard5**	0.65149262	0.04611625	L4	LDR4h
**Stim2**	0.63823313	1.31E-05	L4	LDR4h
**Stk10**	0.84239183	9.60E-06	L4	LDR4h
**Stx1b**	0.74560502	0.00030027	L4	LDR4h
**Synj2**	0.82566705	6.66E-07	L4	LDR4h
**Tanc1**	0.83530305	2.46E-07	L4	LDR4h
**Tbc1d1**	0.70030588	3.66E-06	L4	LDR4h
**Tdg**	0.68725081	0.00032051	L4	LDR4h
**Tet3**	0.84744761	1.24E-09	L4	LDR4h
**Tex2**	0.63810559	0.00080092	L4	LDR4h
**Tjp2**	0.88560346	2.70E-06	L4	LDR4h
**Tmem117**	0.64920567	0.0244299	L4	LDR4h
**Tmem163**	1.28589867	1.43E-06	L4	LDR4h
**Tmem178**	0.90965936	1.18E-11	L4	LDR4h
**Tmtc2**	0.98053167	1.66E-09	L4	LDR4h
**Traf3**	0.92231594	0.00173833	L4	LDR4h
**Trak1**	0.59016895	0.01015362	L4	LDR4h
**Trim9**	0.68983705	5.68E-11	L4	LDR4h
**Trpc3**	1.1331338	1.70E-16	L4	LDR4h
**Trpc6**	0.79443751	0.00121571	L4	LDR4h

Development • Supplementary information

**Ttc28**	0.81027321	0.01086432	L4	LDR4h
**Ttll11**	0.67355444	9.88E-07	L4	LDR4h
**Ubtf**	0.76273348	1.45E-06	L4	LDR4h
**Uck2**	0.6775568	6.07E-07	L4	LDR4h
**Xpo1**	0.74083072	1.72E-11	L4	LDR4h
**Xylt1**	0.81548309	2.03E-07	L4	LDR4h
**Zbtb1**	1.06130326	0.0010345	L4	LDR4h
**Zbtb16**	0.89159448	6.84E-08	L4	LDR4h
**Zc3h12c**	0.72580469	0.00030066	L4	LDR4h
**Zfc3h1**	0.64860931	0.00603436	L4	LDR4h
**Zmiz1**	1.34944088	2.02E-06	L4	LDR4h
**Zswim6**	0.77740613	0.00143142	L4	LDR4h
**Echdc2**	1.09828006	0.0068187	L4	LDR6h
**Ntrk2**	-0.7302178	0.00019742	L4	LDR6h
**Ablim3**	0.71554098	0.033453	L5IT	LDR
**BC006965**	1.00270738	0.00135562	L5IT	LDR
**Kirrel3**	0.62740789	0.01162412	L5IT	LDR
**Nos1**	1.09289332	3.80E-05	L5IT	LDR
**Slc24a4**	0.91397284	0.00012788	L5IT	LDR
**Sorcs2**	0.67027214	5.96E-05	L5IT	LDR
**Vwc2l**	0.70632066	0.02927125	L5IT	LDR
**C1ql3**	1.16425157	0.00786133	L5IT	LDR2h
**Gm10309**	1.18728946	0.01127901	L5IT	LDR2h
**Sertad2**	0.74501775	0.00641533	L5IT	LDR2h
**1600020E01Rik**	0.76466606	0.01651807	L5IT	LDR30m
**Abhd2**	1.01920291	2.42E-06	L5IT	LDR30m
**Acss1**	1.1996127	3.53E-05	L5IT	LDR30m
**Adora1**	1.13528098	0.00010248	L5IT	LDR30m
**Ago2**	1.00792892	6.07E-08	L5IT	LDR30m
**Agpat4**	0.6398608	0.04586088	L5IT	LDR30m
**Ak4**	1.08039004	0.00014038	L5IT	LDR30m
**Ank**	0.83208093	3.99E-08	L5IT	LDR30m
**Ankrd33b**	1.00411904	0.00014209	L5IT	LDR30m
**Anks1**	1.04846776	9.95E-07	L5IT	LDR30m
**Ap2b1**	0.59152561	0.0023365	L5IT	LDR30m
**Arhgef3**	0.98451746	8.11E-07	L5IT	LDR30m
**Arid3b**	0.9781948	0.02195936	L5IT	LDR30m
**Arid5b**	0.79882808	0.00143168	L5IT	LDR30m
**Arih1**	0.58572065	2.26E-06	L5IT	LDR30m

Development • Supplementary information

**Arih2**	0.71580614	0.00104337	L5IT	LDR30m
**Arl5b**	1.23678123	0.00134592	L5IT	LDR30m
**Arpc2**	0.7954217	2.47E-05	L5IT	LDR30m
**Baiap2**	1.09959333	8.54E-07	L5IT	LDR30m
**Bicdl1**	0.68532902	1.87E-08	L5IT	LDR30m
**Btaf1**	0.87568133	4.34E-10	L5IT	LDR30m
**Btbd8**	0.62875256	0.01605925	L5IT	LDR30m
**Cabp1**	0.67518901	5.34E-05	L5IT	LDR30m
**Cacna1g**	0.90224094	0.00443744	L5IT	LDR30m
**Cacng3**	0.62625685	0.00012949	L5IT	LDR30m
**Cbarp**	1.00814811	0.00539924	L5IT	LDR30m
**Ccm2**	0.96101776	0.04224496	L5IT	LDR30m
**Cdc27**	0.76289808	1.06E-07	L5IT	LDR30m
**Chgb**	0.89600681	2.97E-06	L5IT	LDR30m
**Clip2**	0.87890128	5.35E-05	L5IT	LDR30m
**Clstn3**	0.8388023	0.00011543	L5IT	LDR30m
**Cltc**	0.74626005	3.76E-05	L5IT	LDR30m
**Cmip**	0.88370805	5.56E-10	L5IT	LDR30m
**Cnnm1**	0.90444912	0.00479367	L5IT	LDR30m
**Coq10b**	0.89298811	0.02387006	L5IT	LDR30m
**Cpeb3**	1.03296623	1.78E-25	L5IT	LDR30m
**Cpeb4**	0.90978591	1.94E-08	L5IT	LDR30m
**Cry2**	0.92524584	1.21E-07	L5IT	LDR30m
**Csnk1a1**	0.59934559	0.00026622	L5IT	LDR30m
**Csnk1d**	0.82464418	0.00619263	L5IT	LDR30m
**Ctnnd1**	0.74854301	4.06E-09	L5IT	LDR30m
**Ctps**	1.12716625	2.73E-07	L5IT	LDR30m
**Cwc25**	1.05464015	0.04790115	L5IT	LDR30m
**Dlgap4**	0.73756673	6.51E-05	L5IT	LDR30m
**Dnajc1**	0.63579898	0.00089144	L5IT	LDR30m
**Dusp14**	1.11250569	2.35E-07	L5IT	LDR30m
**Ece1**	1.09184957	1.68E-07	L5IT	LDR30m
**Efhd2**	1.09823522	0.02160443	L5IT	LDR30m
**Efr3a**	0.69017149	0.00017949	L5IT	LDR30m
**Egr2**	1.19953714	0.02041085	L5IT	LDR30m
**Elmo1**	0.63482603	1.71E-07	L5IT	LDR30m
**Elovl5**	1.30577056	0.00040013	L5IT	LDR30m
**Eprs**	0.78607533	0.02213318	L5IT	LDR30m
**Fam49a**	0.74927258	0.00128246	L5IT	LDR30m

**Fbl**	1.11729217	0.0006088	L5IT	LDR30m
**Fbrsl1**	0.63906214	0.02895649	L5IT	LDR30m
**Fbxo33**	0.91142026	0.00257572	L5IT	LDR30m
**Frmd6**	1.40456764	1.35E-19	L5IT	LDR30m
**Gabbr1**	0.79637717	8.18E-05	L5IT	LDR30m
**Gfod1**	0.95287093	1.61E-06	L5IT	LDR30m
**Gm47423**	1.36884399	1.13E-05	L5IT	LDR30m
**Gnao1**	0.69232957	0.00632097	L5IT	LDR30m
**Gng2**	1.00945148	1.15E-10	L5IT	LDR30m
**Gng4**	1.11987118	0.00336913	L5IT	LDR30m
**Gpt2**	1.04154482	7.72E-05	L5IT	LDR30m
**Grasp**	1.10972804	6.53E-05	L5IT	LDR30m
**Grhl1**	1.18895092	2.12E-06	L5IT	LDR30m
**Homer1**	1.94120774	6.65E-05	L5IT	LDR30m
**Hspa4**	0.76717437	8.49E-07	L5IT	LDR30m
**Hsph1**	1.01346746	0.00400345	L5IT	LDR30m
**Ifrd1**	0.76997121	0.01989423	L5IT	LDR30m
**Jdp2**	0.98504188	1.51E-05	L5IT	LDR30m
**Kcnk12**	0.84682945	0.02698615	L5IT	LDR30m
**Kdm6b**	1.25463995	0.00040881	L5IT	LDR30m
**Kdm7a**	0.82330859	0.00061577	L5IT	LDR30m
**Kirrel3**	0.59090038	0.00823946	L5IT	LDR30m
**Kpna1**	0.71568571	0.00868663	L5IT	LDR30m
**Kras**	0.79499733	0.0001455	L5IT	LDR30m
**Lemd3**	0.91989808	0.00046712	L5IT	LDR30m
**Lncpint**	0.86654235	1.07E-14	L5IT	LDR30m
**Lonp2**	0.69334044	0.00292414	L5IT	LDR30m
**Lonrf1**	0.9536158	0.00010726	L5IT	LDR30m
**Lrrc8c**	0.67753229	0.02469067	L5IT	LDR30m
**Lrrk2**	0.69994698	8.28E-05	L5IT	LDR30m
**Mapk6**	1.11894129	3.73E-05	L5IT	LDR30m
**3-Mar**	1.18320691	1.71E-15	L5IT	LDR30m
**Mbp**	1.10743171	2.89E-05	L5IT	LDR30m
**Med14**	1.31063105	2.87E-22	L5IT	LDR30m
**Mef2d**	0.77048425	6.27E-06	L5IT	LDR30m
**Mest**	1.03933583	9.38E-06	L5IT	LDR30m
**Mgrn1**	0.88212915	2.60E-07	L5IT	LDR30m
**Mn1**	1.43097971	1.55E-06	L5IT	LDR30m
**Mpc1**	0.7043699	0.00828861	L5IT	LDR30m

Development • Supplementary information

**Mxi1**	1.04970049	9.97E-09	L5IT	LDR30m
**Myh10**	0.71378579	0.01451849	L5IT	LDR30m
**Myh9**	1.01242927	0.00016067	L5IT	LDR30m
**Nap1l1**	0.85058204	4.45E-13	L5IT	LDR30m
**Nectin1**	1.27318232	4.10E-06	L5IT	LDR30m
**Nmnat2**	0.70886637	9.47E-08	L5IT	LDR30m
**Nr3c1**	0.64923306	6.55E-05	L5IT	LDR30m
**Nr4a1**	0.93416549	0.000941	L5IT	LDR30m
**Nr4a2**	2.22327557	2.03E-05	L5IT	LDR30m
**Nr4a3**	2.45382285	0.00730465	L5IT	LDR30m
**Nrd1**	0.96898669	1.19E-10	L5IT	LDR30m
**Ntrk2**	0.73244473	6.29E-14	L5IT	LDR30m
**Nudt4**	1.07928134	3.01E-07	L5IT	LDR30m
**Numbl**	0.88500934	0.02731158	L5IT	LDR30m
**Osbpl8**	0.7641129	9.46E-09	L5IT	LDR30m
**Pcsk1**	1.35703262	2.36E-07	L5IT	LDR30m
**Per1**	1.52610304	8.09E-06	L5IT	LDR30m
**Per2**	1.08235225	0.00020542	L5IT	LDR30m
**Pgm2**	1.00626272	1.83E-06	L5IT	LDR30m
**Pip5k1a**	0.6049611	0.02830097	L5IT	LDR30m
**Pitpna**	0.65384664	3.16E-06	L5IT	LDR30m
**Plcxd2**	1.1282476	0.0038366	L5IT	LDR30m
**Pmepa1**	1.06964064	7.77E-05	L5IT	LDR30m
**Ppp2r2a**	0.6284913	0.00191018	L5IT	LDR30m
**Prkar2a**	0.87944334	0.00648226	L5IT	LDR30m
**Psd3**	0.79602001	0.00026118	L5IT	LDR30m
**Rab6b**	0.82917784	0.00090145	L5IT	LDR30m
**Rgs7bp**	0.6575298	0.00294196	L5IT	LDR30m
**Rheb**	1.21203846	6.89E-06	L5IT	LDR30m
**Rims4**	1.12005383	2.06E-05	L5IT	LDR30m
**Rnf217**	0.70567554	0.00054315	L5IT	LDR30m
**Rock2**	0.7639681	9.42E-08	L5IT	LDR30m
**Scg3**	0.85248917	0.01872701	L5IT	LDR30m
**Sec14l1**	0.68477412	0.00507092	L5IT	LDR30m
**7-Sep**	0.64475624	0.00223207	L5IT	LDR30m
**Sidt1**	0.71538752	4.97E-07	L5IT	LDR30m
**Sik1**	1.42346525	1.45E-06	L5IT	LDR30m
**Sik2**	1.69394036	1.00E-54	L5IT	LDR30m
**Sik3**	1.06961558	2.28E-10	L5IT	LDR30m

Development • Supplementary information

**Sipa1l2**	0.88419105	7.70E-05	L5IT	LDR30m
**Ski**	1.11975876	0.0008996	L5IT	LDR30m
**Slc24a4**	1.14027342	1.54E-09	L5IT	LDR30m
**Slc25a25**	1.35150713	4.37E-09	L5IT	LDR30m
**Slc6a17**	0.85367254	5.31E-11	L5IT	LDR30m
**Slc7a1**	0.8858334	0.01614573	L5IT	LDR30m
**Slc7a8**	0.75714713	0.00040523	L5IT	LDR30m
**Smg7**	0.77329319	5.23E-10	L5IT	LDR30m
**Sorcs2**	0.62306949	0.00422719	L5IT	LDR30m
**Spen**	0.77254579	5.85E-05	L5IT	LDR30m
**Srrm4**	0.59559308	0.00197537	L5IT	LDR30m
**St8sia5**	0.70916127	3.19E-08	L5IT	LDR30m
**Stk10**	0.93873671	0.00466625	L5IT	LDR30m
**Stk40**	0.93737582	0.00680426	L5IT	LDR30m
**Synj2**	0.84181603	0.01218447	L5IT	LDR30m
**Taf1**	0.65815418	0.00631992	L5IT	LDR30m
**Tbc1d1**	1.00718158	1.51E-10	L5IT	LDR30m
**Tbc1d9**	0.96993956	5.32E-09	L5IT	LDR30m
**Tet3**	0.70523116	0.02944358	L5IT	LDR30m
**Tiparp**	1.46513432	3.78E-05	L5IT	LDR30m
**Tnfrsf21**	0.81074438	0.0020033	L5IT	LDR30m
**Trim9**	0.72424042	9.79E-08	L5IT	LDR30m
**Ttbk1**	0.8758422	0.02296465	L5IT	LDR30m
**Tulp4**	0.78447985	6.54E-05	L5IT	LDR30m
**Txndc11**	0.69859167	0.00010923	L5IT	LDR30m
**Txnrd1**	1.13091756	2.22E-05	L5IT	LDR30m
**Ube2ql1**	1.27679292	2.99E-07	L5IT	LDR30m
**Ubtd2**	0.95225697	3.07E-06	L5IT	LDR30m
**Usp9x**	0.61308565	0.00174003	L5IT	LDR30m
**Wdr1**	1.05293114	0.0071484	L5IT	LDR30m
**Ybx3**	0.7927769	0.02175844	L5IT	LDR30m
**Zbtb11**	0.69400766	0.00088179	L5IT	LDR30m
**Zbtb16**	0.87274735	5.02E-06	L5IT	LDR30m
**Zdbf2**	1.27780978	4.47E-05	L5IT	LDR30m
**Zswim6**	0.81836607	6.56E-10	L5IT	LDR30m
**1600020E01Rik**	0.76064268	0.00923918	L5IT	LDR4h
**2510009E07Rik**	0.88478293	0.00031634	L5IT	LDR4h
**9530059O14Rik**	0.62081488	0.02052244	L5IT	LDR4h
**Acvr1**	0.60401154	0.00384533	L5IT	LDR4h

Development • Supplementary information

**Ahdc1**	0.79018935	0.02221588	L5IT	LDR4h
**Anxa11**	1.06829812	0.0134285	L5IT	LDR4h
**Arhgap31**	1.21717161	0.00013072	L5IT	LDR4h
**Baz1a**	1.41854675	0.00170296	L5IT	LDR4h
**Bcor**	0.86836948	0.00260628	L5IT	LDR4h
**Cbfa2t3**	0.8677125	0.03422745	L5IT	LDR4h
**Ccdc88c**	0.85770176	0.00028498	L5IT	LDR4h
**Cdh22**	1.19418181	1.04E-06	L5IT	LDR4h
**Cep85l**	0.7663766	0.00114986	L5IT	LDR4h
**Clstn3**	0.62531609	0.04960834	L5IT	LDR4h
**Crtac1**	1.01961105	7.23E-07	L5IT	LDR4h
**Dagla**	0.62653218	0.03182444	L5IT	LDR4h
**Ddah1**	1.35924415	3.87E-15	L5IT	LDR4h
**Dlgap4**	0.74900541	5.74E-08	L5IT	LDR4h
**Dnajc1**	0.63745758	0.00012996	L5IT	LDR4h
**Dot1l**	0.81686278	7.10E-10	L5IT	LDR4h
**Ece1**	0.69526788	0.02816285	L5IT	LDR4h
**Elmo1**	0.86911881	4.14E-09	L5IT	LDR4h
**Eml5**	0.71080531	2.70E-06	L5IT	LDR4h
**Epb41l1**	0.78117092	0.00561521	L5IT	LDR4h
**Fndc3a**	0.62203174	6.63E-05	L5IT	LDR4h
**Frmd6**	0.86675331	0.00041285	L5IT	LDR4h
**Fstl4**	0.65938818	0.00045168	L5IT	LDR4h
**Galnt14**	0.92514496	0.01040921	L5IT	LDR4h
**Gdpd5**	0.84522157	0.00153791	L5IT	LDR4h
**Gfod1**	0.93545902	6.98E-06	L5IT	LDR4h
**Gmeb2**	0.79384413	0.0003857	L5IT	LDR4h
**Gramd1b**	0.59416332	5.11E-07	L5IT	LDR4h
**Itgav**	0.80940488	0.00414474	L5IT	LDR4h
**Kdm6b**	1.23084951	0.00126002	L5IT	LDR4h
**Klhl3**	0.72205793	0.00302269	L5IT	LDR4h
**Kmt2a**	0.59151436	0.01450555	L5IT	LDR4h
**Lncpint**	0.63838138	7.69E-12	L5IT	LDR4h
**Lrrk2**	0.86801381	4.33E-10	L5IT	LDR4h
**Mamld1**	0.98055739	2.07E-08	L5IT	LDR4h
**Mapk4**	1.03436961	2.44E-14	L5IT	LDR4h
**Med14**	0.72644982	0.00091229	L5IT	LDR4h
**Megf11**	1.89833703	6.29E-25	L5IT	LDR4h
**Mrpl48**	0.77683162	0.047328	L5IT	LDR4h

Development • Supplementary information

**Myo9b**	0.60513944	0.01066037	L5IT	LDR4h
**Nap1l1**	0.61926238	3.05E-05	L5IT	LDR4h
**Ncor2**	0.72086618	6.70E-06	L5IT	LDR4h
**Nrd1**	0.58824261	0.00284147	L5IT	LDR4h
**Nrp1**	0.97906189	0.00242771	L5IT	LDR4h
**Nrxn2**	0.83127361	2.74E-11	L5IT	LDR4h
**Nsun2**	0.61013182	0.0447143	L5IT	LDR4h
**Ntrk2**	0.79575647	4.04E-25	L5IT	LDR4h
**Osbpl10**	0.66671595	5.05E-07	L5IT	LDR4h
**Osbpl3**	0.68635852	5.21E-06	L5IT	LDR4h
**Pawr**	1.30298824	4.72E-09	L5IT	LDR4h
**Per2**	0.86900187	0.00706891	L5IT	LDR4h
**Pip5k1c**	0.67981943	0.00972248	L5IT	LDR4h
**Pitpnm3**	0.65370025	0.00290215	L5IT	LDR4h
**Ppm1h**	0.74882456	9.85E-07	L5IT	LDR4h
**Prim2**	0.89186584	0.01359183	L5IT	LDR4h
**Rai1**	0.64315794	1.83E-05	L5IT	LDR4h
**Rock2**	0.68759016	1.38E-05	L5IT	LDR4h
**Scg3**	0.83344099	0.02877497	L5IT	LDR4h
**Sgsm1**	1.22207785	5.51E-08	L5IT	LDR4h
**Sik2**	1.44493574	2.56E-24	L5IT	LDR4h
**Sik3**	0.7859184	3.00E-05	L5IT	LDR4h
**Slc6a17**	0.85211944	9.33E-14	L5IT	LDR4h
**Slc9a5**	0.95723574	0.00275044	L5IT	LDR4h
**Smg7**	0.64647897	7.06E-05	L5IT	LDR4h
**Sorcs1**	0.63155572	0.00345424	L5IT	LDR4h
**Sorcs3**	1.22454856	1.03E-15	L5IT	LDR4h
**Spred2**	0.82317151	2.97E-09	L5IT	LDR4h
**St8sia5**	0.62771374	8.98E-05	L5IT	LDR4h
**Stim2**	0.64751347	0.00723223	L5IT	LDR4h
**Tet3**	0.82892265	1.09E-08	L5IT	LDR4h
**Tmtc2**	0.76192625	0.00018882	L5IT	LDR4h
**Tpm3**	0.63900043	0.00022744	L5IT	LDR4h
**Zbtb16**	0.62275618	9.55E-07	L5IT	LDR4h
**Zmiz1**	1.21991035	5.58E-09	L5IT	LDR4h
**Zswim6**	0.60909754	0.00027875	L5IT	LDR4h
**Kctd8**	1.60818615	0.00176343	L5NP	LDR
**Chgb**	0.8344515	0.00618283	L5NP	LDR30m
**Egr3**	2.00687676	1.86E-08	L5NP	LDR30m

Development • Supplementary information

**Gm47423**	1.43169451	0.01164111	L5NP	LDR30m
**Homer1**	1.8496095	1.31E-11	L5NP	LDR30m
**Kctd8**	1.77248904	0.00092352	L5NP	LDR30m
**Nr4a3**	2.08798285	0.04856892	L5NP	LDR30m
**Nrd1**	0.76786718	0.02184798	L5NP	LDR30m
**Pcsk1**	1.52030625	9.45E-12	L5NP	LDR30m
**Per1**	1.64896616	0.00021405	L5NP	LDR30m
**Sik2**	1.22707718	1.71E-10	L5NP	LDR30m
**Tiparp**	1.71664736	0.00011778	L5NP	LDR30m
**Zdbf2**	1.43150514	0.00104147	L5NP	LDR30m
**Homer1**	0.9399462	0.01291882	L5NP	LDR4h
**Ntrk2**	0.6343472	0.01602393	L5NP	LDR4h
**Sgcd**	0.76696674	9.93E-09	L5NP	LDR4h
**Sik2**	0.92387911	4.99E-07	L5NP	LDR4h
**Spred2**	0.67794328	0.04065067	L5NP	LDR4h
**Erbb4**	-0.6466224	0.00132265	L5NP	LDR6h
**Adamts17**	1.06091074	7.43E-08	L5PT	LDR
**Dapk1**	0.66364288	0.01464171	L5PT	LDR
**Dgkg**	0.7094413	0.00220647	L5PT	LDR
**Fgf1**	-0.646015	0.00756361	L5PT	LDR
**Gm45323**	1.19069425	0.00290273	L5PT	LDR
**Rhbdl3**	0.78722928	5.18E-06	L5PT	LDR
**Robo3**	0.89358951	0.04997175	L5PT	LDR
**Rps6ka2**	1.00156855	0.01855197	L5PT	LDR
**Syt17**	0.99167119	0.00626393	L5PT	LDR
**Baz1a**	1.2558689	0.01919347	L5PT	LDR2h
**Elmo1**	0.92948716	0.03727374	L5PT	LDR2h
**Gm37229**	1.44993001	6.73E-07	L5PT	LDR2h
**Gm45323**	1.22571848	0.02144233	L5PT	LDR2h
**4931406P16Rik**	0.85931084	0.00034154	L5PT	LDR30m
**Adamts17**	1.19052226	1.87E-07	L5PT	LDR30m
**Ago2**	0.85458815	0.01498563	L5PT	LDR30m
**Ak4**	1.25047182	0.00257751	L5PT	LDR30m
**Alk**	-0.687049	4.05E-14	L5PT	LDR30m
**Ankrd33b**	1.01339749	7.77E-08	L5PT	LDR30m
**Anks1**	0.92144842	8.69E-06	L5PT	LDR30m
**Arhgap1**	0.9494498	0.00073547	L5PT	LDR30m
**Arhgef3**	1.30034612	9.43E-11	L5PT	LDR30m
**Arih2**	0.89783858	3.36E-07	L5PT	LDR30m

Development • Supplementary information

**Arl5b**	1.45300212	7.59E-11	L5PT	LDR30m
**Arpc2**	0.92760735	4.39E-08	L5PT	LDR30m
**Atp6v0d1**	0.82945188	0.00288307	L5PT	LDR30m
**Atxn7**	0.74267231	0.00413503	L5PT	LDR30m
**Baiap2**	1.0662359	2.73E-10	L5PT	LDR30m
**Bcl6**	0.79893488	8.14E-06	L5PT	LDR30m
**Bdnf**	1.16423165	0.00439758	L5PT	LDR30m
**Btaf1**	0.80126273	1.92E-05	L5PT	LDR30m
**Cabp1**	0.83906205	8.42E-13	L5PT	LDR30m
**Cdc27**	0.87802946	9.45E-06	L5PT	LDR30m
**Cds1**	0.78988824	4.83E-08	L5PT	LDR30m
**Cep120**	0.84421979	0.00104386	L5PT	LDR30m
**Chgb**	0.99280904	3.78E-05	L5PT	LDR30m
**Ciart**	1.16212061	5.41E-05	L5PT	LDR30m
**Clip2**	0.9279142	1.11E-05	L5PT	LDR30m
**Clstn3**	0.95798428	9.69E-05	L5PT	LDR30m
**Cltc**	0.78018793	2.97E-05	L5PT	LDR30m
**Cmip**	0.78413848	2.19E-05	L5PT	LDR30m
**Cnnm1**	0.7477169	0.00580893	L5PT	LDR30m
**Coq10b**	1.15319117	0.00375231	L5PT	LDR30m
**Cpeb3**	0.98089248	1.46E-07	L5PT	LDR30m
**Cpeb4**	0.81551444	5.81E-06	L5PT	LDR30m
**Cry2**	0.84186339	9.58E-05	L5PT	LDR30m
**Csnk1d**	0.90304401	0.00094963	L5PT	LDR30m
**Cwc25**	1.24863572	3.02E-09	L5PT	LDR30m
**Ddx3y**	0.89933364	0.04846927	L5PT	LDR30m
**Dexi**	0.68671052	0.00435291	L5PT	LDR30m
**Dnajc1**	0.68451038	3.29E-05	L5PT	LDR30m
**Ece1**	1.1027515	4.38E-10	L5PT	LDR30m
**Egr3**	1.78690712	0.04720284	L5PT	LDR30m
**Eif2ak3**	0.92381999	0.0132183	L5PT	LDR30m
**Elovl5**	1.22062987	9.91E-10	L5PT	LDR30m
**Elovl6**	0.64241711	0.0399522	L5PT	LDR30m
**Eprs**	1.16538861	3.55E-11	L5PT	LDR30m
**Ern1**	0.93355274	0.00117683	L5PT	LDR30m
**Fam91a1**	1.22821474	2.66E-07	L5PT	LDR30m
**Fbl**	1.1400939	1.79E-05	L5PT	LDR30m
**Fbrsl1**	0.75724082	0.00116537	L5PT	LDR30m
**Fbxo33**	1.10226721	3.03E-07	L5PT	LDR30m

**Flrt2**	0.60207989	0.00072989	L5PT	LDR30m
**Fosb**	1.63367858	4.91E-15	L5PT	LDR30m
**Fosl2**	1.71891089	7.58E-18	L5PT	LDR30m
**Frmd6**	1.37304638	1.82E-23	L5PT	LDR30m
**Gabbr1**	0.70296611	0.01319393	L5PT	LDR30m
**Galnt9**	0.67038448	0.00058382	L5PT	LDR30m
**Gclc**	1.04181426	3.87E-06	L5PT	LDR30m
**Gfod1**	0.86217363	1.26E-10	L5PT	LDR30m
**Gm3294**	0.76554412	0.02271183	L5PT	LDR30m
**Gm42941**	1.28342991	9.24E-06	L5PT	LDR30m
**Gm45323**	1.27703481	0.00014165	L5PT	LDR30m
**Gm47423**	1.38164383	1.80E-12	L5PT	LDR30m
**Gpr19**	1.20929977	1.48E-05	L5PT	LDR30m
**Grhl1**	1.29163028	7.57E-10	L5PT	LDR30m
**Hnrnpll**	0.86558169	0.02755553	L5PT	LDR30m
**Homer1**	1.60649957	8.06E-15	L5PT	LDR30m
**Hspa4**	1.19868215	1.13E-18	L5PT	LDR30m
**Insyn2a**	1.07079751	0.00109138	L5PT	LDR30m
**Iqgap1**	0.69202616	0.00035381	L5PT	LDR30m
**Ivns1abp**	1.07153244	1.63E-09	L5PT	LDR30m
**Jdp2**	1.01961609	0.0010747	L5PT	LDR30m
**Kdm6b**	1.1754213	9.12E-05	L5PT	LDR30m
**Kdm7a**	1.13307784	1.86E-14	L5PT	LDR30m
**Kif5c**	0.60452978	0.02688102	L5PT	LDR30m
**Kpna1**	0.81090986	0.01416954	L5PT	LDR30m
**Kras**	0.67853036	0.00959893	L5PT	LDR30m
**Lemd3**	1.06254709	1.97E-07	L5PT	LDR30m
**Lncpint**	0.74701653	2.66E-05	L5PT	LDR30m
**Lonrf1**	0.94216024	2.76E-07	L5PT	LDR30m
**Lrrc28**	0.77312367	0.03027822	L5PT	LDR30m
**Lrrk2**	0.75047806	0.01487175	L5PT	LDR30m
**Mapk4**	1.09491872	6.66E-09	L5PT	LDR30m
**Mbp**	0.87917897	4.01E-07	L5PT	LDR30m
**Med14**	1.11810288	4.81E-11	L5PT	LDR30m
**Mef2d**	0.70867429	0.01201179	L5PT	LDR30m
**Mest**	1.18946801	8.25E-09	L5PT	LDR30m
**Mgrn1**	0.89490153	3.18E-07	L5PT	LDR30m
**Mia3**	0.93897172	6.94E-05	L5PT	LDR30m
**Mras**	1.07754963	0.00015172	L5PT	LDR30m

Development • Supplementary information

**Myh9**	1.10983785	0.00019731	L5PT	LDR30m
**Nap1l1**	0.80244971	0.00021287	L5PT	LDR30m
**Ndel1**	0.87674726	0.00039727	L5PT	LDR30m
**Nmnat2**	0.83593612	3.54E-07	L5PT	LDR30m
**Npas4**	1.18154985	0.02846243	L5PT	LDR30m
**Nr4a1**	0.77416979	2.93E-05	L5PT	LDR30m
**Nr4a2**	2.29636409	0.00062162	L5PT	LDR30m
**Nrd1**	0.84668403	2.38E-05	L5PT	LDR30m
**Ntrk2**	0.64951089	5.30E-06	L5PT	LDR30m
**Osbpl8**	0.66828644	4.86E-08	L5PT	LDR30m
**Pdlim1**	1.18656213	2.40E-08	L5PT	LDR30m
**Per1**	1.417619	2.45E-08	L5PT	LDR30m
**Per2**	1.15241264	2.12E-05	L5PT	LDR30m
**Pip5k1a**	0.75264177	0.00011634	L5PT	LDR30m
**Pitpna**	0.71836658	4.42E-07	L5PT	LDR30m
**Plcxd2**	1.44361816	3.92E-10	L5PT	LDR30m
**Pmepa1**	1.00310721	0.00131258	L5PT	LDR30m
**Pou6f1**	0.78534612	0.00298714	L5PT	LDR30m
**Ppard**	0.9113908	0.01809926	L5PT	LDR30m
**Prkar2a**	0.91088625	0.00200945	L5PT	LDR30m
**Psen1**	0.84120667	0.00217812	L5PT	LDR30m
**Pvr**	1.25430734	0.00495325	L5PT	LDR30m
**Rab10**	0.72284703	0.00235237	L5PT	LDR30m
**Rab6b**	0.8591368	0.00020437	L5PT	LDR30m
**Rabgef1**	0.99576832	0.00056991	L5PT	LDR30m
**Rasgef1b**	0.76376594	0.01697888	L5PT	LDR30m
**Rcan2**	0.80146339	0.02207769	L5PT	LDR30m
**Rcc2**	1.23021355	1.99E-06	L5PT	LDR30m
**Rgs7bp**	0.75343619	2.30E-08	L5PT	LDR30m
**Rhbdl3**	0.66734214	0.03585084	L5PT	LDR30m
**Rheb**	1.33677116	6.15E-11	L5PT	LDR30m
**Rims3**	1.28641966	0.02615002	L5PT	LDR30m
**Rims4**	1.0138713	4.55E-09	L5PT	LDR30m
**Rnf217**	1.42781337	1.80E-25	L5PT	LDR30m
**Rps6ka2**	0.98133155	0.0208057	L5PT	LDR30m
**Scg3**	1.02337464	1.40E-06	L5PT	LDR30m
**Setd7**	0.79445532	0.01750162	L5PT	LDR30m
**Sidt1**	0.87830536	1.68E-08	L5PT	LDR30m
**Sik2**	1.34202575	4.93E-23	L5PT	LDR30m

**Sik3**	0.64419843	0.01710221	L5PT	LDR30m
**Ski**	1.05520134	4.12E-05	L5PT	LDR30m
**Slc24a4**	0.8692071	0.00311654	L5PT	LDR30m
**Slc25a25**	1.27835626	1.63E-06	L5PT	LDR30m
**Slc7a1**	1.05952274	0.00051894	L5PT	LDR30m
**Slc7a8**	0.94802976	1.41E-07	L5PT	LDR30m
**Smad1**	0.96823458	3.26E-05	L5PT	LDR30m
**Smg7**	0.71491758	0.00296952	L5PT	LDR30m
**Snx4**	0.86505432	0.03751395	L5PT	LDR30m
**Specc1**	0.59086288	0.00534016	L5PT	LDR30m
**Spock2**	0.78637583	2.00E-05	L5PT	LDR30m
**Stk40**	1.26129777	3.70E-13	L5PT	LDR30m
**Tmem178**	0.67010835	0.00733297	L5PT	LDR30m
**Tnfrsf21**	0.74236415	0.03516852	L5PT	LDR30m
**Trim9**	0.73965077	2.87E-11	L5PT	LDR30m
**Tulp4**	0.87785122	6.58E-07	L5PT	LDR30m
**Txnrd1**	1.04276259	0.00522484	L5PT	LDR30m
**Ube2ql1**	1.39403714	4.90E-24	L5PT	LDR30m
**Usp36**	1.02117759	9.79E-08	L5PT	LDR30m
**Vmp1**	0.68537112	0.00069767	L5PT	LDR30m
**Zbtb11**	0.89953264	4.93E-08	L5PT	LDR30m
**Zc3h12c**	0.95879076	0.00794077	L5PT	LDR30m
**Zdbf2**	1.50270719	8.92E-11	L5PT	LDR30m
**Zfp516**	1.143368	4.42E-06	L5PT	LDR30m
**Zswim6**	0.94123672	3.94E-08	L5PT	LDR30m
**2510009E07Rik**	0.79128681	0.00325039	L5PT	LDR4h
**Ankrd33b**	0.83778481	1.68E-05	L5PT	LDR4h
**Baz1a**	1.74037698	3.63E-24	L5PT	LDR4h
**Ccdc6**	0.78508314	0.00725956	L5PT	LDR4h
**Chrm2**	1.01654743	0.04967664	L5PT	LDR4h
**Cop1**	0.62526701	0.03035258	L5PT	LDR4h
**Dgkz**	0.68293671	0.00012777	L5PT	LDR4h
**Dnajc1**	0.70143642	5.54E-09	L5PT	LDR4h
**Dot1l**	0.70933739	0.00013052	L5PT	LDR4h
**Ece1**	0.82866648	0.00377062	L5PT	LDR4h
**Elmo1**	0.96285547	0.000662	L5PT	LDR4h
**Epb41l1**	0.65719297	4.07E-08	L5PT	LDR4h
**Eprs**	0.9167053	1.42E-05	L5PT	LDR4h
**Fbrsl1**	0.61421358	0.03279753	L5PT	LDR4h

Development • Supplementary information

**Fndc3a**	0.91230201	1.58E-07	L5PT	LDR4h
**Frmd6**	0.81150956	3.03E-06	L5PT	LDR4h
**Galnt9**	0.60486312	0.01676911	L5PT	LDR4h
**Gfod1**	0.73944538	2.73E-05	L5PT	LDR4h
**Gm6225**	1.29575871	0.00262255	L5PT	LDR4h
**Gramd4**	0.90570189	0.00627589	L5PT	LDR4h
**Homer1**	1.0092702	1.62E-05	L5PT	LDR4h
**Ipmk**	0.9024979	0.02006279	L5PT	LDR4h
**Iqgap1**	0.60576065	0.03165327	L5PT	LDR4h
**Lncpint**	0.58611723	0.00663584	L5PT	LDR4h
**Lrrc28**	0.80507054	0.00260687	L5PT	LDR4h
**Lrrk2**	0.86100741	2.41E-09	L5PT	LDR4h
**Mapk14**	0.62291629	0.02968907	L5PT	LDR4h
**Mapk4**	1.32723405	2.02E-26	L5PT	LDR4h
**Mir670hg**	0.99756613	0.0001885	L5PT	LDR4h
**Nap1l1**	0.67274497	0.01529663	L5PT	LDR4h
**Nedd4l**	0.63144461	9.93E-05	L5PT	LDR4h
**Nmnat2**	0.68488934	9.93E-05	L5PT	LDR4h
**Nptx2**	1.37096756	0.00060596	L5PT	LDR4h
**Ntrk2**	0.73461267	1.54E-08	L5PT	LDR4h
**Osbpl3**	0.68980549	0.00012417	L5PT	LDR4h
**Peak1**	0.68484746	0.00713492	L5PT	LDR4h
**Per2**	0.9983686	0.00037456	L5PT	LDR4h
**Phf21b**	1.09594103	4.25E-05	L5PT	LDR4h
**Plcl1**	0.9427402	0.00148775	L5PT	LDR4h
**Plekhg5**	0.68413558	0.00423397	L5PT	LDR4h
**Ppm1h**	0.79412705	1.79E-05	L5PT	LDR4h
**Ppme1**	0.73772312	0.00061719	L5PT	LDR4h
**Ptpn3**	0.69470232	0.00069374	L5PT	LDR4h
**Rnf217**	0.78941909	2.45E-05	L5PT	LDR4h
**Rock2**	0.63440098	0.00218277	L5PT	LDR4h
**Rph3a**	1.02240088	8.50E-07	L5PT	LDR4h
**Sgsm1**	1.25410014	8.99E-10	L5PT	LDR4h
**Sik2**	1.06449819	2.61E-15	L5PT	LDR4h
**Sik3**	0.68254874	3.06E-05	L5PT	LDR4h
**Slc9a5**	1.0383909	0.0049861	L5PT	LDR4h
**Smad3**	0.70921179	0.0194519	L5PT	LDR4h
**Sorcs3**	1.53225259	2.66E-18	L5PT	LDR4h
**Stk40**	0.89518297	0.00256804	L5PT	LDR4h

Development • Supplementary information

**Tet3**	0.89072627	9.80E-08	L5PT	LDR4h
**Tle3**	1.15623381	0.04469111	L5PT	LDR4h
**Tmem163**	0.70897786	0.00038712	L5PT	LDR4h
**Tmem178**	0.59652001	0.00549324	L5PT	LDR4h
**Trim9**	0.59637804	8.99E-06	L5PT	LDR4h
**Zmiz1**	0.9058236	0.04879109	L5PT	LDR4h
**Zswim6**	0.79314334	4.25E-10	L5PT	LDR4h
**Adamts17**	1.09734148	0.00011785	L6IT	LDR
**Cntnap5a**	1.01861906	4.85E-12	L6IT	LDR
**Col23a1**	1.08455125	1.03E-05	L6IT	LDR
**Crim1**	0.84384765	4.12E-05	L6IT	LDR
**Gm20754**	0.8385337	3.62E-05	L6IT	LDR
**Gm42303**	-0.7645457	1.39E-05	L6IT	LDR
**Inpp4b**	1.01275857	1.52E-06	L6IT	LDR
**Kirrel3**	0.88397597	3.38E-07	L6IT	LDR
**Matn2**	1.02804	0.00668165	L6IT	LDR
**Nr3c2**	0.75025247	0.00266713	L6IT	LDR
**Nrn1**	-0.6296775	0.0211244	L6IT	LDR
**Pdzrn4**	1.03239377	2.58E-05	L6IT	LDR
**Rps6ka5**	0.75200337	0.00132433	L6IT	LDR
**Rxfp1**	0.96630438	2.97E-10	L6IT	LDR
**Slc24a4**	0.8614886	5.09E-06	L6IT	LDR
**Trpc5**	0.64845384	0.03070803	L6IT	LDR
**Adgrd1**	1.10888519	0.00109699	L6IT	LDR2h
**Brinp1**	0.88074371	9.29E-06	L6IT	LDR2h
**C1ql3**	1.35945656	1.36E-05	L6IT	LDR2h
**Cdh9**	0.87696538	2.81E-06	L6IT	LDR2h
**Cntn3**	0.79908684	8.70E-08	L6IT	LDR2h
**Dot1l**	0.99039301	0.00073684	L6IT	LDR2h
**Elmo1**	1.27260322	0.00034383	L6IT	LDR2h
**Gadd45g**	1.44024404	2.63E-06	L6IT	LDR2h
**Gm10309**	1.23066385	0.00018892	L6IT	LDR2h
**Gm14636**	0.82494024	0.00030239	L6IT	LDR2h
**Gnal**	0.96377133	3.98E-05	L6IT	LDR2h
**Gpr158**	0.94898358	0.0489386	L6IT	LDR2h
**Grik1**	1.23553271	0.01038901	L6IT	LDR2h
**Hcn1**	0.9728398	0.00126514	L6IT	LDR2h
**Inhba**	1.26183496	1.42E-07	L6IT	LDR2h
**Klf5**	1.16361566	0.00814304	L6IT	LDR2h

Development • Supplementary information

**Mapk4**	1.10878641	0.00060215	L6IT	LDR2h
**Mir670hg**	1.27991105	5.68E-07	L6IT	LDR2h
**Neat1**	1.34897843	5.36E-06	L6IT	LDR2h
**Nptx2**	1.22197729	0.01004152	L6IT	LDR2h
**Nrep**	1.1336107	0.0015074	L6IT	LDR2h
**Nrsn1**	0.98859946	0.00695037	L6IT	LDR2h
**Pcdh17**	0.90008586	0.01712839	L6IT	LDR2h
**Penk**	1.380148	1.17E-07	L6IT	LDR2h
**Phlpp1**	0.79650226	0.00310575	L6IT	LDR2h
**Ppme1**	0.9066613	0.0300503	L6IT	LDR2h
**Ptgs2**	1.25404484	5.13E-05	L6IT	LDR2h
**Rapgef5**	1.0783321	6.13E-08	L6IT	LDR2h
**Rgs2**	1.02167491	0.01703026	L6IT	LDR2h
**Sertad2**	0.78903671	0.00296253	L6IT	LDR2h
**Slc9a5**	1.18580488	0.00781246	L6IT	LDR2h
**Strip2**	1.06887662	0.01047028	L6IT	LDR2h
**1600020E01Rik**	1.04019492	4.13E-10	L6IT	LDR30m
**1700016P03Rik**	2.27006649	0.00514889	L6IT	LDR30m
**Abhd2**	0.86242383	0.00076079	L6IT	LDR30m
**Acsl4**	1.10383353	6.37E-09	L6IT	LDR30m
**Acss1**	1.32823914	0.00046408	L6IT	LDR30m
**Adamts17**	1.00839557	0.00562079	L6IT	LDR30m
**Adora1**	1.29302598	1.83E-13	L6IT	LDR30m
**Agap2**	0.77476003	0.00704921	L6IT	LDR30m
**Agap3**	0.93591732	4.66E-08	L6IT	LDR30m
**Ago2**	0.93403879	3.40E-07	L6IT	LDR30m
**Ago3**	0.83672136	1.89E-07	L6IT	LDR30m
**Ajap1**	0.76128451	0.00715209	L6IT	LDR30m
**Ak4**	1.17422842	5.78E-10	L6IT	LDR30m
**Ank**	0.93422406	2.09E-12	L6IT	LDR30m
**Ankrd33b**	1.28836433	9.54E-23	L6IT	LDR30m
**Anks1**	1.38621929	1.93E-19	L6IT	LDR30m
**Ap2b1**	0.82284575	4.72E-07	L6IT	LDR30m
**Arf4**	0.85498588	0.00320283	L6IT	LDR30m
**Arhgap1**	0.87138806	0.02548076	L6IT	LDR30m
**Arhgef3**	1.45407361	3.69E-14	L6IT	LDR30m
**Arhgef7**	0.62918554	0.00468467	L6IT	LDR30m
**Arid3b**	0.92527542	0.00397687	L6IT	LDR30m
**Arid5a**	1.18260831	0.00417409	L6IT	LDR30m

**Arid5b**	0.88236989	0.00177632	L6IT	LDR30m
**Arih1**	0.68461843	1.72E-10	L6IT	LDR30m
**Arih2**	0.95745795	1.97E-11	L6IT	LDR30m
**Arl5b**	2.01556461	3.50E-20	L6IT	LDR30m
**Arpc2**	1.0344701	6.75E-10	L6IT	LDR30m
**Atl2**	0.84562692	0.00243886	L6IT	LDR30m
**Atp6v0d1**	0.90532747	9.87E-09	L6IT	LDR30m
**Atxn7**	0.78955584	5.38E-05	L6IT	LDR30m
**Baiap2**	1.50209572	1.13E-32	L6IT	LDR30m
**Bicdl1**	0.75298072	0.00182098	L6IT	LDR30m
**Btaf1**	1.15550704	8.21E-21	L6IT	LDR30m
**Btbd8**	0.69866587	0.00285258	L6IT	LDR30m
**Btbd9**	0.5908024	0.02554714	L6IT	LDR30m
**Cabp1**	0.95529025	5.76E-13	L6IT	LDR30m
**Cacng3**	0.63522421	1.51E-07	L6IT	LDR30m
**Cbfa2t3**	0.91031819	0.00619946	L6IT	LDR30m
**Cbfb**	0.97235227	1.75E-05	L6IT	LDR30m
**Ccm2**	1.215097	3.82E-08	L6IT	LDR30m
**Ccnl1**	0.66875394	0.00055278	L6IT	LDR30m
**Cdc27**	0.80499008	7.59E-08	L6IT	LDR30m
**Cdc42ep3**	0.9982683	0.00048097	L6IT	LDR30m
**Chgb**	0.72669351	0.04547513	L6IT	LDR30m
**Ciapin1**	0.92467368	0.04521975	L6IT	LDR30m
**Ciart**	1.42336234	1.70E-13	L6IT	LDR30m
**Clip2**	0.91938669	5.56E-05	L6IT	LDR30m
**Clstn2**	0.6336378	0.01343211	L6IT	LDR30m
**Clstn3**	1.11133323	7.64E-09	L6IT	LDR30m
**Cltc**	0.87669769	1.91E-08	L6IT	LDR30m
**Cmip**	0.78780362	1.08E-07	L6IT	LDR30m
**Cnnm1**	0.77086407	5.08E-09	L6IT	LDR30m
**Col23a1**	0.93877372	0.01201744	L6IT	LDR30m
**Coq10b**	1.1161116	6.77E-07	L6IT	LDR30m
**Cpeb3**	1.26206236	6.69E-19	L6IT	LDR30m
**Cpeb4**	0.98130009	1.39E-08	L6IT	LDR30m
**Crem**	1.05684897	9.23E-06	L6IT	LDR30m
**Crim1**	0.76600722	0.02458149	L6IT	LDR30m
**Cry2**	0.97805628	1.33E-07	L6IT	LDR30m
**Csnk1a1**	0.78187635	9.37E-10	L6IT	LDR30m
**Csnk1d**	0.80159752	0.00606682	L6IT	LDR30m

Development • Supplementary information

**Csrp1**	1.22112567	0.00108957	L6IT	LDR30m
**Ctnnd1**	0.93585837	9.60E-14	L6IT	LDR30m
**Ctps**	1.14754502	3.40E-07	L6IT	LDR30m
**Cwc25**	1.33398334	4.83E-13	L6IT	LDR30m
**D5Ertd579e**	0.59794274	0.00118551	L6IT	LDR30m
**Ddx3y**	0.96085863	0.00027934	L6IT	LDR30m
**Dennd5b**	0.68335664	2.01E-05	L6IT	LDR30m
**Dexi**	0.82530813	0.00804059	L6IT	LDR30m
**Dlg4**	0.68521908	2.64E-05	L6IT	LDR30m
**Dlgap3**	0.65354262	0.00880283	L6IT	LDR30m
**Dlgap4**	0.88043866	3.06E-09	L6IT	LDR30m
**Dnajc1**	0.72207854	0.00014064	L6IT	LDR30m
**Dpysl5**	1.29886519	1.66E-08	L6IT	LDR30m
**Dusp14**	1.02091277	2.99E-05	L6IT	LDR30m
**Dyrk1a**	0.65123204	0.00619564	L6IT	LDR30m
**E330009J07Rik**	0.94115819	7.98E-05	L6IT	LDR30m
**Ece1**	1.05659255	3.04E-06	L6IT	LDR30m
**Efhd2**	1.25233985	1.31E-07	L6IT	LDR30m
**Eif4a1**	0.90602678	0.00686421	L6IT	LDR30m
**Elovl5**	1.32011068	1.63E-07	L6IT	LDR30m
**Epb41l1**	0.83526985	0.00011347	L6IT	LDR30m
**Epha4**	0.6591583	0.00077702	L6IT	LDR30m
**Eprs**	1.06637812	2.92E-11	L6IT	LDR30m
**Erf**	1.06295134	0.0009237	L6IT	LDR30m
**Etv5**	0.93373517	0.00204331	L6IT	LDR30m
**Fbl**	1.24307755	1.97E-10	L6IT	LDR30m
**Fbrsl1**	0.78564222	0.00019593	L6IT	LDR30m
**Fbxo33**	1.14117667	9.79E-12	L6IT	LDR30m
**Fbxo42**	0.75747017	0.03118644	L6IT	LDR30m
**Foxo3**	0.8938631	0.03366561	L6IT	LDR30m
**Frmd6**	1.43783499	6.85E-21	L6IT	LDR30m
**Gabbr1**	1.03168603	2.21E-10	L6IT	LDR30m
**Gadd45b**	1.25672052	0.00016332	L6IT	LDR30m
**Gadd45g**	1.33989342	2.78E-05	L6IT	LDR30m
**Gak**	0.83146957	3.46E-06	L6IT	LDR30m
**Gclc**	0.86903429	3.36E-05	L6IT	LDR30m
**Gdap2**	0.87213481	0.0407737	L6IT	LDR30m
**Gfod1**	0.98916987	8.50E-11	L6IT	LDR30m
**Gm1043**	0.67939819	0.0125045	L6IT	LDR30m

Development • Supplementary information

**Gnai3**	0.94591721	6.21E-07	L6IT	LDR30m
**Gnb5**	0.85778725	1.89E-06	L6IT	LDR30m
**Gng2**	0.69951456	0.00027983	L6IT	LDR30m
**Gpr19**	1.03803573	7.67E-05	L6IT	LDR30m
**Grhl1**	1.17807935	8.52E-10	L6IT	LDR30m
**Hdac5**	1.11320487	1.37E-05	L6IT	LDR30m
**Hivep3**	0.62338028	0.00228509	L6IT	LDR30m
**Hmgcr**	0.83441753	0.00106933	L6IT	LDR30m
**Hnrnpll**	1.11573833	2.31E-08	L6IT	LDR30m
**Homer1**	2.10846541	9.71E-10	L6IT	LDR30m
**Hrh1**	0.99873926	8.08E-06	L6IT	LDR30m
**Hsd17b12**	0.98442391	1.15E-05	L6IT	LDR30m
**Hspa4**	1.23795906	8.92E-11	L6IT	LDR30m
**Hsph1**	1.21751361	5.87E-08	L6IT	LDR30m
**Ifrd1**	0.97330278	0.00028441	L6IT	LDR30m
**Ina**	1.06018407	4.43E-08	L6IT	LDR30m
**Inpp4b**	1.19316432	3.88E-11	L6IT	LDR30m
**Iqgap1**	0.69281299	0.03882073	L6IT	LDR30m
**Ivns1abp**	0.99775608	1.41E-14	L6IT	LDR30m
**Jdp2**	1.65746955	1.08E-15	L6IT	LDR30m
**Kcnmb4**	1.06318688	1.70E-20	L6IT	LDR30m
**Kdm6b**	1.49048503	2.38E-07	L6IT	LDR30m
**Kdm7a**	1.34588165	4.85E-22	L6IT	LDR30m
**Kirrel3**	0.85493638	0.00022282	L6IT	LDR30m
**Klf9**	0.84361357	0.00821893	L6IT	LDR30m
**Kmt2a**	0.73819804	4.29E-07	L6IT	LDR30m
**Kpna1**	0.86046418	2.26E-07	L6IT	LDR30m
**Kras**	0.69145054	0.02015552	L6IT	LDR30m
**Lemd3**	0.98980291	1.14E-08	L6IT	LDR30m
**Lncpint**	1.02424255	4.99E-16	L6IT	LDR30m
**Lonp2**	1.00656627	7.81E-15	L6IT	LDR30m
**Lonrf1**	1.22475943	8.15E-10	L6IT	LDR30m
**Lrrc8b**	0.61364152	0.02347169	L6IT	LDR30m
**Lrrk2**	0.76263175	5.67E-05	L6IT	LDR30m
**Mamld1**	0.86825298	3.70E-06	L6IT	LDR30m
**Map3k13**	0.89528429	0.00248779	L6IT	LDR30m
**Map3k14**	1.2635223	1.44E-08	L6IT	LDR30m
**Mapk4**	0.79764735	0.00030371	L6IT	LDR30m
**Mark3**	0.61164312	3.37E-07	L6IT	LDR30m

Development • Supplementary information

**Mbnl1**	0.84430551	2.33E-07	L6IT	LDR30m
**Mbnl2**	0.73867407	3.79E-13	L6IT	LDR30m
**Mbp**	1.27102493	2.02E-07	L6IT	LDR30m
**Mdk**	1.14487401	0.00223055	L6IT	LDR30m
**Med14**	1.53625039	1.37E-27	L6IT	LDR30m
**Mef2d**	0.72147541	5.24E-05	L6IT	LDR30m
**Mest**	1.4151264	2.77E-16	L6IT	LDR30m
**Mfhas1**	0.62911289	0.00824237	L6IT	LDR30m
**Mgrn1**	1.01413448	1.07E-15	L6IT	LDR30m
**Mia3**	0.80654187	0.00475226	L6IT	LDR30m
**Midn**	1.17564083	0.01838342	L6IT	LDR30m
**Mir670hg**	0.89107332	0.00145309	L6IT	LDR30m
**Mn1**	1.39105291	6.58E-09	L6IT	LDR30m
**Mon2**	0.6715809	0.01276166	L6IT	LDR30m
**Mxi1**	0.93856029	4.27E-05	L6IT	LDR30m
**Myh9**	0.95221159	4.03E-05	L6IT	LDR30m
**Nab1**	0.99469273	0.00349997	L6IT	LDR30m
**Nap1l1**	1.0412333	6.05E-15	L6IT	LDR30m
**Ndel1**	0.90098004	0.00011506	L6IT	LDR30m
**Ndfip2**	1.01649366	0.00912401	L6IT	LDR30m
**Neat1**	1.17554147	0.04117377	L6IT	LDR30m
**Nmnat2**	1.0793112	1.40E-33	L6IT	LDR30m
**Nr3c1**	0.7097192	0.00173189	L6IT	LDR30m
**Nr4a1**	1.2301676	1.76E-07	L6IT	LDR30m
**Nr4a2**	1.90355602	1.01E-18	L6IT	LDR30m
**Nr4a3**	2.50200188	1.66E-05	L6IT	LDR30m
**Nrd1**	1.13795532	4.06E-08	L6IT	LDR30m
**Ntrk2**	0.9875468	3.11E-32	L6IT	LDR30m
**Nudt4**	1.03843422	1.48E-05	L6IT	LDR30m
**Numbl**	1.00666157	5.55E-05	L6IT	LDR30m
**Nup98**	0.59856171	0.00109486	L6IT	LDR30m
**Olfm2**	0.61185518	0.03390704	L6IT	LDR30m
**Osbpl8**	0.81162898	3.74E-06	L6IT	LDR30m
**Otof**	1.24391138	0.0001361	L6IT	LDR30m
**P4ha1**	0.92955105	1.74E-05	L6IT	LDR30m
**Parm1**	1.13548644	0.01291925	L6IT	LDR30m
**Pcsk1**	1.84061723	0.01895648	L6IT	LDR30m
**Pde4a**	0.70704273	0.00815598	L6IT	LDR30m
**Pdlim1**	1.05310619	2.62E-06	L6IT	LDR30m

**Pdzrn4**	1.02637608	0.00031136	L6IT	LDR30m
**Per1**	1.80655329	4.26E-19	L6IT	LDR30m
**Per2**	1.39680726	1.20E-08	L6IT	LDR30m
**Pip5k1a**	0.66346712	0.00033712	L6IT	LDR30m
**Pitpna**	0.82938286	3.00E-11	L6IT	LDR30m
**Plat**	1.01311865	0.02190714	L6IT	LDR30m
**Plekhg5**	0.68338619	0.00506378	L6IT	LDR30m
**Plxnc1**	0.81606048	0.00485999	L6IT	LDR30m
**Pmepa1**	1.07888533	0.0007032	L6IT	LDR30m
**Por**	1.13291054	2.15E-09	L6IT	LDR30m
**Ppard**	1.15330028	0.00105998	L6IT	LDR30m
**Ppp2ca**	0.84093805	0.00376387	L6IT	LDR30m
**Ppp2r2a**	0.68034956	0.00522149	L6IT	LDR30m
**Prkce**	0.64832134	0.00767841	L6IT	LDR30m
**Psd3**	0.78152011	7.98E-08	L6IT	LDR30m
**Ptk2b**	0.67675065	0.00110341	L6IT	LDR30m
**Pvr**	1.19340157	0.00064785	L6IT	LDR30m
**R3hdm2**	0.66262092	1.15E-10	L6IT	LDR30m
**Rab6a**	0.71893213	0.00173366	L6IT	LDR30m
**Rab6b**	0.91646452	5.05E-08	L6IT	LDR30m
**Ranbp9**	0.69168953	3.06E-07	L6IT	LDR30m
**Rap1b**	0.77804338	0.00116184	L6IT	LDR30m
**Rcc2**	1.18965735	9.28E-07	L6IT	LDR30m
**Rgs12**	1.04181937	1.64E-05	L6IT	LDR30m
**Rgs7bp**	0.89436243	1.51E-11	L6IT	LDR30m
**Rheb**	1.27601285	4.90E-10	L6IT	LDR30m
**Rims3**	0.86282301	0.00010532	L6IT	LDR30m
**Rims4**	1.23627202	6.54E-09	L6IT	LDR30m
**Rnf217**	1.31577666	2.11E-12	L6IT	LDR30m
**Rock2**	0.70665836	3.10E-05	L6IT	LDR30m
**Rps6ka2**	0.73318012	0.0041284	L6IT	LDR30m
**Rundc1**	0.99034238	0.00119184	L6IT	LDR30m
**Safb2**	0.7441337	0.00122666	L6IT	LDR30m
**Samd4b**	0.82639877	0.00882521	L6IT	LDR30m
**Samd8**	0.85212532	0.0058353	L6IT	LDR30m
**Sec14l1**	0.88850331	0.00013816	L6IT	LDR30m
**Sec24a**	0.72873365	2.03E-07	L6IT	LDR30m
**7-Sep**	0.80897334	3.04E-06	L6IT	LDR30m
**8-Sep**	0.66354304	0.03544636	L6IT	LDR30m

**9-Sep**	0.88097891	0.03830241	L6IT	LDR30m
**Setd7**	0.72665814	0.0440767	L6IT	LDR30m
**Sez6l**	0.6228831	0.01161024	L6IT	LDR30m
**Shank2**	0.59514378	0.01844168	L6IT	LDR30m
**Siah2**	1.08199504	0.00804948	L6IT	LDR30m
**Sidt1**	0.87539822	4.17E-11	L6IT	LDR30m
**Sik2**	1.80895737	3.26E-37	L6IT	LDR30m
**Sik3**	1.10735674	2.34E-18	L6IT	LDR30m
**Ski**	1.11088273	8.67E-07	L6IT	LDR30m
**Skil**	1.02750383	2.67E-05	L6IT	LDR30m
**Slc16a2**	0.90785528	0.0014067	L6IT	LDR30m
**Slc24a4**	0.99547691	3.77E-12	L6IT	LDR30m
**Slc25a25**	1.29529324	5.71E-10	L6IT	LDR30m
**Slc2a1**	1.41878382	5.40E-12	L6IT	LDR30m
**Slc6a17**	0.93903277	1.79E-15	L6IT	LDR30m
**Slc7a1**	1.01655872	0.00017247	L6IT	LDR30m
**Slc7a8**	0.69705861	0.0086274	L6IT	LDR30m
**Smad1**	1.03006245	5.56E-06	L6IT	LDR30m
**Smad3**	0.80220924	4.65E-12	L6IT	LDR30m
**Smarca2**	0.74212425	0.0002229	L6IT	LDR30m
**Smarca5**	0.91116707	2.41E-05	L6IT	LDR30m
**Smg7**	0.87056414	8.04E-12	L6IT	LDR30m
**Socs7**	0.63438241	0.0041491	L6IT	LDR30m
**Spen**	0.84421622	2.28E-06	L6IT	LDR30m
**Spock2**	0.66291765	0.01227571	L6IT	LDR30m
**Sptbn2**	0.83381844	2.81E-07	L6IT	LDR30m
**Sptbn4**	0.59134945	0.03146764	L6IT	LDR30m
**Srrm4**	0.77740011	7.22E-08	L6IT	LDR30m
**St8sia5**	0.70809136	0.00014705	L6IT	LDR30m
**Stau1**	0.80728123	0.0003209	L6IT	LDR30m
**Stk40**	1.48024129	2.59E-11	L6IT	LDR30m
**Stx1b**	0.87999314	0.00209435	L6IT	LDR30m
**Synj2**	1.02194311	5.58E-07	L6IT	LDR30m
**Taf1**	0.82252988	6.29E-08	L6IT	LDR30m
**Tbc1d1**	1.37857101	6.80E-14	L6IT	LDR30m
**Tbc1d9**	1.16968771	1.90E-14	L6IT	LDR30m
**Tead1**	0.80730303	0.00308655	L6IT	LDR30m
**Tet3**	1.03666431	6.34E-08	L6IT	LDR30m
**Tm9sf3**	0.78067106	0.00108045	L6IT	LDR30m

**Tmem178**	1.21281981	2.05E-20	L6IT	LDR30m
**Tnfrsf21**	0.93216657	5.27E-06	L6IT	LDR30m
**Tpm3**	0.79787332	3.15E-11	L6IT	LDR30m
**Trak1**	0.74732493	0.00153988	L6IT	LDR30m
**Trib1**	1.18872857	0.03566139	L6IT	LDR30m
**Trim9**	0.76946148	9.77E-17	L6IT	LDR30m
**Ttbk1**	0.96033817	3.43E-06	L6IT	LDR30m
**Ttpal**	1.14048067	4.25E-05	L6IT	LDR30m
**Tulp4**	1.16213472	6.17E-25	L6IT	LDR30m
**Txndc11**	1.2197406	7.09E-25	L6IT	LDR30m
**Txnrd1**	1.17560025	1.89E-11	L6IT	LDR30m
**Uba6**	0.82753685	0.03258171	L6IT	LDR30m
**Ube2ql1**	1.77755607	4.90E-19	L6IT	LDR30m
**Unc13a**	0.59349874	0.00679755	L6IT	LDR30m
**Usp36**	1.04629265	6.20E-12	L6IT	LDR30m
**Usp38**	0.924811	0.00893254	L6IT	LDR30m
**Usp9x**	0.70032817	6.04E-07	L6IT	LDR30m
**Vmp1**	0.64984167	0.00023483	L6IT	LDR30m
**Wdr1**	1.03261465	5.16E-05	L6IT	LDR30m
**Xpo1**	0.81873993	1.43E-08	L6IT	LDR30m
**Zbtb11**	0.87826188	5.32E-08	L6IT	LDR30m
**Zbtb16**	0.89646459	0.00457111	L6IT	LDR30m
**Zdbf2**	1.89885322	6.68E-18	L6IT	LDR30m
**Zfp948**	0.90154123	0.01681397	L6IT	LDR30m
**Zhx2**	1.14855348	2.43E-10	L6IT	LDR30m
**Zswim6**	1.20377436	4.34E-18	L6IT	LDR30m
**1600020E01Rik**	0.96950283	1.83E-08	L6IT	LDR4h
**1810030O07Rik**	0.9225177	0.03264724	L6IT	LDR4h
**Adgrd1**	1.06816401	0.00187749	L6IT	LDR4h
**Ankrd33b**	0.78783349	1.61E-06	L6IT	LDR4h
**Arhgef7**	0.73946594	3.15E-06	L6IT	LDR4h
**B530045E10Rik**	1.16285034	0.00014643	L6IT	LDR4h
**Baiap2**	0.63961952	7.40E-06	L6IT	LDR4h
**Baz1a**	1.68458061	0.00303779	L6IT	LDR4h
**Bcor**	1.05022481	9.45E-11	L6IT	LDR4h
**Bdnf**	1.01965311	0.0083057	L6IT	LDR4h
**Brinp1**	0.94739684	4.43E-20	L6IT	LDR4h
**Btbd9**	0.58508158	0.02275071	L6IT	LDR4h
**Cacng2**	0.60564013	8.39E-05	L6IT	LDR4h

Development • Supplementary information

**Caln1**	0.89914805	3.27E-06	L6IT	LDR4h
**Ccdc6**	0.85645889	0.00017942	L6IT	LDR4h
**Ccdc88c**	1.0637456	1.33E-11	L6IT	LDR4h
**Cdh22**	1.0384329	0.0002234	L6IT	LDR4h
**Cdyl**	0.87936779	0.0263063	L6IT	LDR4h
**Chst11**	0.64166196	0.03537782	L6IT	LDR4h
**Chst8**	1.07682268	0.0031035	L6IT	LDR4h
**D5Ertd615e**	1.13941972	0.02861954	L6IT	LDR4h
**Ddah1**	1.01831596	0.00011664	L6IT	LDR4h
**Dgki**	0.76411779	4.88E-07	L6IT	LDR4h
**Disp3**	1.48996101	2.20E-14	L6IT	LDR4h
**Dlgap2**	0.71139897	1.46E-06	L6IT	LDR4h
**Dlgap4**	0.74667304	4.31E-07	L6IT	LDR4h
**Dnajc1**	0.83736053	2.30E-10	L6IT	LDR4h
**Dock4**	0.74093472	0.00119008	L6IT	LDR4h
**Dot1l**	0.96796183	1.72E-13	L6IT	LDR4h
**Dpy19l3**	1.02754378	0.01793697	L6IT	LDR4h
**Dpysl5**	1.07772927	0.00015821	L6IT	LDR4h
**Ece1**	0.76886678	0.00250246	L6IT	LDR4h
**Emb**	0.72913289	0.03655706	L6IT	LDR4h
**Eml5**	0.66662261	0.00019676	L6IT	LDR4h
**Epb41l1**	1.01631676	3.17E-14	L6IT	LDR4h
**Eprs**	0.68327363	0.00166072	L6IT	LDR4h
**Evl**	0.74801728	2.09E-07	L6IT	LDR4h
**Fam129b**	0.92071117	0.01459242	L6IT	LDR4h
**Fbrsl1**	0.60183102	0.03851353	L6IT	LDR4h
**Fmnl1**	0.92526356	6.84E-05	L6IT	LDR4h
**Fndc3a**	0.70223324	7.37E-06	L6IT	LDR4h
**Frmd6**	0.71660517	0.00511457	L6IT	LDR4h
**Fstl4**	0.78100299	0.00327261	L6IT	LDR4h
**Galnt14**	0.82823926	3.15E-10	L6IT	LDR4h
**Galnt9**	0.99808723	0.01288866	L6IT	LDR4h
**Gdpd5**	0.8375435	0.00117603	L6IT	LDR4h
**Gfod1**	0.93746619	1.17E-08	L6IT	LDR4h
**Gm3764**	0.60146813	1.09E-07	L6IT	LDR4h
**Gnb5**	0.70457378	0.00478524	L6IT	LDR4h
**Gpr158**	0.97962357	1.26E-06	L6IT	LDR4h
**Gramd1b**	0.90800198	6.34E-09	L6IT	LDR4h
**Gramd4**	0.71188652	0.01273581	L6IT	LDR4h

Development • Supplementary information

**Grb2**	1.02017485	1.70E-09	L6IT	LDR4h
**Grin2a**	0.65369097	6.78E-05	L6IT	LDR4h
**Hectd2**	1.05031171	5.18E-05	L6IT	LDR4h
**Homer1**	1.01723638	0.00418764	L6IT	LDR4h
**Hrh1**	0.88424456	0.00219073	L6IT	LDR4h
**Hs3st2**	0.85976985	1.29E-09	L6IT	LDR4h
**Hsph1**	1.03640513	5.89E-05	L6IT	LDR4h
**Igsf9b**	0.66906131	8.36E-05	L6IT	LDR4h
**Inpp4b**	0.82804287	0.0096776	L6IT	LDR4h
**Itgav**	0.83081136	1.87E-06	L6IT	LDR4h
**Jarid2**	0.86893714	2.00E-09	L6IT	LDR4h
**Jdp2**	1.05023066	0.00072597	L6IT	LDR4h
**Kdm6b**	1.21761331	0.00270451	L6IT	LDR4h
**Kdm7a**	0.8942967	2.18E-09	L6IT	LDR4h
**Kirrel3**	0.73871899	0.01118122	L6IT	LDR4h
**Klhl3**	0.71542135	7.42E-06	L6IT	LDR4h
**Kmt2a**	0.60305629	4.02E-05	L6IT	LDR4h
**Lncpint**	0.67565543	1.01E-07	L6IT	LDR4h
**Lrrk2**	0.71382991	9.04E-06	L6IT	LDR4h
**Ltbp1**	0.87664757	0.00287692	L6IT	LDR4h
**Mamld1**	0.75496599	2.90E-05	L6IT	LDR4h
**Mapk4**	1.32713627	3.48E-18	L6IT	LDR4h
**Med13**	0.59840533	0.01439717	L6IT	LDR4h
**Megf11**	1.79156449	2.42E-06	L6IT	LDR4h
**Mgrn1**	0.60374139	0.04405479	L6IT	LDR4h
**Mical2**	0.6303395	0.00268446	L6IT	LDR4h
**Mir670hg**	0.94485853	9.35E-06	L6IT	LDR4h
**Mrpl48**	0.82283372	5.43E-06	L6IT	LDR4h
**Mthfd1l**	0.66799761	0.00223696	L6IT	LDR4h
**Myo9b**	0.70821009	5.39E-06	L6IT	LDR4h
**Nap1l1**	0.63493424	2.53E-05	L6IT	LDR4h
**Ncor2**	0.62857849	0.03469277	L6IT	LDR4h
**Neat1**	1.39139086	5.80E-11	L6IT	LDR4h
**Nmnat2**	0.63329782	1.29E-07	L6IT	LDR4h
**Nos1ap**	0.63934268	0.00232339	L6IT	LDR4h
**Nptx2**	1.13513284	0.00181863	L6IT	LDR4h
**Nrp1**	0.97798829	1.51E-08	L6IT	LDR4h
**Nrxn2**	0.95766977	3.98E-12	L6IT	LDR4h
**Ntrk2**	0.90871568	4.32E-38	L6IT	LDR4h

**Ntrk3**	0.75496113	0.00010038	L6IT	LDR4h
**Osbpl3**	0.69994354	4.54E-06	L6IT	LDR4h
**Pawr**	1.36399223	2.55E-09	L6IT	LDR4h
**Pcdh15**	0.69312591	0.00409001	L6IT	LDR4h
**Peli1**	0.72895894	3.17E-05	L6IT	LDR4h
**Per2**	1.05889905	0.00061844	L6IT	LDR4h
**Phf21b**	0.99895558	9.27E-05	L6IT	LDR4h
**Phlpp1**	0.68829644	2.93E-09	L6IT	LDR4h
**Plxna4**	0.74926811	0.00613283	L6IT	LDR4h
**Por**	0.86699262	0.00151079	L6IT	LDR4h
**Ppm1h**	0.74739267	7.06E-12	L6IT	LDR4h
**Ppp1r16b**	0.77884275	0.00730063	L6IT	LDR4h
**Prim2**	1.0406541	4.48E-06	L6IT	LDR4h
**Prkce**	0.66496811	0.00209037	L6IT	LDR4h
**Prkg2**	1.14508068	0.00011075	L6IT	LDR4h
**Prmt8**	0.59148752	0.03208672	L6IT	LDR4h
**Ptprg**	0.70176905	0.00107061	L6IT	LDR4h
**Rai1**	0.62330635	1.51E-05	L6IT	LDR4h
**Rapgef5**	0.84932374	3.61E-11	L6IT	LDR4h
**Rcan2**	0.92756211	3.76E-05	L6IT	LDR4h
**Retreg1**	0.75225095	0.00030076	L6IT	LDR4h
**Rgs20**	0.85664775	0.00072363	L6IT	LDR4h
**Rims3**	0.69658451	0.03821543	L6IT	LDR4h
**Rock2**	0.60644753	0.0002182	L6IT	LDR4h
**Rph3a**	0.87476047	0.00389753	L6IT	LDR4h
**Rps6ka2**	0.75631555	0.00027585	L6IT	LDR4h
**Safb2**	0.63534071	0.00488294	L6IT	LDR4h
**Samd4**	1.05045113	0.00255048	L6IT	LDR4h
**Scube1**	0.85440907	8.78E-05	L6IT	LDR4h
**Sema5b**	0.82487579	1.87E-12	L6IT	LDR4h
**Sgsm1**	1.10340583	1.56E-11	L6IT	LDR4h
**Shank2**	0.60000141	0.01516934	L6IT	LDR4h
**Sik2**	1.504082	2.30E-21	L6IT	LDR4h
**Sik3**	1.1277349	1.53E-18	L6IT	LDR4h
**Slc38a1**	0.6829744	0.0023112	L6IT	LDR4h
**Slc6a17**	0.81940339	1.13E-12	L6IT	LDR4h
**Slc9a5**	1.42490253	2.38E-18	L6IT	LDR4h
**Smad3**	0.79575025	1.15E-08	L6IT	LDR4h
**Smg7**	0.59551885	0.00025249	L6IT	LDR4h

**Sorcs1**	0.70762532	2.25E-06	L6IT	LDR4h
**Sorcs3**	1.05698245	1.07E-10	L6IT	LDR4h
**Spata5**	0.61336929	5.03E-05	L6IT	LDR4h
**Spns2**	0.88512165	8.98E-06	L6IT	LDR4h
**Spred2**	1.00794992	6.27E-10	L6IT	LDR4h
**St8sia5**	0.59840616	0.03767352	L6IT	LDR4h
**Tet3**	1.2167826	3.30E-15	L6IT	LDR4h
**Tmem178**	1.41773918	6.23E-28	L6IT	LDR4h
**Tmtc2**	0.93683558	3.58E-11	L6IT	LDR4h
**Tpm3**	0.59072108	1.03E-05	L6IT	LDR4h
**Traf5**	1.02231275	0.00017463	L6IT	LDR4h
**Trim9**	0.58688884	2.81E-10	L6IT	LDR4h
**Ttll11**	0.58560667	4.71E-06	L6IT	LDR4h
**Tulp4**	0.67906121	3.86E-09	L6IT	LDR4h
**Ubash3b**	1.64881744	0.00036243	L6IT	LDR4h
**Unc13a**	0.62413128	2.50E-06	L6IT	LDR4h
**Xpo1**	0.74954855	6.52E-08	L6IT	LDR4h
**Zbtb16**	0.67544933	0.00019988	L6IT	LDR4h
**Zc3h12c**	0.76344913	0.00052318	L6IT	LDR4h
**Zdhhc14**	0.73254981	0.0017188	L6IT	LDR4h
**Zmiz1**	1.45179783	1.40E-11	L6IT	LDR4h
**Zswim6**	0.93983742	2.17E-10	L6IT	LDR4h
**9630028H03Rik**	-0.7818982	9.05E-05	L6a	LDR
**Adamts17**	1.38626516	5.56E-06	L6a	LDR
**Adamtsl1**	0.76609361	0.00446667	L6a	LDR
**Aff2**	0.70341234	0.00173315	L6a	LDR
**Cacna1g**	1.0852145	0.01772565	L6a	LDR
**Cemip**	1.32135279	5.11E-06	L6a	LDR
**Cntnap4**	1.05244008	4.23E-07	L6a	LDR
**Cntnap5b**	0.89907423	0.00030726	L6a	LDR
**Deptor**	1.06114221	3.24E-05	L6a	LDR
**Dpysl5**	0.89875756	0.01391005	L6a	LDR
**Flnb**	0.68997355	0.04826003	L6a	LDR
**Gm42303**	-1.0260136	2.89E-07	L6a	LDR
**Hlf**	0.85179731	0.0003177	L6a	LDR
**Igdcc4**	0.87813167	0.03603845	L6a	LDR
**Kit**	1.01362903	0.02472288	L6a	LDR
**Nkain3**	0.97664469	2.33E-06	L6a	LDR
**Rps6ka2**	0.71344475	0.0209174	L6a	LDR

Development • Supplementary information

**Sema6a**	0.96986435	3.79E-06	L6a	LDR
**Sh3d19**	0.69701916	0.00075495	L6a	LDR
**Slco3a1**	0.79843957	0.01989006	L6a	LDR
**Smoc2**	1.54094908	6.82E-25	L6a	LDR
**Sox11**	0.82704977	0.02695042	L6a	LDR
**Trpm3**	1.04615179	5.88E-16	L6a	LDR
**Airn**	0.98884957	0.00729912	L6a	LDR2h
**Baz1a**	1.2690685	0.00598355	L6a	LDR2h
**Cdh9**	0.93159612	0.00666951	L6a	LDR2h
**Cenpa**	1.14106137	0.01355452	L6a	LDR2h
**Dot1l**	0.88530737	0.00111822	L6a	LDR2h
**Gadd45b**	1.34833077	6.78E-09	L6a	LDR2h
**Gadd45g**	1.59167601	0.02204934	L6a	LDR2h
**Gm14636**	0.99809654	0.00272309	L6a	LDR2h
**Gm26652**	0.92737489	0.01645624	L6a	LDR2h
**Grhl1**	0.98205168	0.03774549	L6a	LDR2h
**Hcn1**	0.7868063	0.02290378	L6a	LDR2h
**Inhba**	1.36844936	1.99E-11	L6a	LDR2h
**Klf5**	0.98409432	0.01153239	L6a	LDR2h
**Mas1**	1.02558275	0.00243044	L6a	LDR2h
**Mir670hg**	1.38346423	9.78E-07	L6a	LDR2h
**Nefl**	1.24447374	0.00377739	L6a	LDR2h
**Nptx2**	1.52185046	1.36E-10	L6a	LDR2h
**Nrn1**	1.39198615	5.85E-11	L6a	LDR2h
**Nrsn1**	0.99057626	0.01866986	L6a	LDR2h
**Pcdh17**	0.92944222	9.31E-05	L6a	LDR2h
**Pim1**	1.21419825	2.32E-06	L6a	LDR2h
**Ppme1**	0.89794023	0.03279621	L6a	LDR2h
**Rgs2**	1.24797284	4.48E-09	L6a	LDR2h
**Rgs4**	0.91347849	0.00362099	L6a	LDR2h
**Rnd3**	1.3679248	0.00036056	L6a	LDR2h
**Sertad2**	0.75899061	0.00011362	L6a	LDR2h
**Slc9a5**	1.19194121	0.00806128	L6a	LDR2h
**1600020E01Rik**	0.99887589	8.87E-05	L6a	LDR30m
**1700019D03Rik**	-0.6028436	8.74E-10	L6a	LDR30m
**4931406P16Rik**	0.80874235	1.77E-05	L6a	LDR30m
**Abhd2**	0.83523011	0.02813641	L6a	LDR30m
**Acox1**	0.86752825	1.29E-05	L6a	LDR30m
**Acsl4**	0.98141065	1.72E-08	L6a	LDR30m

Development • Supplementary information

**Acss1**	1.27510274	2.02E-10	L6a	LDR30m
**Adam15**	0.90808797	2.68E-05	L6a	LDR30m
**Adamts17**	1.49891913	9.94E-08	L6a	LDR30m
**Adamtsl1**	0.66254804	0.00732958	L6a	LDR30m
**Adar**	0.74588705	0.01010441	L6a	LDR30m
**Adnp**	0.65656735	0.00264659	L6a	LDR30m
**Adora1**	1.41543703	7.47E-26	L6a	LDR30m
**Aff4**	0.71005178	8.06E-15	L6a	LDR30m
**Agap2**	0.85432829	1.49E-07	L6a	LDR30m
**Agap3**	1.2132996	1.88E-14	L6a	LDR30m
**Ago2**	0.98264723	9.57E-10	L6a	LDR30m
**Ak4**	1.18725273	5.17E-10	L6a	LDR30m
**Aldh18a1**	1.21482407	2.84E-12	L6a	LDR30m
**Ank**	0.82994153	0.0010406	L6a	LDR30m
**Ankrd33b**	1.43347088	3.76E-13	L6a	LDR30m
**Anks1**	1.15347554	6.54E-10	L6a	LDR30m
**Ap2b1**	0.8773512	1.72E-11	L6a	LDR30m
**Arf4**	0.98571391	0.00016103	L6a	LDR30m
**Arhgap23**	0.88243851	0.00030482	L6a	LDR30m
**Arhgef3**	1.35283019	1.98E-17	L6a	LDR30m
**Arhgef7**	0.6834942	6.23E-09	L6a	LDR30m
**Arid1a**	0.72661547	0.02317259	L6a	LDR30m
**Arid3b**	0.95586451	0.02634257	L6a	LDR30m
**Arid5b**	0.90646285	8.84E-05	L6a	LDR30m
**Arih1**	1.01216987	7.62E-18	L6a	LDR30m
**Arih2**	1.03756794	6.93E-21	L6a	LDR30m
**Arl4d**	1.17497958	0.01648001	L6a	LDR30m
**Arl5b**	2.27936833	7.72E-06	L6a	LDR30m
**Arpc2**	1.15365941	5.09E-09	L6a	LDR30m
**Atp6v0d1**	1.025571	3.31E-11	L6a	LDR30m
**Atp7a**	0.88988034	0.00033426	L6a	LDR30m
**Atxn7**	1.07913841	1.04E-16	L6a	LDR30m
**BC005537**	0.69569734	0.01588417	L6a	LDR30m
**Baiap2**	1.3856047	9.32E-47	L6a	LDR30m
**Bcor**	0.75014196	0.00366443	L6a	LDR30m
**Bcr**	0.73205637	0.00818416	L6a	LDR30m
**Bicdl1**	0.96858123	5.40E-06	L6a	LDR30m
**Bin1**	0.6004947	0.02502628	L6a	LDR30m
**Braf**	0.70365533	1.08E-08	L6a	LDR30m

**Brd4**	0.60720929	0.03817475	L6a	LDR30m
**Btaf1**	1.2160839	8.04E-19	L6a	LDR30m
**Btbd9**	0.63545158	0.00923777	L6a	LDR30m
**C230057M02Rik**	0.65350622	0.00050344	L6a	LDR30m
**Cabp1**	1.07779803	1.35E-25	L6a	LDR30m
**Cacna1g**	1.27634367	2.04E-12	L6a	LDR30m
**Cacng3**	0.8646066	5.21E-09	L6a	LDR30m
**Camk1g**	1.16326812	0.04407723	L6a	LDR30m
**Cbarp**	1.04401636	0.02071785	L6a	LDR30m
**Cbfa2t3**	0.90127828	1.07E-05	L6a	LDR30m
**Ccdc6**	0.83351581	0.00739012	L6a	LDR30m
**Ccm2**	1.08192479	6.97E-10	L6a	LDR30m
**Ccnl1**	0.94256863	6.27E-11	L6a	LDR30m
**Cdk11b**	0.8399958	5.11E-06	L6a	LDR30m
**Cds1**	0.73029939	5.42E-09	L6a	LDR30m
**Cenpa**	1.22037234	4.35E-08	L6a	LDR30m
**Cherp**	0.80900389	0.00251608	L6a	LDR30m
**Chgb**	1.40596473	4.17E-11	L6a	LDR30m
**Chp1**	0.69652329	0.01345913	L6a	LDR30m
**Ciart**	1.53759281	5.84E-14	L6a	LDR30m
**Clip2**	0.92069961	0.00023264	L6a	LDR30m
**Clstn3**	1.26714889	8.95E-14	L6a	LDR30m
**Cltc**	1.41239445	1.09E-37	L6a	LDR30m
**Cmip**	0.92292894	7.92E-09	L6a	LDR30m
**Coq10b**	1.42511509	3.25E-08	L6a	LDR30m
**Coro1c**	1.15595811	3.43E-08	L6a	LDR30m
**Cpeb3**	1.15004522	3.45E-14	L6a	LDR30m
**Cpeb4**	1.05238581	1.65E-08	L6a	LDR30m
**Cpne6**	1.04360103	0.02009531	L6a	LDR30m
**Crim1**	1.00558611	0.00647394	L6a	LDR30m
**Crtac1**	0.87676283	0.0073389	L6a	LDR30m
**Cry2**	1.14734823	2.40E-12	L6a	LDR30m
**Csde1**	0.60055689	0.01440589	L6a	LDR30m
**Csnk1a1**	0.94157501	6.33E-20	L6a	LDR30m
**Csnk1d**	1.04346485	0.00024155	L6a	LDR30m
**Ctnnb1**	0.79993754	0.00033935	L6a	LDR30m
**Ctnnd1**	1.1139629	2.88E-19	L6a	LDR30m
**Ctps**	1.12943468	3.07E-14	L6a	LDR30m
**Cwc25**	1.6059107	3.92E-43	L6a	LDR30m

Development • Supplementary information

**Cystm1**	0.9291847	9.37E-09	L6a	LDR30m
**D10Wsu102e**	0.60930744	3.47E-05	L6a	LDR30m
**Dbn1**	0.68768253	0.00367863	L6a	LDR30m
**Dcun1d3**	1.0072481	3.69E-08	L6a	LDR30m
**Ddx3y**	1.07763482	6.38E-07	L6a	LDR30m
**Dexi**	0.85585459	3.50E-07	L6a	LDR30m
**Diaph1**	0.73362071	0.00033321	L6a	LDR30m
**Dlgap4**	0.73245435	0.00219534	L6a	LDR30m
**Dnajc1**	0.79170816	0.0023711	L6a	LDR30m
**Dot1l**	0.61754267	7.77E-07	L6a	LDR30m
**Dpy19l3**	1.05610114	1.45E-08	L6a	LDR30m
**Dusp14**	1.35025893	6.18E-07	L6a	LDR30m
**Dyrk1a**	0.74174036	0.02284775	L6a	LDR30m
**E330009J07Rik**	0.9350479	1.29E-10	L6a	LDR30m
**Ece1**	1.3037938	7.21E-14	L6a	LDR30m
**Eepd1**	0.89598695	0.02210871	L6a	LDR30m
**Efhd2**	1.27872656	9.65E-18	L6a	LDR30m
**Efr3b**	0.9132544	7.95E-09	L6a	LDR30m
**Egr3**	2.45511514	0.00899972	L6a	LDR30m
**Eif5**	0.71892915	0.00433247	L6a	LDR30m
**Elk4**	0.85583281	0.01674569	L6a	LDR30m
**Elmsan1**	1.08374823	0.00016745	L6a	LDR30m
**Elovl5**	1.42066368	5.19E-32	L6a	LDR30m
**Emd**	1.3922347	1.28E-07	L6a	LDR30m
**Eprs**	1.36846865	6.86E-43	L6a	LDR30m
**Erf**	1.16926754	4.53E-09	L6a	LDR30m
**Ern1**	0.89732193	7.16E-05	L6a	LDR30m
**Errfi1**	1.37855043	4.31E-07	L6a	LDR30m
**Etf1**	0.99107052	8.50E-13	L6a	LDR30m
**Etv5**	0.88982184	0.00160511	L6a	LDR30m
**Fam117b**	0.62996578	0.001214	L6a	LDR30m
**Fam91a1**	0.96341402	1.11E-06	L6a	LDR30m
**Fbl**	1.37139097	4.67E-23	L6a	LDR30m
**Fbrsl1**	0.85786636	1.36E-13	L6a	LDR30m
**Fbxo33**	1.0212377	0.00014393	L6a	LDR30m
**Fosl2**	2.08889552	0.04078389	L6a	LDR30m
**Foxo3**	1.06134926	3.80E-05	L6a	LDR30m
**Frmd6**	1.40335262	8.92E-10	L6a	LDR30m
**Gabbr1**	0.90776975	2.19E-13	L6a	LDR30m

**Gadd45b**	1.56499588	2.69E-16	L6a	LDR30m
**Gadd45g**	1.24540715	0.00257386	L6a	LDR30m
**Gak**	1.12133953	4.38E-16	L6a	LDR30m
**Galnt9**	0.90356984	8.30E-07	L6a	LDR30m
**Gclc**	1.13081654	3.69E-14	L6a	LDR30m
**Gfod1**	0.82486092	3.29E-05	L6a	LDR30m
**Gm1043**	0.66219148	0.00866521	L6a	LDR30m
**Gm10563**	0.91725239	0.00480652	L6a	LDR30m
**Gm12940**	0.78813711	0.00085877	L6a	LDR30m
**Gm17501**	0.9349198	0.0230656	L6a	LDR30m
**Gm42303**	-0.7475068	3.41E-05	L6a	LDR30m
**Gm6225**	1.32132085	4.27E-09	L6a	LDR30m
**Gmeb2**	0.81446662	1.44E-05	L6a	LDR30m
**Gnai3**	1.00527413	4.62E-12	L6a	LDR30m
**Gnao1**	0.62194522	0.01367338	L6a	LDR30m
**Gnb5**	0.63595081	0.00668105	L6a	LDR30m
**Gng2**	0.77543606	0.022847	L6a	LDR30m
**Gng4**	1.12130926	0.00686303	L6a	LDR30m
**Gng7**	1.17757791	4.69E-06	L6a	LDR30m
**Golph3**	0.86529246	0.00128082	L6a	LDR30m
**Gpr19**	1.17927739	1.14E-12	L6a	LDR30m
**Gpt2**	0.84557928	0.00547085	L6a	LDR30m
**Gramd1a**	0.77713117	0.00086124	L6a	LDR30m
**Grhl1**	1.15028867	1.25E-09	L6a	LDR30m
**Grm4**	1.22840671	2.72E-07	L6a	LDR30m
**Hdac5**	1.10798539	1.17E-06	L6a	LDR30m
**Heca**	0.80743524	0.0048908	L6a	LDR30m
**Hmgcr**	1.04974492	0.00039275	L6a	LDR30m
**Hnrnpll**	1.04900357	0.00172551	L6a	LDR30m
**Hsd17b12**	1.0672471	5.21E-15	L6a	LDR30m
**Hspa4**	1.84107742	3.56E-32	L6a	LDR30m
**Hsph1**	1.07593736	2.88E-07	L6a	LDR30m
**Ifrd1**	0.91884037	0.00016841	L6a	LDR30m
**Ilf3**	0.8720305	2.06E-05	L6a	LDR30m
**Ilrun**	0.78409057	0.03673182	L6a	LDR30m
**Inpp4b**	1.21816597	0.00610778	L6a	LDR30m
**Iqgap1**	0.75973845	1.76E-15	L6a	LDR30m
**Isy1**	0.86952395	0.00844559	L6a	LDR30m
**Ivns1abp**	1.03592361	1.23E-16	L6a	LDR30m

Development • Supplementary information

**Jak1**	0.6099714	0.00521686	L6a	LDR30m
**Jdp2**	1.21663807	8.70E-08	L6a	LDR30m
**Kcnk1**	0.9232502	0.00012352	L6a	LDR30m
**Kcnk2**	-0.6282354	0.03360923	L6a	LDR30m
**Kctd8**	1.32069063	5.17E-15	L6a	LDR30m
**Kdm6b**	1.6376529	6.18E-28	L6a	LDR30m
**Kdm6bos**	1.12963678	0.0022033	L6a	LDR30m
**Kdm7a**	1.17525361	1.17E-19	L6a	LDR30m
**Kif5c**	0.6503905	8.73E-06	L6a	LDR30m
**Klf9**	0.71046978	0.00735255	L6a	LDR30m
**Klhl15**	0.66527825	0.00554921	L6a	LDR30m
**Kmt2a**	0.68991301	2.60E-10	L6a	LDR30m
**Kpna1**	0.94934908	3.93E-11	L6a	LDR30m
**Kras**	0.82498994	1.75E-10	L6a	LDR30m
**Lats2**	1.09696767	0.00127351	L6a	LDR30m
**Lmbr1l**	1.03558024	0.04602313	L6a	LDR30m
**Lmo3**	-0.7471898	7.00E-07	L6a	LDR30m
**Lncpint**	1.11672181	4.94E-18	L6a	LDR30m
**Lonp2**	0.81747193	4.67E-07	L6a	LDR30m
**Lonrf1**	1.01329707	2.45E-06	L6a	LDR30m
**Lrrc8a**	0.98911092	0.01610106	L6a	LDR30m
**Lrrk2**	0.66423371	0.00020242	L6a	LDR30m
**Mamld1**	0.91944547	0.00096509	L6a	LDR30m
**Map3k14**	1.21074453	6.21E-09	L6a	LDR30m
**Mapre1**	0.73993971	0.00252999	L6a	LDR30m
**11-Mar**	0.91338978	4.07E-08	L6a	LDR30m
**Mark3**	0.75088643	3.98E-14	L6a	LDR30m
**Mbnl1**	0.9693006	2.06E-17	L6a	LDR30m
**Mbp**	1.03207847	4.89E-06	L6a	LDR30m
**Med14**	1.51223645	4.88E-35	L6a	LDR30m
**Mef2d**	0.91065605	5.04E-13	L6a	LDR30m
**Mest**	1.50881193	4.99E-20	L6a	LDR30m
**Mgrn1**	1.20670957	2.20E-37	L6a	LDR30m
**Mia3**	1.00430057	9.13E-12	L6a	LDR30m
**Mir670hg**	0.89209036	0.01996391	L6a	LDR30m
**Mir9-3hg**	0.59276755	0.04170194	L6a	LDR30m
**Mknk2**	1.17729098	6.20E-05	L6a	LDR30m
**Mn1**	1.42465904	2.88E-13	L6a	LDR30m
**Mon2**	0.66253713	6.03E-06	L6a	LDR30m

Development • Supplementary information

**Mpc1**	0.79451053	0.01306862	L6a	LDR30m
**Mpp2**	0.94256825	8.62E-05	L6a	LDR30m
**Mrpl48**	0.94393946	1.45E-05	L6a	LDR30m
**Mtf1**	0.80381527	0.00293591	L6a	LDR30m
**Mthfd1l**	1.00616308	8.86E-08	L6a	LDR30m
**Mxi1**	0.95122263	9.75E-09	L6a	LDR30m
**Myh9**	1.37542398	2.19E-18	L6a	LDR30m
**Myo1e**	1.19619944	0.00121821	L6a	LDR30m
**Nab2**	1.12091637	2.38E-06	L6a	LDR30m
**Nap1l1**	0.91093427	1.46E-18	L6a	LDR30m
**Nav1**	0.93144961	0.03403966	L6a	LDR30m
**Ncor2**	0.90481609	9.79E-06	L6a	LDR30m
**Ndel1**	1.01104762	4.29E-08	L6a	LDR30m
**Nedd4l**	0.70262739	6.83E-08	L6a	LDR30m
**Nkain3**	0.85232751	0.01084685	L6a	LDR30m
**Nmnat2**	1.50643001	5.19E-41	L6a	LDR30m
**Nolc1**	1.08122729	1.50E-05	L6a	LDR30m
**Nop53**	1.23903562	4.22E-07	L6a	LDR30m
**Nr4a1**	0.99059891	4.77E-05	L6a	LDR30m
**Nr4a2**	3.06980736	0.00038921	L6a	LDR30m
**Nr4a3**	2.89915061	0.00018759	L6a	LDR30m
**Nrd1**	1.01989378	2.61E-08	L6a	LDR30m
**Nrn1**	1.07086594	0.00015125	L6a	LDR30m
**Ntrk2**	0.87817596	2.59E-21	L6a	LDR30m
**Nudt4**	1.00760701	1.65E-07	L6a	LDR30m
**Numbl**	0.95932009	0.00506459	L6a	LDR30m
**Nup98**	0.77915048	6.07E-11	L6a	LDR30m
**Olfml2b**	0.95863811	0.02813273	L6a	LDR30m
**Orai1**	1.09734483	0.03910054	L6a	LDR30m
**Paf1**	0.9157329	0.0085279	L6a	LDR30m
**Parm1**	1.04457063	0.02843647	L6a	LDR30m
**Pcdh10**	-0.7359982	0.00022394	L6a	LDR30m
**Pcdh19**	1.06920461	0.01836442	L6a	LDR30m
**Pcsk1**	2.13524421	3.79E-06	L6a	LDR30m
**Pde4a**	0.78148832	8.81E-08	L6a	LDR30m
**Pdlim1**	0.94208125	0.00477413	L6a	LDR30m
**Per2**	1.30125739	1.38E-12	L6a	LDR30m
**Pex5**	0.9788923	0.00660166	L6a	LDR30m
**Pgm2**	1.04175459	5.49E-11	L6a	LDR30m

Development • Supplementary information

**Phf20**	0.60853467	3.82E-05	L6a	LDR30m
**Pi4k2a**	0.72001024	0.04490817	L6a	LDR30m
**Pisd**	0.7741728	0.00687534	L6a	LDR30m
**Pitpna**	0.93570531	7.55E-17	L6a	LDR30m
**Pitpnm2**	0.79865994	0.01195868	L6a	LDR30m
**Plat**	1.19727986	1.66E-05	L6a	LDR30m
**Plcd1**	1.31596284	2.99E-12	L6a	LDR30m
**Plcxd2**	1.25059499	0.00014017	L6a	LDR30m
**Plekhg5**	0.61350128	6.44E-05	L6a	LDR30m
**Pmvk**	1.2445308	2.08E-10	L6a	LDR30m
**Por**	1.06115757	1.29E-08	L6a	LDR30m
**Pou6f1**	0.90506815	5.48E-07	L6a	LDR30m
**Ppan**	1.04079924	0.01128022	L6a	LDR30m
**Ppard**	1.06659861	1.62E-06	L6a	LDR30m
**Ppargc1b**	0.71242295	0.00129498	L6a	LDR30m
**Ppm1d**	0.93516724	6.14E-06	L6a	LDR30m
**Ppp1cc**	1.1472717	1.04E-16	L6a	LDR30m
**Ppp2ca**	0.80125724	0.0041852	L6a	LDR30m
**Pprc1**	0.94124895	0.01191505	L6a	LDR30m
**Prkx**	1.05768921	6.67E-06	L6a	LDR30m
**Prox1**	1.36957524	6.32E-15	L6a	LDR30m
**Ptk2b**	0.64838764	4.86E-10	L6a	LDR30m
**Ptpn12**	0.69046257	0.00319777	L6a	LDR30m
**Ptpn2**	0.71317877	4.77E-06	L6a	LDR30m
**Pvr**	1.13475427	1.84E-06	L6a	LDR30m
**Rab11fip4os1**	1.05372652	0.03137603	L6a	LDR30m
**Rab6a**	0.86656792	2.65E-06	L6a	LDR30m
**Rab6b**	0.88455279	1.35E-10	L6a	LDR30m
**Rab7**	0.75180234	0.00035611	L6a	LDR30m
**Rabgef1**	0.86828289	3.90E-06	L6a	LDR30m
**Rai1**	0.72762616	0.01178722	L6a	LDR30m
**Ranbp2**	0.95840279	8.16E-06	L6a	LDR30m
**Rap1b**	0.84579647	1.25E-06	L6a	LDR30m
**Rapgef2**	0.62092947	1.61E-09	L6a	LDR30m
**Rassf5**	1.0490463	0.00050275	L6a	LDR30m
**Rassf8**	0.90007518	0.02390199	L6a	LDR30m
**Rbms2**	1.16716218	6.03E-07	L6a	LDR30m
**Rcan2**	0.96936088	2.21E-06	L6a	LDR30m
**Rcc2**	1.42186209	6.47E-30	L6a	LDR30m

Development • Supplementary information

**Relb**	1.12226789	0.00623065	L6a	LDR30m
**Rell1**	0.85361072	6.12E-07	L6a	LDR30m
**Rgl1**	0.64808667	0.00057641	L6a	LDR30m
**Rgs2**	1.06539687	0.01557618	L6a	LDR30m
**Rgs7bp**	0.78480244	1.12E-14	L6a	LDR30m
**Rheb**	1.5713536	4.68E-09	L6a	LDR30m
**Rhot2**	0.89825263	5.71E-07	L6a	LDR30m
**Rims3**	1.27837329	0.00021116	L6a	LDR30m
**Rims4**	1.2045814	3.32E-08	L6a	LDR30m
**Rnf217**	1.3896861	4.71E-28	L6a	LDR30m
**Rock2**	0.67200449	0.01519209	L6a	LDR30m
**Rph3a**	0.61485438	0.01765746	L6a	LDR30m
**Rps6ka2**	0.90709576	2.30E-09	L6a	LDR30m
**Rundc1**	0.86258146	0.0224937	L6a	LDR30m
**Samd4b**	0.80548932	8.87E-06	L6a	LDR30m
**Sap130**	0.64470363	0.00053859	L6a	LDR30m
**Schip1**	0.77635404	0.01150846	L6a	LDR30m
**Sec14l1**	0.992485	1.82E-10	L6a	LDR30m
**Sec24a**	0.82722981	8.41E-06	L6a	LDR30m
**8-Sep**	0.6536246	0.0051087	L6a	LDR30m
**9-Sep**	0.98100538	1.24E-05	L6a	LDR30m
**Setd7**	0.7928848	6.25E-06	L6a	LDR30m
**Siah2**	1.28903697	2.70E-15	L6a	LDR30m
**Sidt1**	0.90028347	2.01E-07	L6a	LDR30m
**Sik2**	1.67893824	3.40E-26	L6a	LDR30m
**Sik3**	1.1556309	5.48E-25	L6a	LDR30m
**Ski**	1.30525447	7.97E-11	L6a	LDR30m
**Skil**	1.04025685	0.00325124	L6a	LDR30m
**Slc12a2**	0.90176117	1.24E-06	L6a	LDR30m
**Slc16a10**	0.92449132	6.26E-07	L6a	LDR30m
**Slc16a2**	1.27894536	0.00300398	L6a	LDR30m
**Slc24a4**	1.20723837	1.69E-05	L6a	LDR30m
**Slc25a25**	1.09241137	5.82E-05	L6a	LDR30m
**Slc2a1**	1.44514714	7.20E-16	L6a	LDR30m
**Slc2a3**	0.76651822	1.08E-06	L6a	LDR30m
**Slc6a17**	0.88909202	3.89E-15	L6a	LDR30m
**Slc6a8**	0.8197478	0.04960955	L6a	LDR30m
**Slc7a1**	1.06154031	1.25E-07	L6a	LDR30m
**Slc7a5**	1.28744799	4.56E-08	L6a	LDR30m

**Slc7a8**	0.87756412	2.51E-22	L6a	LDR30m
**Slco3a1**	0.70418383	0.01403926	L6a	LDR30m
**Smad1**	1.00736081	2.50E-09	L6a	LDR30m
**Smad3**	0.97350283	2.92E-10	L6a	LDR30m
**Smarca2**	0.79846435	4.01E-05	L6a	LDR30m
**Smarca5**	1.06356024	2.72E-12	L6a	LDR30m
**Smg7**	0.9940547	6.08E-18	L6a	LDR30m
**Smoc2**	1.28070791	1.06E-10	L6a	LDR30m
**Socs7**	0.82404247	1.42E-07	L6a	LDR30m
**Spag9**	0.61617507	2.05E-05	L6a	LDR30m
**Spen**	1.07734262	1.86E-21	L6a	LDR30m
**Spock2**	0.59425171	0.0226191	L6a	LDR30m
**Sptbn2**	0.83335757	5.01E-12	L6a	LDR30m
**Spty2d1**	1.36634558	3.54E-08	L6a	LDR30m
**Srrm4**	0.78209485	3.66E-06	L6a	LDR30m
**St8sia5**	0.81347404	4.83E-19	L6a	LDR30m
**Stat3**	1.57326713	7.60E-34	L6a	LDR30m
**Stau1**	0.69579835	0.01070338	L6a	LDR30m
**Stk38l**	0.77937563	0.02904869	L6a	LDR30m
**Stk40**	1.51732825	6.31E-09	L6a	LDR30m
**Stx1b**	0.95512698	9.16E-07	L6a	LDR30m
**Supt6**	0.62808935	0.02005777	L6a	LDR30m
**Susd6**	0.610018	0.00628358	L6a	LDR30m
**Sv2c**	1.27238668	5.65E-08	L6a	LDR30m
**Synj2**	0.70479855	0.00019881	L6a	LDR30m
**Syt4**	1.14791752	3.63E-05	L6a	LDR30m
**Taf1**	0.71444799	7.64E-09	L6a	LDR30m
**Taf13**	1.04299631	0.01333615	L6a	LDR30m
**Tarsl2**	0.73902727	0.04620885	L6a	LDR30m
**Tbc1d1**	1.19458908	1.86E-15	L6a	LDR30m
**Tbc1d16**	1.06626365	0.00037813	L6a	LDR30m
**Tbc1d9**	0.97198201	7.23E-11	L6a	LDR30m
**Tet2**	0.62012717	0.00224395	L6a	LDR30m
**Tet3**	0.79364462	0.00025717	L6a	LDR30m
**Tiparp**	2.47325054	0.00323298	L6a	LDR30m
**Tm9sf3**	0.73886448	1.31E-05	L6a	LDR30m
**Tmem178**	0.64771739	0.00233072	L6a	LDR30m
**Tmem196**	-0.6096609	3.93E-06	L6a	LDR30m
**Tnfaip6**	1.33232034	1.73E-11	L6a	LDR30m

Development • Supplementary information

**Top1**	0.6203492	0.00684887	L6a	LDR30m
**Tor1aip1**	0.8230786	0.00885743	L6a	LDR30m
**Tpm3**	0.74453553	2.67E-12	L6a	LDR30m
**Trak1**	1.20668523	1.32E-14	L6a	LDR30m
**Trim71**	1.07960802	0.0407974	L6a	LDR30m
**Trim9**	0.68996029	2.20E-09	L6a	LDR30m
**Trpm3**	1.20521356	3.11E-20	L6a	LDR30m
**Tsc22d2**	0.77828232	0.00788516	L6a	LDR30m
**Ttbk1**	1.1078187	1.03E-09	L6a	LDR30m
**Ttc28**	1.13034305	0.00174017	L6a	LDR30m
**Ttll3**	0.96533895	0.0193753	L6a	LDR30m
**Ttpal**	0.93677943	0.00108448	L6a	LDR30m
**Tulp4**	1.05015979	6.41E-32	L6a	LDR30m
**Txndc11**	1.04888669	5.00E-29	L6a	LDR30m
**Txnrd1**	1.10598681	1.66E-09	L6a	LDR30m
**Ube2h**	0.75120387	8.71E-07	L6a	LDR30m
**Ube2ql1**	1.47564328	3.24E-13	L6a	LDR30m
**Ubl3**	0.87908364	1.72E-07	L6a	LDR30m
**Ubtf**	0.87649474	5.00E-07	L6a	LDR30m
**Ugdh**	0.98193468	2.13E-05	L6a	LDR30m
**Unc13a**	0.59212296	4.84E-05	L6a	LDR30m
**Unc45a**	1.07093009	4.28E-07	L6a	LDR30m
**Usp36**	1.42545584	1.43E-29	L6a	LDR30m
**Usp38**	0.80599806	0.00047873	L6a	LDR30m
**Usp4**	0.64290765	0.00036425	L6a	LDR30m
**Usp9x**	0.80408771	6.64E-08	L6a	LDR30m
**Vcl**	0.76652075	0.03050387	L6a	LDR30m
**Vps37b**	1.0528904	3.28E-10	L6a	LDR30m
**Wdfy2**	1.05588472	5.84E-16	L6a	LDR30m
**Wdr1**	1.14235446	1.30E-12	L6a	LDR30m
**Wdr26**	0.63065614	0.00415165	L6a	LDR30m
**Wdr45b**	0.69908481	0.00137396	L6a	LDR30m
**Ywhaz**	0.61853412	0.00200745	L6a	LDR30m
**Zbtb11**	0.97401511	7.73E-16	L6a	LDR30m
**Zbtb4**	0.91877738	1.40E-06	L6a	LDR30m
**Zdbf2**	1.96634696	2.95E-05	L6a	LDR30m
**Zfp598**	0.89902032	0.02721265	L6a	LDR30m
**Zhx2**	1.26107429	1.32E-15	L6a	LDR30m
**Zkscan3**	0.62938395	0.01951932	L6a	LDR30m

Development • Supplementary information

**Zswim6**	1.28690531	5.33E-24	L6a	LDR30m
**Zwint**	0.62030747	0.04032324	L6a	LDR30m
**1600020E01Rik**	0.87345383	0.00642033	L6a	LDR4h
**1810030O07Rik**	1.01310724	0.00121396	L6a	LDR4h
**6430548M08Rik**	0.93204538	6.37E-06	L6a	LDR4h
**Actn4**	0.79560124	0.00072561	L6a	LDR4h
**Airn**	0.87517126	9.91E-05	L6a	LDR4h
**Arhgef7**	0.83174753	3.16E-10	L6a	LDR4h
**Baiap2**	0.61617888	2.25E-07	L6a	LDR4h
**Baz1a**	1.72788326	5.43E-21	L6a	LDR4h
**Bcor**	0.90171395	1.50E-16	L6a	LDR4h
**Brinp1**	0.84019113	9.19E-10	L6a	LDR4h
**Btbd9**	0.73156208	0.00180542	L6a	LDR4h
**Cabp1**	0.58991184	4.78E-05	L6a	LDR4h
**Ccdc6**	0.95779938	1.82E-07	L6a	LDR4h
**Cdh22**	1.12957802	0.00592706	L6a	LDR4h
**Cdk13**	0.63602888	3.04E-09	L6a	LDR4h
**Cenpa**	1.11141152	0.01491073	L6a	LDR4h
**Clstn3**	0.78046213	0.04887403	L6a	LDR4h
**Cmip**	0.78651689	0.00010044	L6a	LDR4h
**Col4a1**	0.70445065	0.03830995	L6a	LDR4h
**Col4a2**	0.66246164	0.00153599	L6a	LDR4h
**Crim1**	0.92425935	0.0158976	L6a	LDR4h
**Crtac1**	0.88344527	0.00026561	L6a	LDR4h
**Csnk1a1**	0.61569543	8.98E-10	L6a	LDR4h
**Dab2ip**	0.59459542	0.01602734	L6a	LDR4h
**Dagla**	0.5976492	0.02616223	L6a	LDR4h
**Ddah1**	0.8366619	1.88E-09	L6a	LDR4h
**Dgki**	0.5866057	0.00433048	L6a	LDR4h
**Dgkz**	0.5859289	7.30E-06	L6a	LDR4h
**Disp3**	1.22383528	0.00060896	L6a	LDR4h
**Dlgap4**	0.72245127	5.46E-05	L6a	LDR4h
**Dmtn**	0.6471482	4.72E-09	L6a	LDR4h
**Dnajc1**	0.84675132	6.60E-07	L6a	LDR4h
**Dnmt1**	0.85072792	0.00210553	L6a	LDR4h
**Dot1l**	0.85359825	7.61E-21	L6a	LDR4h
**Dpy19l3**	0.91372018	1.72E-06	L6a	LDR4h
**Ece1**	0.91359458	0.00033137	L6a	LDR4h
**Efr3b**	0.68319329	1.20E-05	L6a	LDR4h

**Elf2**	0.80870374	3.44E-05	L6a	LDR4h
**Eml5**	0.78621538	1.40E-07	L6a	LDR4h
**Enox1**	0.72159995	0.01951257	L6a	LDR4h
**Entpd7**	0.88775088	1.75E-06	L6a	LDR4h
**Epb41l1**	0.6541344	4.40E-14	L6a	LDR4h
**Epha10**	0.88737724	1.87E-08	L6a	LDR4h
**Eprs**	0.86762883	8.61E-08	L6a	LDR4h
**Fam129b**	0.88904813	0.00110457	L6a	LDR4h
**Fam171a1**	0.60119091	0.03223136	L6a	LDR4h
**Fbrsl1**	0.62700463	8.62E-08	L6a	LDR4h
**Fstl4**	1.03834323	1.44E-06	L6a	LDR4h
**Gak**	0.64818535	0.0068411	L6a	LDR4h
**Galnt9**	0.71858702	0.00045578	L6a	LDR4h
**Gdpd5**	0.76220685	2.61E-05	L6a	LDR4h
**Gfod1**	0.89527792	7.31E-09	L6a	LDR4h
**Gfra1**	1.87367593	0.00409872	L6a	LDR4h
**Gm42303**	-0.8451952	0.00431541	L6a	LDR4h
**Gm6225**	1.13361471	0.00074992	L6a	LDR4h
**Gmeb1**	0.60605502	0.03875709	L6a	LDR4h
**Gmeb2**	0.93257667	5.11E-11	L6a	LDR4h
**Gramd4**	0.70283247	0.00303281	L6a	LDR4h
**Grb2**	0.98323341	4.62E-13	L6a	LDR4h
**Grin2a**	0.63514896	2.45E-10	L6a	LDR4h
**Hs3st2**	0.69034126	2.45E-10	L6a	LDR4h
**Hsd17b12**	0.82135615	1.83E-06	L6a	LDR4h
**Inhba**	1.13178906	0.00136636	L6a	LDR4h
**Inpp4b**	0.98567435	0.01344755	L6a	LDR4h
**Inpp5a**	0.66518942	0.04366868	L6a	LDR4h
**Ipmk**	0.85355608	0.00634403	L6a	LDR4h
**Ippk**	0.82699479	0.00037014	L6a	LDR4h
**Iqsec3**	0.72253043	0.00016679	L6a	LDR4h
**Itgav**	0.85548642	6.53E-12	L6a	LDR4h
**Jdp2**	1.20859489	2.50E-14	L6a	LDR4h
**Kdm6b**	1.29821504	6.30E-14	L6a	LDR4h
**Kdm7a**	0.73204557	4.87E-07	L6a	LDR4h
**Kmt2a**	0.63291368	3.11E-05	L6a	LDR4h
**Lingo1**	0.59227133	0.04823799	L6a	LDR4h
**Lncpint**	0.83509519	1.34E-09	L6a	LDR4h
**Lrrk2**	0.69312408	0.00031702	L6a	LDR4h

Development • Supplementary information

**Mamld1**	1.04227924	2.88E-09	L6a	LDR4h
**Mapk4**	1.74391198	1.18E-18	L6a	LDR4h
**Mapk6**	1.11229421	0.00011784	L6a	LDR4h
**Mark4**	0.69088286	0.00046931	L6a	LDR4h
**Med14**	0.69588388	4.00E-05	L6a	LDR4h
**Megf11**	1.40396945	3.22E-07	L6a	LDR4h
**Mical2**	0.6380612	1.02E-05	L6a	LDR4h
**Mir22hg**	0.89269038	0.04977322	L6a	LDR4h
**Mir670hg**	1.23491285	1.11E-12	L6a	LDR4h
**Mrpl48**	0.88711812	5.36E-05	L6a	LDR4h
**Myh9**	1.00479226	0.00087589	L6a	LDR4h
**Myo9b**	0.72160455	8.71E-09	L6a	LDR4h
**Ncor2**	0.83925404	0.00011965	L6a	LDR4h
**Neat1**	1.08164429	0.00404851	L6a	LDR4h
**Nedd4l**	0.61060217	4.97E-06	L6a	LDR4h
**Nfkb1**	0.71069641	0.0006956	L6a	LDR4h
**Nmnat2**	1.07436967	1.21E-16	L6a	LDR4h
**Nptx2**	1.17589172	2.39E-05	L6a	LDR4h
**Nrn1**	0.91612613	0.01127285	L6a	LDR4h
**Nrp1**	0.69048053	6.45E-10	L6a	LDR4h
**Nrxn2**	0.96751236	1.66E-30	L6a	LDR4h
**Nsun2**	0.72689974	3.83E-10	L6a	LDR4h
**Ntrk2**	0.95155539	8.73E-26	L6a	LDR4h
**Osbpl8**	0.64121171	8.99E-13	L6a	LDR4h
**Pcdh15**	0.7404098	0.01350209	L6a	LDR4h
**Pde9a**	0.71168595	0.00358518	L6a	LDR4h
**Pdzd2**	0.85772414	0.00047917	L6a	LDR4h
**Peak1**	0.6633606	0.00034079	L6a	LDR4h
**Per2**	0.9799767	7.05E-07	L6a	LDR4h
**Phf21b**	1.08454736	8.23E-05	L6a	LDR4h
**Pim1**	1.16980829	8.19E-05	L6a	LDR4h
**Pknox1**	0.83297126	0.01477806	L6a	LDR4h
**Plekhg4**	1.10778862	0.04796636	L6a	LDR4h
**Plekhg5**	0.64780888	1.75E-06	L6a	LDR4h
**Pou6f1**	0.69486817	0.01488372	L6a	LDR4h
**Ppard**	1.01960161	0.00026589	L6a	LDR4h
**Ppargc1b**	0.67416304	1.52E-05	L6a	LDR4h
**Ppm1e**	0.59455132	2.37E-07	L6a	LDR4h
**Ppm1h**	1.10043103	2.33E-15	L6a	LDR4h

Development • Supplementary information

**Ppme1**	0.68812864	0.02682883	L6a	LDR4h
**Ppp1r16b**	0.68384023	0.01267591	L6a	LDR4h
**Prim2**	1.03310302	4.37E-15	L6a	LDR4h
**Ptp4a2**	0.79427286	2.69E-09	L6a	LDR4h
**Rai1**	0.78388008	5.93E-06	L6a	LDR4h
**Rb1**	0.71191837	2.27E-12	L6a	LDR4h
**Rcan2**	0.8504362	1.41E-05	L6a	LDR4h
**Rcor1**	0.67573207	7.62E-06	L6a	LDR4h
**Relb**	1.12995645	0.00245602	L6a	LDR4h
**Retreg1**	0.65880278	1.63E-07	L6a	LDR4h
**Rgs20**	0.76647888	0.01185788	L6a	LDR4h
**Rhoq**	1.09728745	4.05E-09	L6a	LDR4h
**Ripor1**	0.69591627	0.01818036	L6a	LDR4h
**Rnf217**	0.72534842	1.36E-09	L6a	LDR4h
**Rph3a**	0.59086449	4.67E-05	L6a	LDR4h
**Samd4**	0.65686345	0.03678162	L6a	LDR4h
**Schip1**	0.79358196	2.19E-06	L6a	LDR4h
**Scube1**	0.73615803	0.02258228	L6a	LDR4h
**Sema3e**	0.76434479	0.00025886	L6a	LDR4h
**Sgsm1**	1.3863055	6.46E-09	L6a	LDR4h
**Shank2**	0.71872329	0.00978627	L6a	LDR4h
**Sik2**	1.20972803	6.28E-10	L6a	LDR4h
**Sik3**	1.04821284	4.84E-17	L6a	LDR4h
**Slc6a17**	0.78184551	3.31E-14	L6a	LDR4h
**Slc6a8**	0.79256676	0.04705567	L6a	LDR4h
**Slc9a5**	1.41262875	1.16E-18	L6a	LDR4h
**Smad3**	1.29402863	3.47E-17	L6a	LDR4h
**Smg7**	0.69191591	6.46E-08	L6a	LDR4h
**Socs7**	0.65958429	5.45E-05	L6a	LDR4h
**Sorcs1**	0.74331506	3.45E-05	L6a	LDR4h
**Sorcs3**	1.99740091	9.06E-22	L6a	LDR4h
**Spns2**	0.76477359	0.03231092	L6a	LDR4h
**Spred2**	0.88020438	0.00099185	L6a	LDR4h
**St8sia5**	0.61898524	1.36E-06	L6a	LDR4h
**Stk10**	0.70911088	0.0041353	L6a	LDR4h
**Stx4a**	0.99363156	0.00101472	L6a	LDR4h
**Tet2**	0.61145661	2.34E-06	L6a	LDR4h
**Tet3**	1.1915261	1.95E-17	L6a	LDR4h
**Tjp2**	0.99966264	0.00267968	L6a	LDR4h

Development • Supplementary information

**Tmem178**	0.72349631	7.10E-06	L6a	LDR4h
**Trim9**	0.75366398	2.81E-12	L6a	LDR4h
**Tyro3**	0.76151205	0.0004996	L6a	LDR4h
**Ubash3b**	1.28325675	7.54E-20	L6a	LDR4h
**Ubtf**	0.70916057	0.04967698	L6a	LDR4h
**Unc13a**	0.59486446	4.54E-08	L6a	LDR4h
**Wdfy2**	0.73714142	0.00021216	L6a	LDR4h
**Xpo1**	0.72573713	8.07E-09	L6a	LDR4h
**Zdhhc14**	1.13284608	2.04E-06	L6a	LDR4h
**Zfc3h1**	0.70932552	4.05E-05	L6a	LDR4h
**Zfp407**	0.62565026	0.00227034	L6a	LDR4h
**Zfp710**	0.83925697	5.83E-06	L6a	LDR4h
**Zhx2**	0.94716795	1.18E-07	L6a	LDR4h
**Zmiz1**	1.46201398	6.16E-13	L6a	LDR4h
**Zswim6**	0.91871003	9.38E-11	L6a	LDR4h
**Pitpnc1**	-0.9727325	0.01685979	L6a	LDR6h
**Ank**	0.89065248	0.01599419	L6b	LDR30m
**Arhgef3**	1.21473516	4.56E-05	L6b	LDR30m
**Arl5b**	1.46883484	0.04870041	L6b	LDR30m
**Baiap2**	1.03603056	0.00172258	L6b	LDR30m
**Btaf1**	1.14146834	0.00035434	L6b	LDR30m
**Chst11**	1.38897421	3.38E-06	L6b	LDR30m
**Cpeb3**	1.22359575	9.77E-11	L6b	LDR30m
**Cpeb4**	1.06274128	0.00052601	L6b	LDR30m
**Egr3**	2.63438952	4.89E-12	L6b	LDR30m
**Fosb**	1.92589589	0.01290403	L6b	LDR30m
**Frmd6**	1.59039444	0.00552214	L6b	LDR30m
**Gfod1**	0.88195202	0.00350808	L6b	LDR30m
**Gm47423**	1.43584597	0.00240661	L6b	LDR30m
**Homer1**	1.91846299	0.00933375	L6b	LDR30m
**Lncpint**	0.76433687	0.00078914	L6b	LDR30m
**Mbnl2**	0.84570729	0.00642943	L6b	LDR30m
**Nedd4l**	1.0363171	0.00959246	L6b	LDR30m
**Nmnat2**	1.03708302	0.0001783	L6b	LDR30m
**Npas4**	2.04567192	5.30E-05	L6b	LDR30m
**Ntrk2**	0.86018773	3.20E-07	L6b	LDR30m
**Pdlim1**	1.47476468	0.00555342	L6b	LDR30m
**Per2**	1.68055061	0.01305112	L6b	LDR30m
**Pik3r3**	1.06327621	0.04229706	L6b	LDR30m

**Psd3**	0.89268734	0.00019812	L6b	LDR30m
**Rheb**	1.33412103	0.0098853	L6b	LDR30m
**Rock2**	0.99162489	0.00020024	L6b	LDR30m
**Sik2**	1.27640372	1.42E-05	L6b	LDR30m
**Slc6a17**	0.91661882	0.01518222	L6b	LDR30m
**Tmem178**	1.00750474	0.0015207	L6b	LDR30m
**Tulp4**	1.2148869	0.00018212	L6b	LDR30m
**Ube2ql1**	1.86334925	3.28E-06	L6b	LDR30m
**Zdbf2**	1.55513667	0.02465338	L6b	LDR30m
**Zswim6**	1.19173854	0.00648821	L6b	LDR30m
**Airn**	1.52874159	0.01272429	L6b	LDR4h
**Car10**	1.06531059	9.28E-05	L6b	LDR4h
**Dgki**	0.61575891	0.00178319	L6b	LDR4h
**Dock4**	0.92337087	0.04396505	L6b	LDR4h
**Gfod1**	0.79447597	0.01207799	L6b	LDR4h
**Gramd1b**	0.76875123	0.01237846	L6b	LDR4h
**Grin2a**	0.78891671	0.00027769	L6b	LDR4h
**Mamld1**	1.36527562	0.04200077	L6b	LDR4h
**Mapk4**	1.39081381	0.0003974	L6b	LDR4h
**Nedd4l**	0.87116887	0.01929853	L6b	LDR4h
**Ntrk2**	0.58895838	0.00153749	L6b	LDR4h
**Ppm1h**	0.85148908	0.03345419	L6b	LDR4h
**Rgs7**	0.6848233	0.01408337	L6b	LDR4h
**Sgsm1**	1.20032142	0.00013265	L6b	LDR4h
**Sik2**	1.00571488	0.0129215	L6b	LDR4h
**Slit3**	1.10204506	0.00019737	L6b	LDR4h
**Sorcs3**	0.99595707	0.00152864	L6b	LDR4h
**Tmem178**	1.10942866	7.79E-05	L6b	LDR4h
**Abca1**	0.86925646	0.03067387	Mg	LDR4h
**Apbb1ip**	-0.8254487	0.00157426	Mg	LDR4h
**Arhgap5**	-0.7929251	0.02764495	Mg	LDR4h
**Homer1**	1.51969759	0.01164666	Mg	LDR4h
**Ly86**	-1.0401493	0.00141375	Mg	LDR4h
**H3f3b**	1.27527311	0.03254062	Mg	LDR6h
**Gria1**	0.79621019	6.74E-05	Npy	LDR
**1700016P03Rik**	1.71901613	4.58E-05	Npy	LDR30m
**Homer1**	1.24418458	0.00037082	Npy	LDR30m
**Npy**	-0.8231953	0.04843097	Npy	LDR30m
**Gria1**	0.58721859	0.01713031	Npy	LDR4h

Development • Supplementary information

**Mgat5**	0.66253647	0.01058632	Olg	LDR
**Prom1**	0.93482894	0.00242699	Olg	LDR
**5830416I19Rik**	1.40284325	0.04850252	Olg	LDR2h
**Gm47423**	1.71045572	0.0140789	Olg	LDR30m
**Mgat5**	0.73072837	0.00331832	Olg	LDR30m
**Nr4a2**	1.96625082	8.05E-05	Olg	LDR30m
**Parm1**	0.76951272	0.03016974	Olg	LDR30m
**Zdbf2**	1.55609043	0.03143667	Olg	LDR30m
**Galnt9**	1.40408144	0.0465158	Olg	LDR4h
**Iqgap2**	1.36350857	0.04121688	Olg	LDR4h
**Pip5k1b**	0.98481317	0.01927925	Olg	LDR4h
**Sgsm1**	1.45705339	0.00272713	Olg	LDR4h
**Slc4a10**	1.1181252	0.04746633	Olg	LDR4h
**Spred2**	1.27272084	0.01556371	Olg	LDR4h
**Tmem178**	1.19331752	0.04521554	Olg	LDR4h
**Trim9**	1.43929984	0.00061677	Olg	LDR4h
**Cntnap5c**	-0.6328008	0.00120944	Pval	LDR
**Fgf1**	-0.6532933	0.00047796	Pval	LDR
**Mir670hg**	2.05691259	0.02365792	Pval	LDR2h
**Slc1a3**	1.42665168	0.02163015	Pval	LDR2h
**Xlr5a**	1.45670183	0.02937825	Pval	LDR2h
**Arid5b**	0.78338864	0.00137163	Pval	LDR30m
**Cdh4**	1.10072168	0.00716057	Pval	LDR30m
**Eepd1**	0.95053421	0.00152597	Pval	LDR30m
**Fosl2**	1.67504033	1.90E-10	Pval	LDR30m
**Gad1**	0.64589511	5.98E-05	Pval	LDR30m
**Gli3**	1.33289851	0.01602978	Pval	LDR30m
**Homer1**	1.54899121	0.00121116	Pval	LDR30m
**Mef2d**	0.83083298	0.02528678	Pval	LDR30m
**Nr4a2**	1.38960794	0.00235518	Pval	LDR30m
**Nr4a3**	2.2789387	0.00225724	Pval	LDR30m
**Pcsk1**	1.62926592	7.84E-07	Pval	LDR30m
**Plxnc1**	0.9417509	1.80E-08	Pval	LDR30m
**Ppargc1b**	0.66516602	0.04680841	Pval	LDR30m
**Rgs7bp**	0.68253359	8.78E-08	Pval	LDR30m
**Rock2**	0.61946228	0.049574	Pval	LDR30m
**Sik3**	0.68648831	0.00137666	Pval	LDR30m
**Slc6a17**	0.59996831	0.00528313	Pval	LDR30m
**Gad1**	0.65906822	7.08E-07	Pval	LDR4h

Development • Supplementary information

**Gfod1**	0.6886359	0.02312333	Pval	LDR4h
**Homer1**	0.88643742	0.00101165	Pval	LDR4h
**Htr2a**	0.80916506	0.01150818	Pval	LDR4h
**Maml3**	0.94867729	0.01388439	Pval	LDR4h
**Mir670hg**	1.91528199	2.99E-08	Pval	LDR4h
**Plxnc1**	0.66771974	0.00183914	Pval	LDR4h
**Rock2**	0.62977698	0.00022733	Pval	LDR4h
**Sik3**	0.70126842	6.81E-06	Pval	LDR4h
**Slc6a17**	0.68571264	4.95E-07	Pval	LDR4h
**Mir670hg**	2.01663211	3.16E-09	Vip	LDR2h
**Tll1**	2.01232172	0.00505495	Vip	LDR2h
**1700016P03Rik**	2.72019504	2.76E-21	Vip	LDR30m
**Atp2c1**	1.13639202	0.00788478	Vip	LDR30m
**Dlx6os1**	1.04159882	0.00023182	Vip	LDR30m
**Fam107b**	2.55803925	5.55E-08	Vip	LDR30m
**Fosb**	2.46731004	1.90E-06	Vip	LDR30m
**Fosl2**	2.59477369	1.76E-09	Vip	LDR30m
**Gad1**	1.41647453	1.38E-06	Vip	LDR30m
**Gm47423**	1.32093318	0.02536835	Vip	LDR30m
**Homer1**	1.94868419	3.68E-11	Vip	LDR30m
**Mest**	1.51475669	0.01740918	Vip	LDR30m
**Nr4a2**	2.77071967	4.24E-08	Vip	LDR30m
**Nr4a3**	3.50946199	0.02733404	Vip	LDR30m
**Pcsk1**	1.69905199	1.32E-06	Vip	LDR30m
**Rab6a**	1.44140118	5.34E-06	Vip	LDR30m
**Rock2**	0.91220705	0.00309788	Vip	LDR30m
**Sipa1l2**	1.60463708	0.01570471	Vip	LDR30m
**Zswim6**	1.04475699	0.02392231	Vip	LDR30m
**Col19a1**	1.19658664	0.01652156	Vip	LDR4h
**Gabra2**	0.78558031	0.04533462	Vip	LDR4h
**Gfod1**	1.23288847	0.00027641	Vip	LDR4h
**Mir670hg**	1.34729124	1.22E-09	Vip	LDR4h
**Nectin3**	1.11336811	0.00848249	Vip	LDR4h
**Nos1ap**	1.05497051	0.02677918	Vip	LDR4h
**Pcdh11x**	1.34099748	0.00053224	Vip	LDR4h
**Trpc6**	1.65668513	0.00016776	Vip	LDR4h
**Zswim6**	1.07810521	0.00035595	Vip	LDR4h

Development • Supplementary information

Table S4. Genes identified as induced or repressed based upon RNA velocity.

	**Cell type**	**Pattern**
**AC149090.1**	ExcL23	induction
**Ptprn**	ExcL23	induction
**Scg2**	ExcL23	induction
**Btg2**	ExcL23	induction
**Adora1**	ExcL23	induction
**Igfn1**	ExcL23	induction
**Rgs2**	ExcL23	induction
**Rgs4**	ExcL23	induction
**Atp1a2**	ExcL23	induction
**Bzw1**	ExcL23	induction
**Zdbf2**	ExcL23	induction
**Tmem198**	ExcL23	induction
**Inpp5d**	ExcL23	induction
**Ptgs2**	ExcL23	induction
**Cd34**	ExcL23	induction
**Ddx50**	ExcL23	induction
**Zfp365**	ExcL23	induction
**Cbarp**	ExcL23	induction
**Slc25a3**	ExcL23	induction
**Cpsf6**	ExcL23	induction
**Lemd3**	ExcL23	induction
**Pip4k2c**	ExcL23	induction
**P4ha1**	ExcL23	induction
**Arhgap45**	ExcL23	induction
**Atp5d**	ExcL23	induction
**Midn**	ExcL23	induction
**Gadd45b**	ExcL23	induction
**Txnrd1**	ExcL23	induction
**Dusp6**	ExcL23	induction
**Kitl**	ExcL23	induction
**Gls2**	ExcL23	induction
**Hspa4**	ExcL23	induction
**Ndel1**	ExcL23	induction
**Kdm6b**	ExcL23	induction

Development • Supplementary information

**Eif4a1**	ExcL23	induction
**Dusp14**	ExcL23	induction
**Cwc25**	ExcL23	induction
**Dusp3**	ExcL23	induction
**Usp36**	ExcL23	induction
**Wdr45b**	ExcL23	induction
**Cpeb4**	ExcL23	induction
**Ppp2ca**	ExcL23	induction
**Per1**	ExcL23	induction
**Thra**	ExcL23	induction
**A830036E02Rik**	ExcL23	induction
**Rundc1**	ExcL23	induction
**Arl4d**	ExcL23	induction
**Baiap2**	ExcL23	induction
**Ifrd1**	ExcL23	induction
**Fbxo33**	ExcL23	induction
**Hsp90aa1**	ExcL23	induction
**Ckb**	ExcL23	induction
**Tmem179**	ExcL23	induction
**Fos**	ExcL23	induction
**Nrn1**	ExcL23	induction
**Ube2ql1**	ExcL23	induction
**Nr2f1**	ExcL23	induction
**Hmgcr**	ExcL23	induction
**Fst**	ExcL23	induction
**Brd9**	ExcL23	induction
**Pcsk1**	ExcL23	induction
**Lysmd3**	ExcL23	induction
**Nefm**	ExcL23	induction
**Nefl**	ExcL23	induction
**Tpt1**	ExcL23	induction
**Ndfip2**	ExcL23	induction
**Dnajc21**	ExcL23	induction
**Pou6f1**	ExcL23	induction
**Golph3**	ExcL23	induction
**Pfkm**	ExcL23	induction
**Grasp**	ExcL23	induction
**Nr4a1**	ExcL23	induction
**Txndc11**	ExcL23	induction

Development • Supplementary information

**Plcxd2**	ExcL23	induction
**Nrip1**	ExcL23	induction
**Aifm3**	ExcL23	induction
**Ephb3**	ExcL23	induction
**Tfrc**	ExcL23	induction
**Kpna1**	ExcL23	induction
**Dcbld2**	ExcL23	induction
**Rhot2**	ExcL23	induction
**Sik1**	ExcL23	induction
**Stk19**	ExcL23	induction
**Cdc42ep3**	ExcL23	induction
**Gnl1**	ExcL23	induction
**Gm26917**	ExcL23	induction
**Syt4**	ExcL23	induction
**Nrep**	ExcL23	induction
**Etf1**	ExcL23	induction
**Csf1r**	ExcL23	induction
**Csnk1a1**	ExcL23	induction
**Npas4**	ExcL23	induction
**Rorb**	ExcL23	induction
**Sptbn2**	ExcL23	induction
**Sf1**	ExcL23	induction
**Syt7**	ExcL23	induction
**Minpp1**	ExcL23	induction
**Ina**	ExcL23	induction
**Cry2**	ExcL23	induction
**Necab3**	ExcL23	induction
**Cbln4**	ExcL23	induction
**Pmepa1**	ExcL23	induction
**Stmn3**	ExcL23	induction
**Arl5b**	ExcL23	induction
**Rif1**	ExcL23	induction
**Gm13629**	ExcL23	induction
**Slc1a2**	ExcL23	induction
**Bdnf**	ExcL23	induction
**Mertk**	ExcL23	induction
**Chgb**	ExcL23	induction
**Dlgap4**	ExcL23	induction
**Ywhab**	ExcL23	induction

**Setd7**	ExcL23	induction
**Siah2**	ExcL23	induction
**Adam15**	ExcL23	induction
**Ciart**	ExcL23	induction
**Atp1a1**	ExcL23	induction
**Ntng1**	ExcL23	induction
**Ccn1**	ExcL23	induction
**Prkacb**	ExcL23	induction
**Skil**	ExcL23	induction
**Tiparp**	ExcL23	induction
**Map9**	ExcL23	induction
**Mcl1**	ExcL23	induction
**Taf13**	ExcL23	induction
**Pdp1**	ExcL23	induction
**Tmem245**	ExcL23	induction
**Frrs1l**	ExcL23	induction
**Whrn**	ExcL23	induction
**Plk3**	ExcL23	induction
**Szt2**	ExcL23	induction
**Ptpru**	ExcL23	induction
**Efhd2**	ExcL23	induction
**Dnajb5**	ExcL23	induction
**Nr4a3**	ExcL23	induction
**Rnf20**	ExcL23	induction
**Nrd1**	ExcL23	induction
**Rims3**	ExcL23	induction
**Epha10**	ExcL23	induction
**Stk40**	ExcL23	induction
**Rcc2**	ExcL23	induction
**Chd5**	ExcL23	induction
**Cdk11b**	ExcL23	induction
**Rheb**	ExcL23	induction
**Lgi2**	ExcL23	induction
**Grk3**	ExcL23	induction
**Cabp1**	ExcL23	induction
**Atp2a2**	ExcL23	induction
**Ywhag**	ExcL23	induction
**Actb**	ExcL23	induction
**Hsph1**	ExcL23	induction

**Fosl2**	ExcL23	induction
**Parm1**	ExcL23	induction
**Cds1**	ExcL23	induction
**Ephx4**	ExcL23	induction
**Btbd8**	ExcL23	induction
**Por**	ExcL23	induction
**Nptx2**	ExcL23	induction
**Kdm7a**	ExcL23	induction
**Tet3**	ExcL23	induction
**Cnbp**	ExcL23	induction
**Clstn3**	ExcL23	induction
**Ccnd2**	ExcL23	induction
**Gpr19**	ExcL23	induction
**Mest**	ExcL23	induction
**Peg3**	ExcL23	induction
**Fosb**	ExcL23	induction
**Pvr**	ExcL23	induction
**Erf**	ExcL23	induction
**Chd2**	ExcL23	induction
**Nrip3**	ExcL23	induction
**Oat**	ExcL23	induction
**Numbl**	ExcL23	induction
**Fbl**	ExcL23	induction
**Pak1**	ExcL23	induction
**Lonrf1**	ExcL23	induction
**Ing2**	ExcL23	induction
**Zfp869**	ExcL23	induction
**Smad1**	ExcL23	induction
**Smarca5**	ExcL23	induction
**Atp6v0d1**	ExcL23	induction
**Ccsap**	ExcL23	induction
**Arhgef7**	ExcL23	induction
**Plpbp**	ExcL23	induction
**Saraf**	ExcL23	induction
**Dnajb1**	ExcL23	induction
**Gpt2**	ExcL23	induction
**Cx3cl1**	ExcL23	induction
**Zfhx3**	ExcL23	induction
**Tsnax**	ExcL23	induction

Development • Supplementary information

**Nrp1**	ExcL23	induction
**Dpy19l1**	ExcL23	induction
**AI593442**	ExcL23	induction
**Ubl5**	ExcL23	induction
**Igsf9b**	ExcL23	induction
**Hspa8**	ExcL23	induction
**Anp32a**	ExcL23	induction
**Arpp19**	ExcL23	induction
**Rassf1**	ExcL23	induction
**Usp19**	ExcL23	induction
**Trak1**	ExcL23	induction
**Prkx**	ExcL23	induction
**Sat1**	ExcL23	induction
**Ddx3y**	ExcL23	induction
**Ikzf2**	ExcL23	repression
**Lypd1**	ExcL23	repression
**Cntnap5a**	ExcL23	repression
**Ptpn14**	ExcL23	repression
**Marcks**	ExcL23	repression
**Plk5**	ExcL23	repression
**Timp2**	ExcL23	repression
**Col23a1**	ExcL23	repression
**Arhgef28**	ExcL23	repression
**Ccn3**	ExcL23	repression
**Celf4**	ExcL23	repression
**Wnt4**	ExcL23	repression
**Rilpl1**	ExcL23	repression
**Met**	ExcL23	repression
**Prkcg**	ExcL23	repression
**Necab2**	ExcL23	repression
**Cpne7**	ExcL23	repression
**Pygo1**	ExcL23	repression
**Tenm1**	ExcL23	repression
**Pgap1**	ExcL23	induction
**Ptprn**	ExcL23	induction
**Scg2**	ExcL23	induction
**Per2**	ExcL23	induction
**Btg2**	ExcL23	induction
**Nav1**	ExcL23	induction

**Rgs4**	ExcL23	induction
**R3hdm1**	ExcL23	induction
**Ptgs2**	ExcL23	induction
**Eprs**	ExcL23	induction
**Nudt4**	ExcL23	induction
**Trhde**	ExcL23	induction
**Egr2**	ExcL23	induction
**Midn**	ExcL23	induction
**Dot1l**	ExcL23	induction
**Gadd45b**	ExcL23	induction
**Pip5k1c**	ExcL23	induction
**Nt5dc3**	ExcL23	induction
**Nap1l1**	ExcL23	induction
**Kdm6b**	ExcL23	induction
**Dusp14**	ExcL23	induction
**Xpo1**	ExcL23	induction
**Camkk1**	ExcL23	induction
**Baiap2**	ExcL23	induction
**Ifrd1**	ExcL23	induction
**Baz1a**	ExcL23	induction
**Eml5**	ExcL23	induction
**Coch**	ExcL23	induction
**Frmd6**	ExcL23	induction
**Fos**	ExcL23	induction
**Cdk13**	ExcL23	induction
**Gm32036**	ExcL23	induction
**Map1b**	ExcL23	induction
**Inhba**	ExcL23	induction
**Gadd45g**	ExcL23	induction
**Pcsk1**	ExcL23	induction
**Enc1**	ExcL23	induction
**Nefm**	ExcL23	induction
**Spry2**	ExcL23	induction
**Nefl**	ExcL23	induction
**Egr3**	ExcL23	induction
**Myh9**	ExcL23	induction
**Cacng2**	ExcL23	induction
**Scube1**	ExcL23	induction
**Pou6f1**	ExcL23	induction

**Lrrk2**	ExcL23	induction
**Grasp**	ExcL23	induction
**2510009E07Rik**	ExcL23	induction
**Etv5**	ExcL23	induction
**Arhgap31**	ExcL23	induction
**Plcxd2**	ExcL23	induction
**Mas1**	ExcL23	induction
**Pim1**	ExcL23	induction
**Gm26917**	ExcL23	induction
**Egr1**	ExcL23	induction
**Csnk1a1**	ExcL23	induction
**D730045A05Rik**	ExcL23	induction
**Cbln2**	ExcL23	induction
**Npas4**	ExcL23	induction
**Malat1**	ExcL23	induction
**Rorb**	ExcL23	induction
**Sptbn2**	ExcL23	induction
**Fth1**	ExcL23	induction
**Hectd2**	ExcL23	induction
**Ina**	ExcL23	induction
**C1ql3**	ExcL23	induction
**Rnd3**	ExcL23	induction
**Dgkz**	ExcL23	induction
**Cbln4**	ExcL23	induction
**Slc1a2**	ExcL23	induction
**Bdnf**	ExcL23	induction
**Spred1**	ExcL23	induction
**Pak6**	ExcL23	induction
**Mertk**	ExcL23	induction
**Mrps26**	ExcL23	induction
**Epb41l1**	ExcL23	induction
**Dlgap4**	ExcL23	induction
**Ppp1r16b**	ExcL23	induction
**Npnt**	ExcL23	induction
**Ccn1**	ExcL23	induction
**Gm37229**	ExcL23	induction
**Tiparp**	ExcL23	induction
**Gm3764**	ExcL23	induction
**Igsf3**	ExcL23	induction

Development • Supplementary information

**Penk**	ExcL23	induction
**Tnc**	ExcL23	induction
**Map7d1**	ExcL23	induction
**Efhd2**	ExcL23	induction
**Disp3**	ExcL23	induction
**Tgfbr1**	ExcL23	induction
**Nrd1**	ExcL23	induction
**Rims3**	ExcL23	induction
**Hpcal4**	ExcL23	induction
**Epha10**	ExcL23	induction
**Stk40**	ExcL23	induction
**Rheb**	ExcL23	induction
**Arap2**	ExcL23	induction
**Prkg2**	ExcL23	induction
**Sgsm1**	ExcL23	induction
**Cabp1**	ExcL23	induction
**Rph3a**	ExcL23	induction
**Ncor2**	ExcL23	induction
**Pdgfa**	ExcL23	induction
**Actb**	ExcL23	induction
**Fosl2**	ExcL23	induction
**Galnt9**	ExcL23	induction
**Nptx2**	ExcL23	induction
**Kdm7a**	ExcL23	induction
**Osbpl3**	ExcL23	induction
**Tet3**	ExcL23	induction
**Clstn3**	ExcL23	induction
**Dennd5b**	ExcL23	induction
**Fosb**	ExcL23	induction
**Shank1**	ExcL23	induction
**Acan**	ExcL23	induction
**Mir9-3hg**	ExcL23	induction
**Gdpd5**	ExcL23	induction
**Rab6a**	ExcL23	induction
**Tacc1**	ExcL23	induction
**Junb**	ExcL23	induction
**Arhgef7**	ExcL23	induction
**Dnajb1**	ExcL23	induction
**Cacna1a**	ExcL23	induction

Development • Supplementary information

**Cx3cl1**	ExcL23	induction
**Slc9a5**	ExcL23	induction
**6430548M08Rik**	ExcL23	induction
**Dpy19l1**	ExcL23	induction
**Fam81a**	ExcL23	induction
**Scg3**	ExcL23	induction
**Igsf9b**	ExcL23	induction
**Hspa8**	ExcL23	induction
**Prkar2a**	ExcL23	induction
**AC149090.1**	ExcL23	repression
**Kdsr**	ExcL23	repression
**Atp2b4**	ExcL23	repression
**Atp1a2**	ExcL23	repression
**Vxn**	ExcL23	repression
**Cntnap5a**	ExcL23	repression
**Ptpn14**	ExcL23	repression
**Kif5a**	ExcL23	repression
**Spock2**	ExcL23	repression
**Plk5**	ExcL23	repression
**Timp3**	ExcL23	repression
**Limk2**	ExcL23	repression
**Chd3**	ExcL23	repression
**Adcy1**	ExcL23	repression
**Nsg2**	ExcL23	repression
**Col23a1**	ExcL23	repression
**Slc16a11**	ExcL23	repression
**Sez6**	ExcL23	repression
**Thra**	ExcL23	repression
**Eif1**	ExcL23	repression
**Mgat5b**	ExcL23	repression
**Tnrc6c**	ExcL23	repression
**Arhgef28**	ExcL23	repression
**Cpne6**	ExcL23	repression
**Elfn2**	ExcL23	repression
**Tuba1a**	ExcL23	repression
**Fam173a**	ExcL23	repression
**Slc6a7**	ExcL23	repression
**Tcf4**	ExcL23	repression
**Ptgds**	ExcL23	repression

Development • Supplementary information

**Scn3a**	ExcL23	repression
**Mpped2**	ExcL23	repression
**Rps21**	ExcL23	repression
**6430590A07Rik**	ExcL23	repression
**St6galnac5**	ExcL23	repression
**Ankrd6**	ExcL23	repression
**Nfib**	ExcL23	repression
**Crocc**	ExcL23	repression
**Raver2**	ExcL23	repression
**Epha5**	ExcL23	repression
**Grk3**	ExcL23	repression
**Tmem243**	ExcL23	repression
**Ablim2**	ExcL23	repression
**Uchl1**	ExcL23	repression
**Hrk**	ExcL23	repression
**Thsd7a**	ExcL23	repression
**Gsdme**	ExcL23	repression
**Srgap3**	ExcL23	repression
**Adcyap1r1**	ExcL23	repression
**Mcf2l**	ExcL23	repression
**D830024N08Rik**	ExcL23	repression
**Mt3**	ExcL23	repression
**Cpne7**	ExcL23	repression
**Icam5**	ExcL23	repression
**Fxyd6**	ExcL23	repression
**Dalrd3**	ExcL23	repression
**Tenm1**	ExcL23	repression
**AC149090.1**	ExcL23	induction
**Igfn1**	ExcL23	induction
**Atp1a2**	ExcL23	induction
**Ptgs2**	ExcL23	induction
**Eprs**	ExcL23	induction
**Trhde**	ExcL23	induction
**Rpl41**	ExcL23	induction
**Dot1l**	ExcL23	induction
**Chd3**	ExcL23	induction
**Col23a1**	ExcL23	induction
**Camkk1**	ExcL23	induction
**Thra**	ExcL23	induction

**Eif1**	ExcL23	induction
**Baz1a**	ExcL23	induction
**Tmem179**	ExcL23	induction
**Inf2**	ExcL23	induction
**Gm32036**	ExcL23	induction
**Adamts16**	ExcL23	induction
**Nr2f1**	ExcL23	induction
**A430090L17Rik**	ExcL23	induction
**Inhba**	ExcL23	induction
**Ly6h**	ExcL23	induction
**Nptxr**	ExcL23	induction
**Tuba1a**	ExcL23	induction
**Pou6f1**	ExcL23	induction
**Ccn3**	ExcL23	induction
**Arhgap31**	ExcL23	induction
**Pcp4**	ExcL23	induction
**Mas1**	ExcL23	induction
**Dact2**	ExcL23	induction
**Pim1**	ExcL23	induction
**Gm26917**	ExcL23	induction
**Celf4**	ExcL23	induction
**Nrep**	ExcL23	induction
**Slc6a7**	ExcL23	induction
**Csf1r**	ExcL23	induction
**Malat1**	ExcL23	induction
**Sptbn2**	ExcL23	induction
**Fth1**	ExcL23	induction
**Scd2**	ExcL23	induction
**C1ql3**	ExcL23	induction
**Ptgds**	ExcL23	induction
**1110008P14Rik**	ExcL23	induction
**Rnd3**	ExcL23	induction
**Commd3**	ExcL23	induction
**Dipk1b**	ExcL23	induction
**Dab2ip**	ExcL23	induction
**Mrps26**	ExcL23	induction
**Dlgap4**	ExcL23	induction
**Rps21**	ExcL23	induction
**Sertm1**	ExcL23	induction

Development • Supplementary information

**Adam15**	ExcL23	induction
**Npnt**	ExcL23	induction
**Gm37229**	ExcL23	induction
**Gm20089**	ExcL23	induction
**Penk**	ExcL23	induction
**Rps8**	ExcL23	induction
**Disp3**	ExcL23	induction
**Rnf20**	ExcL23	induction
**Hpcal4**	ExcL23	induction
**Epha10**	ExcL23	induction
**Wnt4**	ExcL23	induction
**Chd5**	ExcL23	induction
**Arap2**	ExcL23	induction
**Ociad2**	ExcL23	induction
**Prkg2**	ExcL23	induction
**Rph3a**	ExcL23	induction
**Taok3**	ExcL23	induction
**Nptx2**	ExcL23	induction
**Osbpl3**	ExcL23	induction
**Gm15594**	ExcL23	induction
**Peg3**	ExcL23	induction
**Cpt1c**	ExcL23	induction
**Shank1**	ExcL23	induction
**Gdpd5**	ExcL23	induction
**Ypel3**	ExcL23	induction
**Clec18a**	ExcL23	induction
**Maf**	ExcL23	induction
**D830024N08Rik**	ExcL23	induction
**Mt3**	ExcL23	induction
**Slc9a5**	ExcL23	induction
**Tsnax**	ExcL23	induction
**Gramd1b**	ExcL23	induction
**Igsf9b**	ExcL23	induction
**Gm15520**	ExcL23	induction
**Dalrd3**	ExcL23	induction
**Gm26652**	ExcL23	induction
**Tenm1**	ExcL23	induction
**Tuba4a**	ExcL23	repression
**Scg2**	ExcL23	repression

Development • Supplementary information

**Atp2b4**	ExcL23	repression
**Btg2**	ExcL23	repression
**Adora1**	ExcL23	repression
**Rgs2**	ExcL23	repression
**Rgs4**	ExcL23	repression
**Zdbf2**	ExcL23	repression
**Inpp5d**	ExcL23	repression
**Ptpn14**	ExcL23	repression
**Cbarp**	ExcL23	repression
**Slc25a3**	ExcL23	repression
**Nudt4**	ExcL23	repression
**P4ha1**	ExcL23	repression
**Spock2**	ExcL23	repression
**Egr2**	ExcL23	repression
**Arhgap45**	ExcL23	repression
**Plk5**	ExcL23	repression
**Gadd45b**	ExcL23	repression
**Kitl**	ExcL23	repression
**Nap1l1**	ExcL23	repression
**Gls2**	ExcL23	repression
**Hspa4**	ExcL23	repression
**Ndel1**	ExcL23	repression
**Kdm6b**	ExcL23	repression
**Eif4a1**	ExcL23	repression
**Dusp14**	ExcL23	repression
**Cltc**	ExcL23	repression
**Hlf**	ExcL23	repression
**Cwc25**	ExcL23	repression
**Stat3**	ExcL23	repression
**Usp36**	ExcL23	repression
**Cpeb4**	ExcL23	repression
**Ebf1**	ExcL23	repression
**Ppp2ca**	ExcL23	repression
**Per1**	ExcL23	repression
**A830036E02Rik**	ExcL23	repression
**Rundc1**	ExcL23	repression
**Arl4d**	ExcL23	repression
**Mgat5b**	ExcL23	repression
**Baiap2**	ExcL23	repression

**Ifrd1**	ExcL23	repression
**Fbxo33**	ExcL23	repression
**Hsp90aa1**	ExcL23	repression
**Ckb**	ExcL23	repression
**Fos**	ExcL23	repression
**Nrn1**	ExcL23	repression
**Hmgcr**	ExcL23	repression
**Arhgef28**	ExcL23	repression
**Fst**	ExcL23	repression
**BC005537**	ExcL23	repression
**Gadd45g**	ExcL23	repression
**Pcsk1**	ExcL23	repression
**Jph4**	ExcL23	repression
**Ptk2b**	ExcL23	repression
**Nefm**	ExcL23	repression
**Nefl**	ExcL23	repression
**Tpt1**	ExcL23	repression
**Ndfip2**	ExcL23	repression
**Elfn2**	ExcL23	repression
**Golph3**	ExcL23	repression
**Matn2**	ExcL23	repression
**Trib1**	ExcL23	repression
**Grasp**	ExcL23	repression
**Txndc11**	ExcL23	repression
**Plcxd2**	ExcL23	repression
**Nrip1**	ExcL23	repression
**Ephb3**	ExcL23	repression
**Tfrc**	ExcL23	repression
**Rhot2**	ExcL23	repression
**Sik1**	ExcL23	repression
**Cdc42ep3**	ExcL23	repression
**Zfp948**	ExcL23	repression
**Pkd1**	ExcL23	repression
**Osbpl1a**	ExcL23	repression
**Syt4**	ExcL23	repression
**Etf1**	ExcL23	repression
**Tcf4**	ExcL23	repression
**Npas4**	ExcL23	repression
**Rorb**	ExcL23	repression

Development • Supplementary information

**Cnnm1**	ExcL23	repression
**Ina**	ExcL23	repression
**Itga8**	ExcL23	repression
**Slc25a25**	ExcL23	repression
**Ctnnd1**	ExcL23	repression
**Cry2**	ExcL23	repression
**Rims4**	ExcL23	repression
**Adnp**	ExcL23	repression
**Bcas1**	ExcL23	repression
**Cbln4**	ExcL23	repression
**Pmepa1**	ExcL23	repression
**Arl5b**	ExcL23	repression
**Gm13629**	ExcL23	repression
**Bdnf**	ExcL23	repression
**Mertk**	ExcL23	repression
**Chgb**	ExcL23	repression
**Ttpal**	ExcL23	repression
**Ywhab**	ExcL23	repression
**Siah2**	ExcL23	repression
**Ciart**	ExcL23	repression
**Gnai3**	ExcL23	repression
**Ccn1**	ExcL23	repression
**Skil**	ExcL23	repression
**Hspa4l**	ExcL23	repression
**Tiparp**	ExcL23	repression
**Map9**	ExcL23	repression
**Mef2d**	ExcL23	repression
**Whrn**	ExcL23	repression
**Nfib**	ExcL23	repression
**Plk3**	ExcL23	repression
**Ptpru**	ExcL23	repression
**Maco1**	ExcL23	repression
**Crocc**	ExcL23	repression
**Efhd2**	ExcL23	repression
**Gem**	ExcL23	repression
**Nr4a3**	ExcL23	repression
**Tmem38b**	ExcL23	repression
**Nrd1**	ExcL23	repression
**Stk40**	ExcL23	repression

Development • Supplementary information

**Rcc2**	ExcL23	repression
**Rheb**	ExcL23	repression
**Uba6**	ExcL23	repression
**Grk3**	ExcL23	repression
**Cabp1**	ExcL23	repression
**Actb**	ExcL23	repression
**Hsph1**	ExcL23	repression
**Fosl2**	ExcL23	repression
**Ephx4**	ExcL23	repression
**Bmt2**	ExcL23	repression
**Kdm7a**	ExcL23	repression
**Tet3**	ExcL23	repression
**Clstn3**	ExcL23	repression
**Ccnd2**	ExcL23	repression
**Gpr19**	ExcL23	repression
**Mest**	ExcL23	repression
**Adcyap1r1**	ExcL23	repression
**Fosb**	ExcL23	repression
**Pvr**	ExcL23	repression
**Erf**	ExcL23	repression
**Chd2**	ExcL23	repression
**Iqgap1**	ExcL23	repression
**Nrip3**	ExcL23	repression
**Syt17**	ExcL23	repression
**Numbl**	ExcL23	repression
**Fbl**	ExcL23	repression
**Pak1**	ExcL23	repression
**Rab6a**	ExcL23	repression
**Irs2**	ExcL23	repression
**Lonrf1**	ExcL23	repression
**Ing2**	ExcL23	repression
**Smad1**	ExcL23	repression
**Smarca5**	ExcL23	repression
**Junb**	ExcL23	repression
**Dnajb1**	ExcL23	repression
**Gpt2**	ExcL23	repression
**Cx3cl1**	ExcL23	repression
**Cpne7**	ExcL23	repression
**Elovl4**	ExcL23	repression

Development • Supplementary information

**Hspa8**	ExcL23	repression
**Arpp19**	ExcL23	repression
**Gclc**	ExcL23	repression
**Elovl5**	ExcL23	repression
**Trak1**	ExcL23	repression
**Prkx**	ExcL23	repression
**Maged1**	ExcL23	repression
**Ddx3y**	ExcL23	repression
**AC149090.1**	ExcL23	induction
**Kdsr**	ExcL23	induction
**Atp2b4**	ExcL23	induction
**Atp1a2**	ExcL23	induction
**Inha**	ExcL23	induction
**Cntnap5a**	ExcL23	induction
**Ptpn14**	ExcL23	induction
**Spock2**	ExcL23	induction
**Adcy1**	ExcL23	induction
**Col23a1**	ExcL23	induction
**Slc16a11**	ExcL23	induction
**Mgat5b**	ExcL23	induction
**Clmn**	ExcL23	induction
**Inf2**	ExcL23	induction
**Arhgef28**	ExcL23	induction
**Cpne6**	ExcL23	induction
**Klf10**	ExcL23	induction
**Elfn2**	ExcL23	induction
**4930588J15Rik**	ExcL23	induction
**Rgs11**	ExcL23	induction
**Atp6v1g2**	ExcL23	induction
**Malat1**	ExcL23	induction
**Cox8a**	ExcL23	induction
**Rorb**	ExcL23	induction
**Ptgds**	ExcL23	induction
**Ano3**	ExcL23	induction
**Cst3**	ExcL23	induction
**Snhg11**	ExcL23	induction
**Trim2**	ExcL23	induction
**Ntng1**	ExcL23	induction
**Camk2d**	ExcL23	induction

Development • Supplementary information

**Nfib**	ExcL23	induction
**Clic4**	ExcL23	induction
**Crocc**	ExcL23	induction
**Uchl1**	ExcL23	induction
**Hrk**	ExcL23	induction
**Thsd7a**	ExcL23	induction
**Gsdme**	ExcL23	induction
**Adcyap1r1**	ExcL23	induction
**Junb**	ExcL23	induction
**D830024N08Rik**	ExcL23	induction
**Cpne7**	ExcL23	induction
**Car12**	ExcL23	induction
**Tenm1**	ExcL23	induction
**L1cam**	ExcL23	induction
**Tmsb4x**	ExcL23	induction
**Plp1**	ExcL23	induction
**Pgap1**	ExcL23	repression
**Ptprn**	ExcL23	repression
**Epha4**	ExcL23	repression
**Scg2**	ExcL23	repression
**Per2**	ExcL23	repression
**Btg2**	ExcL23	repression
**Nav1**	ExcL23	repression
**Rgs4**	ExcL23	repression
**Zdbf2**	ExcL23	repression
**R3hdm1**	ExcL23	repression
**Ptgs2**	ExcL23	repression
**Ivns1abp**	ExcL23	repression
**Eprs**	ExcL23	repression
**Rnf217**	ExcL23	repression
**Cep85l**	ExcL23	repression
**Cbarp**	ExcL23	repression
**Nudt4**	ExcL23	repression
**Ranbp2**	ExcL23	repression
**Zwint**	ExcL23	repression
**Midn**	ExcL23	repression
**Dot1l**	ExcL23	repression
**Gadd45b**	ExcL23	repression
**Nt5dc3**	ExcL23	repression

**Dusp6**	ExcL23	repression
**Kdm6b**	ExcL23	repression
**Abr**	ExcL23	repression
**Dusp14**	ExcL23	repression
**Xpo1**	ExcL23	repression
**Dlg4**	ExcL23	repression
**Baiap2**	ExcL23	repression
**Ifrd1**	ExcL23	repression
**Baz1a**	ExcL23	repression
**Eml5**	ExcL23	repression
**Rock2**	ExcL23	repression
**Coch**	ExcL23	repression
**Frmd6**	ExcL23	repression
**Zbtb1**	ExcL23	repression
**Fos**	ExcL23	repression
**Rapgef5**	ExcL23	repression
**Cdk13**	ExcL23	repression
**Gm32036**	ExcL23	repression
**Ube2ql1**	ExcL23	repression
**Hmgcr**	ExcL23	repression
**Map1b**	ExcL23	repression
**Pcsk1**	ExcL23	repression
**Fzd3**	ExcL23	repression
**Nefm**	ExcL23	repression
**Cdh6**	ExcL23	repression
**Azin1**	ExcL23	repression
**Ago2**	ExcL23	repression
**Myh9**	ExcL23	repression
**Nptxr**	ExcL23	repression
**Scube1**	ExcL23	repression
**A730060N03Rik**	ExcL23	repression
**Ccdc134**	ExcL23	repression
**Lrrk2**	ExcL23	repression
**Grasp**	ExcL23	repression
**2510009E07Rik**	ExcL23	repression
**Etv5**	ExcL23	repression
**Arhgap31**	ExcL23	repression
**Plcxd2**	ExcL23	repression
**Ephb3**	ExcL23	repression

Development • Supplementary information

**Qk**	ExcL23	repression
**Mas1**	ExcL23	repression
**Slc8a1**	ExcL23	repression
**Pim1**	ExcL23	repression
**Nrep**	ExcL23	repression
**Jcad**	ExcL23	repression
**Csnk1a1**	ExcL23	repression
**D730045A05Rik**	ExcL23	repression
**Cbln2**	ExcL23	repression
**Tm9sf3**	ExcL23	repression
**Sptbn2**	ExcL23	repression
**Hectd2**	ExcL23	repression
**1110008P14Rik**	ExcL23	repression
**Rnd3**	ExcL23	repression
**Dgkz**	ExcL23	repression
**Mir670hg**	ExcL23	repression
**Rasgrp1**	ExcL23	repression
**Zmynd8**	ExcL23	repression
**Cbln4**	ExcL23	repression
**Pmepa1**	ExcL23	repression
**Col5a1**	ExcL23	repression
**Dab2ip**	ExcL23	repression
**Tnfaip6**	ExcL23	repression
**Slc1a2**	ExcL23	repression
**Bdnf**	ExcL23	repression
**Spred1**	ExcL23	repression
**Pak6**	ExcL23	repression
**Mertk**	ExcL23	repression
**Fkbp1a**	ExcL23	repression
**Epb41l1**	ExcL23	repression
**Dlgap4**	ExcL23	repression
**Ppp1r16b**	ExcL23	repression
**Sertm1**	ExcL23	repression
**Atp1a1**	ExcL23	repression
**Npnt**	ExcL23	repression
**Skil**	ExcL23	repression
**Tiparp**	ExcL23	repression
**Gm3764**	ExcL23	repression
**Tpm3**	ExcL23	repression

Development • Supplementary information

**Igsf3**	ExcL23	repression
**Tnc**	ExcL23	repression
**Map7d1**	ExcL23	repression
**Efhd2**	ExcL23	repression
**Disp3**	ExcL23	repression
**Tgfbr1**	ExcL23	repression
**Nr4a3**	ExcL23	repression
**Nrd1**	ExcL23	repression
**Rims3**	ExcL23	repression
**Hpcal4**	ExcL23	repression
**Epha10**	ExcL23	repression
**Stk40**	ExcL23	repression
**Plekhg5**	ExcL23	repression
**Dvl1**	ExcL23	repression
**Rheb**	ExcL23	repression
**Wfs1**	ExcL23	repression
**Arap2**	ExcL23	repression
**Prkg2**	ExcL23	repression
**Sgsm1**	ExcL23	repression
**Cabp1**	ExcL23	repression
**Rph3a**	ExcL23	repression
**Pitpnm2**	ExcL23	repression
**Ncor2**	ExcL23	repression
**Pdgfa**	ExcL23	repression
**Prkar1b**	ExcL23	repression
**Fosl2**	ExcL23	repression
**Por**	ExcL23	repression
**Nptx2**	ExcL23	repression
**Dlx6os1**	ExcL23	repression
**Kdm7a**	ExcL23	repression
**Osbpl3**	ExcL23	repression
**Tet3**	ExcL23	repression
**Clstn3**	ExcL23	repression
**Dennd5b**	ExcL23	repression
**Gm3294**	ExcL23	repression
**Il17ra**	ExcL23	repression
**Mlf2**	ExcL23	repression
**Fosb**	ExcL23	repression
**Erf**	ExcL23	repression

Development • Supplementary information

**Ppme1**	ExcL23	repression
**Stx1b**	ExcL23	repression
**Shank1**	ExcL23	repression
**Acan**	ExcL23	repression
**Mir9-3hg**	ExcL23	repression
**Pak1**	ExcL23	repression
**Gdpd5**	ExcL23	repression
**Rab6a**	ExcL23	repression
**Sbk1**	ExcL23	repression
**Tacc1**	ExcL23	repression
**Maf**	ExcL23	repression
**Arhgef7**	ExcL23	repression
**Gm45345**	ExcL23	repression
**Naf1**	ExcL23	repression
**Slc9a5**	ExcL23	repression
**6430548M08Rik**	ExcL23	repression
**Tsnax**	ExcL23	repression
**Kmt2a**	ExcL23	repression
**Lingo1**	ExcL23	repression
**Scg3**	ExcL23	repression
**Igsf9b**	ExcL23	repression
**Rab6b**	ExcL23	repression
**Prkar2a**	ExcL23	repression
**Trak1**	ExcL23	repression
**Bcor**	ExcL23	repression
**AC149090.1**	ExcL4	induction
**Ogfrl1**	ExcL4	induction
**Ptprn**	ExcL4	induction
**Scg2**	ExcL4	induction
**Per2**	ExcL4	induction
**Lypd1**	ExcL4	induction
**Btg2**	ExcL4	induction
**Adora1**	ExcL4	induction
**Rgs4**	ExcL4	induction
**Atp1a2**	ExcL4	induction
**Coq10b**	ExcL4	induction
**Zdbf2**	ExcL4	induction
**Slc4a3**	ExcL4	induction
**Cntnap5a**	ExcL4	induction

Development • Supplementary information

**Ivns1abp**	ExcL4	induction
**Eprs**	ExcL4	induction
**Ddx50**	ExcL4	induction
**Cbarp**	ExcL4	induction
**Nfic**	ExcL4	induction
**Slc25a3**	ExcL4	induction
**Nudt4**	ExcL4	induction
**P4ha1**	ExcL4	induction
**Midn**	ExcL4	induction
**Dusp6**	ExcL4	induction
**Ube2b**	ExcL4	induction
**Hspa4**	ExcL4	induction
**Kdm6b**	ExcL4	induction
**Dusp14**	ExcL4	induction
**Cwc25**	ExcL4	induction
**Hdac5**	ExcL4	induction
**Ubtf**	ExcL4	induction
**BC006965**	ExcL4	induction
**Adcy1**	ExcL4	induction
**Ppp2ca**	ExcL4	induction
**Per1**	ExcL4	induction
**Camkk1**	ExcL4	induction
**Baiap2**	ExcL4	induction
**Eif5**	ExcL4	induction
**Inf2**	ExcL4	induction
**Pfkp**	ExcL4	induction
**Nrn1**	ExcL4	induction
**Ube2ql1**	ExcL4	induction
**Nr2f1**	ExcL4	induction
**Hmgcr**	ExcL4	induction
**BC005537**	ExcL4	induction
**Rreb1**	ExcL4	induction
**Brd9**	ExcL4	induction
**Pcsk1**	ExcL4	induction
**Hmgcs1**	ExcL4	induction
**Fzd3**	ExcL4	induction
**Tpt1**	ExcL4	induction
**Ndfip2**	ExcL4	induction
**Dnajc21**	ExcL4	induction

Development • Supplementary information

**Basp1**	ExcL4	induction
**Nptxr**	ExcL4	induction
**Pou6f1**	ExcL4	induction
**Csdc2**	ExcL4	induction
**Ccdc134**	ExcL4	induction
**Grasp**	ExcL4	induction
**Nr4a1**	ExcL4	induction
**Plcxd2**	ExcL4	induction
**Aifm3**	ExcL4	induction
**Gm15638**	ExcL4	induction
**Zbtb11**	ExcL4	induction
**Syngr3**	ExcL4	induction
**Rhot2**	ExcL4	induction
**Sik1**	ExcL4	induction
**Cdc42ep3**	ExcL4	induction
**Zfp948**	ExcL4	induction
**Gabbr1**	ExcL4	induction
**Gm26917**	ExcL4	induction
**Slc6a7**	ExcL4	induction
**Matr3**	ExcL4	induction
**Npas4**	ExcL4	induction
**Sptbn2**	ExcL4	induction
**Syt7**	ExcL4	induction
**Klf9**	ExcL4	induction
**Ina**	ExcL4	induction
**Slc25a25**	ExcL4	induction
**Scn7a**	ExcL4	induction
**Ctnnd1**	ExcL4	induction
**Cry2**	ExcL4	induction
**Adam33**	ExcL4	induction
**Bcas1**	ExcL4	induction
**Pmepa1**	ExcL4	induction
**Arl5b**	ExcL4	induction
**Kcnt1**	ExcL4	induction
**Tnfaip6**	ExcL4	induction
**7-Mar**	ExcL4	induction
**Syt13**	ExcL4	induction
**Bdnf**	ExcL4	induction
**Slc20a1**	ExcL4	induction

Development • Supplementary information

**Chgb**	ExcL4	induction
**Dlgap4**	ExcL4	induction
**Snhg11**	ExcL4	induction
**Top1**	ExcL4	induction
**Ccnl1**	ExcL4	induction
**Rxfp1**	ExcL4	induction
**Lmna**	ExcL4	induction
**Pip5k1a**	ExcL4	induction
**Atp1a1**	ExcL4	induction
**Gnai3**	ExcL4	induction
**Ntng1**	ExcL4	induction
**Npnt**	ExcL4	induction
**Ccn1**	ExcL4	induction
**Skil**	ExcL4	induction
**Tiparp**	ExcL4	induction
**Map9**	ExcL4	induction
**Mef2d**	ExcL4	induction
**Tpm3**	ExcL4	induction
**Nfib**	ExcL4	induction
**Smap2**	ExcL4	induction
**Efhd2**	ExcL4	induction
**Agrn**	ExcL4	induction
**Cnr1**	ExcL4	induction
**Dnajb5**	ExcL4	induction
**Nr4a3**	ExcL4	induction
**Ak4**	ExcL4	induction
**Nrd1**	ExcL4	induction
**Rims3**	ExcL4	induction
**Epha10**	ExcL4	induction
**Stk40**	ExcL4	induction
**Rcc2**	ExcL4	induction
**Plekhg5**	ExcL4	induction
**Chd5**	ExcL4	induction
**Sparcl1**	ExcL4	induction
**Miat**	ExcL4	induction
**Cabp1**	ExcL4	induction
**Hsph1**	ExcL4	induction
**Fosl2**	ExcL4	induction
**Nptx2**	ExcL4	induction

Development • Supplementary information

**Dlx6os1**	ExcL4	induction
**Thsd7a**	ExcL4	induction
**Kdm7a**	ExcL4	induction
**Nap1l5**	ExcL4	induction
**Tet3**	ExcL4	induction
**Mest**	ExcL4	induction
**Peg3**	ExcL4	induction
**Fosb**	ExcL4	induction
**Erf**	ExcL4	induction
**Stx1b**	ExcL4	induction
**Prkcg**	ExcL4	induction
**Mir9-3hg**	ExcL4	induction
**Adgra1**	ExcL4	induction
**Irs2**	ExcL4	induction
**Tacc1**	ExcL4	induction
**Smarca5**	ExcL4	induction
**Junb**	ExcL4	induction
**Ccsap**	ExcL4	induction
**Gpt2**	ExcL4	induction
**Cx3cl1**	ExcL4	induction
**Cpne7**	ExcL4	induction
**Dpy19l1**	ExcL4	induction
**Robo3**	ExcL4	induction
**Nrgn**	ExcL4	induction
**Pde4a**	ExcL4	induction
**Igsf9b**	ExcL4	induction
**Hspa8**	ExcL4	induction
**Rab6b**	ExcL4	induction
**Tenm1**	ExcL4	induction
**Gria3**	ExcL4	induction
**Cd34**	ExcL4	repression
**Alcam**	ExcL4	repression
**Dcbld2**	ExcL4	repression
**Gria2**	ExcL4	repression
**Pcdh10**	ExcL4	repression
**Ddit4l**	ExcL4	repression
**Calb1**	ExcL4	repression
**Slc4a4**	ExcL4	repression
**AC149090.1**	ExcL4	induction

**Ptprn**	ExcL4	induction
**Ptgs2**	ExcL4	induction
**Dot1l**	ExcL4	induction
**Gadd45b**	ExcL4	induction
**Dusp6**	ExcL4	induction
**Kdm6b**	ExcL4	induction
**Camkk1**	ExcL4	induction
**Rock2**	ExcL4	induction
**Map1b**	ExcL4	induction
**Homer1**	ExcL4	induction
**Dgkh**	ExcL4	induction
**Cacng2**	ExcL4	induction
**Pdgfb**	ExcL4	induction
**Arhgap31**	ExcL4	induction
**Gm26917**	ExcL4	induction
**Slc6a7**	ExcL4	induction
**Csnk1a1**	ExcL4	induction
**Cbln2**	ExcL4	induction
**Npas4**	ExcL4	induction
**Bdnf**	ExcL4	induction
**Spred1**	ExcL4	induction
**Npnt**	ExcL4	induction
**Ptprf**	ExcL4	induction
**Raver2**	ExcL4	induction
**Rims3**	ExcL4	induction
**Hpcal4**	ExcL4	induction
**Sgsm1**	ExcL4	induction
**Fosl2**	ExcL4	induction
**Dlx6os1**	ExcL4	induction
**Thsd7a**	ExcL4	induction
**Cped1**	ExcL4	induction
**Peg3**	ExcL4	induction
**Fosb**	ExcL4	induction
**Prkcg**	ExcL4	induction
**Shank1**	ExcL4	induction
**Gdpd5**	ExcL4	induction
**Ptpre**	ExcL4	induction
**Adgra1**	ExcL4	induction
**Cpne7**	ExcL4	induction

Development • Supplementary information

**Robo3**	ExcL4	induction
**Gramd1b**	ExcL4	induction
**Pde4a**	ExcL4	induction
**Igsf9b**	ExcL4	induction
**Gria3**	ExcL4	induction
**Scg2**	ExcL4	repression
**Btg2**	ExcL4	repression
**Rgs4**	ExcL4	repression
**Atp1a2**	ExcL4	repression
**Cntnap5a**	ExcL4	repression
**Tmem199**	ExcL4	repression
**Eif1**	ExcL4	repression
**Etv1**	ExcL4	repression
**Fos**	ExcL4	repression
**Alcam**	ExcL4	repression
**Malat1**	ExcL4	repression
**Cox8a**	ExcL4	repression
**Fth1**	ExcL4	repression
**Ptgds**	ExcL4	repression
**Adam33**	ExcL4	repression
**Cst3**	ExcL4	repression
**Gad2**	ExcL4	repression
**Rps21**	ExcL4	repression
**Ntng1**	ExcL4	repression
**Ccn1**	ExcL4	repression
**Calb1**	ExcL4	repression
**Hpgds**	ExcL4	repression
**Mrpl53**	ExcL4	repression
**Junb**	ExcL4	repression
**Nrgn**	ExcL4	repression
**AI593442**	ExcL4	repression
**Hspa8**	ExcL4	repression
**Camkv**	ExcL4	repression
**Plp1**	ExcL4	repression
**AC149090.1**	ExcL4	induction
**Scg2**	ExcL4	induction
**Atp1a2**	ExcL4	induction
**Cntnap5a**	ExcL4	induction
**Ptgs2**	ExcL4	induction

Development • Supplementary information

**H3f3b**	ExcL4	induction
**Eif1**	ExcL4	induction
**Sox9**	ExcL4	induction
**Snhg20**	ExcL4	induction
**Inf2**	ExcL4	induction
**Nr2f1**	ExcL4	induction
**Dgkh**	ExcL4	induction
**Lrtm1**	ExcL4	induction
**Pabpn1**	ExcL4	induction
**Tuba1a**	ExcL4	induction
**Alcam**	ExcL4	induction
**Htr1f**	ExcL4	induction
**Dact2**	ExcL4	induction
**Gm26917**	ExcL4	induction
**Malat1**	ExcL4	induction
**Cox8a**	ExcL4	induction
**B3gat3**	ExcL4	induction
**Fth1**	ExcL4	induction
**Syt7**	ExcL4	induction
**C1ql3**	ExcL4	induction
**Adam33**	ExcL4	induction
**Cst3**	ExcL4	induction
**Dlgap4**	ExcL4	induction
**Rps21**	ExcL4	induction
**Ntng1**	ExcL4	induction
**Npnt**	ExcL4	induction
**Ddit4l**	ExcL4	induction
**Frrs1l**	ExcL4	induction
**Ptprf**	ExcL4	induction
**Plekhg5**	ExcL4	induction
**Ociad2**	ExcL4	induction
**Prkg2**	ExcL4	induction
**Nptx2**	ExcL4	induction
**Dlx6os1**	ExcL4	induction
**Ndufa4**	ExcL4	induction
**C1galt1**	ExcL4	induction
**Gm15594**	ExcL4	induction
**Hras**	ExcL4	induction
**Prkcg**	ExcL4	induction

**Gdpd5**	ExcL4	induction
**Slc25a4**	ExcL4	induction
**Junb**	ExcL4	induction
**Cpne7**	ExcL4	induction
**Nrgn**	ExcL4	induction
**AI593442**	ExcL4	induction
**Slc6a20a**	ExcL4	induction
**Igsf9b**	ExcL4	induction
**Gm15520**	ExcL4	induction
**Plp1**	ExcL4	induction
**Btg2**	ExcL4	repression
**Adora1**	ExcL4	repression
**Rgs4**	ExcL4	repression
**Mia3**	ExcL4	repression
**Coq10b**	ExcL4	repression
**Zdbf2**	ExcL4	repression
**Ivns1abp**	ExcL4	repression
**Cbarp**	ExcL4	repression
**Slc25a3**	ExcL4	repression
**Nudt4**	ExcL4	repression
**Arhgap45**	ExcL4	repression
**Pip5k1c**	ExcL4	repression
**Ube2b**	ExcL4	repression
**Kdm6b**	ExcL4	repression
**Dusp14**	ExcL4	repression
**Srcin1**	ExcL4	repression
**Hdac5**	ExcL4	repression
**BC006965**	ExcL4	repression
**Ube2o**	ExcL4	repression
**Cpeb4**	ExcL4	repression
**Ppp2ca**	ExcL4	repression
**Dlg4**	ExcL4	repression
**Baiap2**	ExcL4	repression
**Clmn**	ExcL4	repression
**Ckb**	ExcL4	repression
**Fos**	ExcL4	repression
**Pfkp**	ExcL4	repression
**Ube2ql1**	ExcL4	repression
**Pcsk1**	ExcL4	repression

Development • Supplementary information

**Nr1d2**	ExcL4	repression
**Jph4**	ExcL4	repression
**Fzd3**	ExcL4	repression
**Nefl**	ExcL4	repression
**Pou6f1**	ExcL4	repression
**Plcxd2**	ExcL4	repression
**Gm15638**	ExcL4	repression
**Cdc42ep3**	ExcL4	repression
**Syt4**	ExcL4	repression
**Slc6a7**	ExcL4	repression
**Csnk1a1**	ExcL4	repression
**Npas4**	ExcL4	repression
**Sptbn2**	ExcL4	repression
**Klf9**	ExcL4	repression
**Slc25a25**	ExcL4	repression
**Scn7a**	ExcL4	repression
**Ctnnd1**	ExcL4	repression
**Cry2**	ExcL4	repression
**Cbln4**	ExcL4	repression
**Pmepa1**	ExcL4	repression
**Arl5b**	ExcL4	repression
**Tnfaip6**	ExcL4	repression
**Syt13**	ExcL4	repression
**Bdnf**	ExcL4	repression
**Mertk**	ExcL4	repression
**Atp1a1**	ExcL4	repression
**Ccn1**	ExcL4	repression
**Skil**	ExcL4	repression
**Tiparp**	ExcL4	repression
**Mef2d**	ExcL4	repression
**Kcnn3**	ExcL4	repression
**Tpm3**	ExcL4	repression
**Nfib**	ExcL4	repression
**Nr4a3**	ExcL4	repression
**Ak4**	ExcL4	repression
**Nrd1**	ExcL4	repression
**Stk40**	ExcL4	repression
**Rcc2**	ExcL4	repression
**Cabp1**	ExcL4	repression

**Slc7a1**	ExcL4	repression
**Fosl2**	ExcL4	repression
**Thsd7a**	ExcL4	repression
**Kdm7a**	ExcL4	repression
**Tet3**	ExcL4	repression
**Mbd4**	ExcL4	repression
**Ybx3**	ExcL4	repression
**Peg3**	ExcL4	repression
**Fosb**	ExcL4	repression
**Pak1**	ExcL4	repression
**Tacc1**	ExcL4	repression
**Adgrl1**	ExcL4	repression
**Gpt2**	ExcL4	repression
**Robo3**	ExcL4	repression
**Plcd1**	ExcL4	repression
**Tenm1**	ExcL4	repression
**Gria3**	ExcL4	repression
**Ddx3y**	ExcL4	repression
**AC149090.1**	ExcL4	induction
**Btg2**	ExcL4	induction
**Rgs4**	ExcL4	induction
**Atp1a2**	ExcL4	induction
**Cntnap5a**	ExcL4	induction
**Cd34**	ExcL4	induction
**Ebf1**	ExcL4	induction
**Etv1**	ExcL4	induction
**Klf10**	ExcL4	induction
**Alcam**	ExcL4	induction
**Malat1**	ExcL4	induction
**Cox8a**	ExcL4	induction
**Rorb**	ExcL4	induction
**Fth1**	ExcL4	induction
**C1ql3**	ExcL4	induction
**Ptgds**	ExcL4	induction
**Ano3**	ExcL4	induction
**Adam33**	ExcL4	induction
**Gad1**	ExcL4	induction
**Mrps26**	ExcL4	induction
**Snhg11**	ExcL4	induction

Development • Supplementary information

**Ntng1**	ExcL4	induction
**Nfib**	ExcL4	induction
**Calb1**	ExcL4	induction
**Thsd7a**	ExcL4	induction
**Ptpre**	ExcL4	induction
**Junb**	ExcL4	induction
**Camkv**	ExcL4	induction
**Cspg5**	ExcL4	induction
**Plp1**	ExcL4	induction
**Ptprn**	ExcL4	repression
**Per2**	ExcL4	repression
**Adora1**	ExcL4	repression
**Midn**	ExcL4	repression
**Dot1l**	ExcL4	repression
**Pip5k1c**	ExcL4	repression
**Gm16105**	ExcL4	repression
**Nap1l1**	ExcL4	repression
**R3hdm2**	ExcL4	repression
**Kdm6b**	ExcL4	repression
**Pitpnm3**	ExcL4	repression
**Neurod2**	ExcL4	repression
**Camkk1**	ExcL4	repression
**Baz1a**	ExcL4	repression
**Actn1**	ExcL4	repression
**Eml5**	ExcL4	repression
**Rock2**	ExcL4	repression
**Frmd6**	ExcL4	repression
**Zbtb1**	ExcL4	repression
**Fos**	ExcL4	repression
**Inf2**	ExcL4	repression
**Gm32036**	ExcL4	repression
**Map1b**	ExcL4	repression
**Pcsk1**	ExcL4	repression
**Enc1**	ExcL4	repression
**Fzd3**	ExcL4	repression
**Dgkh**	ExcL4	repression
**Zmiz1**	ExcL4	repression
**Pdgfb**	ExcL4	repression
**A730060N03Rik**	ExcL4	repression

Development • Supplementary information

**Arhgap31**	ExcL4	repression
**Qk**	ExcL4	repression
**Slc6a7**	ExcL4	repression
**Rnf165**	ExcL4	repression
**Csnk1a1**	ExcL4	repression
**Npas4**	ExcL4	repression
**Tmem132a**	ExcL4	repression
**Sptbn2**	ExcL4	repression
**Hectd2**	ExcL4	repression
**Rnd3**	ExcL4	repression
**Mir670hg**	ExcL4	repression
**Bcas1**	ExcL4	repression
**Gad2**	ExcL4	repression
**Col5a1**	ExcL4	repression
**Syt13**	ExcL4	repression
**Slc1a2**	ExcL4	repression
**Bdnf**	ExcL4	repression
**Spred1**	ExcL4	repression
**Pak6**	ExcL4	repression
**Dlgap4**	ExcL4	repression
**Rxfp1**	ExcL4	repression
**Npnt**	ExcL4	repression
**Tiparp**	ExcL4	repression
**Igsf3**	ExcL4	repression
**Efhd2**	ExcL4	repression
**Disp3**	ExcL4	repression
**Dnajb5**	ExcL4	repression
**Tgfbr1**	ExcL4	repression
**Nr4a3**	ExcL4	repression
**Raver2**	ExcL4	repression
**Nrd1**	ExcL4	repression
**Rims3**	ExcL4	repression
**Hpcal4**	ExcL4	repression
**Epha10**	ExcL4	repression
**Adgrb2**	ExcL4	repression
**Plekhg5**	ExcL4	repression
**Prkg2**	ExcL4	repression
**Sgsm1**	ExcL4	repression
**Pitpnm2**	ExcL4	repression

Development • Supplementary information

**Ncor2**	ExcL4	repression
**Fosl2**	ExcL4	repression
**Galnt9**	ExcL4	repression
**Nptx2**	ExcL4	repression
**Tet3**	ExcL4	repression
**Iqsec3**	ExcL4	repression
**Clstn3**	ExcL4	repression
**Peg3**	ExcL4	repression
**Ppp1r37**	ExcL4	repression
**Prkcg**	ExcL4	repression
**Shank1**	ExcL4	repression
**Akap13**	ExcL4	repression
**Mir9-3hg**	ExcL4	repression
**Gdpd5**	ExcL4	repression
**Adgra1**	ExcL4	repression
**Galnt7**	ExcL4	repression
**Cers4**	ExcL4	repression
**Slc9a5**	ExcL4	repression
**Cpne7**	ExcL4	repression
**Nrp1**	ExcL4	repression
**Robo3**	ExcL4	repression
**Gramd1b**	ExcL4	repression
**Kmt2a**	ExcL4	repression
**Lingo1**	ExcL4	repression
**Igsf9b**	ExcL4	repression
**Nectin1**	ExcL4	repression
**Vipr1**	ExcL4	repression
**Gria3**	ExcL4	repression

Table S5. Signaling modules identified by CellChat.

Ligand-receptor pairs
WNT5B_FZD3
TGFA_EGFR
NRG1_ERBB4
NRG2_ERBB4
NRG3_ERBB4
FGF1_FGFR1
FGF1_FGFR2
FGF1_FGFR3
FGF9_FGFR1
FGF9_FGFR2
FGF9_FGFR3
PDGFC_PDGFRA
IGF1_IGF1R
CX3CL1_CX3CR1
IL34_CSF1R
PTN_PTPRZ1
PTN_SDC2
PTN_NCL
PTN_ALK
CCK_CCKBR
VIP_VIPR1
KITL_KIT
BDNF_NTRK2
SEMA3A_NRP1_PLXNA2
SEMA3A_NRP1_PLXNA4
SEMA3C_NRP1_PLXNA2
SEMA3C_NRP1_PLXNA4
SEMA3D_NRP1_PLXNA2
SEMA3D_NRP1_PLXNA4
SEMA3C_NRP2_PLXNA2
SEMA3C_NRP2_PLXNA4
SEMA3D_NRP2_PLXNA2
SEMA3D_NRP2_PLXNA4
GAS6_MERTK
GAS6_TYRO3

Development • Supplementary information

PROS1_TYRO3
PSAP_GPR37L1
PTPRS_NTRK3
SLITRK1_PTPRD
SLITRK1_PTPRS
SLITRK5_PTPRS
BDNF_SORT1
SLIT1_ROBO1
SLIT2_ROBO1
SLIT2_ROBO2
TUB_MERTK
PROS1_MERTK
TNR_ITGA8_ITGB1
TNR_ITGA9_ITGB1
COL4A1_ITGA9_ITGB1
COL4A2_ITGA9_ITGB1
COL6A1_ITGA9_ITGB1
LAMA2_ITGA9_ITGB1
LAMA3_ITGA9_ITGB1
LAMA4_ITGA9_ITGB1
LAMC1_ITGA9_ITGB1
LAMA2_SV2A
LAMA3_SV2A
LAMA4_SV2A
LAMC1_SV2A
LAMA2_SV2B
LAMA3_SV2B
LAMA4_SV2B
LAMC1_SV2B
LAMA2_SV2C
LAMA3_SV2C
LAMA4_SV2C
LAMC1_SV2C
AGRN_DAG1
LAMA2_DAG1
LAMA3_DAG1
LAMA4_DAG1
LAMC1_DAG1
RELN_LRP8

RELN_VLDLR
GABA-A-GAD1_SLC6A1_GABR_A1B3G2
GABA-A-GAD1_SLC6A1_GABR_A1B2G2
GABA-A-GAD1_SLC6A1_GABR_A1B2
GABA-A-GAD1_SLC6A1_GABR_A1B3
GABA-A-GAD1_SLC6A1_GABR_A1B2D
GABA-A-GAD1_SLC6A1_GABR_A2B3G2
GABA-A-GAD1_SLC6A1_GABR_A3B3G2
GABA-A-GAD1_SLC6A1_GABR_A4B3G2
GABA-A-GAD1_SLC6A1_GABR_A4B3D
GABA-A-GAD1_SLC6A1_GABR_A4B2D
GABA-A-GAD1_SLC6A1_GABR_A5B3G2
GABA-A-GAD1_SLC6A6_GABR_A1B3G2
GABA-A-GAD1_SLC6A6_GABR_A1B2G2
GABA-A-GAD1_SLC6A6_GABR_A1B2
GABA-A-GAD1_SLC6A6_GABR_A1B3
GABA-A-GAD1_SLC6A6_GABR_A1B2D
GABA-A-GAD1_SLC6A6_GABR_A2B3G2
GABA-A-GAD1_SLC6A6_GABR_A3B3G2
GABA-A-GAD1_SLC6A6_GABR_A4B3G2
GABA-A-GAD1_SLC6A6_GABR_A4B3D
GABA-A-GAD1_SLC6A6_GABR_A4B2D
GABA-A-GAD1_SLC6A6_GABR_A5B3G2
GABA-A-GAD2_SLC6A1_GABR_A1B3G2
GABA-A-GAD2_SLC6A1_GABR_A1B2G2
GABA-A-GAD2_SLC6A1_GABR_A1B2
GABA-A-GAD2_SLC6A1_GABR_A1B3
GABA-A-GAD2_SLC6A1_GABR_A1B2D
GABA-A-GAD2_SLC6A1_GABR_A2B3G2
GABA-A-GAD2_SLC6A1_GABR_A3B3G2
GABA-A-GAD2_SLC6A1_GABR_A4B3G2
GABA-A-GAD2_SLC6A1_GABR_A4B3D
GABA-A-GAD2_SLC6A1_GABR_A4B2D
GABA-A-GAD2_SLC6A1_GABR_A5B3G2
GABA-A-GAD2_SLC6A6_GABR_A1B3G2
GABA-A-GAD2_SLC6A6_GABR_A1B2G2
GABA-A-GAD2_SLC6A6_GABR_A1B2
GABA-A-GAD2_SLC6A6_GABR_A1B3
GABA-A-GAD2_SLC6A6_GABR_A1B2D

Development • Supplementary information

GABA-A-GAD2_SLC6A6_GABR_A2B3G2
GABA-A-GAD2_SLC6A6_GABR_A3B3G2
GABA-A-GAD2_SLC6A6_GABR_A4B3G2
GABA-A-GAD2_SLC6A6_GABR_A4B3D
GABA-A-GAD2_SLC6A6_GABR_A4B2D
GABA-A-GAD2_SLC6A6_GABR_A5B3G2
GABA-B-GAD1_SLC6A1_GABBR1
GABA-B-GAD1_SLC6A6_GABBR1
GABA-B-GAD2_SLC6A1_GABBR1
GABA-B-GAD2_SLC6A6_GABBR1
GABA-B-GAD1_SLC6A1_GABBR2
GABA-B-GAD1_SLC6A6_GABBR2
GABA-B-GAD2_SLC6A1_GABBR2
GABA-B-GAD2_SLC6A6_GABBR2
Glutamate-Glu-SLC17A7_GLS_GRIA1
Glutamate-Glu-SLC17A8_GLS_GRIA1
Glutamate-Glu-SLC1A1_GLS_GRIA1
Glutamate-Glu-SLC1A2_GLS_GRIA1
Glutamate-Glu-SLC1A3_GLS_GRIA1
Glutamate-Glu-SLC17A7_GLS2_GRIA1
Glutamate-Glu-SLC1A1_GLS2_GRIA1
Glutamate-Glu-SLC1A2_GLS2_GRIA1
Glutamate-Glu-SLC17A7_GLS_GRIA2
Glutamate-Glu-SLC17A8_GLS_GRIA2
Glutamate-Glu-SLC1A1_GLS_GRIA2
Glutamate-Glu-SLC1A2_GLS_GRIA2
Glutamate-Glu-SLC1A3_GLS_GRIA2
Glutamate-Glu-SLC17A7_GLS2_GRIA2
Glutamate-Glu-SLC1A1_GLS2_GRIA2
Glutamate-Glu-SLC1A2_GLS2_GRIA2
Glutamate-Glu-SLC17A7_GLS_GRIA3
Glutamate-Glu-SLC17A8_GLS_GRIA3
Glutamate-Glu-SLC1A1_GLS_GRIA3
Glutamate-Glu-SLC1A2_GLS_GRIA3
Glutamate-Glu-SLC1A3_GLS_GRIA3
Glutamate-Glu-SLC17A7_GLS2_GRIA3
Glutamate-Glu-SLC1A1_GLS2_GRIA3
Glutamate-Glu-SLC1A2_GLS2_GRIA3
Glutamate-Glu-SLC17A7_GLS_GRIA4

Development • Supplementary information

Glutamate-Glu-SLC17A8_GLS_GRIA4
Glutamate-Glu-SLC1A1_GLS_GRIA4
Glutamate-Glu-SLC1A2_GLS_GRIA4
Glutamate-Glu-SLC1A3_GLS_GRIA4
Glutamate-Glu-SLC17A7_GLS2_GRIA4
Glutamate-Glu-SLC1A1_GLS2_GRIA4
Glutamate-Glu-SLC1A2_GLS2_GRIA4
Glutamate-Glu-SLC17A7_GLS_GRIK1
Glutamate-Glu-SLC17A8_GLS_GRIK1
Glutamate-Glu-SLC1A1_GLS_GRIK1
Glutamate-Glu-SLC1A2_GLS_GRIK1
Glutamate-Glu-SLC1A3_GLS_GRIK1
Glutamate-Glu-SLC17A7_GLS2_GRIK1
Glutamate-Glu-SLC1A1_GLS2_GRIK1
Glutamate-Glu-SLC1A2_GLS2_GRIK1
Glutamate-Glu-SLC17A7_GLS_GRIK2
Glutamate-Glu-SLC17A8_GLS_GRIK2
Glutamate-Glu-SLC1A1_GLS_GRIK2
Glutamate-Glu-SLC1A2_GLS_GRIK2
Glutamate-Glu-SLC1A3_GLS_GRIK2
Glutamate-Glu-SLC17A7_GLS2_GRIK2
Glutamate-Glu-SLC1A1_GLS2_GRIK2
Glutamate-Glu-SLC1A2_GLS2_GRIK2
Glutamate-Glu-SLC17A7_GLS_GRIK3
Glutamate-Glu-SLC17A8_GLS_GRIK3
Glutamate-Glu-SLC1A1_GLS_GRIK3
Glutamate-Glu-SLC1A2_GLS_GRIK3
Glutamate-Glu-SLC1A3_GLS_GRIK3
Glutamate-Glu-SLC17A7_GLS2_GRIK3
Glutamate-Glu-SLC1A1_GLS2_GRIK3
Glutamate-Glu-SLC1A2_GLS2_GRIK3
Glutamate-Glu-SLC17A7_GLS_GRM1
Glutamate-Glu-SLC17A8_GLS_GRM1
Glutamate-Glu-SLC1A1_GLS_GRM1
Glutamate-Glu-SLC1A2_GLS_GRM1
Glutamate-Glu-SLC1A3_GLS_GRM1
Glutamate-Glu-SLC17A7_GLS2_GRM1
Glutamate-Glu-SLC1A1_GLS2_GRM1
Glutamate-Glu-SLC1A2_GLS2_GRM1

Development • Supplementary information

Glutamate-Glu-SLC17A7_GLS_GRM3
Glutamate-Glu-SLC17A8_GLS_GRM3
Glutamate-Glu-SLC1A1_GLS_GRM3
Glutamate-Glu-SLC1A2_GLS_GRM3
Glutamate-Glu-SLC1A3_GLS_GRM3
Glutamate-Glu-SLC17A7_GLS2_GRM3
Glutamate-Glu-SLC1A1_GLS2_GRM3
Glutamate-Glu-SLC1A2_GLS2_GRM3
Glutamate-Glu-SLC17A7_GLS_GRM5
Glutamate-Glu-SLC17A8_GLS_GRM5
Glutamate-Glu-SLC1A1_GLS_GRM5
Glutamate-Glu-SLC1A2_GLS_GRM5
Glutamate-Glu-SLC1A3_GLS_GRM5
Glutamate-Glu-SLC17A7_GLS2_GRM5
Glutamate-Glu-SLC1A1_GLS2_GRM5
Glutamate-Glu-SLC1A2_GLS2_GRM5
Glutamate-Glu-SLC17A7_GLS_GRM7
Glutamate-Glu-SLC17A8_GLS_GRM7
Glutamate-Glu-SLC1A1_GLS_GRM7
Glutamate-Glu-SLC1A2_GLS_GRM7
Glutamate-Glu-SLC1A3_GLS_GRM7
Glutamate-Glu-SLC17A7_GLS2_GRM7
Glutamate-Glu-SLC1A1_GLS2_GRM7
Glutamate-Glu-SLC1A2_GLS2_GRM7
Glutamate-Glu-SLC17A7_GLS_GRM8
Glutamate-Glu-SLC17A8_GLS_GRM8
Glutamate-Glu-SLC1A1_GLS_GRM8
Glutamate-Glu-SLC1A2_GLS_GRM8
Glutamate-Glu-SLC1A3_GLS_GRM8
Glutamate-Glu-SLC17A7_GLS2_GRM8
Glutamate-Glu-SLC1A1_GLS2_GRM8
Glutamate-Glu-SLC1A2_GLS2_GRM8
Glutamate-Glu-SLC17A7_GLS_GRIK1_GRIK4
Glutamate-Glu-SLC17A7_GLS_GRIK2_GRIK4
Glutamate-Glu-SLC17A7_GLS_GRIK3_GRIK4
Glutamate-Glu-SLC17A7_GLS_GRIK1_GRIK5
Glutamate-Glu-SLC17A7_GLS_GRIK2_GRIK5
Glutamate-Glu-SLC17A7_GLS_GRIK3_GRIK5
Glutamate-Glu-SLC17A7_GLS_GRIN1_GRIN2A

Development • Supplementary information

Glutamate-Glu-SLC17A7_GLS_GRIN1_GRIN2B
Glutamate-Glu-SLC17A7_GLS_GRIN1_GRIN2D
Glutamate-Glu-SLC17A8_GLS_GRIK1_GRIK4
Glutamate-Glu-SLC17A8_GLS_GRIK2_GRIK4
Glutamate-Glu-SLC17A8_GLS_GRIK3_GRIK4
Glutamate-Glu-SLC17A8_GLS_GRIK1_GRIK5
Glutamate-Glu-SLC17A8_GLS_GRIK2_GRIK5
Glutamate-Glu-SLC17A8_GLS_GRIK3_GRIK5
Glutamate-Glu-SLC17A8_GLS_GRIN1_GRIN2A
Glutamate-Glu-SLC17A8_GLS_GRIN1_GRIN2B
Glutamate-Glu-SLC17A8_GLS_GRIN1_GRIN2D
Glutamate-Glu-SLC1A1_GLS_GRIK1_GRIK4
Glutamate-Glu-SLC1A1_GLS_GRIK2_GRIK4
Glutamate-Glu-SLC1A1_GLS_GRIK3_GRIK4
Glutamate-Glu-SLC1A1_GLS_GRIK1_GRIK5
Glutamate-Glu-SLC1A1_GLS_GRIK2_GRIK5
Glutamate-Glu-SLC1A1_GLS_GRIK3_GRIK5
Glutamate-Glu-SLC1A1_GLS_GRIN1_GRIN2A
Glutamate-Glu-SLC1A1_GLS_GRIN1_GRIN2B
Glutamate-Glu-SLC1A1_GLS_GRIN1_GRIN2D
Glutamate-Glu-SLC1A2_GLS_GRIK1_GRIK4
Glutamate-Glu-SLC1A2_GLS_GRIK2_GRIK4
Glutamate-Glu-SLC1A2_GLS_GRIK3_GRIK4
Glutamate-Glu-SLC1A2_GLS_GRIK1_GRIK5
Glutamate-Glu-SLC1A2_GLS_GRIK2_GRIK5
Glutamate-Glu-SLC1A2_GLS_GRIK3_GRIK5
Glutamate-Glu-SLC1A2_GLS_GRIN1_GRIN2A
Glutamate-Glu-SLC1A2_GLS_GRIN1_GRIN2B
Glutamate-Glu-SLC1A2_GLS_GRIN1_GRIN2D
Glutamate-Glu-SLC1A3_GLS_GRIK1_GRIK4
Glutamate-Glu-SLC1A3_GLS_GRIK2_GRIK4
Glutamate-Glu-SLC1A3_GLS_GRIK3_GRIK4
Glutamate-Glu-SLC1A3_GLS_GRIK1_GRIK5
Glutamate-Glu-SLC1A3_GLS_GRIK2_GRIK5
Glutamate-Glu-SLC1A3_GLS_GRIK3_GRIK5
Glutamate-Glu-SLC1A3_GLS_GRIN1_GRIN2A
Glutamate-Glu-SLC1A3_GLS_GRIN1_GRIN2B
Glutamate-Glu-SLC1A3_GLS_GRIN1_GRIN2D
Glutamate-Glu-SLC17A7_GLS2_GRIK1_GRIK4

Development • Supplementary information

Glutamate-Glu-SLC17A7_GLS2_GRIK2_GRIK4
Glutamate-Glu-SLC17A7_GLS2_GRIK3_GRIK4
Glutamate-Glu-SLC17A7_GLS2_GRIK1_GRIK5
Glutamate-Glu-SLC17A7_GLS2_GRIK2_GRIK5
Glutamate-Glu-SLC17A7_GLS2_GRIK3_GRIK5
Glutamate-Glu-SLC17A7_GLS2_GRIN1_GRIN2A
Glutamate-Glu-SLC17A7_GLS2_GRIN1_GRIN2B
Glutamate-Glu-SLC17A7_GLS2_GRIN1_GRIN2D
Glutamate-Glu-SLC1A1_GLS2_GRIK1_GRIK4
Glutamate-Glu-SLC1A1_GLS2_GRIK2_GRIK4
Glutamate-Glu-SLC1A1_GLS2_GRIK3_GRIK4
Glutamate-Glu-SLC1A1_GLS2_GRIK1_GRIK5
Glutamate-Glu-SLC1A1_GLS2_GRIK2_GRIK5
Glutamate-Glu-SLC1A1_GLS2_GRIK3_GRIK5
Glutamate-Glu-SLC1A1_GLS2_GRIN1_GRIN2A
Glutamate-Glu-SLC1A1_GLS2_GRIN1_GRIN2B
Glutamate-Glu-SLC1A1_GLS2_GRIN1_GRIN2D
Glutamate-Glu-SLC1A2_GLS2_GRIK1_GRIK4
Glutamate-Glu-SLC1A2_GLS2_GRIK2_GRIK4
Glutamate-Glu-SLC1A2_GLS2_GRIK3_GRIK4
Glutamate-Glu-SLC1A2_GLS2_GRIK1_GRIK5
Glutamate-Glu-SLC1A2_GLS2_GRIK2_GRIK5
Glutamate-Glu-SLC1A2_GLS2_GRIK3_GRIK5
Glutamate-Glu-SLC1A2_GLS2_GRIN1_GRIN2A
Glutamate-Glu-SLC1A2_GLS2_GRIN1_GRIN2B
Glutamate-Glu-SLC1A2_GLS2_GRIN1_GRIN2D
2arachidonoylglycerol-2AG-DAGLA_CNR1
2arachidonoylglycerol-2AG-DAGLB_CNR1
Testosterone-Testosterone-HSD17B12_AR
CADM1_CADM1
CADM3_CADM3
ENTPD1_ADORA1
CDH4_CDH4
CDH2_CDH2
CLDN11_CLDN11
NFASC_CNTN1_CNTNAP1
CNTN2_CNTN2_CNTNAP2
CNTN2_CNTN2
CNTN1_NRCAM

Development • Supplementary information

EFNA5_EPHA3
EFNA5_EPHA4
EFNA5_EPHA5
EFNA5_EPHA7
EFNA5_EPHB2
EFNB2_EPHA4
EFNB2_EPHB1
EFNB2_EPHB2
EFNB3_EPHA4
EFNB3_EPHB1
EFNB3_EPHB2
EFNB3_EPHB6
JAM2_ITGAV_ITGB1
JAM2_JAM3
F11R_F11R
JAM2_F11R
JAM2_JAM2
JAM3_F11R
JAM3_JAM3
MAG_MAG
MPZL1_MPZL1
NCAM1_FGFR1
NCAM1_NCAM1
NCAM1_NCAM2
NEGR1_NEGR1
LRRC4C_NTNG1
LRRC4_NTNG2
LRRC4B_PTPRF
NRXN1_NLGN1
NRXN1_NLGN2
NRXN1_NLGN3
NRXN2_NLGN1
NRXN2_NLGN2
NRXN2_NLGN3
NRXN3_NLGN1
NRXN3_NLGN2
NRXN3_NLGN3
PTPRM_PTPRM
SEMA4D_PLXNB1

SEMA4D_PLXNB3
SEMA5A_PLXNB3
SEMA6A_PLXNA2
SEMA6A_PLXNA4
VSIR_IGSF11
GJA1_GJA1
RTN4R_ADGRB1
ITGAV_ITGB5_ADGRB1
C1QL1_ADGRB3
C1QL3_ADGRB3
COL4A1_ADGRG6
COL4A2_ADGRG6
LAMA2_ADGRG6
PRNP_ADGRG6
FLRT1_ADGRL1
FLRT2_ADGRL1
FLRT3_ADGRL1
NRXN1_ADGRL1
NRXN2_ADGRL1
NRXN3_ADGRL1
TENM1_ADGRL1
TENM2_ADGRL1
TENM3_ADGRL1
TENM4_ADGRL1
TENM2_FLRT1_ADGRL1
TENM2_FLRT3_ADGRL1
TENM4_FLRT1_ADGRL1
TENM4_FLRT3_ADGRL1
FLRT1_ADGRL2
FLRT2_ADGRL2
FLRT3_ADGRL2
TENM1_ADGRL2
TENM2_ADGRL2
TENM3_ADGRL2
TENM4_ADGRL2
TENM2_FLRT3_ADGRL2
FLRT1_ADGRL3
FLRT2_ADGRL3
FLRT3_ADGRL3

TENM1_ADGRL3
TENM2_ADGRL3
TENM3_ADGRL3
TENM4_ADGRL3
UNC5A_ADGRL3
TENM3_FLRT1_ADGRL3
TENM3_FLRT3_ADGRL3
FLRT1_FLRT1
FLRT2_FLRT2
FLRT3_FLRT3
FLRT1_UNC5A
FLRT2_UNC5A
FLRT3_UNC5A
FLRT1_UNC5C
FLRT2_UNC5C
FLRT3_UNC5C
FLRT1_UNC5D
FLRT2_UNC5D
FLRT3_UNC5D
NRXN1_CLSTN1
NRXN2_CLSTN1
NRXN1_CLSTN2
NRXN2_CLSTN2
NRXN1_CLSTN3
NRXN2_CLSTN3
NRXN1_DAG1
NRXN2_DAG1
LRFN5_PTPRD
LRFN5_PTPRF
LRFN5_PTPRS
LRRC4B_PTPRD
LRRC4B_PTPRS
LRRC4C_PTPRF
NRXN1_LRRTM1
NRXN2_LRRTM1
NRXN3_LRRTM1
NRXN1_LRRTM3
NRXN2_LRRTM3
NRXN3_LRRTM3

Development • Supplementary information

NRXN1_LRRTM4
NRXN2_LRRTM4
NRXN3_LRRTM4
LRRTM4_PTPRS
APP_SORL1
APP_TNFRSF21
CADM1_NECTIN3
CADM3_CADM1
CADM3_CADM4
CADM3_EPB41L1
CADM3_NECTIN3
NTN1_DCC
NTN1_DSCAM
NTN1_UNC5A
NTN1_UNC5C
NTN4_NTRK2
NTN4_UNC5D
